# UV‐Induced 1,3,4‐Oxadiazole Formation from 5‐Substituted Tetrazoles and Carboxylic Acids in Flow

**DOI:** 10.1002/chem.202002896

**Published:** 2020-10-12

**Authors:** Luke Green, Keith Livingstone, Sophie Bertrand, Simon Peace, Craig Jamieson

**Affiliations:** ^1^ Department of Pure and Applied Chemistry University of Strathclyde 295 Cathedral Street Glasgow G1 1XL UK; ^2^ GlaxoSmithKline Medicines Research Centre Gunnels Wood Road Stevenage Hertfordshire SG1 2NY UK

**Keywords:** flow, heterocycles, Huisgen reaction, nitrile imines, photochemistry

## Abstract

A range of 1,3,4‐oxadiazoles have been synthesized using a UV‐B activated flow approach starting from carboxylic acids and 5‐substituted tetrazoles. The application of UV light represents an attractive alternative to the traditional thermolytic approach and has demonstrated comparable efficiency and versatility, with a diverse substrate scope, including the incorporation of highly substituted amino acids.

## Graphical abstract

**A photochemical, flow‐based** Huisgen synthesis of 1,3,4‐oxadiazoles from 5‐substituted tetrazoles and carboxylic acids has been developed. This has enabled the expedient and facile synthesis of a broad palette of heterocyclic derivatives which are of direct relevance to a range of applications including pharmaceuticals and materials science (see scheme).

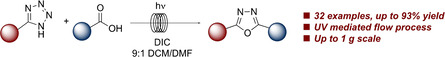

1,3,4‐Oxadiazole derivatives have found a broad spectrum of uses, ranging from pharmacophores with antibacterial, anti‐inflammatory and anticancer properties,[^1^, ^2^] to electron transporting materials in organic light emitting diodes (OLEDS).[^3^, ^4^] The functionality has also found use as a bioisostere for carboxylic acids, esters and amides in medicinal chemistry, and exhibits favorable physicochemical properties when compared to the related 1,2,4‐isomer.[^5^, ^6^, ^7^]

It is unsurprising, therefore, that a range of synthetic approaches towards substituted 1,3,4‐oxadiazoles have been reported utilizing many different reagents. Common approaches include: oxidative cyclisation, cyclodesulfurization, cyclodehydration, condensation and the Huisgen reaction (Scheme 1 a).[^1^, ^8^] Whilst the above methods are effective, most require the synthesis of bespoke starting materials with both the 2‐ and 5‐substituents pre‐installed. Furthermore, the reaction conditions are often harsh, utilizing reagents which may not be compatible with preparations at scale.

**Scheme 1:**
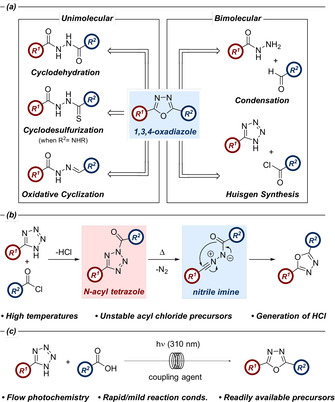
(a) Common approaches towards 2,5‐disubstituted 1,3,4‐oxadiazoles. (b) Mechanism of the Huisgen reaction which proceeds via acyltetrazole and nitrile imine intermediates. (c) This work: 1,3,4‐oxadiazole synthesis from photochemical activation of *N*‐acyl tetrazoles in flow.

The Huisgen reaction between a tetrazole and an acylating reagent is one approach in which two separate components can be combined in a single step to furnish the desired 1,3,4‐oxadiazole.[^9^, ^10^, ^11^, ^12^] Initial reaction between the tetrazole and acyl chloride or acid anhydride yields a 2‐acyltetrazole intermediate which decomposes in a concerted manner to the corresponding nitrile imine upon heating. This dipole subsequently undergoes a 1,5‐dipolar electrocyclization to form the desired 1,3,4‐oxadiazole (Scheme 1 b).^[11]^ The ready availability of both tetrazoles and carboxylic acids renders this an attractive method of oxadiazole synthesis without the need to synthesize several intermediates. Despite this, there are only a paucity of literature examples, likely due to the high temperatures required to generate the nitrile imine.^[13]^ Furthermore, even fewer examples have described the application of carboxylic acids as the acylating agent, with all but one of these using DCC as the coupling mediator.[^14^, ^15^, ^16^, ^17^, ^18^, ^19^]

In addition to this, there are no literature reports where light, rather than heat, is used to form the nitrile imine from the acyl tetrazole precursor. This approach should have several benefits, including improved reaction selectivity through careful selection of the wavelength. As such, any potential by‐product formation related to the thermal instability of tetrazoles and nitrile imines, such as nitriles or heterocyclic compounds,^[20]^ may be reduced in comparison to a thermal reaction.[^21^, ^22^] In the current study, we describe a synthesis of 1,3,4‐oxadiazoles using UV‐B light as an alternative method for nitrile imine formation (Scheme 1 c). This approach also adopts carboxylic acids as acylating reagents, thus avoiding the complications often associated with the use of acid chlorides. A preliminary investigation into the proposed approach found that the use of a UV‐B lamp initiated the Huisgen reaction between **1** and **2** in an analogous manner to the thermal counterpart. The reaction was found to be extremely effective, furnishing an improved yield compared to the thermal process (Scheme 2).

**Scheme 2:**
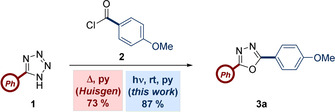
Reaction between 5‐phenyl‐1*H*‐tetrazole (**1**) and 4‐methoxybenzoyl chloride (**2**) using thermal and photochemical activation on 0.25 mmol scale with 2 equiv of acid chloride and 3 equiv pyridine.

Given this result, the possibility of exchanging the acyl chloride for the corresponding carboxylic acid was examined as this was regarded as being generally superior from a practical perspective. Amide coupling reagents were then screened in the batch reaction between **1** and **4** (Table 1).

**Table 1:** Screen of amide coupling reagents for the reaction between 5‐phenyl‐1*H*‐tetrazole (**1**) and 4‐methoxybenzoic acid (**4**) under UV‐B irradiation.^[a]^

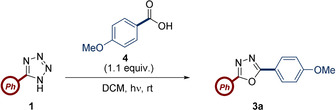				
Entry	Coupling reagent(s)	Solvent	*t* [h]	Yield [%]
1	HATU/DIPEA	DCM	8	N.R.
2	HATU/DIPEA	DMF	21	N.R.
3	PyBOP^®^/DIPEA	DCM	6	N.R.
4	DCC	DCM	19	51
5	PyBrOP^®^/DIPEA	DCM	19	N.R.
6^[b]^	CDI/DMAP	DCM	19	N.R.
7	EDCI/DMAP	DCM	19	4
8	DIC	DCM	23	50
9	DCC/DMAP	DCM	5	55
10^[c]^	DCC	DCM	19	50
11^[c]^	DIC	DCM	19	50
**12^[d]^**	**DIC**	**DCM/DMF (9:1)**	**1**	**85**
13^[d]^	None	DCM/DMF (9:1)	1	N.R.
14^[e]^	DIC	DCM/DMF (9:1)	1	4 (impure)

[a] Reactions carried out in quartz round‐bottom flasks in front of a UV‐B lamp at rt with 1.1 equiv of coupling reagent and at a 0.34 mmol scale of tetrazole. [b] Irradiated for 6.5 h before addition of DMAP and further irradiation for 12.5 h. [c] Scale‐up of reaction (1.37 mmol). [d] Reaction carried out in flow (0.39 mmol of tetrazole). [e] Reaction pumped through flow system with no UV light.

Of the reagents considered, only carbodiimides DCC and diisopropylcarbodiimide (DIC) afforded any significant conversion (Table 1, entries 4 and 8), with others showing little or no reactivity. Combining DCC with DMAP reduced the reaction time but did not significantly improve the yield (Table 1, entry 9). Increasing the scale of the reaction using either DCC or DIC (Table 1, entries 10 and 11) confirmed that both were equally as effective under these conditions.

However, it was noted that less precipitate was formed when using DIC due to the differences in solubility of the corresponding urea by‐products.^[23]^ This observation was important when considering the next stage of optimization, as we envisaged translation of the reaction into a flow process. This was anticipated to maximize the light‐uptake of the reaction mixture through an increase in interfacial surface area which had been shown to reduce the reaction time in previous work.[^24^, ^25^, ^26^, ^27^, ^28^] Clearly, a build‐up of precipitate would hinder the integrity of the flow system and irradiation. To reduce this risk, the concentration was reduced from 0.12 m to 0.04 m; however, the initial attempt was unsuccessful with a precipitate obstructing the flow system. Accordingly, the reaction solvent was modified to a 9:1 DCM/DMF mixture which was able to solubilize both reagents and the diisopropylurea by‐product (Table 1, entry 12). Pleasingly, the first application of these new conditions indicated the reaction was complete after 1 hour in a significantly improved yield of 85 %. The corresponding control reactions confirmed the necessity of DIC for formation of **3 a** (Table 1, entry 13), and the absence of UV light afforded only 4 % of product which appeared to be contaminated by diisopropylurea. Furthermore, a significant amount of the acid anyhydride by‐product was isolated with both starting materials also present after 1 hour.

The flow system consisted of a peristaltic pump, PTFE tubing coiled around a quartz chamber, with a reactor volume of 3.5 mL, placed over the UV‐B lamp, analogous to the reactor described by Booker‐Milburn and Berry,^[29]^ and a round‐bottom flask containing the reaction mixture. This is a straightforward configuration that can be readily assembled by most laboratories. The reaction mixture, generally at 0.34 mmol scale with respect to 5‐phenyl‐1*H*‐tetrazole, was continuously pumped through the system over 1 hour to enable complete conversion to the desired 1,3,4‐oxadiazoles.

With optimized flow conditions identified, the scope of the carboxylic acid was explored (Scheme 3). Yields of up to 93 % were obtained for a broad range of substrates, including both electron‐rich (*cf*. **3 a**, **3 m**) and electron‐poor aromatic carboxylic acids (*cf*. **3 f**, **3 h**). *Ortho*‐, *meta*‐ and *para*‐methyl substituted benzoic acids **3 b**, **3 g** and **3 l** all furnished highly acceptable yields, highlighting the steric tolerance of the process. Heteroaromatics such as furan‐containing oxadiazole **3 d** and pyridine‐containing oxadiazole **3 i** were also tolerated in yields of 54 % and 39 %, respectively.

**Scheme 3:**
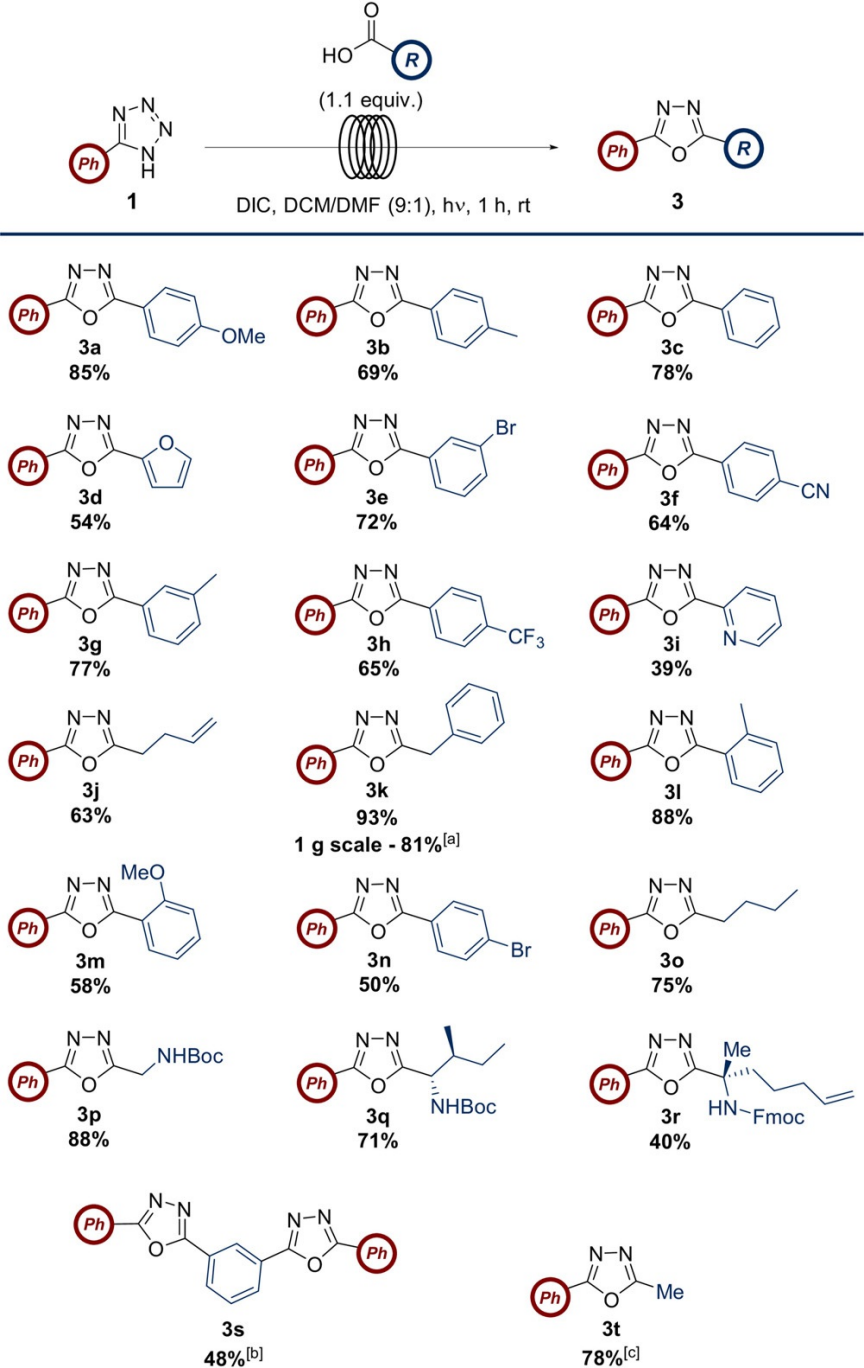
Reaction of 5‐phenyl‐1*H*‐tetrazole (0.34 mmol) with carboxylic acids (1.1 equiv). [a] 4.5 h reaction time. [b] 2 h reaction time, with the acid as the limiting reagent using 2.2 equiv of 5‐phenyl‐1*H*‐tetrazole and DIC. [c] Ac_2_O (2.0 equiv) in dimethoxyethane.

In addition, three aliphatic acids generated oxadiazoles **3 j**, **3 k**, and **3 o** in yields ranging from 63 % to 93 %. In the case of **3 j** no cycloaddition by‐products between the olefin of pentenoic acid and the intermediate nitrile imine were observed, indicating the favorability of the 1,5‐rearrangement. Also of note was the gram‐scale synthesis of **3 k**, with only modest reduction of yield. Importantly, nitrogen‐protected amino acids (**3 p**, **3 q**, **3 r**) were also compatible with these conditions, giving the desired oxadiazoles in yields of 40–88 %, with increasing substitution at the α‐position resulting in lower yields, consistent with the increase in steric demands. Again, no by‐products from the pendant alkene of (*S*)‐*N*‐Fmoc‐α‐4‐n‐pentenylalanine were observed. Furthermore, no evidence of epimerization was noted when using Boc‐Ile‐OH as a substrate. Isophthalic acid could be subjected to the flow conditions with slightly altered stoichiometry and reaction time to yield *bis*‐oxadiazole **3 s**. The formation of two oxadiazoles in conjugation with the same aromatic ring has found application in the synthesis of electron transporting materials in OLEDs.^[30]^ Finally, acetic anhydride could be used as the acylating agent to yield oxadiazole **3 t** with the use of previously optimized flow conditions.^[31]^

With a library of carboxylic acids compatible with the reaction conditions suitably exemplified, the scope of the tetrazole constituent was then explored (Scheme 4). These substrates were subjected to the reaction conditions, with both electron‐rich (*cf*. **5 a**, **5 b**) and electron‐poor aromatics (*cf*. **5 e**, **5 i**) performing well, giving yields in the range of 59–82 %.

**Scheme 4:**
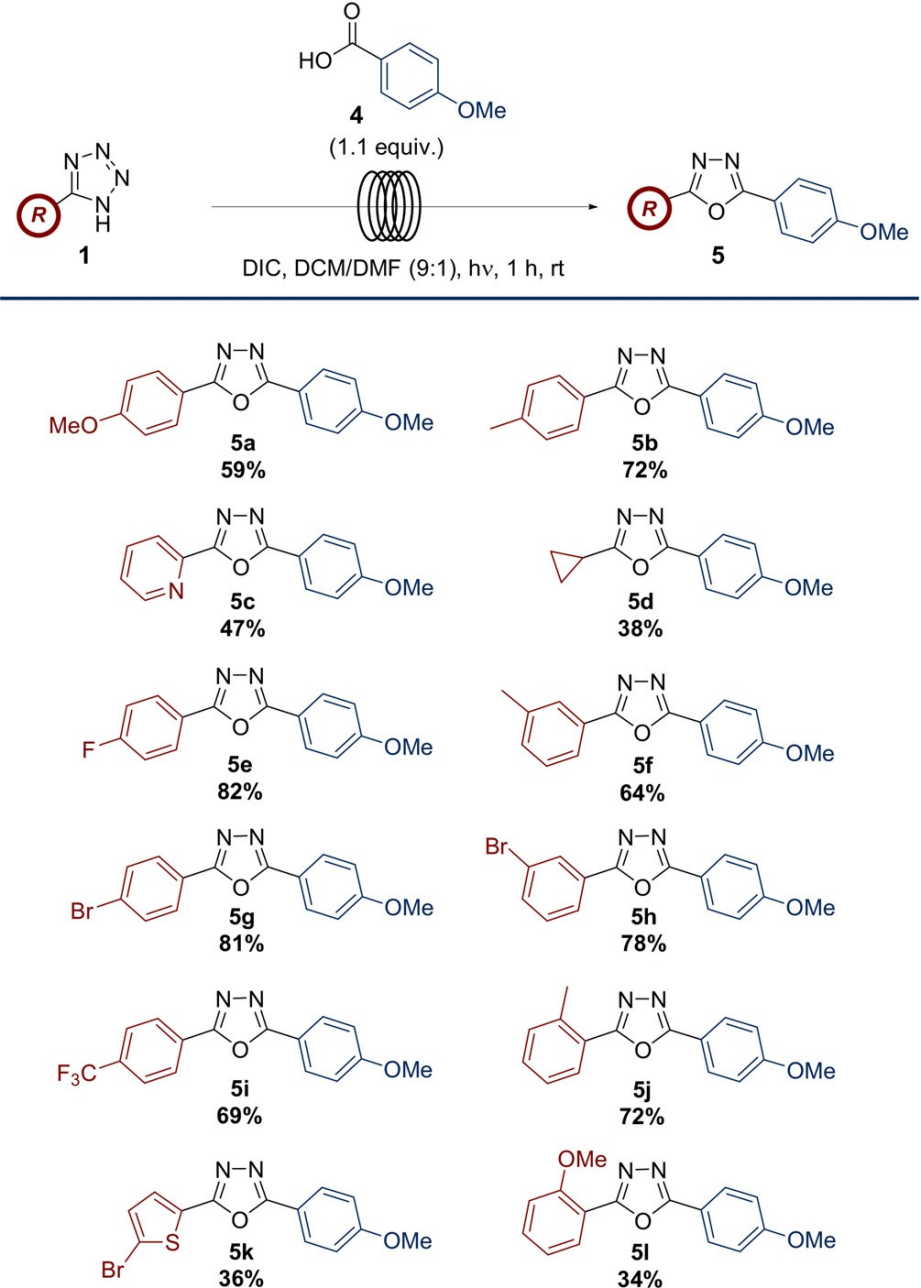
Reaction of 4‐methoxybenzoic acid (1.1 equiv) with tetrazoles on scales of between 0.15–0.34 mmol of tetrazole.

*Ortho*‐methoxy substituted aryl tetrazole **5 l**, which generated the corresponding oxadiazole in a yield of 34 % represents one of two distinctly lower yields. A possible explanation could be that the added electron density from the methoxy group, combined with the steric encumbrance due to its proximity to the reactive center could diminish the efficiency of the rearrangement process. This aligns with previous precedent that 1,3‐dipolar cycloadditions of diaryl nitrile imines are not always completely concerted, and that the electronic nature of the *C*‐aryl ring of the tetrazole may influence the rate of decomposition.[^32^, ^33^] This hypothesis could also explain the lower yield obtained for the electron‐rich thiophene‐containing oxadiazole **5 k**, while electron‐poor substrates were generally obtained in higher yields.

5‐substituted alkyl tetrazoles were examined, however, only the cyclopropyl oxadiazole **5 d** was formed in significant quantities. Figure 1 indicates additional substrates where very little or no reaction was observed. This may be due to a shift in the maximum wavelength of absorption (*λ*_max_) of the tetrazoles, due to the presence of alkyl rather than aromatic groups. As such, the energy of UV‐B light may not be sufficient to enable formation of the nitrile imine and therefore the oxadiazole. The successful formation of oxadiazole **5 d** may be attributed to the interaction between the π‐like orbitals of the cyclopropyl ring^[34]^ with those of the tetrazole, which may lower the *λ*_max_ in an analogous manner to an aromatic substituent.

**Figure 1:**
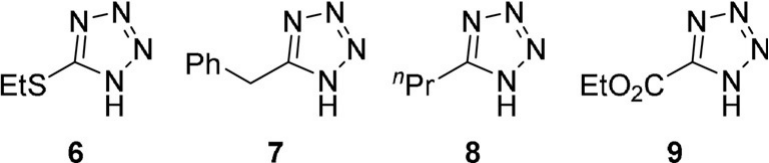
Unsuccessful tetrazole substrates.

To further exemplify this emerging method, the COX‐2 inhibitor **11** ^[35]^ was synthesized from the corresponding carboxylic acid **10** and tetrazole **1** (Scheme 5). This compound was obtained in an excellent yield of 85 %, which is comparable to other reported preparations.[^35^, ^36^]

**Scheme 5:**
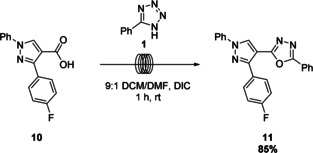
Synthesis of a COX‐2 inhibitor on 0.34 mmol scale with respect to 5‐phenyl‐1*H*‐tetrazole.

In conclusion, the current study has robustly demonstrated that UV‐promotion of the Huisgen reaction for the synthesis of 1,3,4‐oxadiazoles is a valuable direct alternative for the traditional thermolytic process. When incorporated into a simple flow chemistry manifold, this method is amenable to scale up, and enables the synthesis of a broad palette of valuable oxadiazole analogues from readily available precursors in a highly efficient manner. This represents a significant advancement in the light‐mediated synthesis of this fundamentally important heterocyclic template.

## Conflict of interest

The authors declare no conflict of interest.

## Supporting information

As a service to our authors and readers, this journal provides supporting information supplied by the authors. Such materials are peer reviewed and may be re‐organized for online delivery, but are not copy‐edited or typeset. Technical support issues arising from supporting information (other than missing files) should be addressed to the authors.

Supplementary
