# Supplementary material for: UV‐Induced 1,3,4‐Oxadiazole Formation from 5‐Substituted Tetrazoles and Carboxylic Acids in Flow
Source: Chemistry. 2020 Oct 12;26(65):14866–70. doi: 10.1002/chem.202002896 (PMC7756889; doi:10.1002/chem.202002896)
Supplement: Supplementary file 1 — Supplementary [file CHEM-26-14866-s001.pdf]

Chemistry–A European Journal

Supporting Information

UV-Induced 1,3,4-Oxadiazole Formation from 5-Substituted Tetrazoles and Carboxylic Acids in Flow

Luke Green,[a, b] Keith Livingstone,[a] Sophie Bertrand,[b] Simon Peace,[b] and Craig Jamieson*[a]

Supporting Information

UV-Induced 1,3,4-Oxadiazole Formation from 5-Substituted Tetrazoles and Carboxylic Acids in Flow

Luke Green,[a,b] Keith Livingstone,[a] Sophie Bertrand,[b] Simon Peace,[b] Craig Jamieson*[a]

[a] Department of Pure and Applied Chemistry, University of Strathclyde, 295 Cathedral Street, Glasgow, G1 1XL, UK.

[b] GlaxoSmithKline Medicines Research Centre, Gunnels Wood Road, Stevenage, Hertfordshire, SG1 2NY, UK.

Contents

1. General Information. 2
2. Lamp Spectrum..3
3. Optimization Experiments..4
4. Flow Set-up..9
5. General Procedure and Product Characterization..11
6. References 29
8. Spectra 30
9. LCMS and HRMS Parameters 143

1. General Information.

Chemicals were used as received from commercial sources (Sigma Aldrich, Fluorochem, Alfa Aesar) without purification, unless otherwise stated. NMR spectra were recorded on a Bruker AV 500 or a Bruker AV 400. Chemical shifts (δ) are reported in ppm and coupling constants (*J*) are in Hz. The following abbreviations are used for multiplicities: s = singlet; br s = broad singlet; d = doublet; t = triplet; q = quartet; m = multiplet; dd = doublet of doublets; dt = doublet of triplets; spt = septet. Liquid Chromatography Mass Spectrometry (LCMS) methods used for reaction monitoring and final purity analysis are referred to by the modifier used (formic acid or high pH). The analysis was conducted on an Acquity UPLC CSH C18 column (50 mm x 2.1 mm i.d. 1.7µm packing diameter) at 40 °C using a 2-minute method (see Section 9 for gradients and solvents). Mass spectra were recorded using a Waters QDA with an alternate-scan positive and negative electrospray ionization with a range of 100-1500 AMU and a frequency of 5 Hz. The UV detection was a summed signal from 210 nm to 350 nm. High Resolution Mass Spectrometry (HRMS) was obtained using a UPLC-HRMS system. The chromatography was conducted on an Acquity UPLC BEH or UPLC CSH C18 column (100mm x 2.1mm i.d. 1.7µm packing diameter) at 50 °C in either a formic acid or high pH modifier (see Section 9 for gradients and solvents). The UV detection was a summed signal from 210 nm to 500 nm. The HRMS were recorded using a Waters XEVO G2-XS Qtof with positive electrospray ionization mode with a scan range of 100 to 1200 AMU. IR spectra were obtained on a Perkin Elmer Spectrum One spectrometer. Absorption frequencies (ν_{max}) are reported in wavenumbers (cm^{-1}). Photochemical flow reactions were carried out in PTFE tubing (0.7 x 1.6 mm, sourced from ADTECH) wrapped around a quartz tube with diameter of 3.5 cm. The bulb used was a Philips PL-S 9W/12 with a range of 290-315 nm. A Pharmacia Fine Chemicals peristaltic pump was used to pump the reaction mixture through the tubing at a flow rate of 2 mL min^{-1}. The reactor volume was measrured to be 3.5 mL.

2. Lamp Spectrum

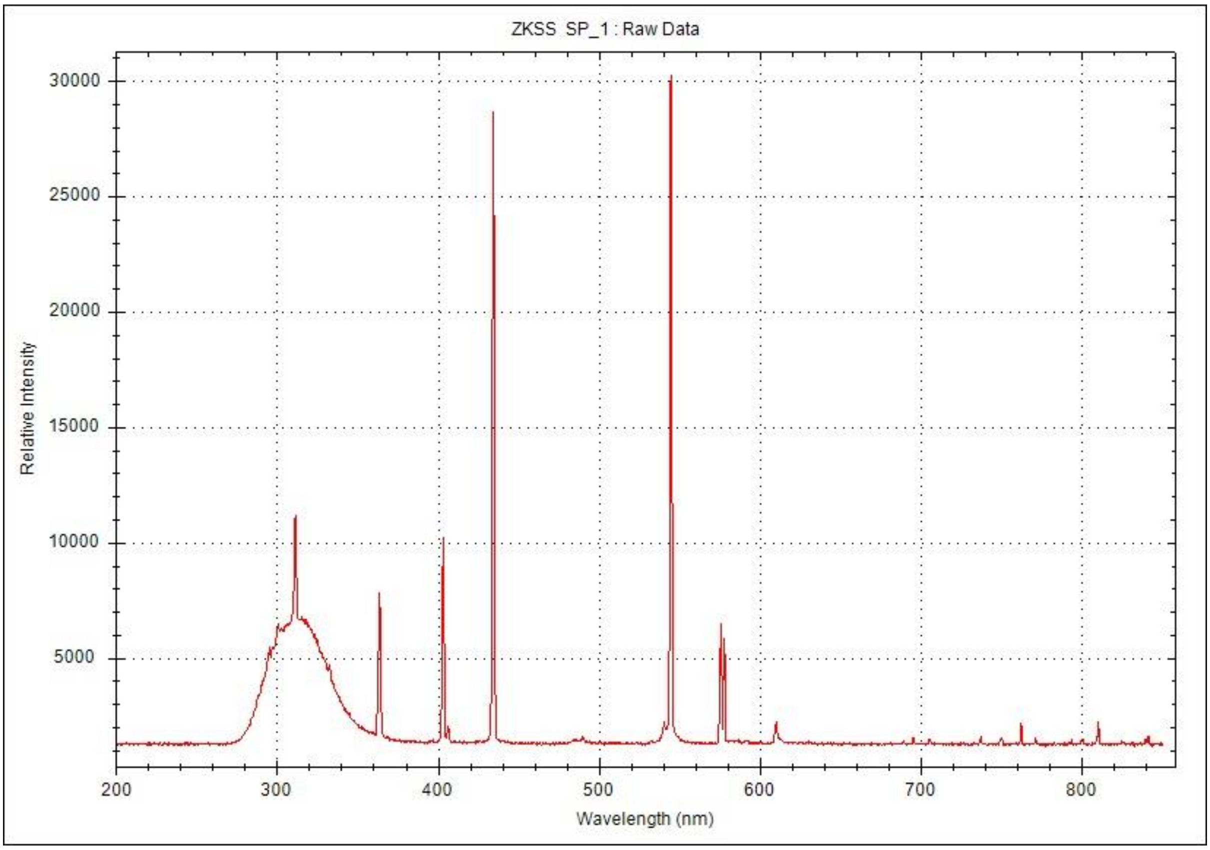

3. Optimization Experiments

Table 1. Screen of amide coupling reagents for the reaction between 5-phenyl-1*H*-tetrazole (**1**) and 4-methoxybenzoic acid (**4**) under UV-B light.

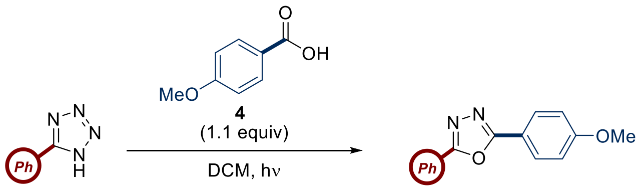

Entry	Coupling Reagent(s)	Solvent	Time (h)	Yield (%)
1	HATU/DIPEA	DCM	8	N.R.
2	HATU/DIPEA	DMF	21	N.R.
3	PyBOP®/DIPEA	DCM	6	N.R.
4	DCC	DCM	19	51
5	PyBrOP®/DIPEA	DCM	19	N.R.
6[a]	CDI/DMAP	DCM	19	N.R.
7	EDCI/DMAP	DCM	19	4
8	DIC	DCM	23	50
9	DCC/DMAP	DCM	5	55
10[b]	DCC	DCM	19	50
11[b]	DIC	DCM	19	50
**12[c]**	**DIC**	**DCM/DMF (9:1)**	**1**	**85**
13[c]	None	DCM/DMF (9:1)	1	N.R.
14[d]	DIC	DCM/DMF (9:1)	1	4% (impure)

Reactions carried out in quartz round-bottom flasks in front of a UV-B lamp. [a] Irradiated for 6.5 h before addition of DMAP and further irradiation for 12.5 h. [b] Scale-up of reaction (1.4 mmol). [c] Reaction carried out in flow. [d] Reaction pumped through flow system with no UV light.

Entry 1.

4-Methoxybenzoic acid (60 mg, 0.394 mmol) and HATU (143 mg, 0.376 mmol) were dissolved in DCM (1.4 mL). DIPEA (179 µL, 1.03 mmol) was added and the reaction mixture stirred for 15 min at rt. 5-Phenyl-1*H*-tetrazole (55 mg, 0.376 mmol) was added and the reaction mixture irradiated for 8 h. Only starting materials were observed by TLC with no product visible when compared to an authentic sample.

Entry 2.

4-Methoxybenzoic acid (60 mg, 0.394 mmol) and HATU (162 mg, 0.426 mmol) were dissolved in DMF (1.4 mL) before DIPEA (179 µL, 1.03 mmol) was added and the reaction mixture stirred for 15 min at rt. 5-Phenyl-1*H*-tetrazole (50 mg, 0.342 mmol) was added and the reaction mixture irradiated for 21 h before being diluted with EtOAc (10 mL) and 1M aqueous NaOH (10 mL). The layers were separated and the organic washed with water (10 mL) and 5% aqueous LiCl solution (2 × 10 mL). The organic was collected, dried using a hydrophobic frit and concentrated *in vacuo* to give an orange residue. NMR and TLC of the crude mixture showed no desired product when compared to an authentic sample.

Entry 3.

5-Phenyl-1*H*-tetrazole (50 mg, 0.342 mmol), 4-methoxybenzoic acid (60 mg, 0.394 mmol) and PyBOP® (196 mg, 0.376 mmol) were dissolved in DCM (1.4 mL). DIPEA (179 µL, 1.03 mmol) was added and the reaction mixture irradiated for 6 h before being concentrated *in vacuo* giving a brown oil which was dissolved in EtOAc (10 mL). This was washed with water (10 mL) and sat. aqueous ammonium chloride solution (10 mL) before being concentrated *in vacuo* to give a brown residue. TLC of this showed no desired product when compared to an authentic sample.

Entry 4. ***Reaction diluted due to solubility of DCC***

5-Phenyl-1*H*-tetrazole (50 mg, 0.342 mmol), 4-methoxybenzoic acid (66 mg, 0.434 mmol) and DCC (78 mg, 0.376 mmol) were dissolved in DCM (2.8 mL). The reaction mixture was stirred at rt for 15 min before being irradiated for 19 h. TLC indicated that the desired product had formed when compared to an authentic sample. Therefore, the reaction mixture was concentrated *in vacuo* giving a white solid which was suspended in EtOAc (5 mL) and filtered. The collected solid was washed with further EtOAc. The filtrate was concentrated *in vacuo* giving a white solid which was purified by flash column chromatography (0-0.5% MeOH/DCM) to give the desired product **3a** as a white solid (44 mg, 0.175 mmol, 51%).

Entry 5.

5-Phenyl-1*H*-tetrazole (52 mg, 0.356 mmol), 4-methoxybezoic acid (62 mg, 0.407 mmol) and PyBrOP® (201 mg, 0.431 mmol) were dissolved in DCM (1.4 mL). DIPEA (179 µL, 1.03 mmol) was added and the reaction stirred for 15 min at rt before being irradiated for 19 h. The reaction mixture was concentrated *in vacuo* to give a yellow oil. This was dissolved in EtOAc (10 mL) and washed with water (10 mL) and sat. aqueous ammonium chloride (10 mL). The organic was collected, dried using a hydrophobic frit and concentrated *in vacuo* to give a yellow oil with some solid present. TLC and NMR of this did not show any desired product when compared to an authentic sample.

Entry 6. ***Reaction diluted due to solubility of CDI***

5-Phenyl-1*H*-tetrazole (53 mg, 0.363 mmol), 4-methoxybenzoic acid (60 mg, 0.394 mmol) and CDI (65 mg, 0.401 mmol) were dissolved in DCM (2.8 mL) and the reaction mixture irradiated for 6.5 h. TLC showed no desired product had formed when compared to an authentic sample. DMAP (70 mg, 0.572 mmol) was added and the reaction mixture irradiated for a further 12.5 h before being diluted with DCM (10 mL). This was washed with water (10 mL) and 1M aqueous HCl (10 mL). The organic was collected, dried using a hydrophobic frit and concentrated *in vacuo* giving a pale yellow oil. TLC and NMR of this showed no desired product had formed when compared to an authentic sample.

Entry 7. ***Reaction diluted due to solubility of EDCI***

5-Phenyl-1*H*-tetrazole (50 mg, 0.342 mmol), 4-methoxybenzoic acid (60 mg, 0.394 mmol), EDCI (72 mg, 0.376 mmol) and DMAP (63 mg, 0.513 mmol) were dissolved in DCM (2.8 mL) and the reaction mixture irradiated for 19 h. This was diluted with DCM (10 mL) and washed with water (10 mL) and brine (10 mL). The organic was collected, dried using a hydrophobic frit and concentrated *in vacuo* to give an off-white residue which was purified by flash column chromatography (0-0.5% MeOH/DCM). **3a** was obtained as an off-white solid (3.2 mg, 0.013 mmol, 4%).

Entry 8. ***Reaction diluted as a direct comparison to DCC reaction***

5-phenyl-1*H*-tetrazole (53 mg, 0.363 mmol) and 4-methoxybenzoic acid (63 mg, 0.414 mmol) were dissolved in DCM (2.8 mL) and DIC (59 µL, 0.376 mmol) was added. The

reaction mixture irradiated for 23 h before being diluted with DCM (10 mL). This was washed with water (10 mL) and 1M NaOH (10 mL). The organic was collected, dried using a hydrophobic frit and concentrated *in vacuo* to give a white solid which was purified by flash column chromatography (0-0.5% MeOH/DCM) to give **3a** as an off-white solid (46 mg, 0.182 mmol, 50%).

Entry 9.

5-Phenyl-1*H*-tetrazole (67 mg, 0.458 mmol), 4-methoxybenzoic acid (77 mg, 0.504 mmol), DCC (104 mg, 0.504 mmol) and DMAP (6 mg, 0.049 mmol) were dissolved in DCM (3.7 mL) and the reaction mixture irradiated for 5 h before being concentrated *in vacuo*. The remaining white solid was suspended in EtOAc (5 mL) and filtered. The solid was washed with further EtOAc and the filtrate collected and concentrated *in vacuo* to give a white solid which was purified by flash column chromatography (0-0.5% MeOH/DCM) to give **3a** as a white solid (64 mg, 0.254 mmol, 55%).

Entry 10. ***Scale-up of DCC reaction***

5-Phenyl-1*H*-tetrazole (200 mg, 1.37 mmol), 4-methoxybenzoic acid (229 mg, 1.51 mmol) and DCC (311 mg, 1.51 mmol) were dissolved in DCM (11.2 mL) and the reaction mixture irradiated for 19 h before being concentrated *in vacuo*. The remaining white solid was suspended in EtOAc (10 mL) and filtered. The solid was washed with further EtOAc and the filtrate collected and concentrated *in vacuo* to give a white solid which was purified by flash column chromatography (0-0.5% MeOH/DCM) to give **3a** as an off-white solid (171 mg, 0.678 mmol, 50%).

Entry 11. ***Scale-up of DIC reaction***

5-Phenyl-1*H*-tetrazole (200 mg, 1.37 mmol), 4-methoxybenzoic acid (229 mg, 1.51 mmol) and DIC (0.235 µL, 1.51 mmol) were dissolved in DCM (11.2 mL) and the reaction mixture irradiated for 19 h before being concentrated *in vacuo*. The remaining white solid was suspended in EtOAc (10 mL) and filtered. The solid was washed with further EtOAc and the filtrate collected and concentrated *in vacuo* to give a white solid which was purified by flash column chromatography (0-0.5% MeOH/DCM) to give **3a** as an off-white solid (171 mg, 0.678 mmol, 50%).

Entry 12. ***DIC Reaction in flow system***

5-Phenyl-1*H*-tetrazole (57 mg, 0.390 mmol) and 4-methoxybenzoic acid (62 mg, 0.407 mmol) were dissolved in DCM/DMF (9:1, 8.4 mL). DIC (64 µL, 0.429 mmol) was added and the reaction mixture pumped through the flow system for 1 h. The reaction mixture was then collected and concentrated *in vacuo* before being dissolved in EtOAc (20 mL). This was washed with water (10 mL) and 5% aqueous LiCl solution (2 × 20 mL). The organic layer was collected, dried using a hydrophobic frit and concentrated *in vacuo*. The resulting residue was purified by flash column chromatography (0-0.5% MeOH/DCM) to give **3a** as a white solid (84 mg, 0.333 mmol, 85%).

Entry 13. ***Control reaction without DIC***

5-Phenyl-1*H*-tetrazole (50 mg, 0.342 mmol) and 4-methoxybenzoic acid (57 mg, 0.376 mmol) were dissolved in DCM/DMF (9:1, 8.4 mL) and the reaction mixture pumped through the flow system for 1 h. The reaction mixture was then collected and concentrated *in vacuo* before being dissolved in EtOAc (20 mL). This was washed with water (10 mL) and 5% aqueous LiCl solution (2 × 20 mL). The organic layer was collected, dried using a hydrophobic frit and concentrated *in vacuo*. NMR and TLC of the crude material showed no desired product when compared to an authentic sample.

Entry 14. ***Control reaction in the dark***

5-Phenyl-1*H*-tetrazole (50 mg, 0.342 mmol) and 4-methoxybenzoic acid (57 mg, 0.376 mmol) were dissolved in DCM/DMF (9:1, 8.4 mL). DIC (64 µL, 0.429 mmol) was added and the reaction mixture pumped through the flow system for 1 h in the dark. The reaction mixture was then collected and concentrated *in vacuo* before being dissolved in EtOAc (20 mL). This was washed with water (10 mL) and 5% aqueous LiCl solution (2 × 20 mL). The organic layer was collected, dried using a hydrophobic frit and concentrated *in vacuo*. The resulting residue was purified by flash column chromatography (0-0.5% MeOH/DCM) to give **3a** as an off-white solid (3.4 mg, 0.013 mmol, 4%). The NMR of the product showed that impurities were also present.

4. Flow Set-up

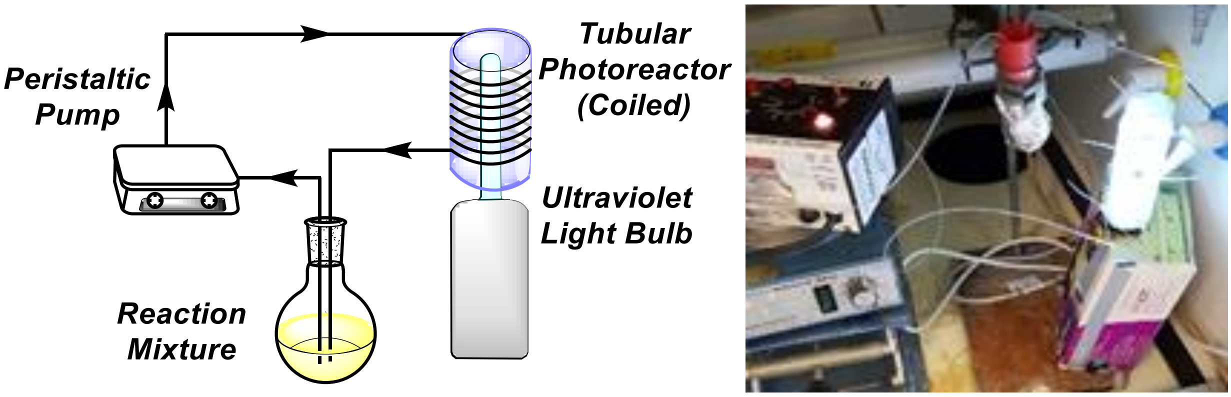

Figure S1. A schematic diagram of the flow set up, containg the UV light bulb and the coiled reactor (3.5 mL) wrapped around it, alongside a photograph of the system used.

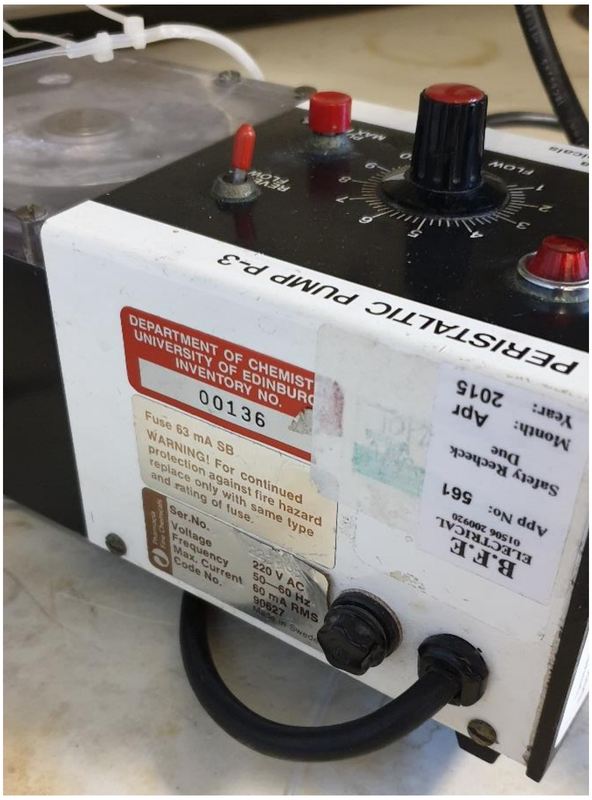

Figure S2. Pharmacia Fine Chemicals peristaltic pump used to pump the reaction mixture through the flow system.

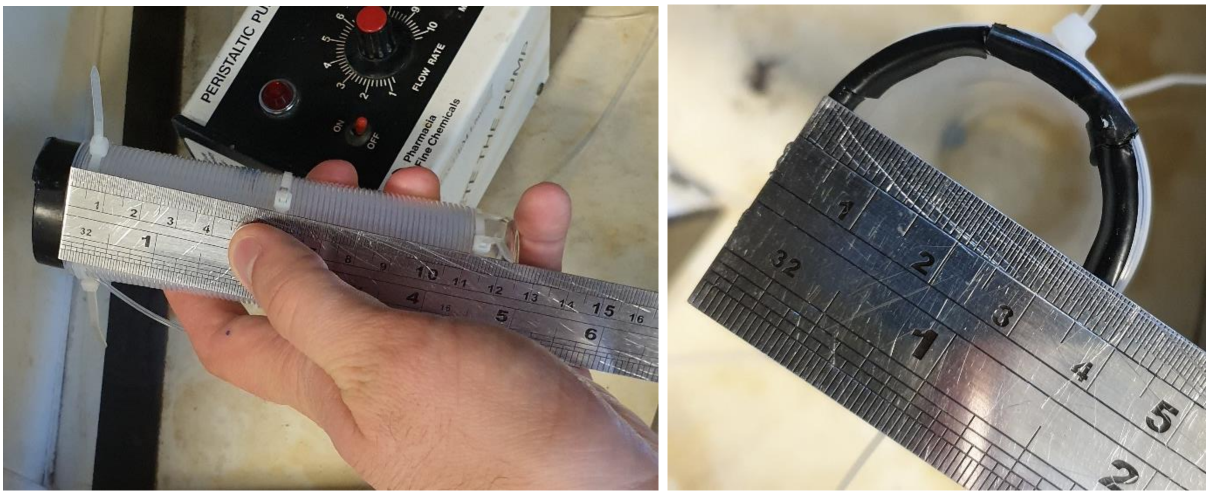

Figure S3. The dimensions of the quartz tube placed over the lamp. The PTFE tubing is wrapped around the outside of the casing and has a reactor volume of 3.5 mL.

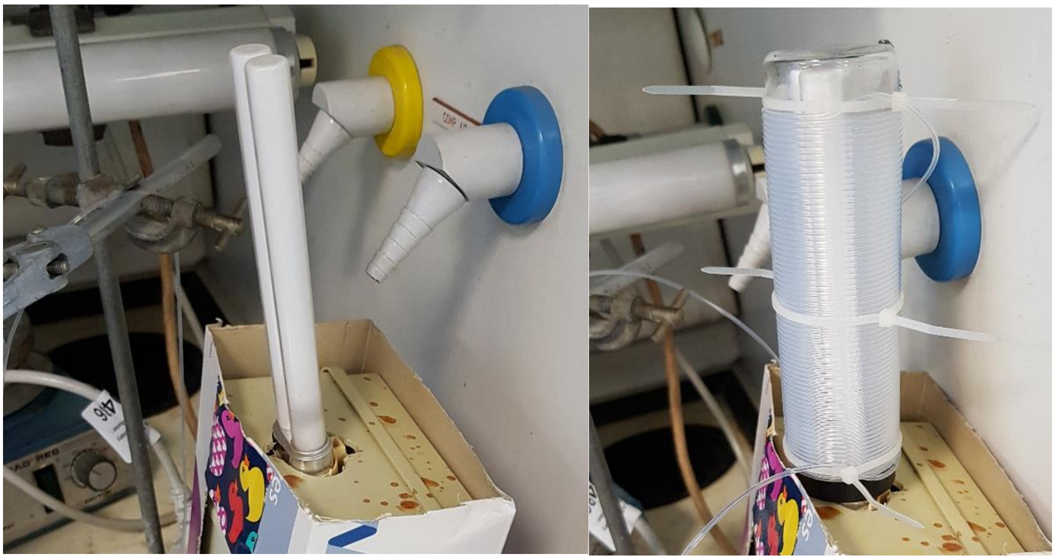

Figure S4. The UV-B lamp used, shown with and without the quartz casing placed over.

5. General Procedure and Product Characterization

2-(4-Methoxyphenyl)-5-phenyl-1,3,4-oxadiazole (3a) – *initial reaction*

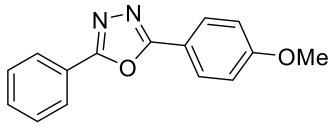

5-Phenyl-1*H*-tetrazole (37 mg, 0.25 mmol), 4-methoxybenzoyl chloride (68 μL, 0.5 mmol) and pyridine (60 μL, 0.75 mmol) were dissolved in toluene (1 mL) and either heated to 100 °C or irradiated with UV-B light until the starting material was consumed. The reaction mixture was purified by flash column chromatography (0-25% EtOAc/petroleum ether) to give the product as a white solid.

Heating: 46 mg, 0.182 mmol, 73%.

UV light: 55 mg, 0.218 mmol, 87%.

General Flow Procedure.

Tetrazole (1 equiv) and carboxylic acid (1.1 equiv) were dissolved in DCM/DMF (9:1, 0.04 M). DIC (1.1 equiv) was added and the reaction mixture pumped through the flow system for 1 h. The reaction mixture was then collected and concentrated *in vacuo* before being dissolved in EtOAc (20 mL). This was washed with water (10 mL) and 5% aqueous LiCl solution (2 × 20 mL). The organic layer was collected, dried using a hydrophobic frit and concentrated *in vacuo*. The resulting residue was purified by flash column chromatography on silica gel.

2-(4-Methoxyphenyl)-5-phenyl-1,3,4-oxadiazole (3a)

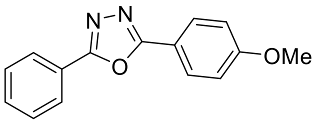

Following the General Flow Procedure using 5-phenyl-1*H*-tetrazole (57 mg, 0.390 mmol), 4-methoxybenzoic acid (62 mg, 0.407 mmol), DIC (64 μL, 0.429 mmol) and 9:1 DCM/DMF

(8.4 mL). Elution with 0-0.5% MeOH/DCM afforded **3a** as a white solid (84 mg, 0.333 mmol, 85%). ^{1}H NMR (400 MHz, $CDCl_3$): δ 8.17 - 8.12 (m, 2H), 8.11 - 8.07 (m, 2H), 7.57 - 7.50 (m, 3H), 7.07 - 7.02 (m, 2H), 3.90 (s, 3H); ^{13}C NMR (101 MHz, $CDCl_3$) δ 164.5, 164.1, 162.3, 131.5, 129.0, 128.7, 126.8, 124.1, 116.5, 114.5, 55.5; LCMS (High pH) t_R = 1.17 mins, [M+H$^+$] 253.0 (99% purity).

3a is a known compound and the NMR data are consistent with reported literature.[1,2]

2-Phenyl-5-(*p*-tolyl)-1,3,4-oxadiazole (3b)

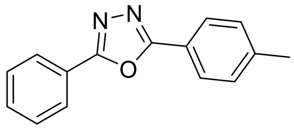

Following the General Flow Procedure using 5-phenyl-1*H*-tetrazole (50 mg, 0.342 mmol), 4-methylbenzoic acid (51 mg, 0.376 mmol), DIC (59 µL, 0.376 mmol) and 9:1 DCM/DMF (8.4 mL). Elution with 0-0.5% MeOH/DCM afforded **3b** as a white solid (56 mg, 0.237 mmol, 69%). ^{1}H NMR (400 MHz, $CDCl_3$) δ 8.17 - 8.13 (m, 2H), 8.04 (dd, *J* = 2.0, 8.5 Hz, 2H), 7.58 - 7.50 (m, 3H), 7.35 (dd, *J* = 1.8, 8.3 Hz, 2H), 2.45 (d, *J* = 2.5 Hz, 3H); ^{13}C NMR (101 MHz, $CDCl_3$) δ = 164.7, 164.3, 142.2, 131.6, 129.7, 129.0, 126.9, 124.0, 121.2, 21.6 1C not observed; LCMS (High pH) t_R = 1.25 mins, [M+H$^+$] 237.0 (97% purity).

3b is a known compound and the NMR data are consistent with reported literature.[3]

2,5-Diphenyl-1,3,4-oxadiazole (3c)

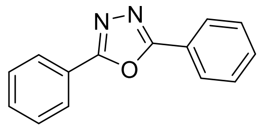

Following the General Flow Procedure using 5-phenyl-1*H*-tetrazole (50 mg, 0.342 mmol), benzoic acid (47 mg, 0.376 mmol), DIC (59 µL, 0.376 mmol) and 9:1 DCM/DMF (8.4 mL). Elution with 0-0.5% MeOH/DCM afforded **3c** as a white solid (59 mg, 0.266 mmol, 78%). ^{1}H NMR (400 MHz, $CDCl_3$) δ = 8.19 - 8.11 (m, 4H), 7.61 - 7.50 (m, 6H); ^{13}C NMR (101

MHz, $CDCl_3$) δ = 164.6, 131.7, 129.1, 126.9, 124.0; LCMS (High pH) t_R = 1.17 mins, [M+H$^+$] 223.0 (purity 100%).

3c is a known compound and the NMR data are consistent with reported literature.[2]

2-(Furan-2-yl)-5-phenyl-1,3,4-oxadiazole (3d)

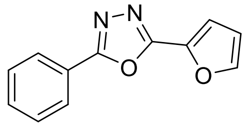

Following the General Flow Procedure using 5-phenyl-1*H*-tetrazole (50 mg, 0.342 mmol), furan-2-carboxylic acid (42 mg, 0.376 mmol), DIC (59 µL, 0.376 mmol) and 9:1 DCM/DMF (8.4 mL). Elution with 0-0.5% MeOH/DCM afforded **3d** as a yellow solid (39 mg, 0.184 mmol, 54%). ^{1}H NMR (400 MHz, $CDCl_3$) δ = 8.13 (dd, *J* = 1.8, 7.8 Hz, 2H), 7.70 - 7.66 (m, 1H), 7.59 - 7.50 (m, 3H), 7.24 (d, *J* = 3.5 Hz, 1H), 6.63 (dd, *J* = 1.5, 3.5 Hz, 1H); ^{13}C NMR (101 MHz, $CDCl_3$) δ = 163.9, 157.4, 145.7, 139.5, 131.8, 129.1, 127.0, 123.5, 114.1, 112.2; LCMS (High pH) t_R = 1.02 mins, [M+H$^+$] 213.0 (purity 100%).

3d is a known compound and the NMR data are consistent with reported literature.[3]

2-(3-Bromophenyl)-5-phenyl-1,3,4-oxadiazole (3e)

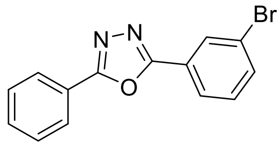

Following the General Flow Procedure using 5-phenyl-1*H*-tetrazole (50 mg, 0.342 mmol), 3-bromobenzoic acid (76 mg, 0.376 mmol), DIC (59 µL, 0.376 mmol) and 9:1 DCM/DMF (8.4 mL). Elution with 5% EtOAc/40-60 °C petroleum ether afforded **3e** as a white solid (74 mg, 0.246 mmol, 72%). ^{1}H NMR (400 MHz, $CDCl_3$) δ = 8.28 (app. t, *J* = 1.8 Hz, 1H), 8.17 - 8.12 (m, 2H), 8.11 - 8.07 (m, 1H), 7.70 - 7.66 (m, 1H), 7.60 - 7.52 (m, 3H), 7.42 (app. t, *J* = 7.8 Hz, 1H); ^{13}C NMR (101 MHz, $CDCl_3$) δ = 164.9, 163.2, 134.6, 131.9, 130.6, 129.7, 129.1, 127.0, 125.7, 125.4, 123.6, 123.1; LCMS (High pH) t_R = 1.32 mins, [M+H$^+$] = 300.9 and 302.9 (purity 100%).

3e is a known compound and the NMR data are consistent with reported literature.[4]

4-(5-Phenyl-1,3,4-oxadiazol-2-yl)benzonitrile (3f)

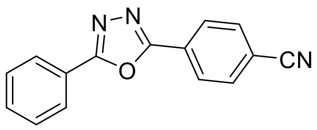

Following the General Flow Procedure using 5-phenyl-1*H*-tetrazole (50 mg, 0.342 mmol), 4-cyanobenzoic acid (55 mg, 0.376 mmol), DIC (59 µL, 0.376 mmol) and 9:1 DCM/DMF (8.4 mL). Elution with 0-0.5% MeOH/DCM afforded **3f** as an off-white solid (54 mg, 0.219 mmol, 64%). ^{1}H NMR (400 MHz, $CDCl_3$) δ = 8.26 (d, *J* = 8.0 Hz, 2H), 8.14 (dd, *J* = 1.3, 7.8 Hz, 2H), 7.84 (d, *J* = 8.5 Hz, 2H), 7.64 - 7.52 (m, 3H); ^{13}C NMR (101 MHz, $CDCl_3$) δ = 165.3, 163.0, 132.8, 132.2, 129.2, 127.7, 127.3, 127.0, 123.3, 117.8, 115.1; LCMS (formic acid) t_R = 1.09 mins, [M+H$^+$] 248.0 (purity 99%).

3f is a known compound and the NMR data are consistent with reported literature.[2]

2-Phenyl-5-(*m*-tolyl)-1,3,4-oxadiazole (3g)

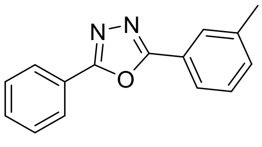

Following the General Flow Procedure using 5-phenyl-1*H*-tetrazole (50 mg, 0.342 mmol), 3-methylbenzoic acid (51 mg, 0.376 mmol), DIC (59 µL, 0.376 mmol) and 9:1 DCM/DMF (8.4 mL). Elution with 2% EtOAc/40-60 °C petroleum ether afforded **3g** as a white solid (62 mg, 0.263 mmol, 77%). ^{1}H NMR (400 MHz, $CDCl_3$) δ = 8.19 - 8.11 (m, 2H), 7.97 (s, 1H), 7.94 (d, *J* = 7.5 Hz, 1H), 7.59 - 7.51 (m, 3H), 7.42 (app. t, *J* = 7.5 Hz, 1H), 7.36 (d, *J* = 7.5 Hz, 1H), 2.46 (s, 3H); ^{13}C NMR (101 MHz, $CDCl_3$) δ = 164.7, 164.5, 138.9, 132.5, 131.6, 129.0, 128.9, 127.4, 126.9, 124.1, 124.0, 123.8, 21.3; LCMS (High pH) t_R = 1.26 mins, [M+H$^+$] 237.0 (purity 100%).

3g is a known compound and the NMR data are consistent with reported literature.[5]

2-Phenyl-5-(4-(trifluoromethyl)phenyl)-1,3,4-oxadiazole (3h)

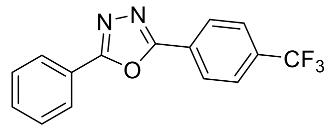

Following the General Flow Procedure using 5-phenyl-1*H*-tetrazole (52 mg, 0.356 mmol), 4-(trifluoromethyl)benzoic acid (72 mg, 0.379 mmol), DIC (59 µL, 0.376 mmol) and 9:1 DCM/DMF (8.4 mL). Elution with 0-0.5% MeOH/DCM afforded **3h** as a white solid (64 mg, 0.221 mmol, 65%). ^{1}H NMR (400 MHz, $CDCl_3$) δ = 8.28 (d, J = 8.0 Hz, 2H), 8.20 - 8.12 (m, 2H), 7.82 (d, J = 8.5 Hz, 2H), 7.63 - 7.52 (m, 3H); ^{19}F NMR (376 MHz, $CDCl_3$) δ = -63.08 (s); ^{13}C NMR (101 MHz, $CDCl_3$) δ = 165.2, 163.4, 133.3 (q, $^{2}J_{CF}$ = 31.9 Hz), 132.1, 129.2, 127.4, 127.2, 127.1, 126.1 (q, $^{3}J_{CF}$ = 3.9 Hz), 123.6, 123.6 (q, $^{1}J_{CF}$ = 272.6 Hz); LCMS (formic acid) t_R = 1.32 mins, [M+H$^+$] 291.0 (purity 95%).

3h is a known compound and the NMR data are consistent with reported literature.[5]

2-Phenyl-5-(pyridin-2-yl)-1,3,4-oxadiazole (3i)

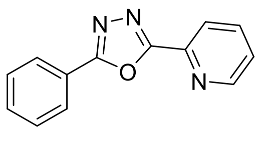

Following the General Flow Procedure using 5-phenyl-1*H*-tetrazole (50 mg, 0.342 mmol), 2-picolinic acid (46 mg, 0.376 mmol), DIC (59 µL, 0.376 mmol) and 9:1 DCM/DMF (8.4 mL). Elution with 0-0.5% MeOH/DCM afforded **3i** as a white solid (30 mg, 0.134 mmol, 39%). ^{1}H NMR (400 MHz, $CDCl_3$) δ = 8.83 (d, J = 4.5 Hz, 1H), 8.33 (d, J = 8.0 Hz, 1H), 8.23 (dd, J = 1.8, 7.8 Hz, 2H), 7.92 (ddd, J = 1.8, 1.8, 7.8 Hz, 1H), 7.60 - 7.51 (m, 3H), 7.49 (ddd, J = 1.0, 5.0, 7.5 Hz, 1H); ^{13}C NMR (101 MHz, $CDCl_3$) δ = 165.6, 163.8, 150.3, 143.6, 137.3, 132.0, 129.0, 127.3, 125.8, 123.6, 123.3; LCMS (High pH) t_R = 0.90 mins, [M+H$^+$] 224.0 (purity 100%).

3i is a known compound and the NMR data are consistent with reported literature.[3,6]

2-(But-3-en-1-yl)-5-phenyl-1,3,4-oxadiazole (3j)

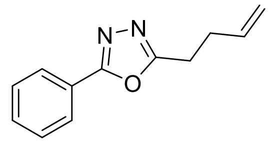

Following the General Flow Procedure using 5-phenyl-1*H*-tetrazole (50 mg, 0.342 mmol), pent-4-enoic acid (38 µL, 0.376 mmol), DIC (59 µL, 0.376 mmol) and 9:1 DCM/DMF (8.4 mL). Elution with 5% EtOAc/40-60 °C petroleum ether afforded **3j** as a pale yellow oil (43 mg, 0.215 mmol, 63%). ^{1}H NMR (500 MHz, $CDCl_3$) δ = 8.07 - 8.02 (m, 2H), 7.56 - 7.48 (m, 3H), 5.91 (tdd, J = 6.4, 10.3, 17.1 Hz, 1H), 5.15 (tdd, J = 1.6, 1.6, 17.2 Hz, 1H), 5.09 (tdd, J = 1.4, 1.4, 10.3 Hz, 1H), 3.05 (t, J = 7.6 Hz, 2H), 2.65 - 2.60 (m, 2H); ^{13}C NMR (101 MHz, $CDCl_3$) δ = 166.3, 164.8, 135.7, 131.5, 129.0, 126.8, 124.0, 116.6, 30.4, 25.0; LCMS (High pH) t_R = 1.05 mins, [M+H$^+$] 201.0 (purity 100%); HRMS (High pH) t_R = 7.72 mins, [M+H$^+$] calculated for $C_{12}H_{13}N_2O$ 201.1022, found 201.1029; IR (neat) ν_{max} = 1572, 1553, 707, 689 cm^{-1}.

2-Benzyl-5-phenyl-1,3,4-oxadiazole (3k)

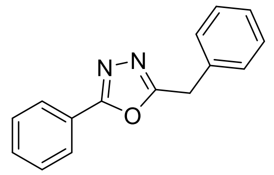

Following the General Flow Procedure using 5-phenyl-1*H*-tetrazole (57 mg, 0.390 mmol), 2-phenylacetic acid (59 mg, 0.433 mmol), DIC (67 µL, 0.433 mmol) and 9:1 DCM/DMF (9.6 mL). Elution with 0-0.5% MeOH/DCM afforded **3k** as a white solid (75 mg, 0.317 mmol, 93%). ^{1}H NMR (400 MHz, $CDCl_3$) δ = 8.05-7.99 (m, 2H), 7.57 - 7.46 (m, 3H), 7.41 - 7.29 (m, 5H), 4.31 (s, 2H); ^{13}C NMR (101 MHz, $CDCl_3$) δ = 165.2, 165.2, 133.9, 131.6, 128.9, 128.9, 128.8, 127.5, 126.8, 123.9, 31.9; LCMS (High pH) t_R = 1.12 mins, [M+H$^+$] 237.0 (purity 100%).

Gram-scale synthesis of 3k. 5-Phenyl-1*H*-tetrazole (1.0 g, 6.84 mmol) and phenylacetic acid (1.02 g, 7.52 mmol) were dissolved in DCM/DMF (9:1, 167 mL). DIC (1.16 mL, 7.52 mmol) was then added and the reaction mixture irradiated in flow for 4.5 h. The reaction mixture was concentrated *in vacuo* before being dissolved in EtOAc (50 mL). This was washed with water (50 mL) and 5% aqueous LiCl solution (2 × 100 mL) before being dried using a

hydrophobic frit and concentrated *in vacuo*. The crude solid was dissolved in DCM (20 mL) and filtered. The filtrate was concentrated *in vacuo* and the resulting solid purified by flash column chromatography. Elution with 0-0.5% MeOH/DCM afforded **3k** as an off-white solid (1.31 g, 5.52 mmol, 81%).

3k is a known compound and the NMR data are consistent with reported literature.[6]

2-Phenyl-5-(*o*-tolyl)-1,3,4-oxadiazole (3l)

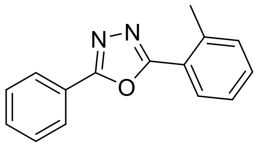

Following the General Flow Procedure using 5-phenyl-1*H*-tetrazole (50 mg, 0.342 mmol), 2-methylbenzoic acid (51 mg, 0.376 mmol), DIC (59 μL, 0.376 mmol) and 9:1 DCM/DMF (8.4 mL). Elution with 0-0.5% MeOH/DCM afforded **3l** as a white solid (71 mg, 0.300 mmol, 88%). ^{1}H NMR (500 MHz, $CDCl_3$) δ = 8.17 - 8.13 (m, 2H), 8.06 - 8.04 (m, 1H), 7.58 - 7.52 (m, 3H), 7.46 - 7.42 (m, 1H), 7.39 - 7.34 (m, 2H), 2.78 (s, 3H); ^{13}C NMR (101 MHz, $CDCl_3$) δ = 164.8, 164.1, 138.4, 131.8, 131.6, 131.2, 129.1, 128.9, 126.9, 126.1, 124.0, 123.0, 22.1; LCMS (High pH) t_R = 1.26 mins, [M+H$^+$] 237.0 (purity 100%).

3l is a known compound and the NMR data are consistent with reported literature.[6]

2-(2-Methoxyphenyl)-5-phenyl-1,3,4-oxadiazole (3m)

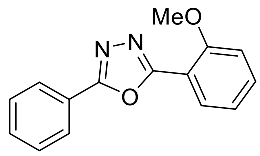

Following the General Flow Procedure using 5-phenyl-1*H*-tetrazole (50mg, 0.342 mmol), 2-methoxybenzoic acid (57 mg, 0.376 mmol), DIC (59 μL, 0.376 mmol) and 9:1 DCM/DMF (8.4 mL). Elution with 0-0.5% MeOH/DCM afforded a white solid which was impure. A second purification using 20% EtOAc/40-60 °C petroleum ether afforded **3m** as a white solid (50 mg, 0.198 mmol, 58%). ^{1}H NMR (400 MHz, $CDCl_3$) δ = 8.18 - 8.11 (m, 2H), 8.03 (dd, *J* = 1.5, 7.5 Hz, 1H), 7.57 - 7.49 (m, 4H), 7.14 - 7.06 (m, 2H), 4.00 (s, 3H); ^{13}C NMR (101

MHz, $CDCl_3$) δ = 164.3, 163.3, 157.9, 133.0, 131.5, 130.5, 129.0, 126.9, 124.2, 120.7, 113.1, 112.0, 56.0; LCMS (High pH) t_R = 1.10 mins, [M+H$^+$] 253.0 (purity 100%).

3m is a known compound and the NMR data are consistent with reported literature.[5]

2-(4-Bromophenyl)-5-phenyl-1,3,4-oxadiazole (3n)

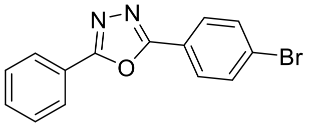

Following the General Flow Procedure using 5-phenyl-1*H*-tetrazole (50 mg, 0.342 mmol), 4-bromobenzoic acid (76 mg, 0.376 mmol), DIC (59 μL, 0.376 mmol) and 9:1 DCM/DMF (8.4 mL). Elution with 0-0.5% MeOH/DCM afforded **3n** as a white solid (52 mg, 0.173 mmol, 50%). ^{1}H NMR (400 MHz, $CDCl_3$) δ = 8.13 (dd, *J* = 1.8, 7.8 Hz, 2H), 8.04 - 7.98 (m, 2H), 7.71 - 7.65 (m, 2H), 7.59 - 7.49 (m, 3H); ^{13}C NMR (101 MHz, $CDCl_3$) δ = 164.7, 163.8, 132.4, 131.8, 129.1, 128.3, 126.9, 126.4, 123.7, 122.8; LCMS (High pH) t_R = 1.31 mins, [M+H$^+$] 300.9 and 302.9 (purity 98%).

3n is a known compound and the NMR data are consistent with reported literature.[2]

2-Butyl-5-phenyl-1,3,4-oxadiazole (3o)

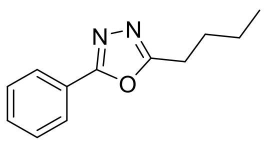

Following the general procedure using 5-phenyl-1*H*-tetrazole (50 mg, 0.342 mmol), pentanoic acid (41 μL, 0.376 mmol), DIC (59 μL, 0.376 mmol) and 9:1 DCM/DMF (8.4 mL). Elution with 0-0.5% MeOH/DCM afforded **3o** as a yellow oil (52 mg, 0.257 mmol, 75%). ^{1}H NMR (400 MHz, $CDCl_3$) δ = 8.07 - 8.00 (m, 2H), 7.57 - 7.46 (m, 3H), 2.93 (t, *J* = 8.0 Hz, 2H), 1.84 (quin., *J* = 7.5 Hz, 2H), 1.52 – 1.43 (m, 2H), 0.98 (t, *J* = 7.5 Hz, 3H); ^{13}C NMR (101 MHz, $CDCl_3$) δ = 167.0, 164.7, 131.4, 129.0, 126.7, 124.1, 28.6, 25.1, 22.1, 13.6; LCMS (High pH) t_R = 1.13 mins, [M+H$^+$] 203.0 (purity 98%).

3o is a known compound and the NMR data are consistent with reported literature.[6]

***tert*-Butyl ((5-phenyl-1,3,4-oxadiazol-2-yl)methyl)carbamate (3p)**

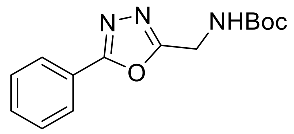

Following the General Flow Procedure using 5-phenyl-1*H*-tetrazole (50 mg, 0.342 mmol), (*tert*-butoxycarbonyl)glycine (66 mg, 0.376 mmol), DIC (59 µL, 0.376 mmol) and 9:1 DCM/DMF (8.4 mL). Elution with 10% EtOAc/DCM afforded **3p** as a colourless oil which solidified at RT (83 mg, 0.301 mmol, 88%). ^{1}H NMR (400 MHz, $CDCl_3$) δ = 8.05 - 8.00 (m, 2H), 7.56 - 7.45 (m, 3H), 5.37 (br s, 1H), 4.63 (br d, *J* = 5.5 Hz, 2H), 1.47 (s, 9H); ^{13}C NMR (101 MHz, $CDCl_3$) δ = 165.2, 163.9, 155.4, 131.8, 129.0, 126.9, 123.6, 80.5, 35.9, 28.2; LCMS (High pH) t_R = 0.98 mins, [M+H$^+$] 276.1 (purity 100%); HRMS (High pH) t_R = 7.38 mins, [M+2H]$^+$-*t*Bu calculated for $C_{10}H_{10}N_3O_3$ 220.0717, found 220.0719; IR (neat) ν_{max} = 3352, 2972, 1710, 1510, 1248, 1160 cm^{-1}.

***tert*-Butyl ((1*S*,2*S*)-2-methyl-1-(5-phenyl-1,3,4-oxadiazol-2-yl)butyl)carbamate (3q)**

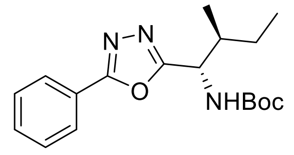

Following the General Flow Procedure using 5-phenyl-1*H*-tetrazole (50 mg, 0.342 mmol), (*tert*-butoxycarbonyl)-*L*-isoleucine (87 mg, 0.376 mmol), DIC (59 µL, 0.376 mmol) and 9:1 DCM/DMF (8.4 mL). Elution with 2.5% EtOAc/DCM afforded **3q** as a pale yellow oil (81 mg, 0.244 mmol, 71%). ^{1}H NMR (400 MHz, DMSO-*d₆*) δ = 8.01 - 7.94 (m, 2H), 7.71 (br d, *J* = 8.3 Hz, 1H), 7.66 - 7.57 (m, 3H), 4.75 (br t, *J* = 7.9 Hz, 1H), 2.04 - 1.92 (m, 1H), 1.59 - 1.46 (m, 1H), 1.38 (s, 8H), 1.30 - 1.21 (m, 2H), 0.88 (t, *J* = 7.4 Hz, 3H), 0.83 (d, *J* = 6.8 Hz, 3H); ^{13}C NMR (101 MHz, DMSO-d_6) δ = 166.3, 163.9, 155.3, 132.0, 129.4, 126.4, 123.3, 78.6, 51.4, 37.0, 28.1, 24.9, 15.2, 10.8; LCMS (High pH) t_R = 1.27 mins, [M+H$^+$] 332.1 (purity 100%); HRMS (High pH) t_R = 10.24 mins, [M+2H]$^+$-^{t}Bu calculated for $C_{14}H_{18}N_3O_3$ - 276.1343, found 276.1342; IR (neat) ν_{max} = 3242, 2969, 1706, 1250, 1163 cm^{-1}.

(9*H*-Fluoren-9-yl)methyl (*S*)-(2-(5-phenyl-1,3,4-oxadiazol-2-yl)hept-6-en-2-yl)carbamate (3r)

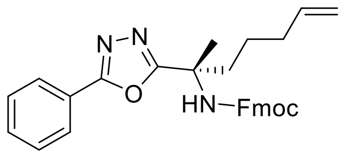

Following the General Flow Procedure using 5-phenyl-1*H*-tetrazole (55 mg, 0.375 mmol), (*S*)-2-((((9*H*-fluoren-9-yl)methoxy)carbonyl)amino)-2-methylhept-6-enoic acid (157 mg, 0.413 mmol), DIC (64 µL, 0.413 mmol) and 9:1 DCM/DMF (9.2 mL). Elution with 0-5% EtOAc/DCM afforded **3r** as a yellow oil (72 mg, 0.150 mmol, 40%). ^{1}H NMR (400 MHz, DMSO-d_6) δ = 8.17 - 8.00 (br s, 1H), 7.94 - 7.82 (m, 4H), 7.69 (br d, *J* = 6.1 Hz, 2H), 7.64 - 7.51 (m, 3H), 7.44 - 7.24 (m, 4H), 5.76 (tdd, *J* = 6.6, 10.3, 17.1 Hz, 1H), 5.03 - 4.90 (m, 2H), 4.35 - 4.13 (m, 3H), 2.14 - 1.88 (m, 4H), 1.64 (br s, 3H), 1.47 - 1.27 (m, 2H); ^{13}C NMR (101 MHz, DMSO-d_6) δ = 169.6, 163.5, 154.8, 143.8-143.6 (m, 1C), 140.7, 138.3, 131.9, 129.4, 127.6, 127.0, 126.3, 125.1, 123.4, 120.1, 115.0, 65.3, 53.5, 46.6, 37.6, 33.0, 23.5, 22.1; LCMS (High pH) t_R = 1.47 mins, [M+H$^+$] 480.2 (purity 100%); HRMS (formic acid) t_R = 6.96 mins, [M+H$^+$] calculated for $C_{30}H_{30}N_3O_3$ 480.2282, found 480.2291; IR (neat) ν_{max} = 3317, 2941, 1448, 1247 cm^{-1}.

1,3-Bis(5-phenyl-1,3,4-oxadiazol-2-yl)benzene (3s)

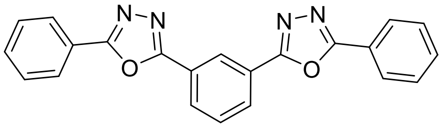

5-phenyl-1*H*-tetrazole (50 mg, 0.342 mmol) and isophthalic acid (26 mg, 0.155 mmol) were dissolved in DCM/DMF (8:2, 8.4 mL). DIC (53 µL, 0.342 mmol) was added and the reaction mixture irradiated in flow for 2 h. The reaction mixture was then collected and concentrated in vacuo before being dissolved in EtOAc (20 mL). This was washed with water (10 mL) and 5% aqueous LiCl solution (2 × 20 mL). DCM (10 mL) was added to the collected organics to dissolve the precipitate before this was dried using a hydrophobic frit and concentrated in vacuo. The crude product was purified by flash column chromatography (0-0.5% MeOH/DCM) to afford **3s** as an off-white solid (27 mg, 0.074 mmol, 48%). ^{1}H NMR (400 MHz, $CDCl_3$) δ = 8.88 (t, *J* = 1.5 Hz, 1H), 8.35 (dd, *J* = 1.8, 7.8 Hz, 2H), 8.23 - 8.17 (m, 4H), 7.74 (t, *J* = 7.8 Hz, 1H), 7.63-7.54 (m, 6H); ^{13}C NMR (101 MHz, $CDCl_3$) δ = 165.1, 163.6,

132.0, 130.0, 129.8, 129.2, 127.1, 125.1, 125.0, 123.6; LCMS (High pH) t_R = 1.32 mins, [M+H$^+$] 367.0 (purity 99%).

3s is a known compound and the NMR data are consistent with reported literature.[7]

2-Methyl-5-phenyl-1,3,4-oxadiazole (3t) – *Conditions adapted from Kappe and Reichart*

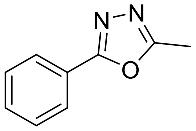

5-Phenyl-1*H*-tetrazole (100 mg, 0.684 mmol) and acetic anhydride (130 µL, 1.37 mmol) were dissolved in DME (8.6 mL). The reaction mixture was pumped through the flow system for 1 h. Water (2 mL) was added and this stirred for 1 h before being concentrated to dryness. The resulting solid was washed with 2M aqueous NaOH and filtered. The collected solid was washed with 2M aqueous NaOH and water several times before being left over vacuum to give an off-white solid (86 mg, 0.537 mmol, 78%). ^{1}H NMR (400 MHz, $CDCl_3$) δ = 8.04 - 8.00 (m, 2H), 7.54 - 7.46 (m, 3H), 2.61 (s, 3H); ^{13}C NMR (101 MHz, $CDCl_3$) δ = 164.8, 163.6, 131.5, 129.0, 126.7, 123.9, 11.0; LCMS (High pH) t_R = 0.79 mins, [M+H$^+$] 161.0 (purity 100%).

3t is a known compound and the NMR data are consistent with reported literature.[8]

2,5-Bis(4-methoxyphenyl)-1,3,4-oxadiazole (5a)

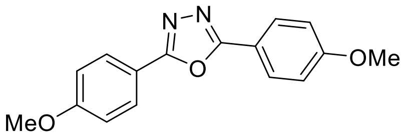

Following the General Flow Procedure using 5-(4-methoxyphenyl)-1*H*-tetrazole (26 mg, 0.148 mmol), 4-methoxybenzoic acid (25 mg, 0.162 mmol), DIC (25 µL, 0.162 mmol) and 9:1 DCM/DMF (3.6 mL). Elution with 0-0.5% MeOH/DCM afforded **5a** as a white solid (19 mg, 0.067 mmol, 59%). ^{1}H NMR (400 MHz, $CDCl_3$) δ = 8.08 - 8.02 (m, 4H), 7.05 - 7.00 (m, 4H), 3.89 (s, 6H); ^{13}C NMR (101 MHz, $CDCl_3$) δ = 164.1, 162.2, 128.5, 116.6, 114.4, 55.4; LCMS (High pH) t_R = 1.17 mins, [M+H$^+$] 283.0 (purity 97%).

5a is a known compound and the NMR data is consistent with reported literature.[2]

2-(4-Methoxyphenyl)-5-(*p*-tolyl)-1,3,4-oxadiazole (5b)

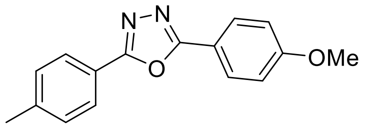

Following the General Flow Procedure using 5-(*p*-tolyl)-1*H*-tetrazole (31 mg, 0.194 mmol), 4-methoxybenzoic acid (32 mg, 0.213 mmol), DIC (33 µL, 0.213 mmol) and 9:1 DCM/DMF (4.7 mL). Elution with 0-0.5% MeOH/DCM afforded **5b** as a white solid (37 mg, 0.139 mmol, 72%). ^{1}H NMR (400 MHz, $CDCl_3$) δ = 8.09 - 8.04 (m, 2H), 8.00 (d, *J* = 8.5 Hz, 2H), 7.32 (d, *J* = 8.0 Hz, 2H), 7.05 - 6.99 (m, 2H), 3.88 (s, 3H), 2.43 (s, 3H); ^{13}C NMR (101 MHz, $CDCl_3$) δ = 164.2, 162.2, 142.0, 129.7, 128.6, 126.7, 121.3, 116.5, 114.4, 55.4, 21.6 1C not observed; LCMS (High pH) t_R = 1.25 mins, [M+H^{+}] 267.0 (purity 100%).

5b is a known compound and the NMR data are consistent with reported literature.[3]

2-(4-Methoxyphenyl)-5-(pyridin-2-yl)-1,3,4-oxadiazole (5c)

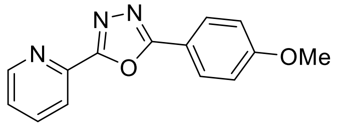

Following the General Flow Procedure using 2-(1*H*-tetrazol-5-yl)pyridine (50 mg, 0.340 mmol), 4-methoxybenzoic acid (57 mg, 0.376 mmol), DIC (59 µL, 0.376 mmol) and 9:1 DCM/DMF (8.4 mL). Elution with 2% MeOH/DCM gave a white solid which was impure. This was recrystallized from hot methanol to afford **5c** as a white solid (41 mg, 0.162 mmol, 47%). MPt: 154.6-157.3 °C; ^{1}H NMR (500 MHz, $CDCl_3$) δ = 8.79 (br d, *J* = 4.6 Hz, 1H), 8.28 (d, *J* = 7.9 Hz, 1H), 8.16 - 8.11 (m, 2H), 7.88 (ddd, *J* = 1.5, 7.8, 7.8 Hz, 1H), 7.45 (ddd, *J* = 1.1, 4.8, 7.6 Hz, 1H), 7.03 - 6.99 (m, 2H), 3.87 (s, 3H); ^{13}C NMR (101 MHz, $CDCl_3$) δ = 165.5, 163.3, 162.5, 150.1, 143.7, 137.2, 129.1, 125.6, 123.1, 116.0, 114.4, 55.4; LCMS (High pH) t_R = 0.92 mins, [M+H^{+}] 254.0 (purity 100%).

5c is a known compound and the NMR data are consistent with reported literature.[9]

2-Cyclopropyl-5-(4-methoxyphenyl)-1,3,4-oxadiazole (5d)

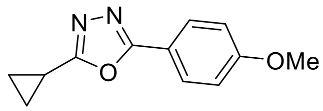

Following the General Flow Procedure using 5-cyclopropyl-1*H*-tetrazole (38 mg, 0.342 mmol), 4-methoxybenzoic acid (57 mg, 0.376 mmol), DIC (59 µL, 0.376 mmol) and 9:1 DCM/DMF (8.4 mL). Reverse phase chromatography was undertaken using a Combiflash EZ Prep with a XSelect® CSH™ Prep C18 5 µm OBD™ column and elution with 20-85% MeCN/H_2O + 0.1% ammonium bicarbonate. The fractions containing product were combined and the acetonitrile removed *in vacuo*. The remaining water was washed with DCM (3 × 20 mL) before being neutralized with ammonium chloride. The aqueous was washed with further DCM (3 × 10 mL). The organics were combined, dried using a hydrophobic frit and concentrated *in vacuo* to give **5d** as a colorless oil (28 mg, 0.129 mmol, 38%). ^{1}H NMR (400 MHz, $CDCl_3$) δ = 7.97 - 7.88 (m, 2H), 7.02 - 6.94 (m, 2H), 3.82 (s, 3H), 2.24 - 2.16 (m, 1H), 1.23 - 1.12 (m, 4H); ^{13}C NMR (101 MHz, $CDCl_3$) δ = 167.8, 163.8, 162.0, 128.3, 116.7, 114.4, 55.4, 8.2, 6.4; LCMS (High pH) t_R = 0.95 mins, [M+H$^+$] 217.1 (purity 100%); HRMS (High pH) t_R = 6.88 mins, [M+H$^+$] calculated for $C_{12}H_{13}N_2O_2$ 217.0972, found 217.0973; IR (neat) ν_{max} = 1614, 1575, 1499, 1252 cm^{-1}.

2-(4-Fluorophenyl)-5-(4-methoxyphenyl)-1,3,4-oxadiazole (5e)

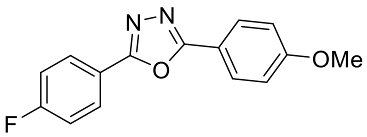

Following the General Flow Procedure using 5-(4-fluorophenyl)-1*H*-tetrazole (31 mg, 0.189 mmol), 4-methoxybenzoic acid (32 mg, 0.208 mmol), DIC (32 µL, 0.208 mmol) and 9:1 DCM/DMF (4.6 mL). Elution with 0-0.5% MeOH/DCM afforded **5e** as a white solid (42 mg, 0.155 mmol, 82%). ^{1}H NMR (500 MHz, $CDCl_3$) δ = 8.16 - 8.11 (m, 2H), 8.09 - 8.05 (m, 2H), 7.25 - 7.20 (m, 2H), 7.06 - 7.01 (m, 2H), 3.90 (s, 3H); ^{19}F NMR (376 MHz, $CDCl_3$) δ = -107.15 - -107.24 (m); ^{13}C NMR (101 MHz, $CDCl_3$) δ = 164.6, 164.7 (d, $^1J_{CF}$ = 253.5 Hz), 163.3, 162.4, 129.1 (d, $^3J_{CF}$ = 8.5 Hz), 128.7, 120.4 (d, $^4J_{CF}$ = 3.9 Hz), 116.3, 116.4 (d, $^2J_{CF}$ = 22.3 Hz), 114.5, 55.5; LCMS (High pH) t_R = 1.19 mins. [M+H$^+$] 271.0 (purity 97%).

5e is a known compound and the NMR data are consistent with reported literature.[2]

2-(4-Methoxyphenyl)-5-(*m*-tolyl)-1,3,4-oxadiazole (5f)

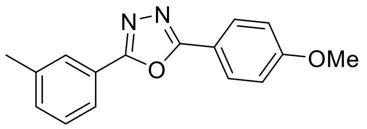

Following the General Flow Procedure using 5-(*m*-tolyl)-1*H*-tetrazole (31 mg, 0.194 mmol), 4-methoxybenzoic acid (32 mg, 0.213 mmol), DIC (33 μL, 0.213 mmol) and 9:1 DCM/DMF (4.7 mL). Elution with 0-0.5% MeOH/DCM gave an impure off-white solid. A second purification using 0.25% MeOH in DCM afforded **5f** as a white solid (33 mg, 0.124 mmol, 64%). ^{1}H NMR (500 MHz, $CDCl_3$) δ = 8.09 - 8.05 (m, 2H), 7.94 (s, 1H), 7.91 (d, *J* = 7.6 Hz, 1H), 7.40 (app. t, *J* = 7.9 Hz, 1H), 7.34 (d, *J* = 7.9 Hz, 1H), 7.04 - 7.00 (m, 2H), 3.88 (s, 3H), 2.45 (s, 3H); ^{13}C NMR (101 MHz, $CDCl_3$) δ = 164.4, 164.3, 162.3, 138.9, 132.3, 128.9, 128.7, 127.3, 123.9, 123.9, 116.5, 114.5, 55.4, 21.3; LCMS (High pH) t_R = 1.26 mins, [M+H$^+$] 267.0 (purity 100%); HRMS (High pH) t_R = 9.89 mins, [M+H$^+$] calculated for $C_{16}H_{15}N_2O_2$ 267.1128, found 267.1136; IR (neat) ν_{max} = 1614, 1497, 1254 cm^{-1}.

5f is a known compound and the NMR data are consistent with reported literature.[10]

2-(4-Bromophenyl)-5-(4-methoxyphenyl)-1,3,4-oxadiazole (5g)

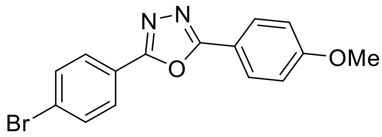

Following the General Flow Procedure 5-(4-bromophenyl)-1*H*-tetrazole (77 mg, 0.342 mmol), 4-methoxybenzoic acid (57 mg, 0.376 mmol), DIC (59 μL, 0.376 mmol) and 9:1 DCM/DMF (8.4 mL). Elution with 0-0.5% MeOH/DCM afforded **5g** as a white solid (92 mg, 0.278 mmol, 81%). ^{1}H NMR (500 MHz, $CDCl_3$) δ = 8.09 - 8.04 (m, 2H), 8.01 - 7.97 (m, 2H), 7.69 - 7.65 (m, 2H), 7.05 - 7.01 (m, 2H), 3.89 (s, 3H); ^{13}C NMR (101 MHz, $CDCl_3$) δ = 164.6, 163.3, 162.4, 132.3, 128.7, 128.1, 126.1, 122.9, 116.1, 114.5, 55.4; LCMS (High pH) t_R = 1.31 mins, [M+H$^+$] 330.9 and 332.8 (purity 98%).

5g is a known compound and the NMR data are consistent with reported literature.[2]

2-(3-Bromophenyl)-5-(4-methoxyphenyl)-1,3,4-oxadiazole (5h)

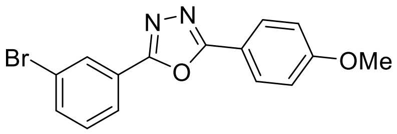

Following the general procedure using from 5-(3-bromophenyl)-1*H*-tetrazole (77mg, 0.342 mmol), 4-methoxybenzoic acid (57 mg, 0.376 mmol), DIC (59 µL, 0.376 mmol) and 9:1 DCM/DMF (8.4 mL). Following the general procedure. Elution with 0-0.5% MeOH/DCM gave **5h** as a white solid (88 mg, 0.266 mmol, 78%). ^{1}H NMR (400 MHz, $CDCl_3$) δ = 8.27 (appt. t, J = 1.8 Hz, 1H), 8.11 - 8.05 (m, 3H), 7.68 (ddd, J = 1.1, 1.9, 8.0 Hz, 1H), 7.41 (appt. t, J = 7.9 Hz, 1H), 7.07 - 7.02 (m, 2H), 3.91 (s, 3H); ^{13}C NMR (101 MHz, $CDCl_3$) δ = 164.9, 162.8, 162.5, 134.5, 130.6, 129.6, 128.8, 125.9, 125.3, 123.1, 116.1, 114.6, 55.5; LCMS (High pH) t_R = 1.31 mins, [M+H$^+$] 330.9 & 332.9 (purity 100%); HRMS (Formic acid) t_R = 5.84 mins, [M+H$^+$] calculated for $C_{15}H_{12}BrN_2O_2$ 331.0077, found 331.0080; IR (neat) ν_{max} = 1616, 1503, 1268 cm^{-1}.

2-(4-Methoxyphenyl)-5-(4-(trifluoromethyl)phenyl)-1,3,4-oxadiazole (5i)

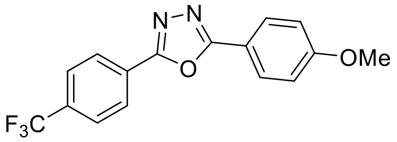

Following the General Flow Procedure 5-(4-(trifluoromethyl)phenyl)-1*H*-tetrazole (31 mg, 0.145 mmol), 4-methoxybenzoic acid (24 mg, 0.160 mmol), DIC (25 µL, 0.160 mmol) and 9:1 DCM/DMF (3.5 mL). Elution with 0-0.5% MeOH/DCM afforded **5i** as a white solid (32 mg, 0.100 mmol, 69%). ^{1}H NMR (400 MHz, $CDCl_3$) δ = 8.25 (d, J = 8.5 Hz, 2H), 8.11 - 8.06 (m, 2H), 7.80 (d, J = 8.0 Hz, 2H), 7.07 - 7.02 (m, 2H), 3.90 (s, 3H); ^{19}F NMR (376 MHz, $CDCl_3$) δ = -63.06 (s); ^{13}C NMR (101 MHz, $CDCl_3$) δ = 165.1, 163.0, 162.6, 133.1 (q, $^2J_{CF}$ = 32.9 Hz), 128.9, 127.3, 127.1, 126.1 (q, $^3J_{CF}$ = 3.9 Hz), 123.6 (q, $^1J_{CF}$ = 272.8 Hz), 116.0, 114.6, 55.5; LCMS (High pH) t_R = 1.32 mins, [M+H$^+$] 321.0 (purity 98%); HRMS (High pH) t_R = 10.66 mins, [M+H$^+$] calculated for $C_{16}H_{12}F_3N_2O_2$ 321.0845, found 321.0850; IR (neat) ν_{max} = 1611, 1495, 1324, 1118 cm^{-1}.

2-(4-Methoxyphenyl)-5-(*o*-tolyl)-1,3,4-oxadiazole (5j)

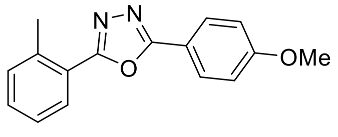

Following the General Flow Procedure using 5-(*o*-tolyl)-1*H*-tetrazole (31 mg, 0.194 mmol), 4-methoxybenzoic acid (32 mg, 0.213 mmol), DIC (33 µL, 0.213 mmol) and 9:1 DCM/DMF (4.7 mL). Elution with 0-0.5% MeOH/DCM afforded **5j** as a white solid (37 mg, 0.139 mmol, 72%). ^{1}H NMR (500 MHz, $CDCl_3$) δ = 8.10 - 8.05 (m, 2H), 8.02 (d, *J* = 7.9 Hz, 1H), 7.44 - 7.40 (m, 1H), 7.38 - 7.33 (m, 2H), 7.05 - 7.01 (m, 2H), 3.89 (s, 3H), 2.77 (s, 3H); ^{13}C NMR (101 MHz, $CDCl_3$) δ = 164.3, 164.0, 162.3, 138.3, 131.7, 131.0, 128.8, 128.6, 126.1, 123.1, 116.4, 114.5, 55.4, 22.1; LCMS (High pH) t_R = 1.26 mins, [M+H$^+$] 267.0 (purity 100%).

5j is a known compound and the NMR data are consistent with reported literature.[11]

2-(5-Bromothiophen-2-yl)-5-(4-methoxyphenyl)-1,3,4-oxadiazole (5k)

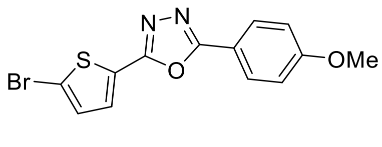

Following the General Flow Procedure using 5-(5-bromothiophen-2-yl)-1*H*-tetrazole (79 mg, 0.342 mmol), 4-methoxybenzoic acid (57 mg, 0.376 mmol), DIC (59 µL, 0.376 mmol) and 9:1 DCM/DMF (8.4 mL). Elution with 0-0.2% MeOH/DCM gave an impure off-white solid. This was further purified eluting with 0-50% TBME/cyclohexane to afford **5k** as a white solid (40 mg, 0.125 mmol, 36%). ^{1}H NMR (400 MHz, $CDCl_3$) δ = 8.06 - 7.95 (m, 2H), 7.52 (d, *J* = 3.9 Hz, 1H), 7.13 (d, *J* = 3.9 Hz, 1H), 7.04 - 6.98 (m, 2H), 3.88 (s, 3H); ^{13}C NMR (101 MHz, $CDCl_3$) δ = 164.1, 162.5, 159.3, 131.1, 129.5, 128.7, 126.8, 117.7, 116.0, 114.6, 55.5; LCMS (High pH) t_R = 1.31 mins, [M+H$^+$] 336.8 and 338.8 (purity 100%); HRMS (High pH) t_R = 10.33 mins, [M+H$^+$] calculated for $C_{13}H_{10}BrN_2O_2S$ 336.9641, found 336.9646; IR (neat) ν_{max} = 1614, 1582, 1491, 1265, 1018 cm^{-1}.

2-(2-Methoxyphenyl)-5-(4-methoxyphenyl)-1,3,4-oxadiazole (5l)

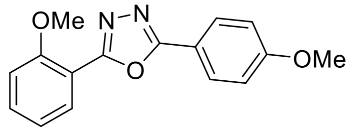

Following the General Flow Procedure using 5-(2-methoxyphenyl)-1*H*-tetrazole (38 mg, 0.342 mmol), 4-methoxybenzoic acid (57 mg, 0.376 mmol), DIC (59 µL, 0.376 mmol) and 9:1 DCM/DMF (8.4 mL). Reverse phase chromatography was undertaken using a Combiflash EZ Prep with a XSelect® CSH™ Prep C18 5 µm OBD™ column and elution with 20-85% MeCN/H_2O + 0.1% ammonium bicarbonate. The fractions containing product were combined and the acetonitrile removed *in vacuo*. The remaining water was washed with DCM (3 × 20 mL) and the organics were combined, dried using a hydrophobic frit and concentrated *in vacuo* to give **5l** as a pale yellow oil (33 mg, 0.117 mmol, 34%). ^{1}H NMR (400 MHz, $CDCl_3$) δ = 8.09 - 8.04 (m, 2H), 7.99 (dd, J = 1.7, 7.6 Hz, 1H), 7.53 - 7.47 (m, 1H), 7.11 - 7.05 (m, 2H), 7.04 - 6.99 (m, 2H), 3.98 (s, 3H), 3.87 (s, 3H); ^{13}C NMR (101 MHz, $CDCl_3$) δ = 164.3, 162.8, 162.1, 157.8, 132.8, 130.3, 128.6, 120.7, 116.7, 114.4, 113.3, 112.0, 56.0, 55.4; LCMS (High pH) t_R = 1.10 mins, [M+H$^+$] 283.0 (purity 100%); HRMS (High pH) t_R = 8.52 mins, [M+H$^+$] calculated for $C_{16}H_{15}N_2O_3$ 283.1077, found 283.1080; IR (neat) ν_{max} = 2836, 1603, 1498, 1249 cm^{-1}.

2-(3-(4-Fluorophenyl)-1-phenyl-1*H*-pyrazol-4-yl)-5-phenyl-1,3,4-oxadiazole (11)

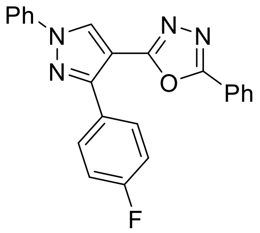

Following the General Flow Procedure using 5-phenyl-1*H*-tetrazole (50 mg, 0.342 mmol), 3-(4-fluorophenyl)-1-phenyl-1*H*-pyrazole-4-carboxylic acid (106 mg, 0.376 mmol), DIC (59 µL, 0.376 mmol) and 9:1 DCM/DMF (8.4 mL). Elution with 0-0.1% MeOH/DCM afforded **11** as a white solid (111 mg, 0.291 mmol, 85%). ^{1}H NMR (400 MHz, $CDCl_3$) δ = 8.68 (s, 1H), 8.02 - 7.96 (m, 4H), 7.86 - 7.82 (m, 2H), 7.58 - 7.48 (m, 5H), 7.43 - 7.38 (m, 1H), 7.24 - 7.17 (m, 2H); ^{19}F NMR (376 MHz, $CDCl_3$) δ = -112.31 - -112.39 (m, 1F); ^{13}C NMR (101 MHz, $CDCl_3$) δ = 164.6, 163.9, 160.9 (d, $^1J_{CF}$ = 242.0 Hz), 150.9, 139.1, 131.7, 131.0 (d, $^3J_{CF}$

= 8.5 Hz), 129.7, 129.4, 129.1, 127.9 (br d, J = 3.1 Hz), 127.7, 126.7, 123.7, 119.5, 115.3 (br d, $^2J_{CF}$ = 21.6 Hz), 106.4; LCMS (High pH) t_R = 1.46 mins, [M+H$^+$] 383.1 (purity 99%); HRMS (High pH) t_R = 12.18 mins, [M+H$^+$] calculated for $C_{23}H_{16}FN_4O$ 383.1303, found 383.1307; IR (neat) ν_{max} = 3384, 1592, 1510, 1221 cm^{-1}.

11 is a known compound and the NMR data are consistent with reported literature.[12,13]

6. References

(1) Huisgen, R.; Sauer, J.; Sturm, H. J.; Markgraf, J. H. Ringöffnungen der Azole, II. Die Bildung von 1.3.4-Oxdiazolen bei der Acylierung 5-substituierter Tetrazole, *Chem. Ber.* **1960**, *93*, 2106.

(2) Kawano, T.; Yoshizumi, T.; Hirano, K.; Satoh, T.; Miura, M. Copper-Mediated Direct Arylation of 1,3,4-Oxadiazoles and 1,2,4-Triazoles with Aryl Iodides, *Org. Lett.* **2009**, *11*, 3072.

(3) Guin, S.; Ghosh, T.; Rout, S. K.; Banerjee, A.; Patel, B. K. Cu(II) Catalyzed Imine C–H Functionalization Leading to Synthesis of 2,5-Substituted 1,3,4-Oxadiazoles, *Org. Lett.* **2011**, *13*, 5976.

(4) Dailey, S.; Feast, W. J.; Peace, R. J.; Sage, I. C.; Till, S.; Wood, E. L. Synthesis and device characterisation of side-chain polymer electron transport materials for organic semiconductor applications, *J. Mater. Chem.* **2001**, *11*, 2238.

(5) Wang, L.; Cao, J.; Chen, Q.; He, M. One-Pot Synthesis of 2,5-Diaryl 1,3,4-Oxadiazoles via Di-tert-butyl Peroxide Promoted N-Acylation of Aryl Tetrazoles with Aldehydes, *J. Org. Chem.* **2015**, *80*, 4743.

(6) Stabile, P.; Lamonica, A.; Ribecai, A.; Castoldi, D.; Guercio, G.; Curcuruto, O. Mild and convenient one-pot synthesis of 1,3,4-oxadiazoles, *Tetrahedron Lett.* **2010**, *51*, 4801.

(7) Yang, C.-C.; Hsu, C.-J.; Chou, P.-T.; Cheng, H. C.; Su, Y. O.; Leung, M.-k. Excited State Luminescence of Multi-(5-phenyl-1,3,4-oxadiazo-2-yl)benzenes in an Electron-Donating Matrix: Exciplex or Electroplex?, *J. Phys. Chem. B* **2010**, *114*, 756.

(8) Reichart, B.; Kappe, C. O. High-temperature continuous flow synthesis of 1,3,4-oxadiazoles via N-acylation of 5-substituted tetrazoles, *Tetrahedron Lett.* **2012**, *53*, 952.

(9) Pazinato, J.; Cruz, O. M.; Naidek, K. P.; Pires, A. R. A.; Westphal, E.; Gallardo, H.; Baubichon-Cortay, H.; Rocha, M. E. M.; Martinez, G. R.; Winnischofer, S. M. B.; Di Pietro, A.; Winnischofer, H. Cytotoxicity of η6-areneruthenium-based molecules to glioblastoma cells and their recognition by multidrug ABC transporters, *Eur. J. Med. Chem.* **2018**, *148*, 165.

(10) Li, Z.; Zhu, A.; Mao, X.; Sun, X.; Gong, X. Silica-supported dichlorophosphate: a recoverable cyclodehydrant for the eco-friendly synthesis of 2,5-disubstituted 1,3,4-oxadiazoles under solvent-free and microwave irradiation conditions, *J. Braz. Chem. Soc.* **2008**, *19*, 1622.

(11) Mule, S. N. R.; Battula, S. K.; Velupula, G.; Guda, D. R.; Bollikolla, H. B. 10-Camphorsulfonic acid ((±)-CSA) catalyzed facile one-pot synthesis of a new class of 2,5-disubstituted 1,3,4-oxadiazoles, *RSC Adv.* **2014**, *4*, 58397.

(12) Bansal, S.; Bala, M.; Suthar, S. K.; Choudhary, S.; Bhattacharya, S.; Bhardwaj, V.; Singla, S.; Joseph, A. Design and synthesis of novel 2-phenyl-5-(1,3-diphenyl-1H-pyrazol-4-yl)-1,3,4-oxadiazoles as selective COX-2 inhibitors with potent anti-inflammatory activity, *Eur. J. Med. Chem.* **2014**, *80*, 167.

(13) Prakash, O.; Kumar, M.; Kumar, R.; Sharma, C.; Aneja, K. R. Hypervalent iodine(III) mediated synthesis of novel unsymmetrical 2,5-disubstituted 1,3,4-oxadiazoles as antibacterial and antifungal agents, *Eur. J. Med. Chem.* **2010**, *45*, 4252.

8. Spectra

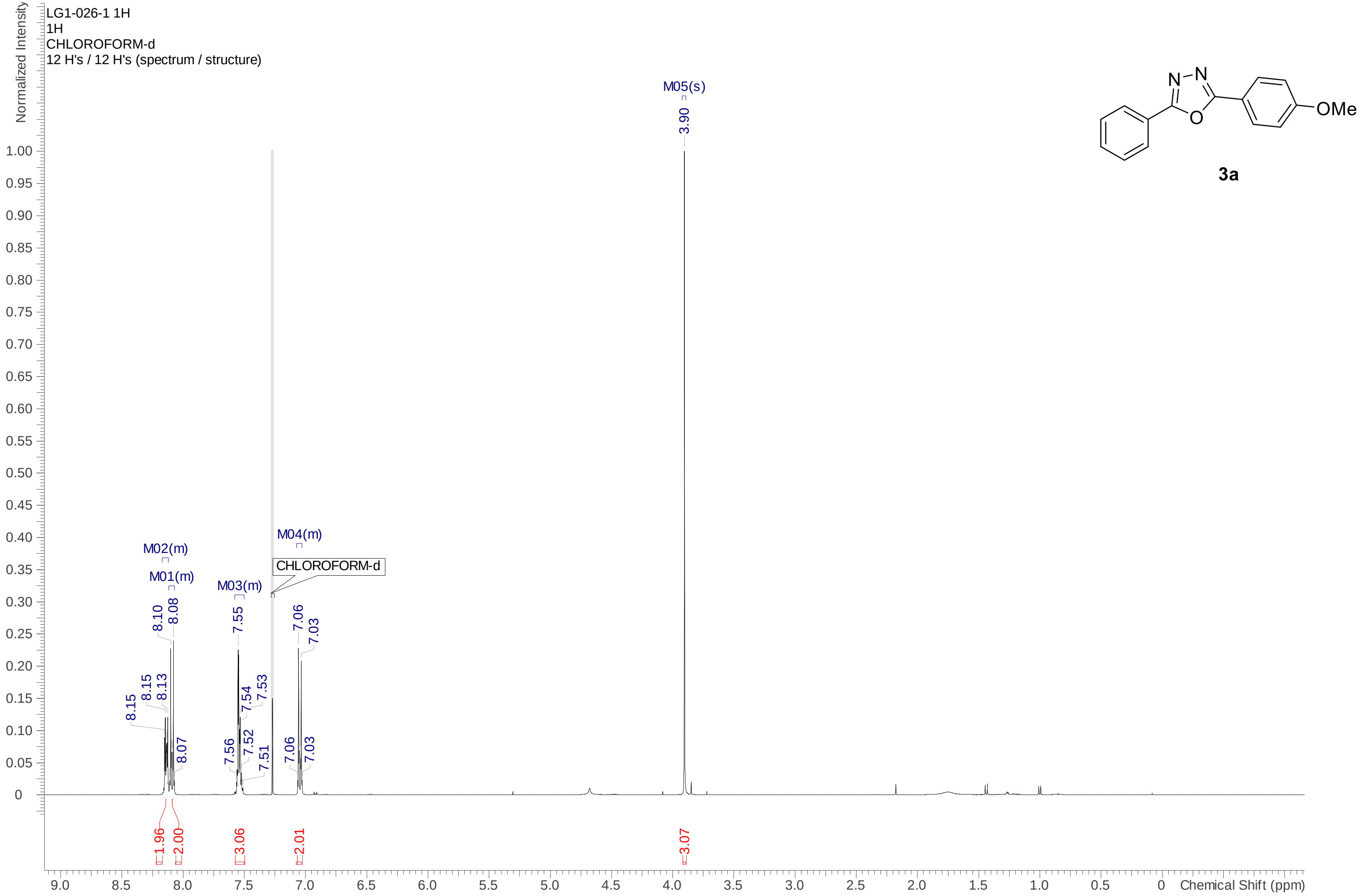
LG1-026-1 1H
1H
CHLOROFORM-d
12 H's / 12 H's (spectrum / structure)
Normalized Intensity
M01(m)
M02(m)
M03(m)
M04(m)
M05(s)
CHLOROFORM-d
3.90
1.96
2.00
3.06
2.01
3.07
Chemical Shift (ppm)
N–N
O
OMe

3a

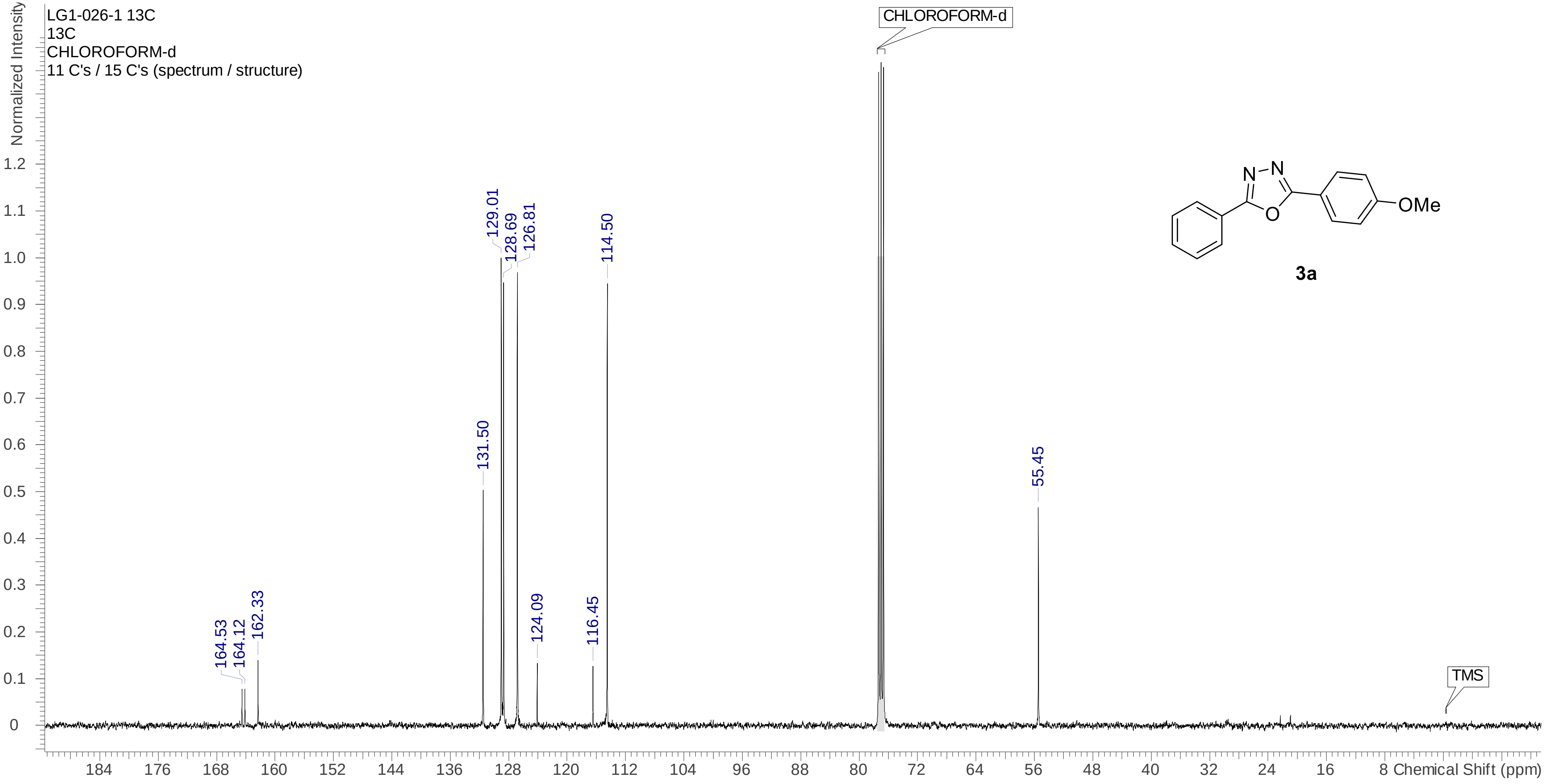
LG1-026-1 13C
13C
CHLOROFORM-d
11 C's / 15 C's (spectrum / structure)
Normalized Intensity
CHLOROFORM-d
TMS
164.53
164.12
162.33
131.50
129.01
128.69
126.81
124.09
116.45
114.50
55.45
N
N
O
OMe
3a
Chemical Shift (ppm)

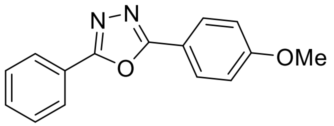
N–N
O
OMe

3a

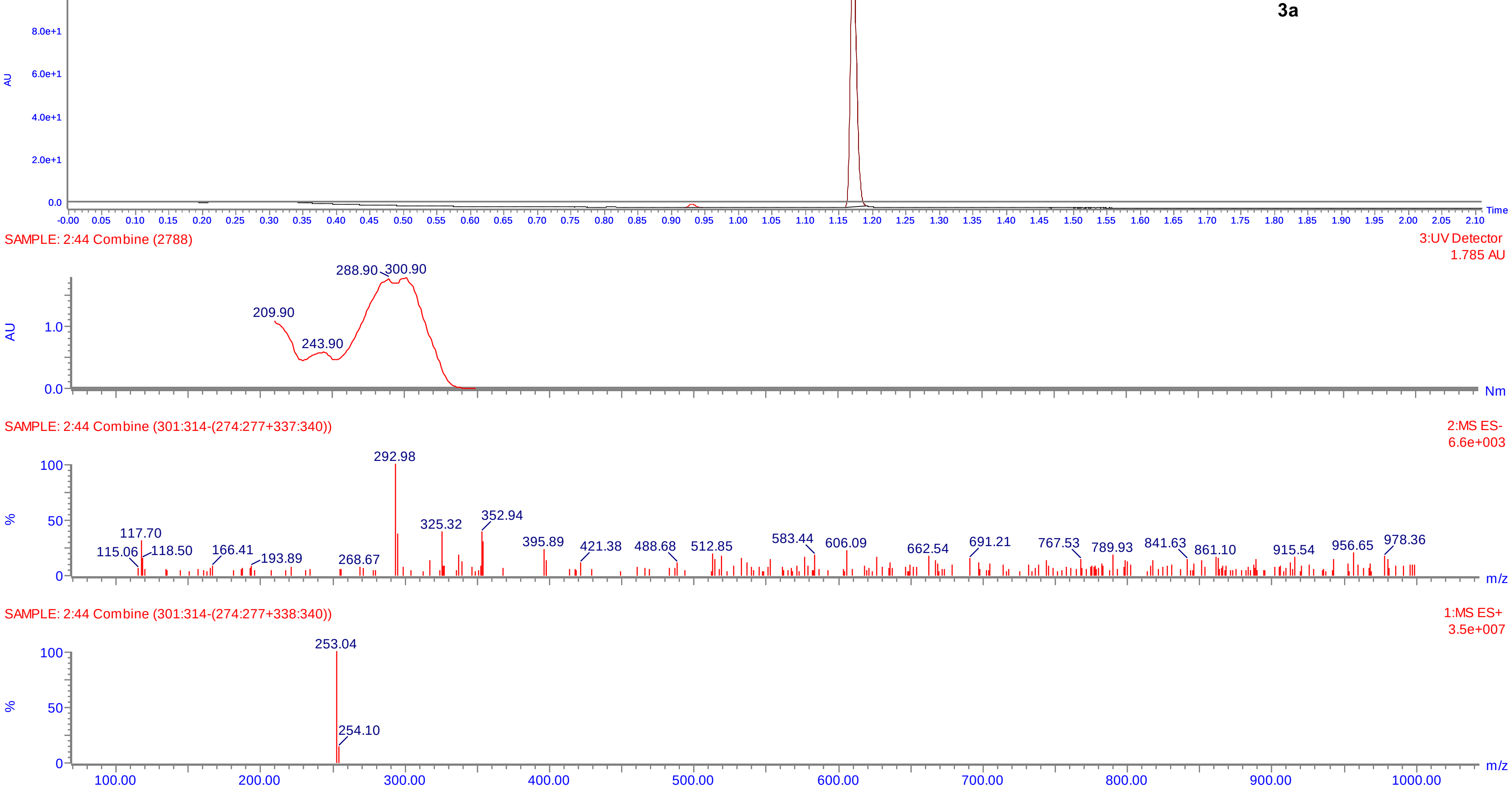
UV Detector: TIC
1.166e+2
Range: 1.196e+2
(2)
99%
1.17
AU
Time
SAMPLE: 2:44 Combine (2788)
3:UV Detector
1.785 AU
209.90
243.90
288.90
300.90
Nm
SAMPLE: 2:44 Combine (301:314-(274:277+337:340))
2:MS ES-
6.6e+003
292.98
325.32
352.94
117.70
115.06
118.50
166.41
193.89
268.67
395.89
421.38
488.68
512.85
583.44
606.09
662.54
691.21
767.53
789.93
841.63
861.10
915.54
956.65
978.36
m/z
SAMPLE: 2:44 Combine (301:314-(274:277+338:340))
1:MS ES+
3.5e+007
253.04
254.10
%
m/z

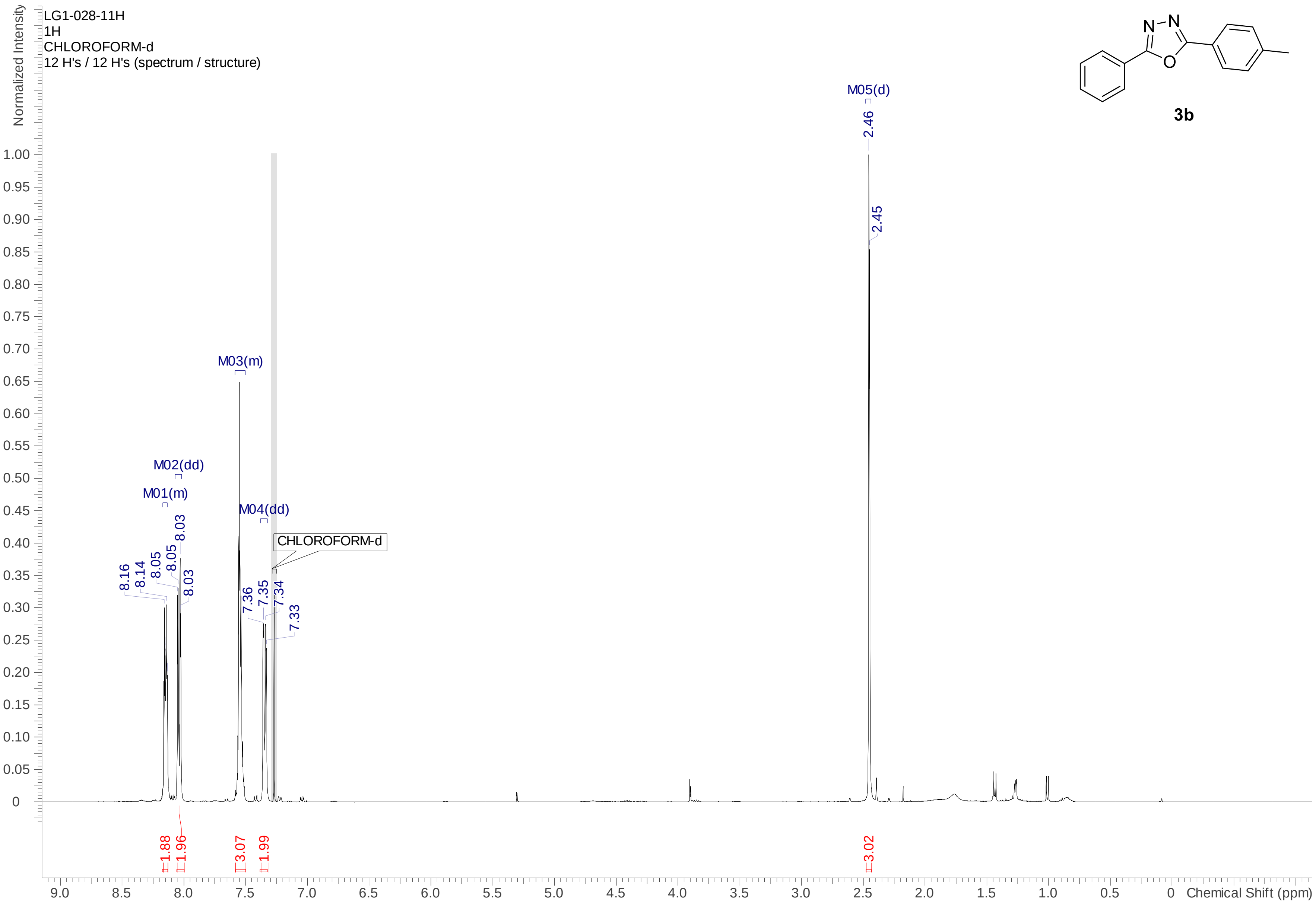
LG1-028-11H
1H
CHLOROFORM-d
12 H's / 12 H's (spectrum / structure)
3b
Normalized Intensity
M01(m)
M02(dd)
M03(m)
M04(dd)
M05(d)
CHLOROFORM-d
8.16
8.14
8.05
8.05
8.03
8.03
7.36
7.35
7.34
7.33
2.46
2.45
1.88
1.96
3.07
1.99
3.02
Chemical Shift (ppm)

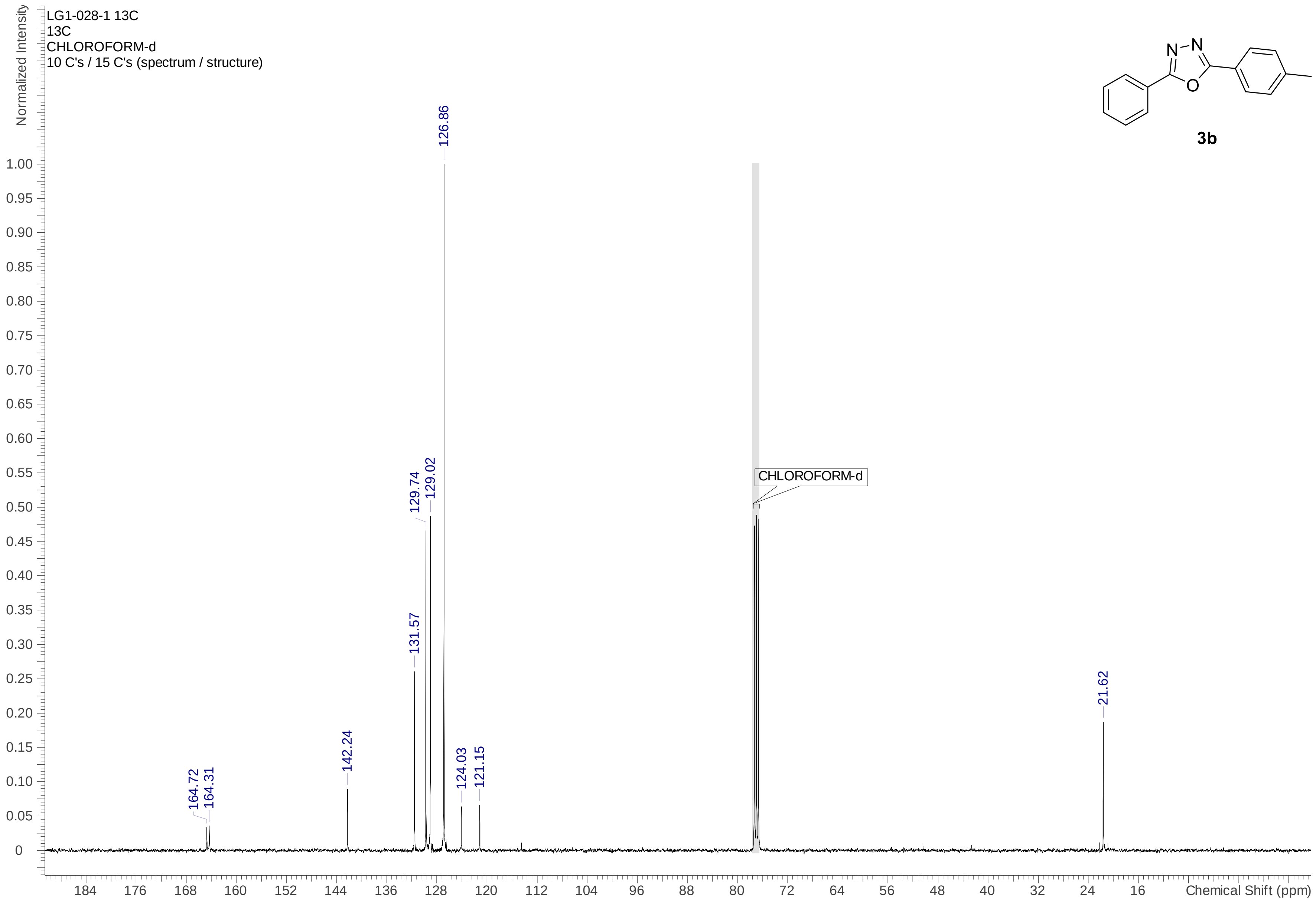

LG1-028-1 13C
13C
CHLOROFORM-d
10 C's / 15 C's (spectrum / structure)
Normalized Intensity
3b
164.72
164.31
142.24
131.57
129.74
129.02
126.86
124.03
121.15
CHLOROFORM-d
21.62
Chemical Shift (ppm)

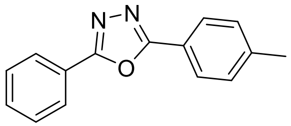
N
N
O

3b

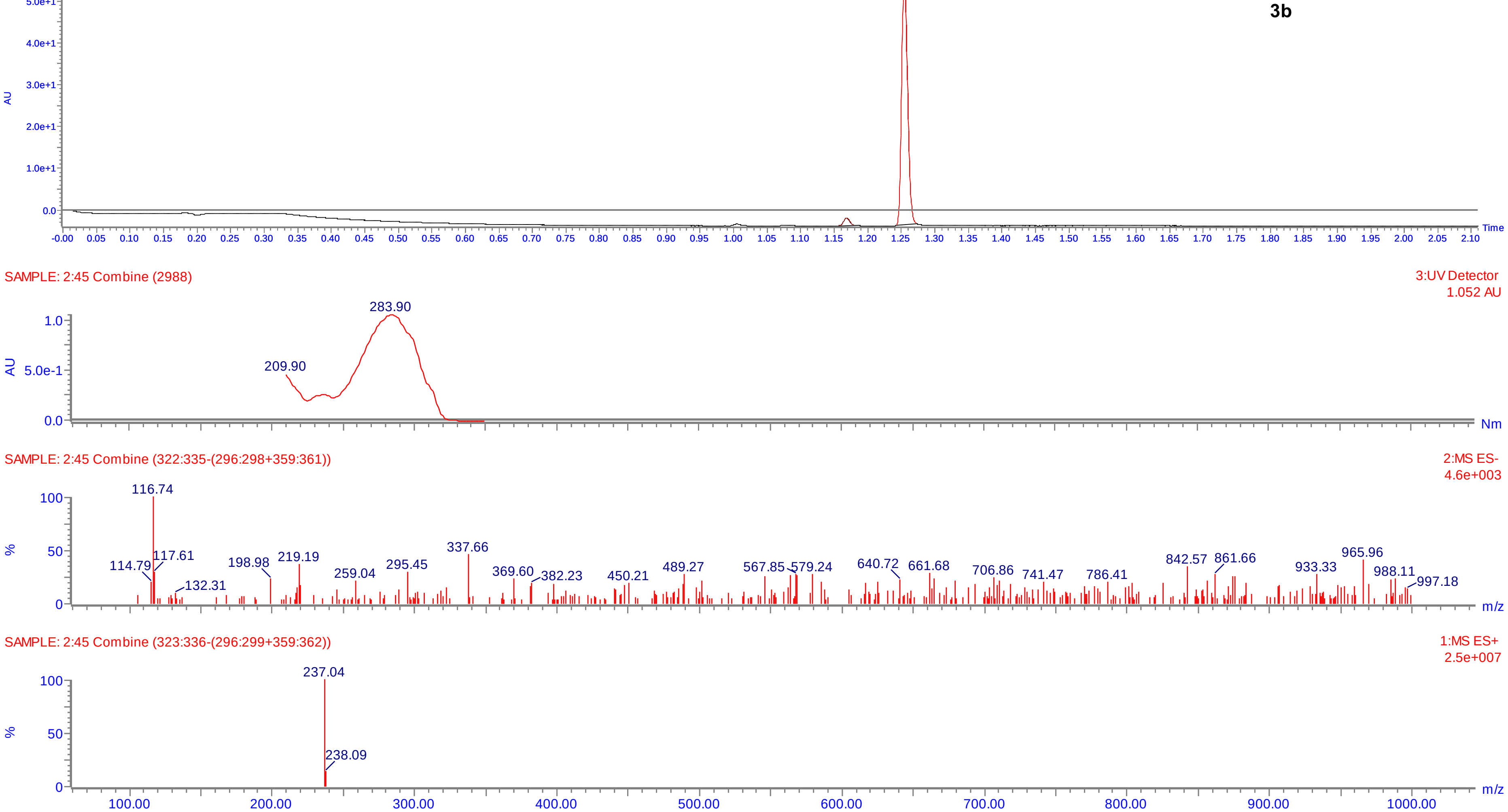
UV Detector: TIC
5.71e+1
Range: 6.095e+1
(2)
97%
1.25
AU
Time
SAMPLE: 2:45 Combine (2988)
3:UV Detector
1.052 AU
283.90
209.90
Nm
SAMPLE: 2:45 Combine (322:335-(296:298+359:361))
2:MS ES-
4.6e+003
116.74
114.79
117.61
132.31
198.98
219.19
259.04
295.45
337.66
369.60
382.23
450.21
489.27
567.85
579.24
640.72
661.68
706.86
741.47
786.41
842.57
861.66
933.33
965.96
988.11
997.18
m/z
SAMPLE: 2:45 Combine (323:336-(296:299+359:362))
1:MS ES+
2.5e+007
237.04
238.09
%
m/z

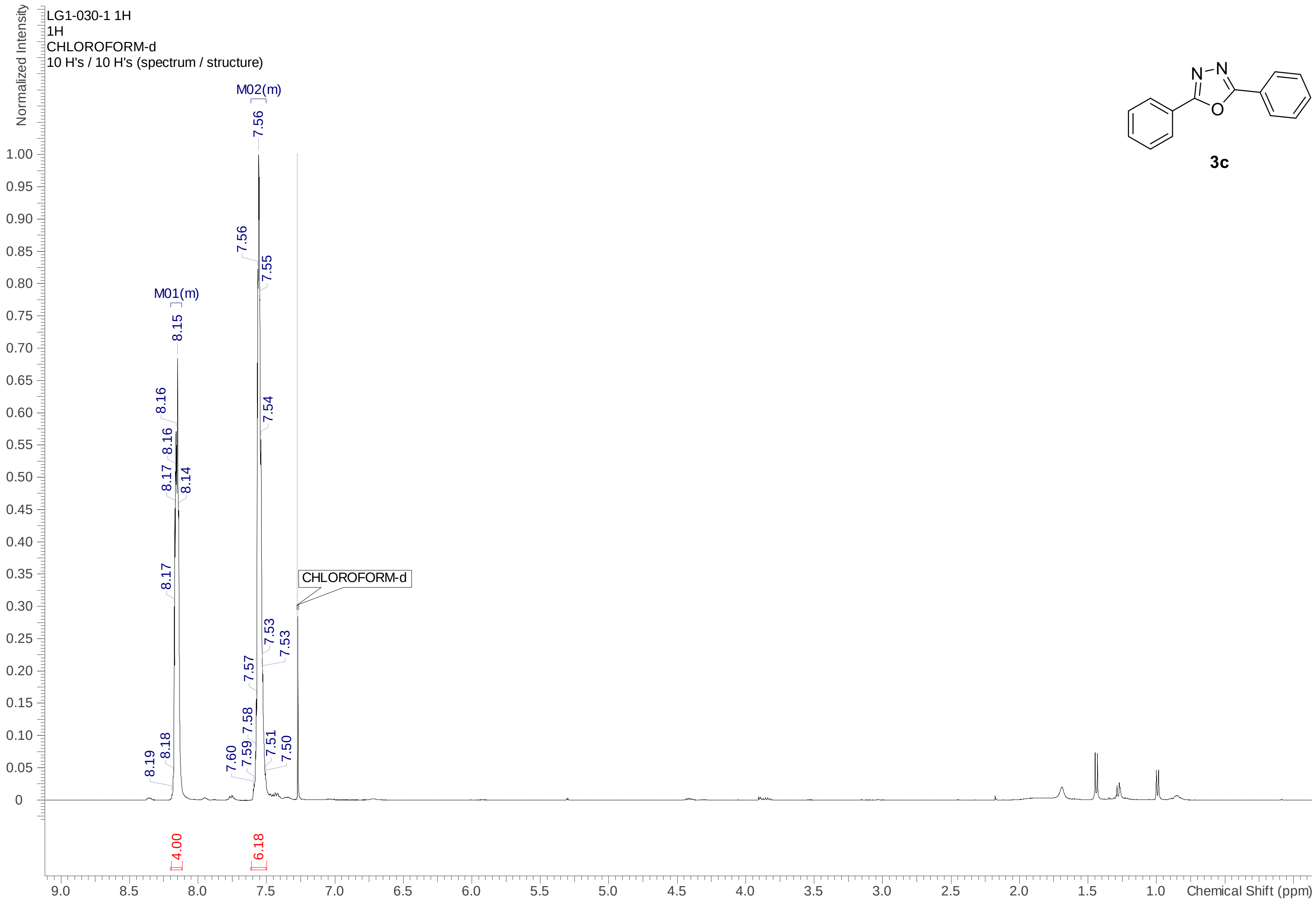
LG1-030-1 1H
1H
CHLOROFORM-d
10 H's / 10 H's (spectrum / structure)
Normalized Intensity
M01(m)
8.15
8.16
8.16
8.17
8.14
8.17
8.18
8.19
M02(m)
7.56
7.56
7.55
7.54
7.53
7.53
7.57
7.58
7.59
7.60
7.51
7.50
CHLOROFORM-d
4.00
6.18
Chemical Shift (ppm)
N
N
O

3c

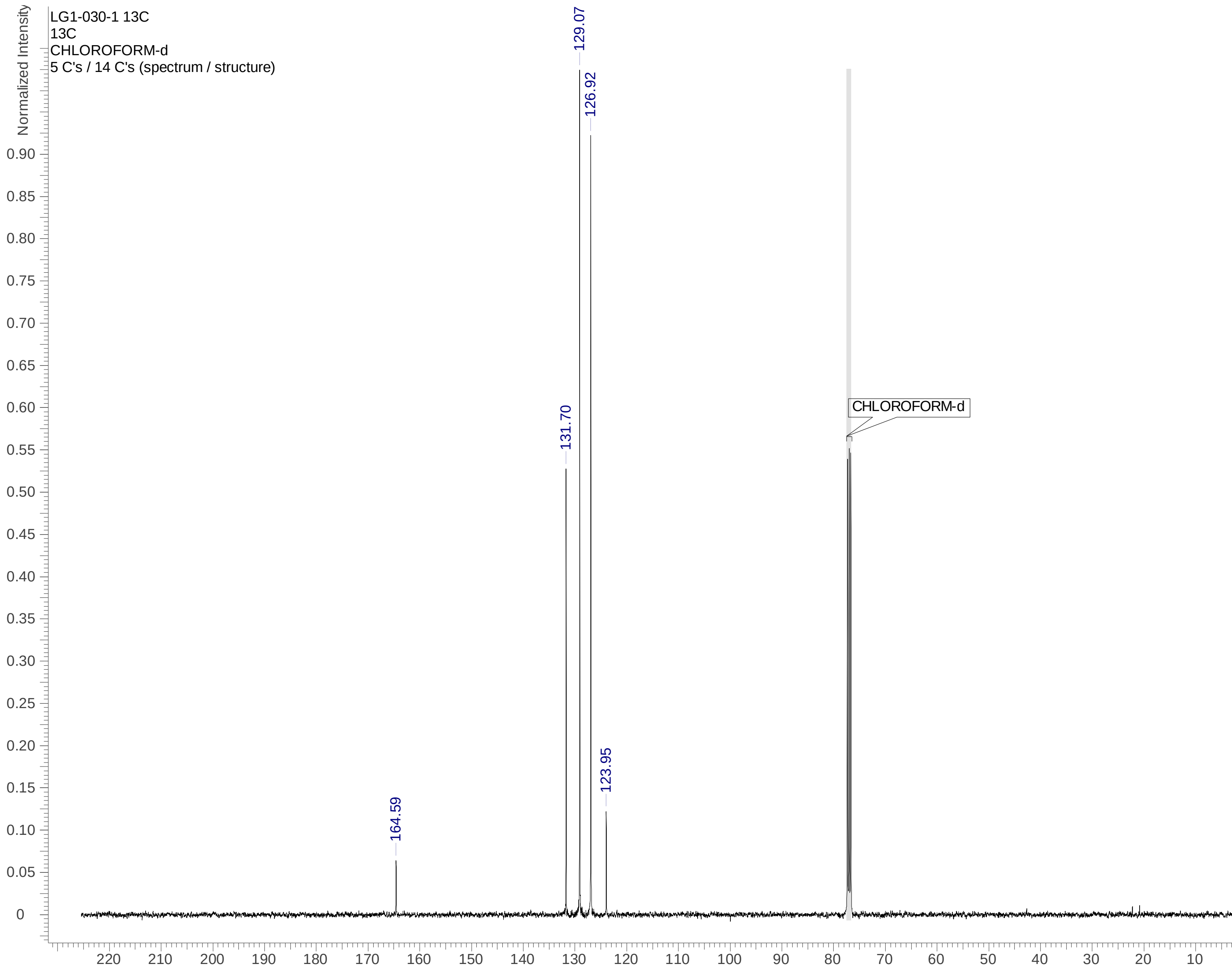
LG1-030-1 13C
13C
CHLOROFORM-d
5 C's / 14 C's (spectrum / structure)
Normalized Intensity
129.07
126.92
131.70
123.95
164.59
CHLOROFORM-d
Chemical Shift (ppm)

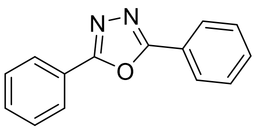
N
N
O

3c

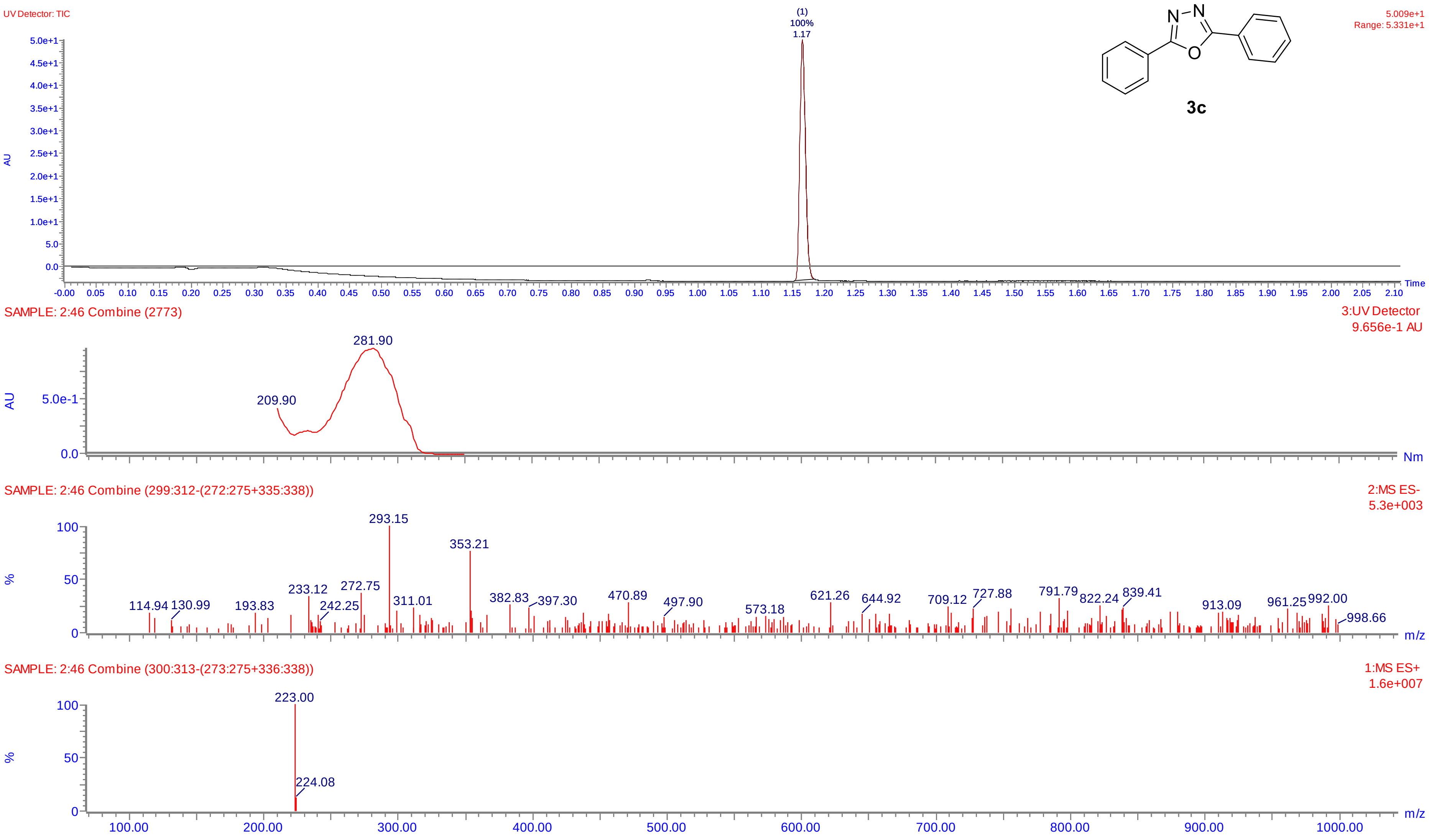

UV Detector: TIC
(1)
100%
1.17
5.009e+1
Range: 5.331e+1
3c
SAMPLE: 2:46 Combine (2773)
3:UV Detector
9.656e-1 AU
281.90
209.90
SAMPLE: 2:46 Combine (299:312-(272:275+335:338))
2:MS ES-
5.3e+003
293.15
353.21
SAMPLE: 2:46 Combine (300:313-(273:275+336:338))
1:MS ES+
1.6e+007
223.00
224.08

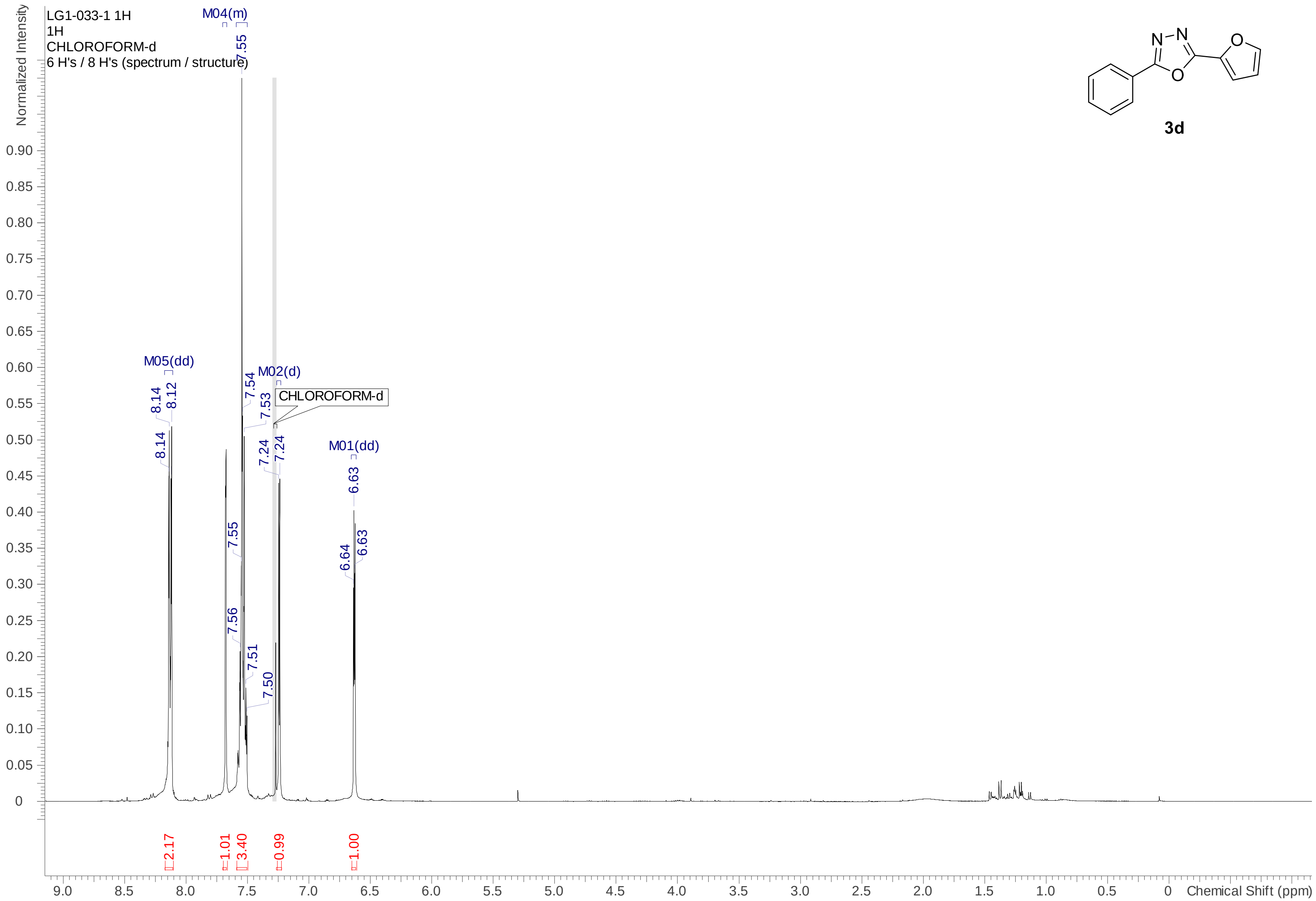
LG1-033-1 1H
1H
CHLOROFORM-d
6 H's / 8 H's (spectrum / structure)
M04(m)
7.55
M05(dd)
8.14
8.12
8.14
M02(d)
7.54
7.53
CHLOROFORM-d
7.24
7.24
M01(dd)
6.63
6.64
6.63
7.55
7.56
7.51
7.50
2.17
1.01
3.40
0.99
1.00
Normalized Intensity
Chemical Shift (ppm)
N
N
O
O

3d

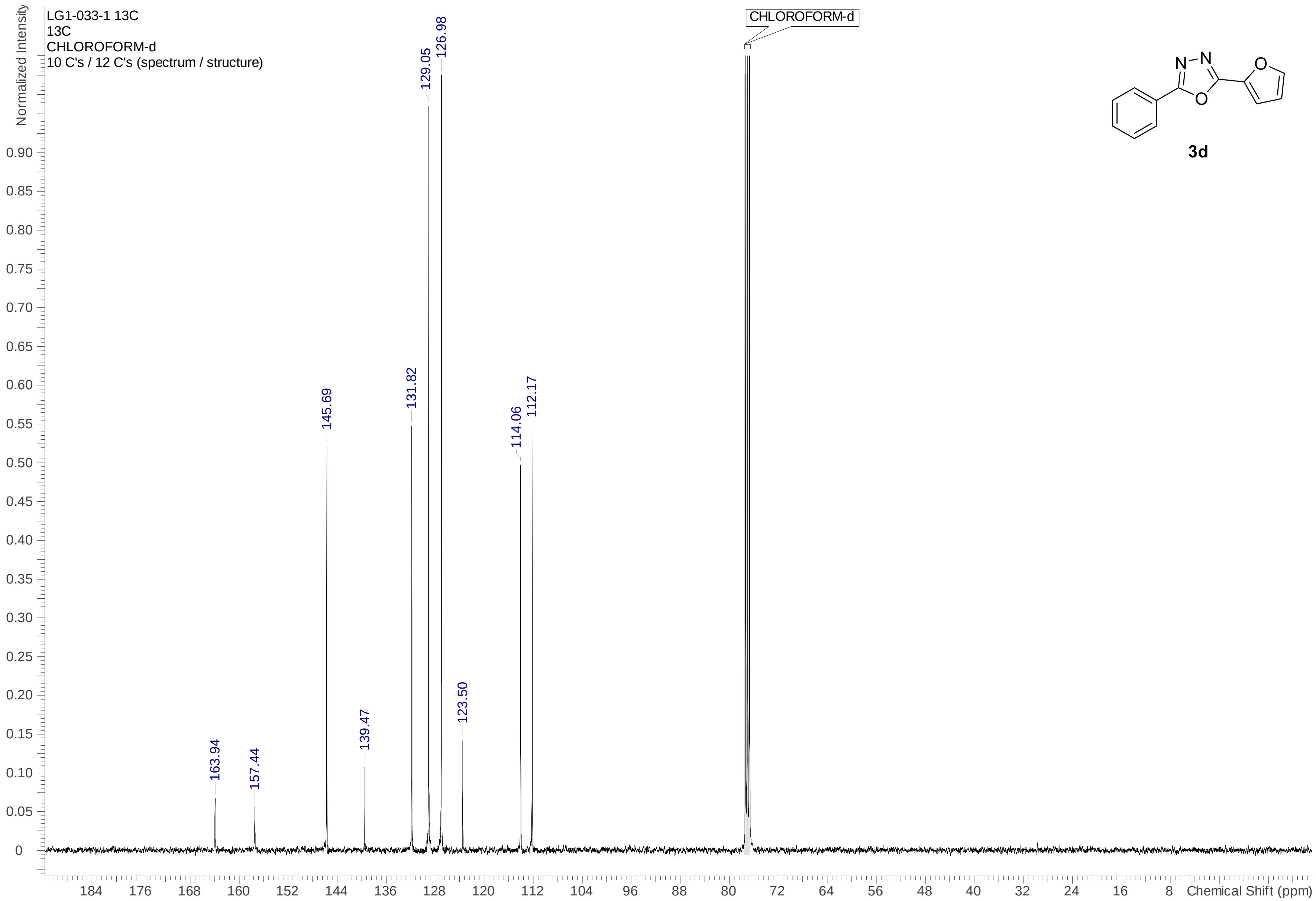
LG1-033-1 13C
13C
CHLOROFORM-d
10 C's / 12 C's (spectrum / structure)
CHLOROFORM-d
163.94
157.44
145.69
139.47
131.82
129.05
126.98
123.50
114.06
112.17
3d
Normalized Intensity
Chemical Shift (ppm)

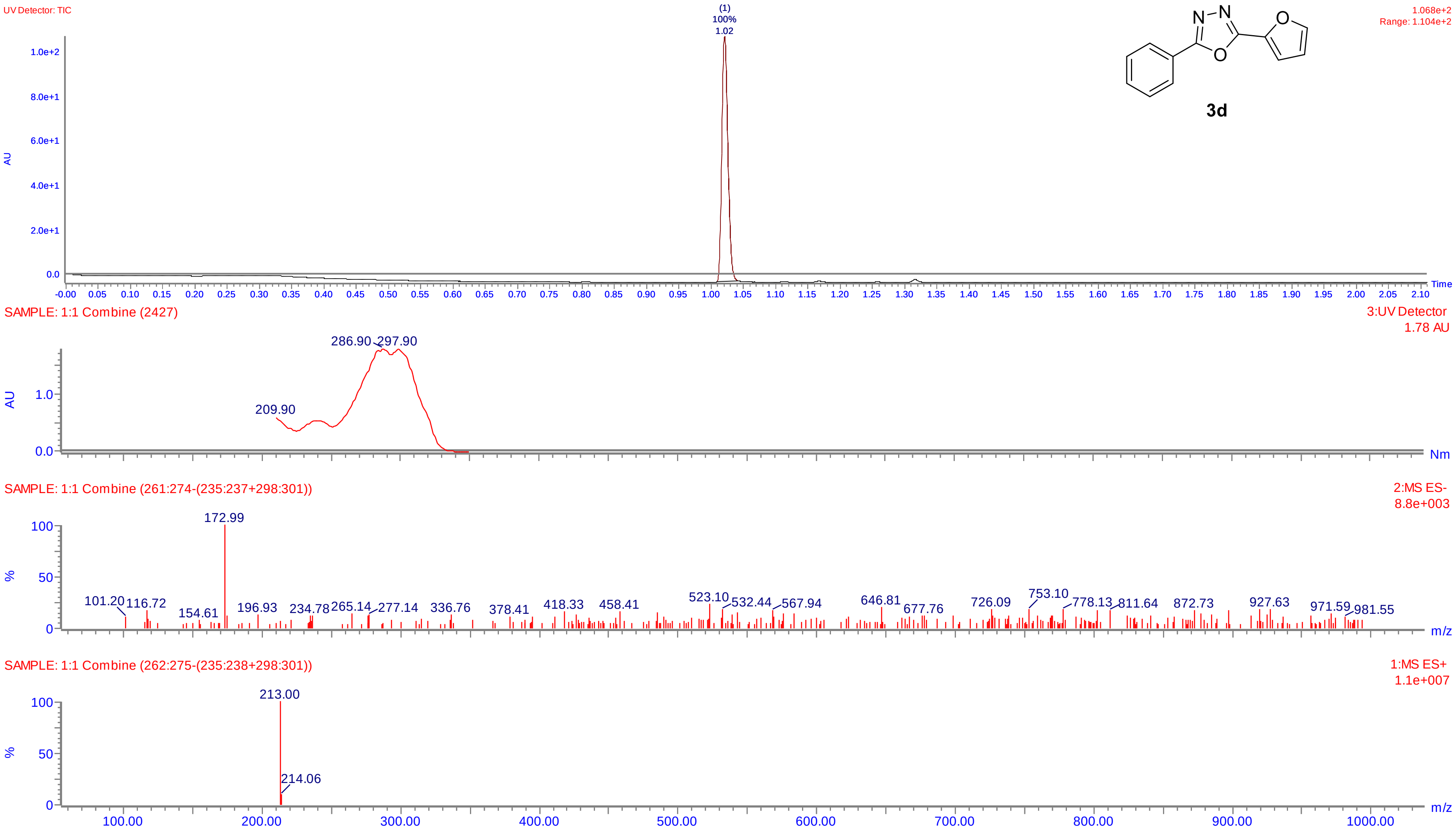
UV Detector: TIC
(1)
100%
1.02
1.068e+2
Range: 1.104e+2
3d
SAMPLE: 1:1 Combine (2427)
3:UV Detector
1.78 AU
286.90
297.90
209.90
SAMPLE: 1:1 Combine (261:274-(235:237+298:301))
2:MS ES-
8.8e+003
172.99
SAMPLE: 1:1 Combine (262:275-(235:238+298:301))
1:MS ES+
1.1e+007
213.00
214.06
m/z

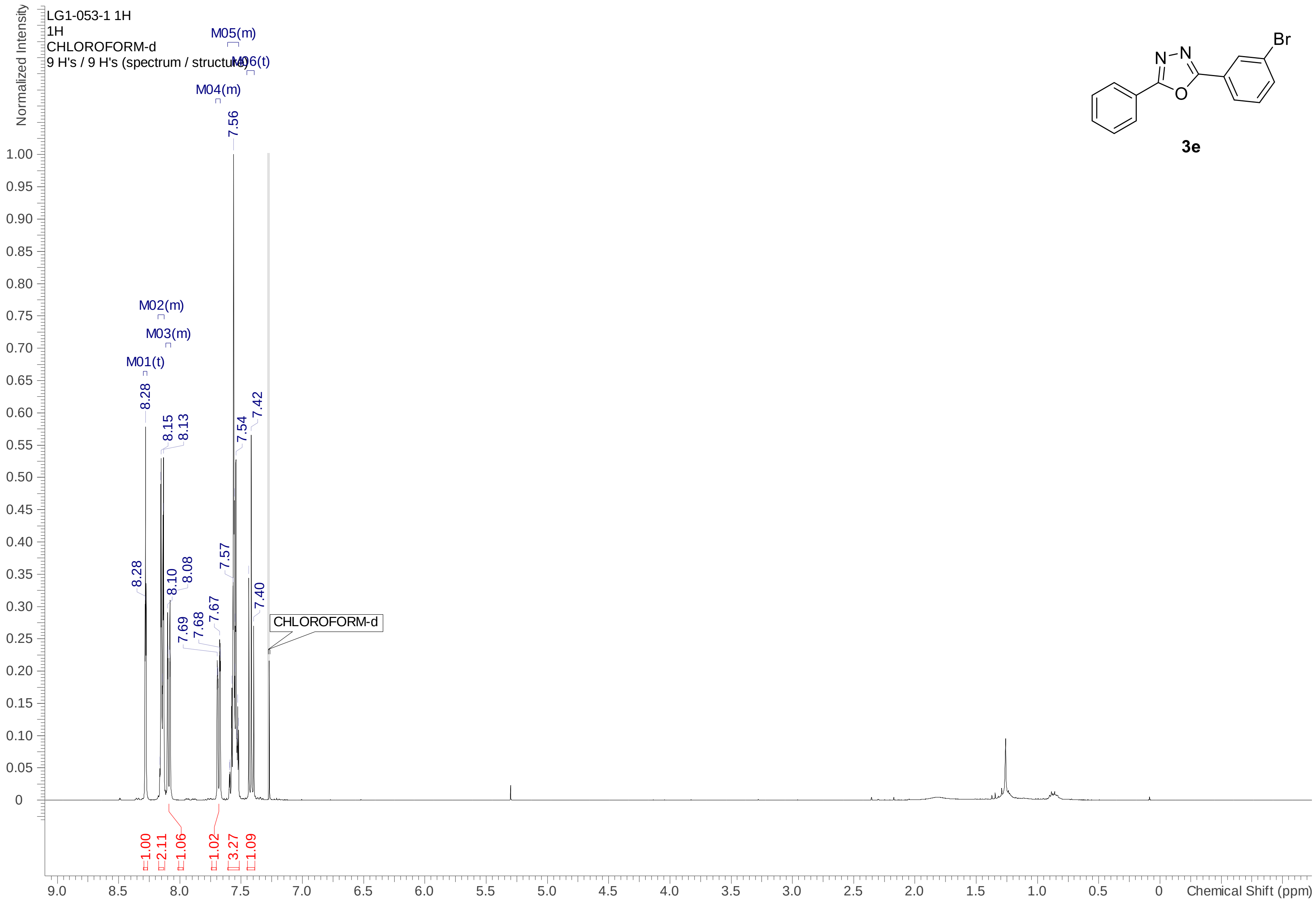

LG1-053-1 1H
1H
CHLOROFORM-d
9 H's / 9 H's (spectrum / structure)
Normalized Intensity
M01(t)
M02(m)
M03(m)
M04(m)
M05(m)
M06(t)
8.28
8.15
8.13
8.28
8.10
8.08
7.69
7.68
7.67
7.57
7.56
7.54
7.42
7.40
CHLOROFORM-d
1.00
2.11
1.06
1.02
3.27
1.09
Chemical Shift (ppm)
N–N
O
Br

3e

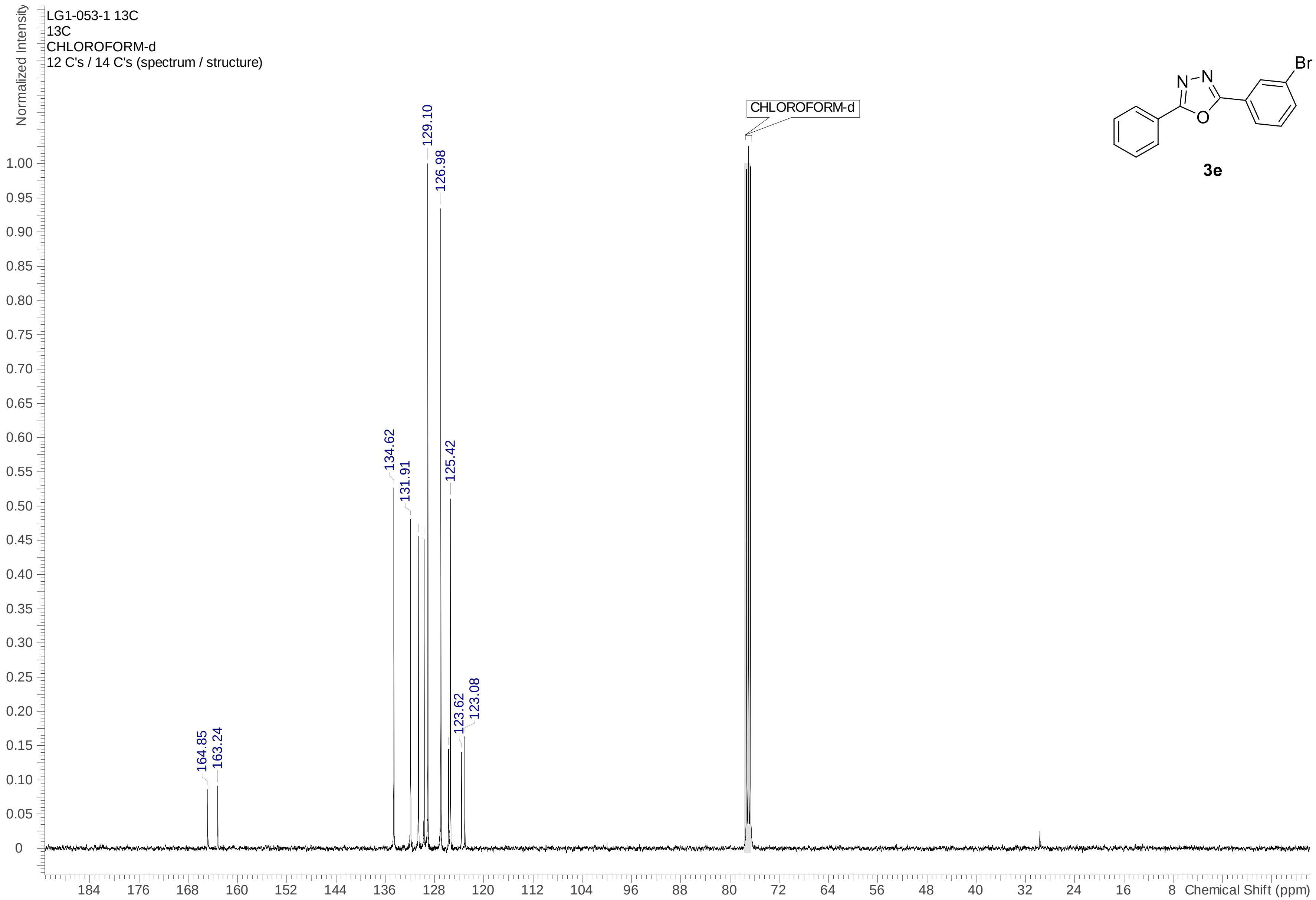

LG1-053-1 13C
13C
CHLOROFORM-d
12 C's / 14 C's (spectrum / structure)
Normalized Intensity
164.85
163.24
134.62
131.91
129.10
126.98
125.42
123.62
123.08
CHLOROFORM-d
Chemical Shift (ppm)
Br
N
N
O

3e

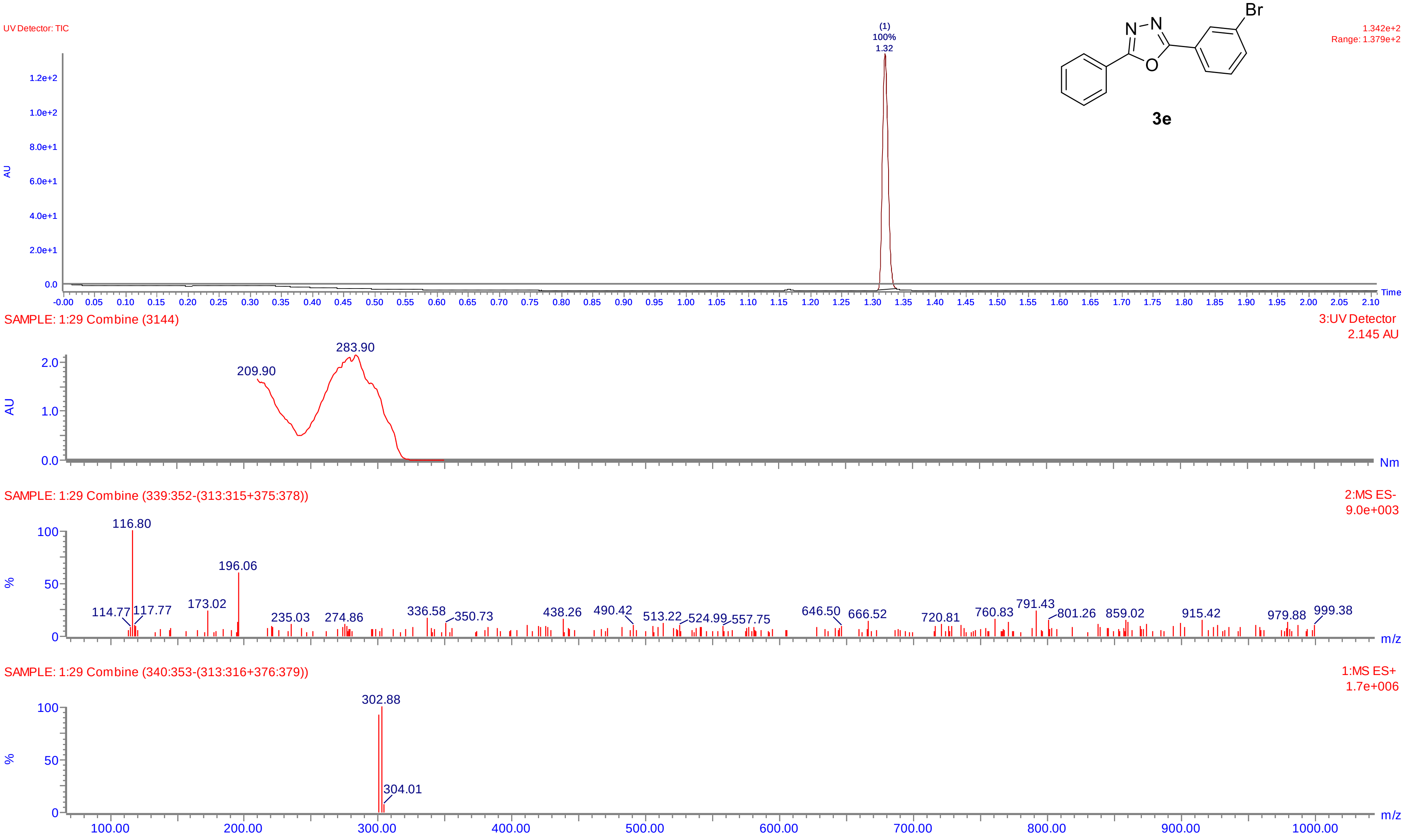
UV Detector: TIC
(1)
100%
1.32
Br
N
N
O
1.342e+2
Range: 1.379e+2
AU
Time
SAMPLE: 1:29 Combine (3144)
3:UV Detector
2.145 AU
209.90
283.90
Nm
SAMPLE: 1:29 Combine (339:352-(313:315+375:378))
2:MS ES-
9.0e+003
116.80
196.06
114.77
117.77
173.02
235.03
274.86
336.58
350.73
438.26
490.42
513.22
524.99
557.75
646.50
666.52
720.81
760.83
791.43
801.26
859.02
915.42
979.88
999.38
m/z
SAMPLE: 1:29 Combine (340:353-(313:316+376:379))
1:MS ES+
1.7e+006
302.88
304.01
%
m/z
100.00
200.00
300.00
400.00
500.00
600.00
700.00
800.00
900.00
1000.00

3e

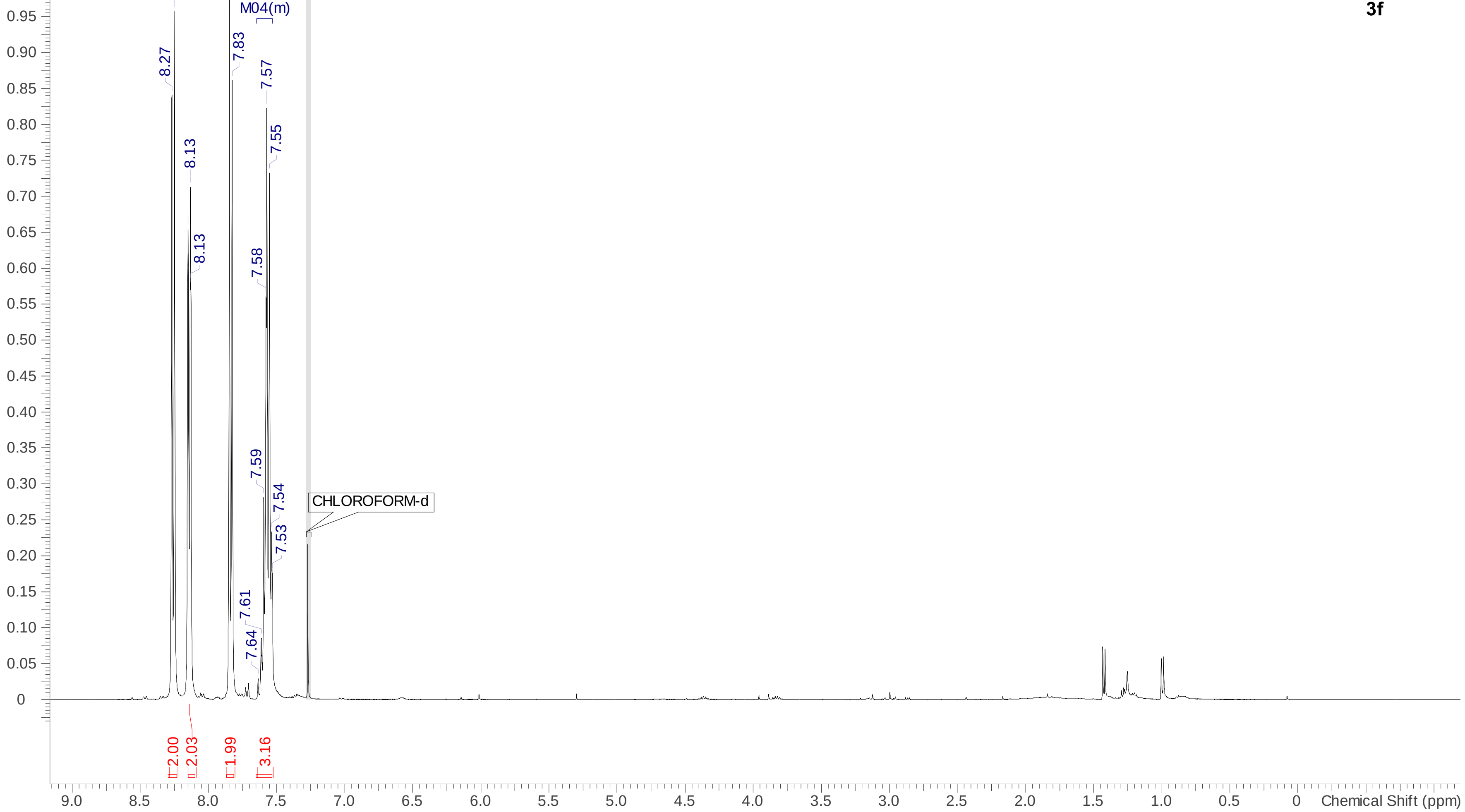
LG1-031-1 1H
1H
CHLOROFORM-d
9 H's / 9 H's (spectrum / structure)
Normalized Intensity
M01(d)
M02(dd)
M03(d)
M04(m)
8.27
8.25
8.13
8.13
7.85
7.83
7.64
7.61
7.59
7.58
7.57
7.55
7.54
7.53
CHLOROFORM-d
2.00
2.03
1.99
3.16
Chemical Shift (ppm)

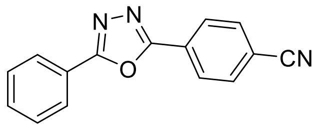
N
N
O
CN

3f

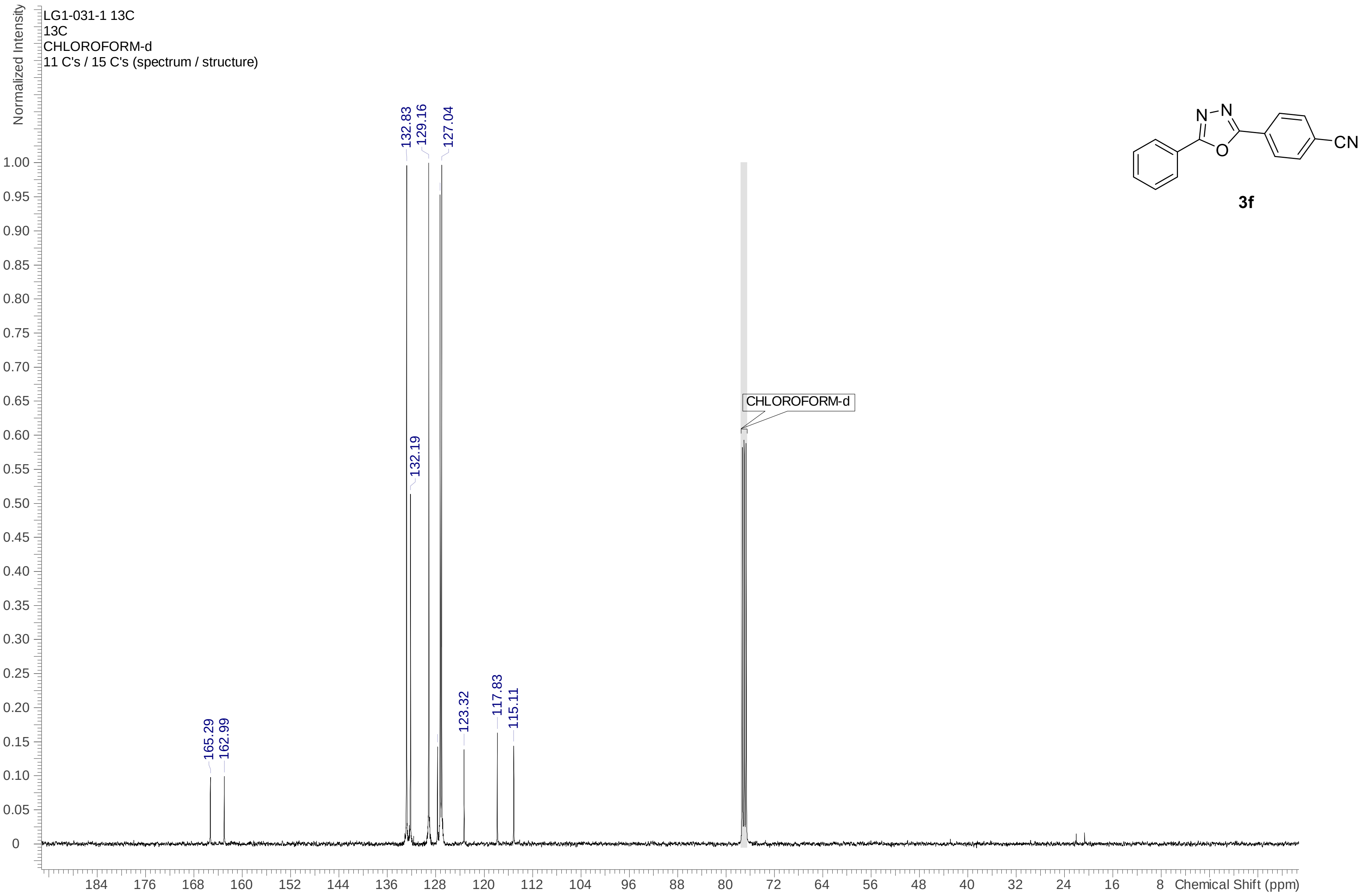
LG1-031-1 13C
13C
CHLOROFORM-d
11 C's / 15 C's (spectrum / structure)
Normalized Intensity
165.29
162.99
132.83
132.19
129.16
127.04
123.32
117.83
115.11
CHLOROFORM-d
Chemical Shift (ppm)
N
N
O
CN

3f

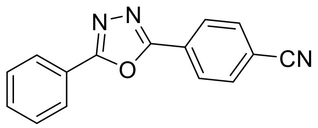
N
N
O
CN

3f

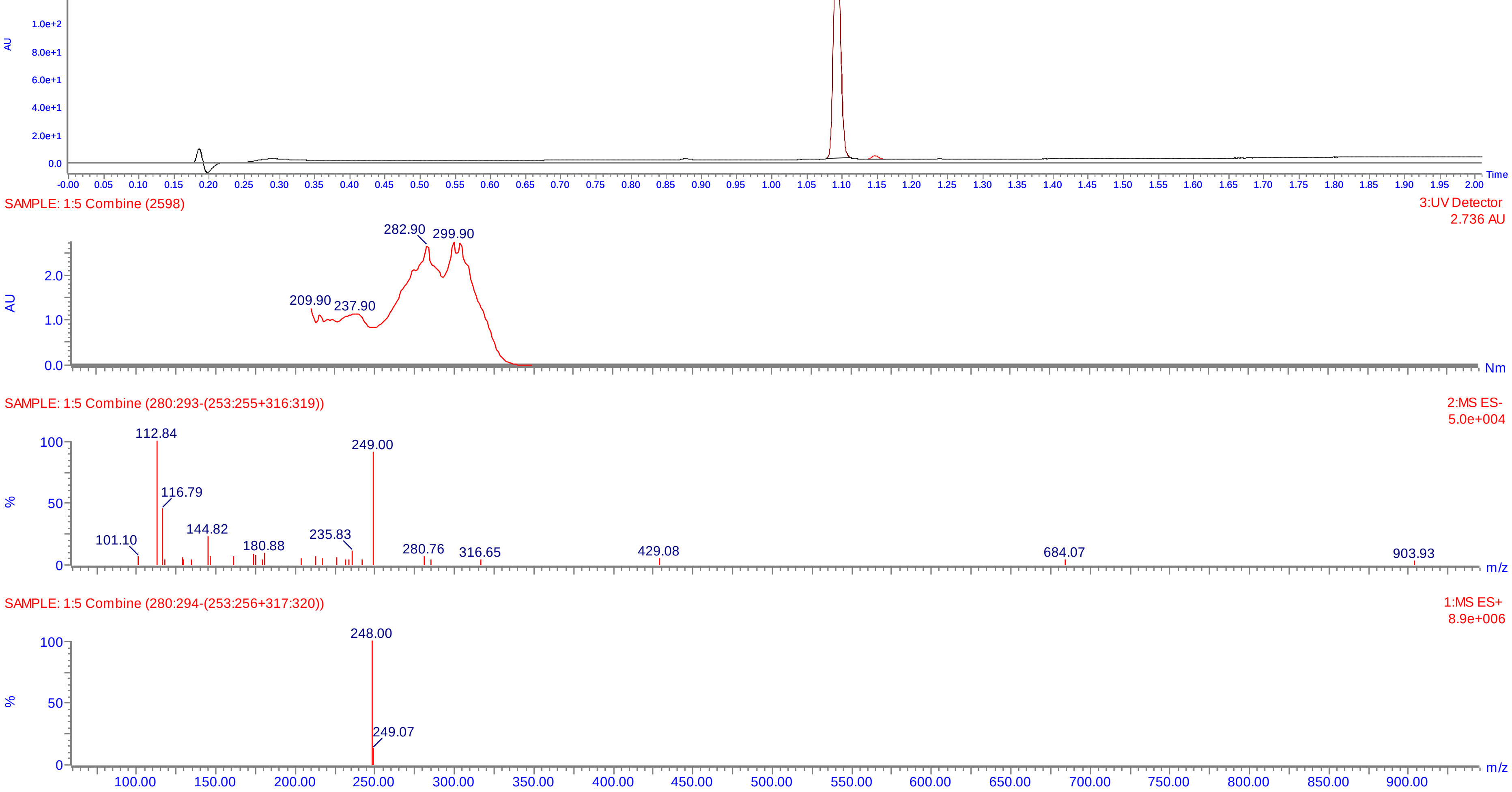
UV Detector: TIC
(1)
99%
1.09
1.765e+2
Range: 1.833e+2
AU
Time
SAMPLE: 1:5 Combine (2598)
3:UV Detector
2.736 AU
282.90
299.90
209.90
237.90
Nm
SAMPLE: 1:5 Combine (280:293-(253:255+316:319))
2:MS ES-
5.0e+004
112.84
249.00
116.79
144.82
101.10
180.88
235.83
280.76
316.65
429.08
684.07
903.93
m/z
SAMPLE: 1:5 Combine (280:294-(253:256+317:320))
1:MS ES+
8.9e+006
248.00
249.07
%
m/z

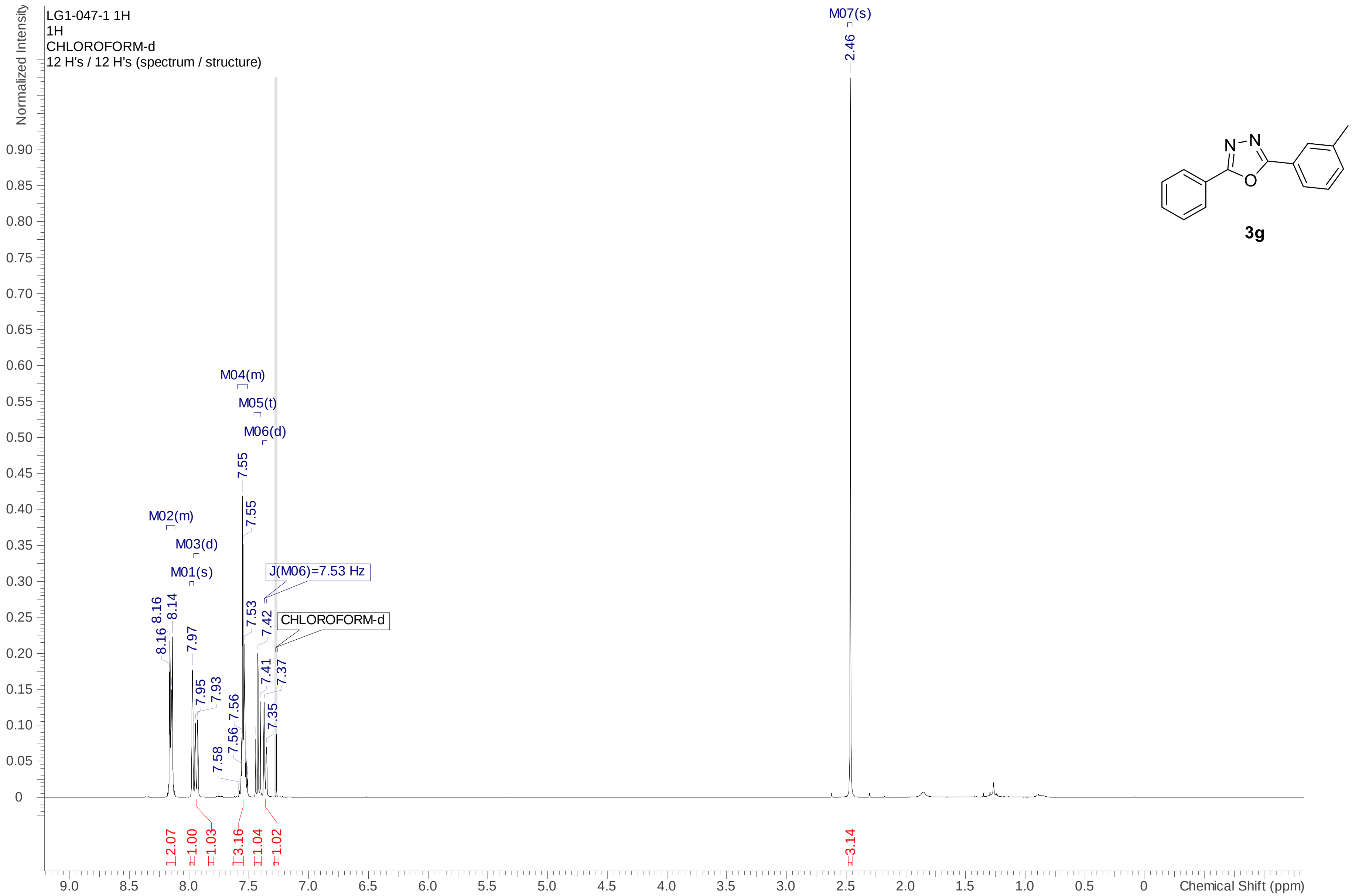

LG1-047-1 1H
1H
CHLOROFORM-d
12 H's / 12 H's (spectrum / structure)
Normalized Intensity
M07(s)
2.46
M04(m)
M05(t)
M06(d)
M02(m)
M03(d)
M01(s)
J(M06)=7.53 Hz
CHLOROFORM-d
8.16
8.16
8.14
7.97
7.95
7.93
7.58
7.56
7.56
7.55
7.55
7.53
7.42
7.41
7.37
7.35
2.07
1.00
1.03
3.16
1.04
1.02
3.14
Chemical Shift (ppm)
N
N
O

3g

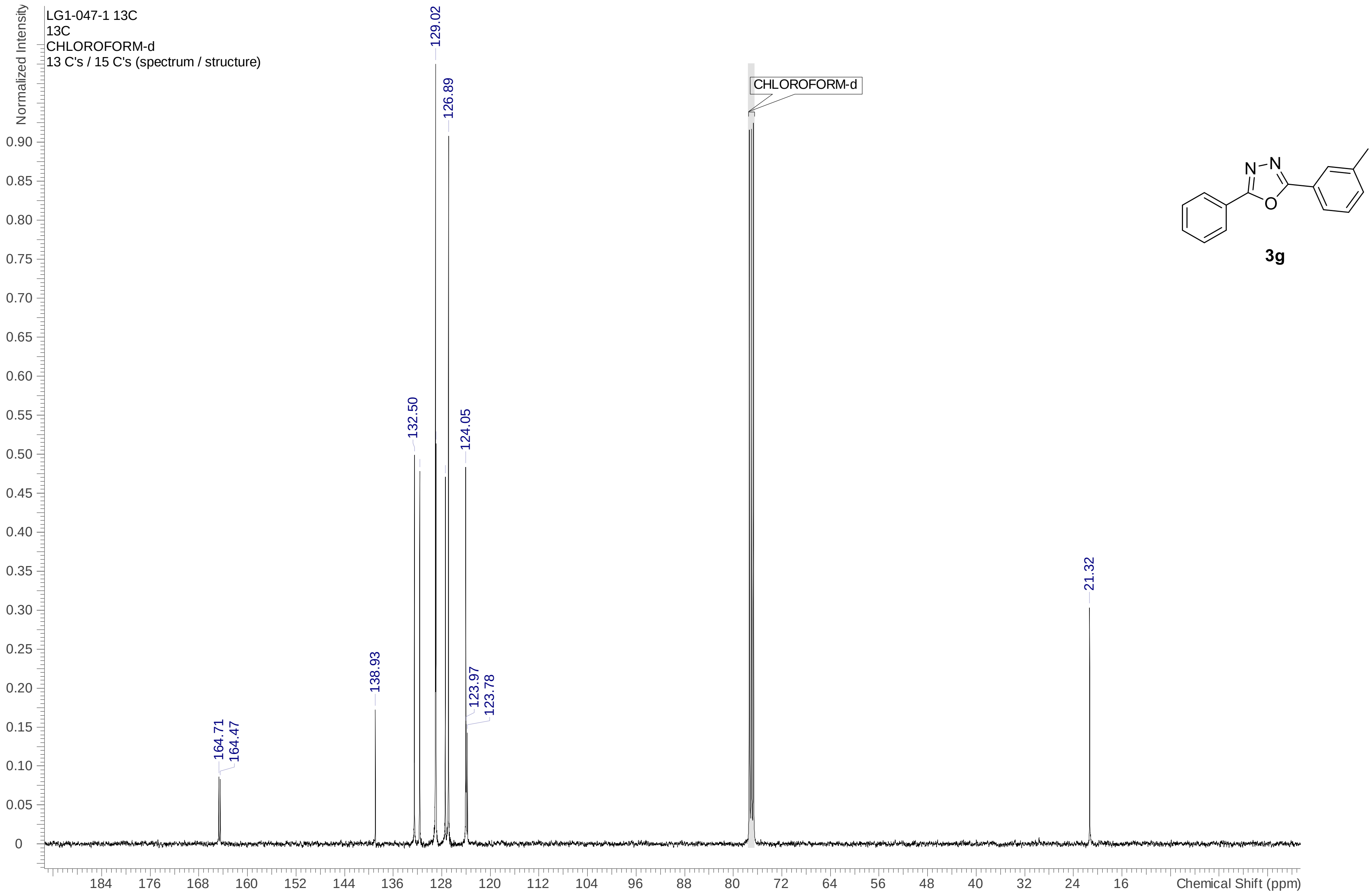

LG1-047-1 13C
13C
CHLOROFORM-d
13 C's / 15 C's (spectrum / structure)
Normalized Intensity
129.02
126.89
132.50
124.05
138.93
123.97
123.78
164.71
164.47
21.32
CHLOROFORM-d
Chemical Shift (ppm)
N
N
O

3g

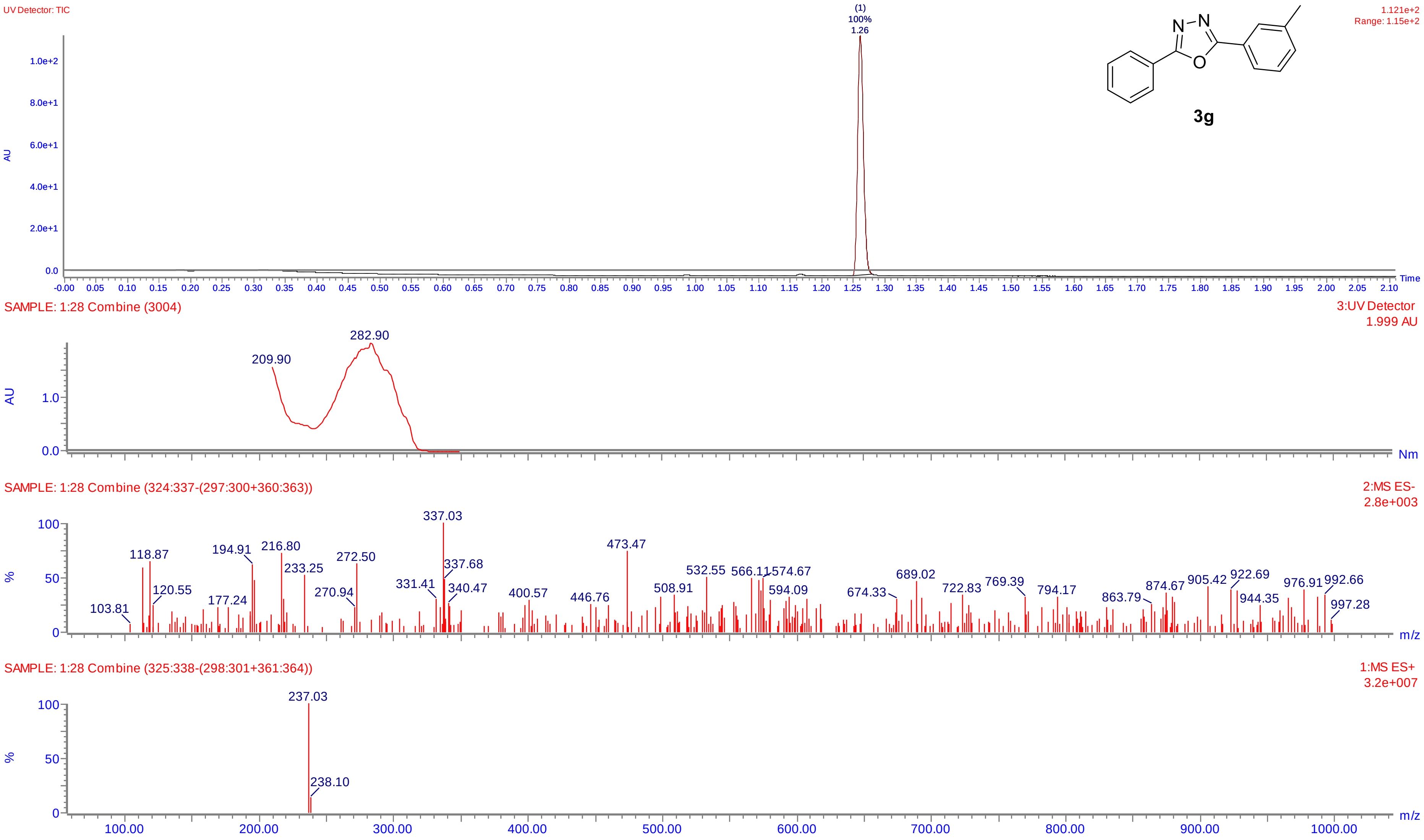
UV Detector: TIC
(1)
100%
1.26
1.121e+2
Range: 1.15e+2
AU
Time
3g
SAMPLE: 1:28 Combine (3004)
3:UV Detector
1.999 AU
209.90
282.90
Nm
SAMPLE: 1:28 Combine (324:337-(297:300+360:363))
2:MS ES-
2.8e+003
337.03
473.47
m/z
SAMPLE: 1:28 Combine (325:338-(298:301+361:364))
1:MS ES+
3.2e+007
237.03
238.10
m/z

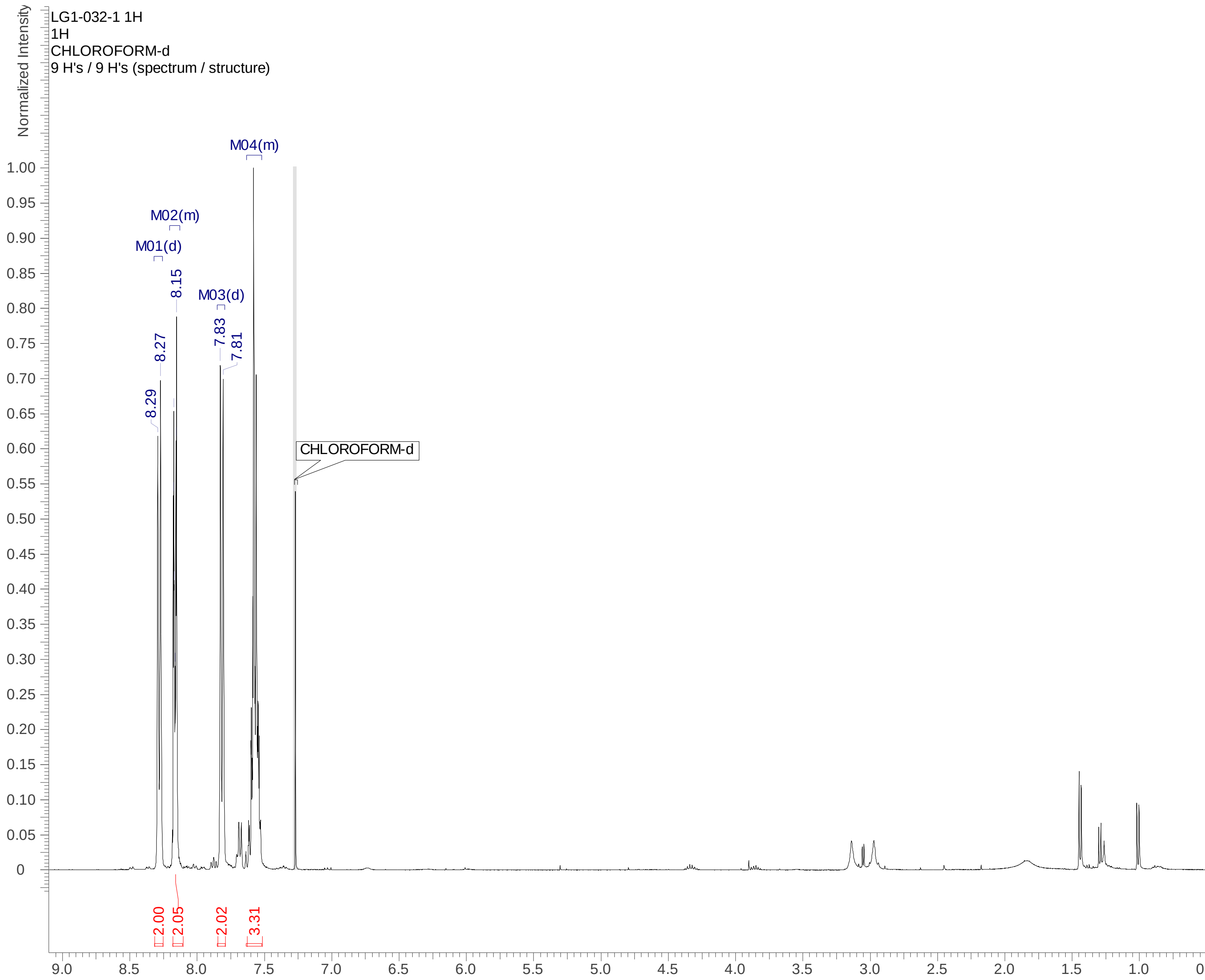
LG1-032-1 1H
1H
CHLOROFORM-d
9 H's / 9 H's (spectrum / structure)
Normalized Intensity
M01(d)
M02(m)
M03(d)
M04(m)
8.29
8.27
8.15
7.83
7.81
CHLOROFORM-d
2.00
2.05
2.02
3.31
Chemical Shift (ppm)

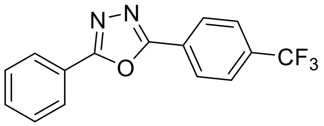
N
N
O
CF3

3h

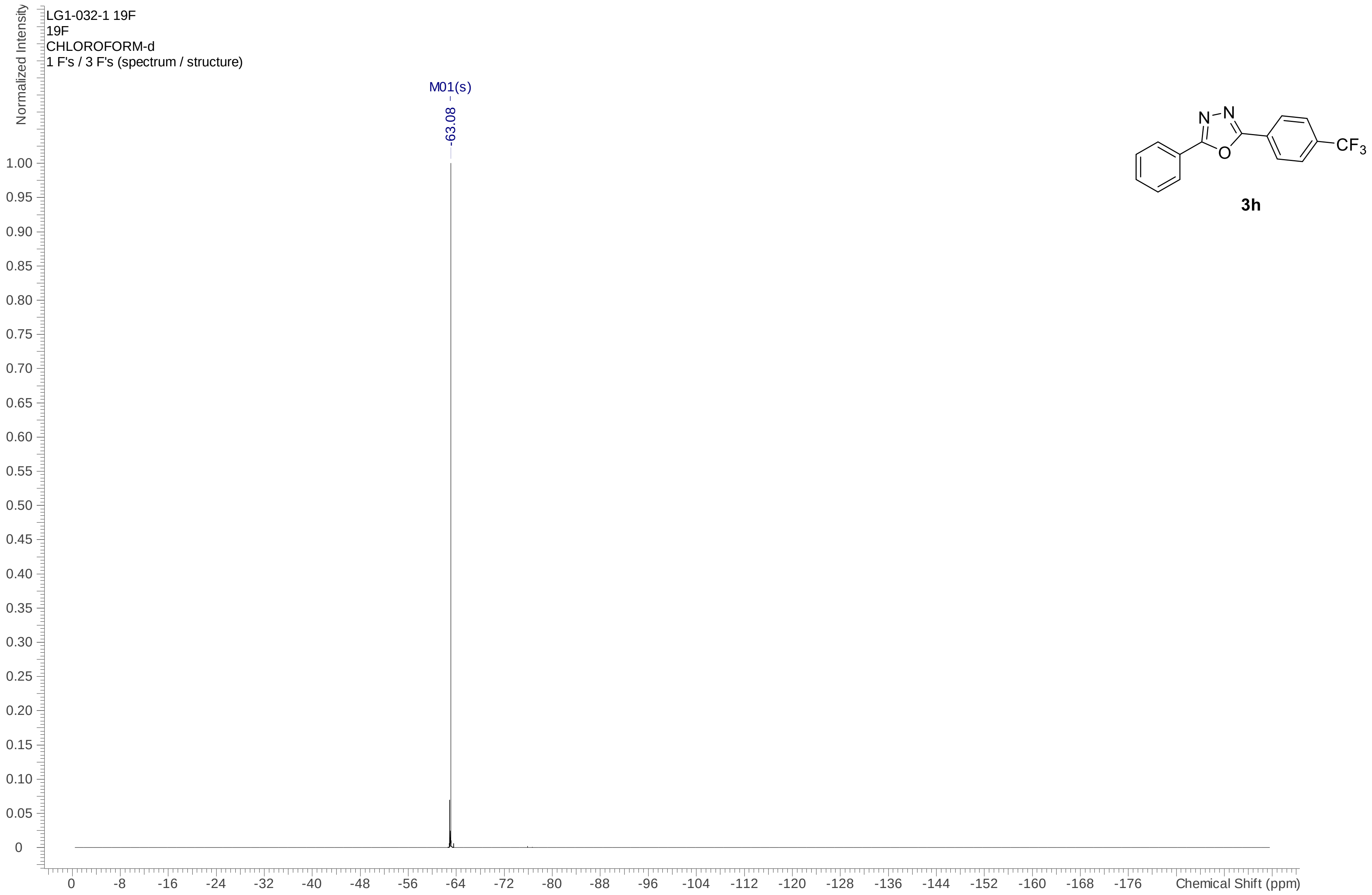
LG1-032-1 19F
19F
CHLOROFORM-d
1 F's / 3 F's (spectrum / structure)
M01(s)
-63.08
Normalized Intensity
Chemical Shift (ppm)
3h

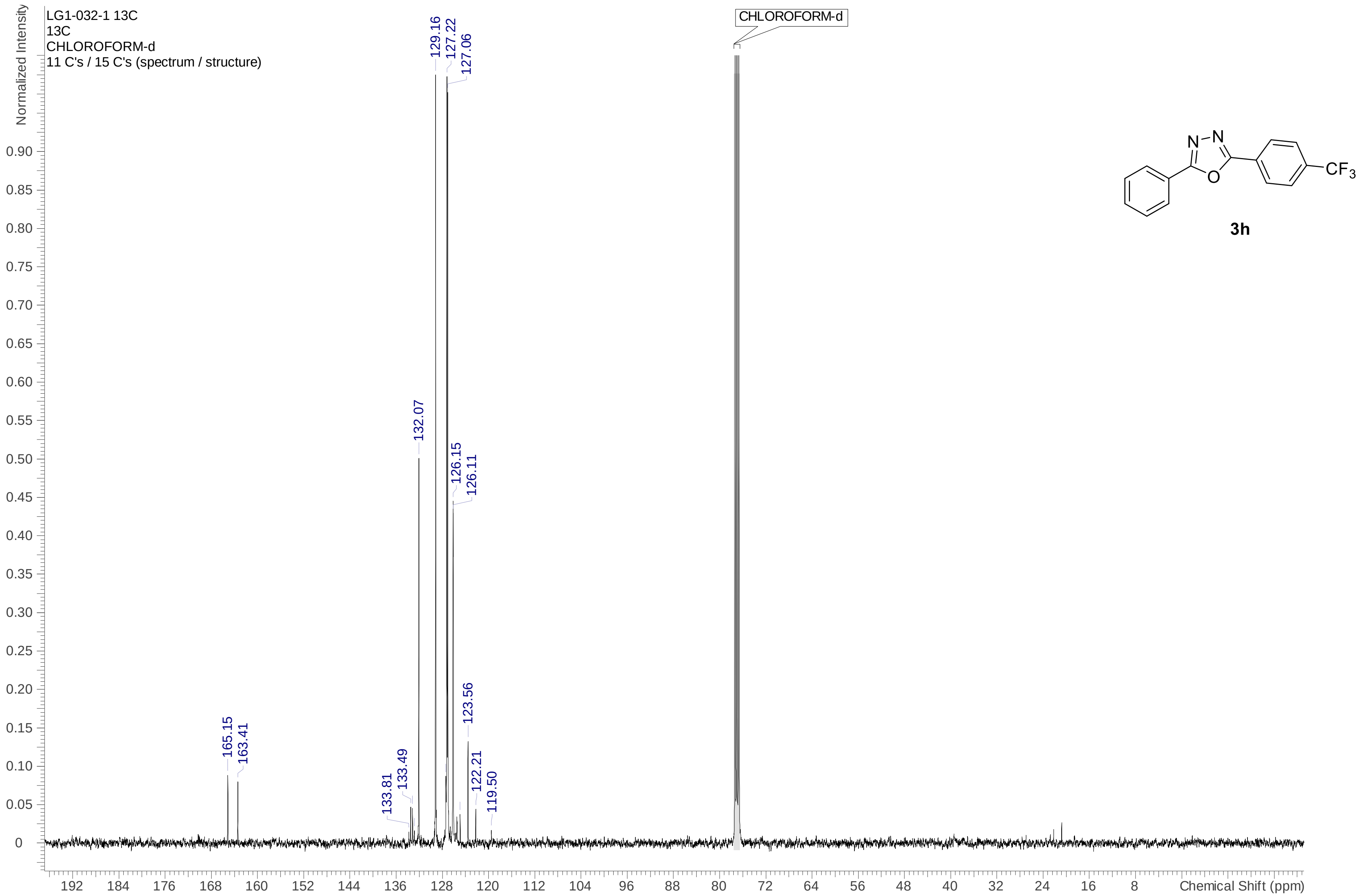

LG1-032-1 13C
13C
CHLOROFORM-d
11 C's / 15 C's (spectrum / structure)
Normalized Intensity
CHLOROFORM-d
129.16
127.22
127.06
132.07
126.15
126.11
123.56
165.15
163.41
133.81
133.49
122.21
119.50
0.90
0.85
0.80
0.75
0.70
0.65
0.60
0.55
0.50
0.45
0.40
0.35
0.30
0.25
0.20
0.15
0.10
0.05
0
192
184
176
168
160
152
144
136
128
120
112
104
96
88
80
72
64
56
48
40
32
24
16
8
Chemical Shift (ppm)
N
N
O
CF3

3h

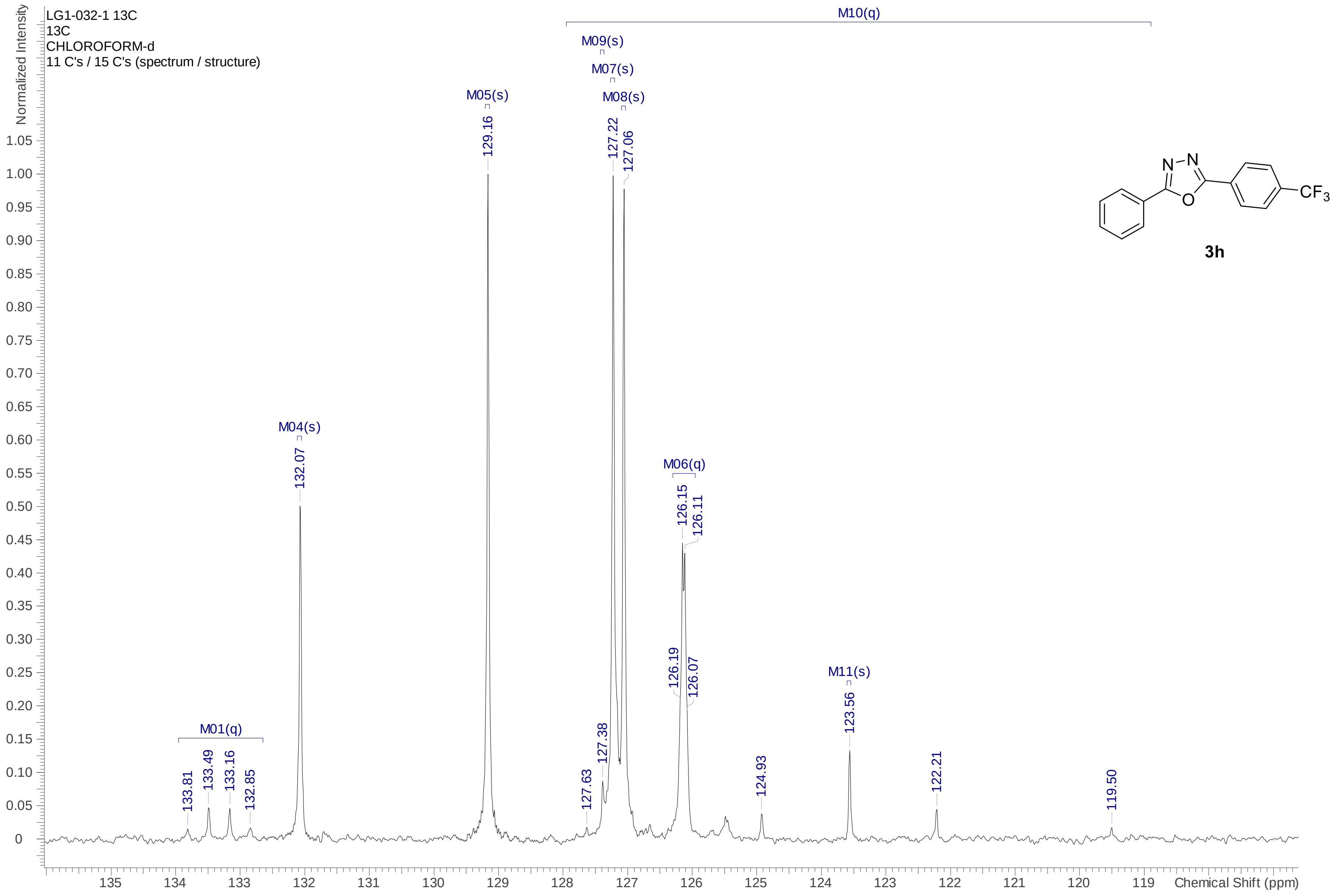

LG1-032-1 13C
13C
CHLOROFORM-d
11 C's / 15 C's (spectrum / structure)
Normalized Intensity
M10(q)
M09(s)
M07(s)
M08(s)
M05(s)
M04(s)
M06(q)
M11(s)
M01(q)
129.16
127.22
127.06
132.07
126.15
126.11
126.19
126.07
123.56
133.81
133.49
133.16
132.85
127.63
127.38
124.93
122.21
119.50
Chemical Shift (ppm)
N
N
O
CF_3

3h

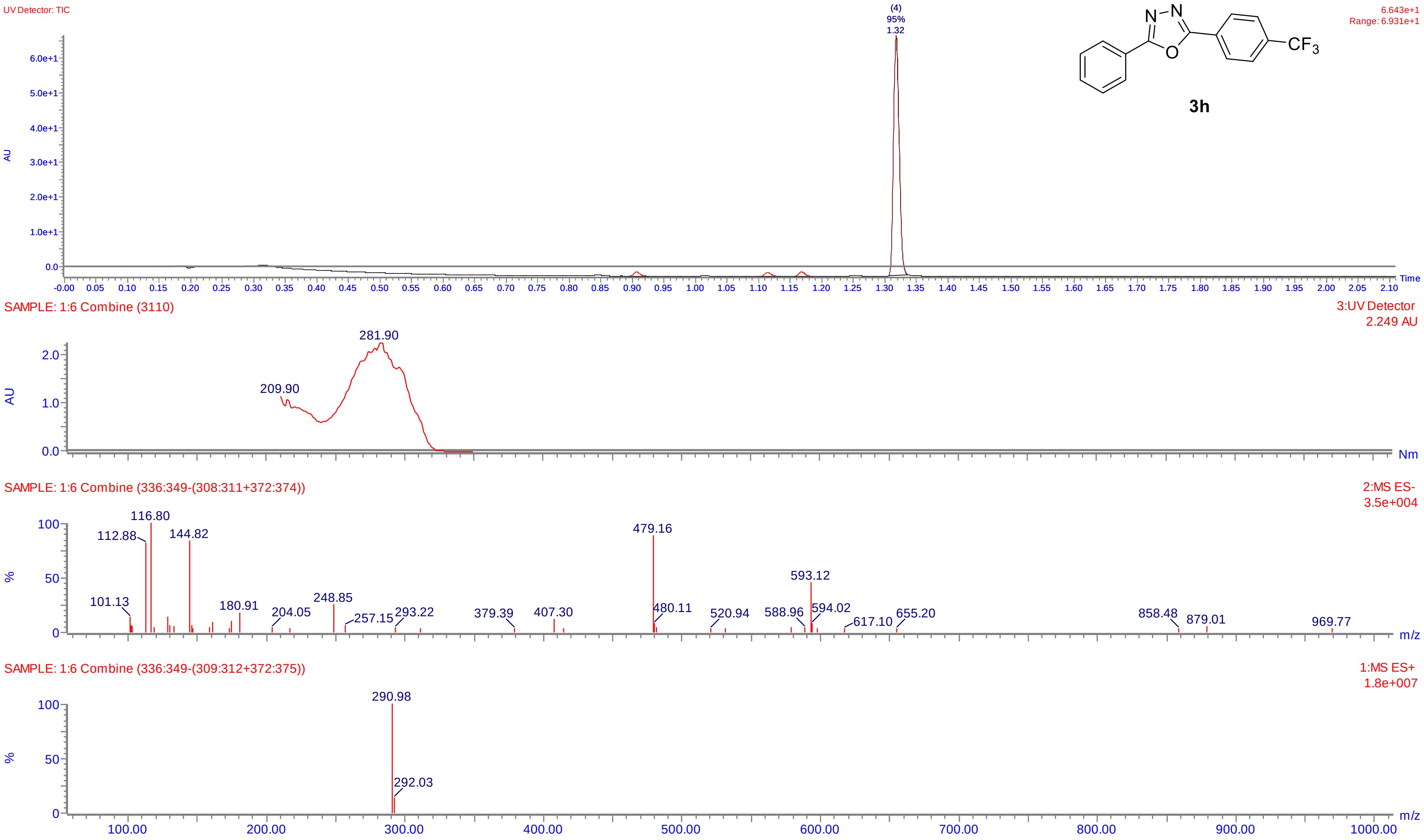
UV Detector: TIC
(4)
95%
1.32
6.643e+1
Range: 6.931e+1
3h
AU
Time
SAMPLE: 1:6 Combine (3110)
3:UV Detector
2.249 AU
281.90
209.90
Nm
SAMPLE: 1:6 Combine (336:349-(308:311+372:374))
2:MS ES-
3.5e+004
116.80
112.88
144.82
479.16
593.12
101.13
180.91
248.85
204.05
257.15
293.22
379.39
407.30
480.11
520.94
588.96
594.02
617.10
655.20
858.48
879.01
969.77
m/z
SAMPLE: 1:6 Combine (336:349-(309:312+372:375))
1:MS ES+
1.8e+007
290.98
292.03
m/z

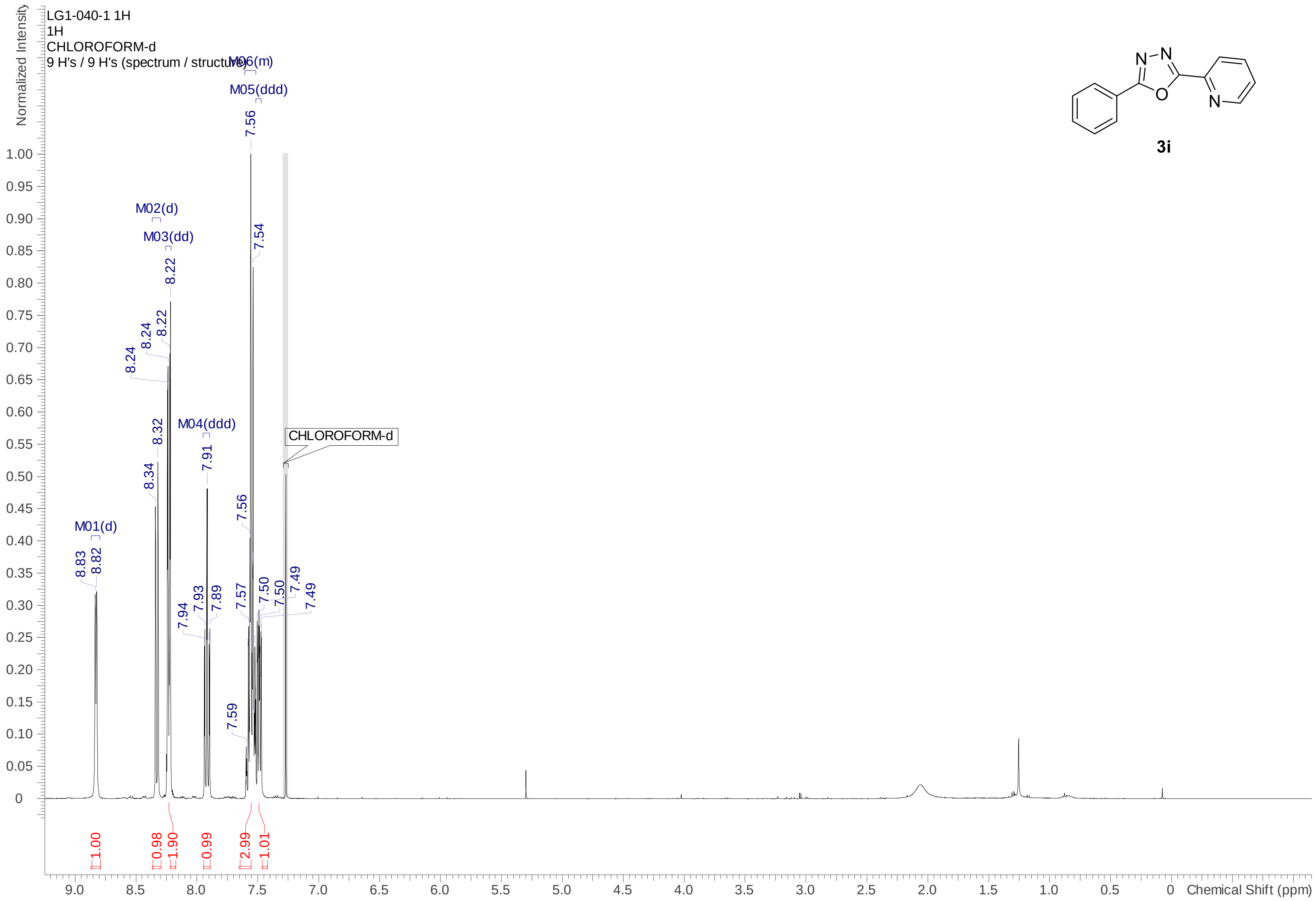

LG1-040-1 1H
1H
CHLOROFORM-d
9 H's / 9 H's (spectrum / structure)
Normalized Intensity
M01(d)
M02(d)
M03(dd)
M04(ddd)
M05(ddd)
M06(m)
CHLOROFORM-d
8.83
8.82
8.34
8.32
8.24
8.24
8.22
8.22
7.94
7.93
7.91
7.89
7.59
7.57
7.56
7.56
7.54
7.50
7.50
7.49
7.49
1.00
0.98
1.90
0.99
2.99
1.01
Chemical Shift (ppm)

3i

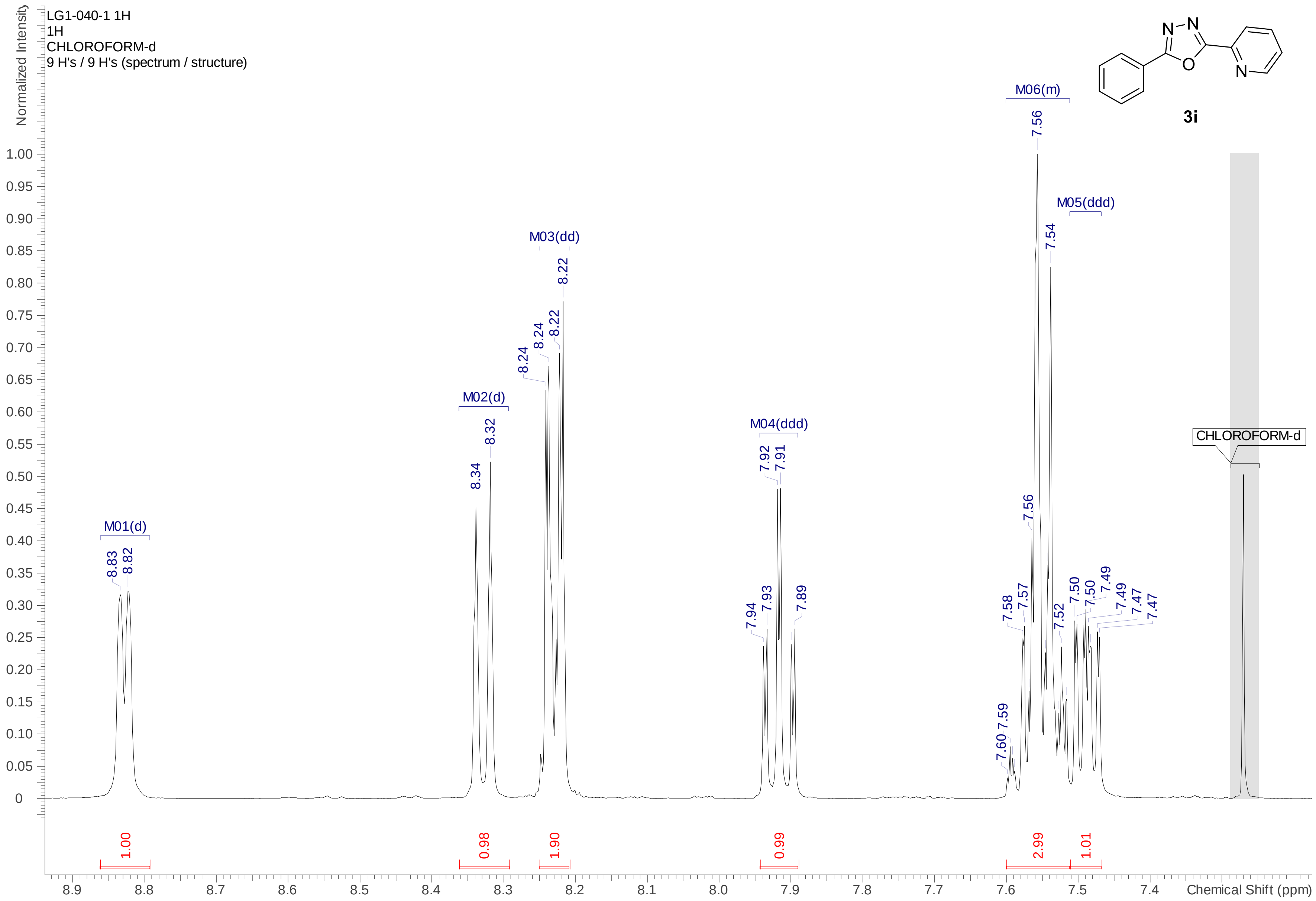

LG1-040-1 1H
1H
CHLOROFORM-d
9 H's / 9 H's (spectrum / structure)
Normalized Intensity
M01(d)
M02(d)
M03(dd)
M04(ddd)
M05(ddd)
M06(m)
CHLOROFORM-d
3i
Chemical Shift (ppm)

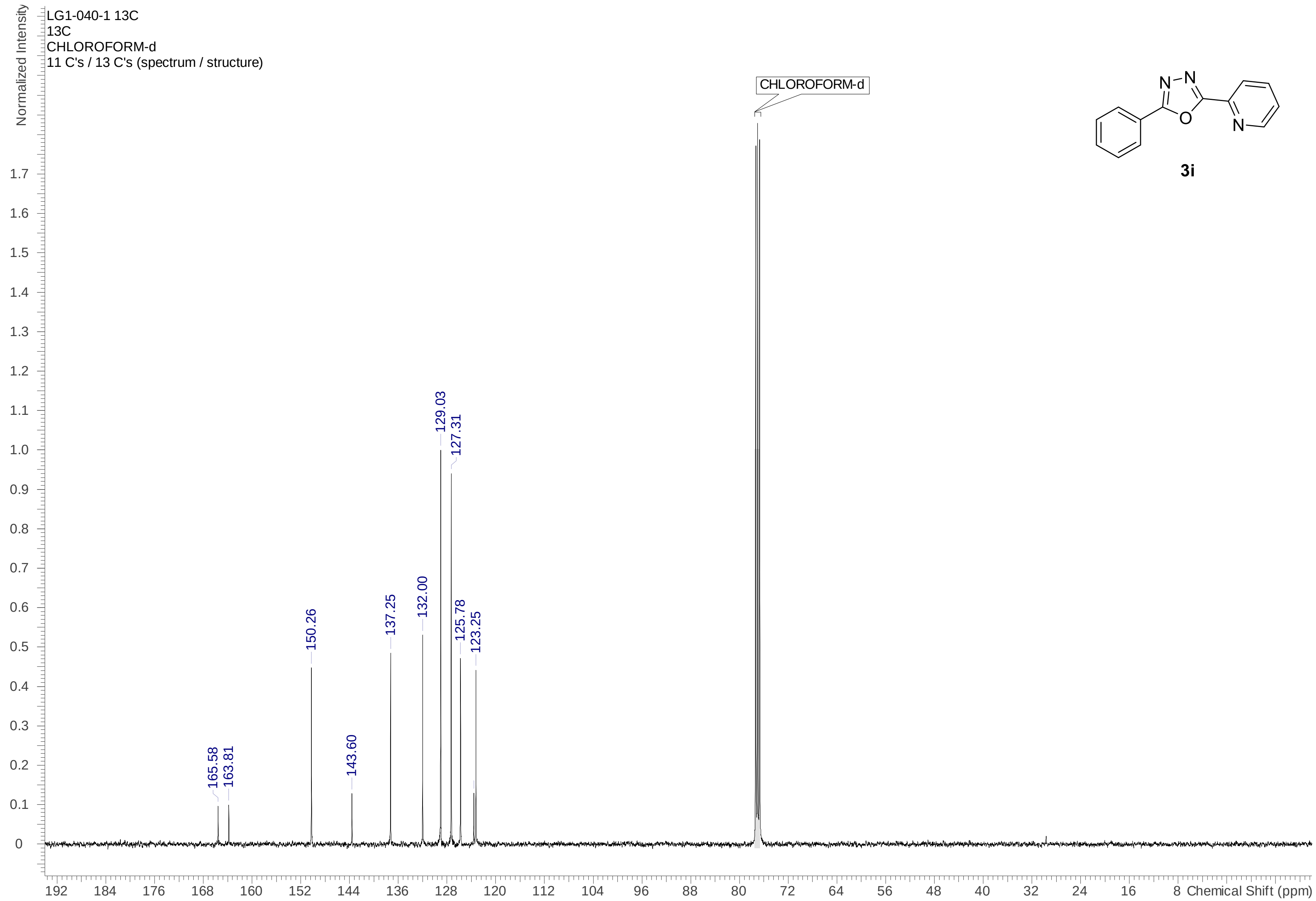

LG1-040-1 13C
13C
CHLOROFORM-d
11 C's / 13 C's (spectrum / structure)
CHLOROFORM-d
3i
165.58
163.81
150.26
143.60
137.25
132.00
129.03
127.31
125.78
123.25
Normalized Intensity
Chemical Shift (ppm)

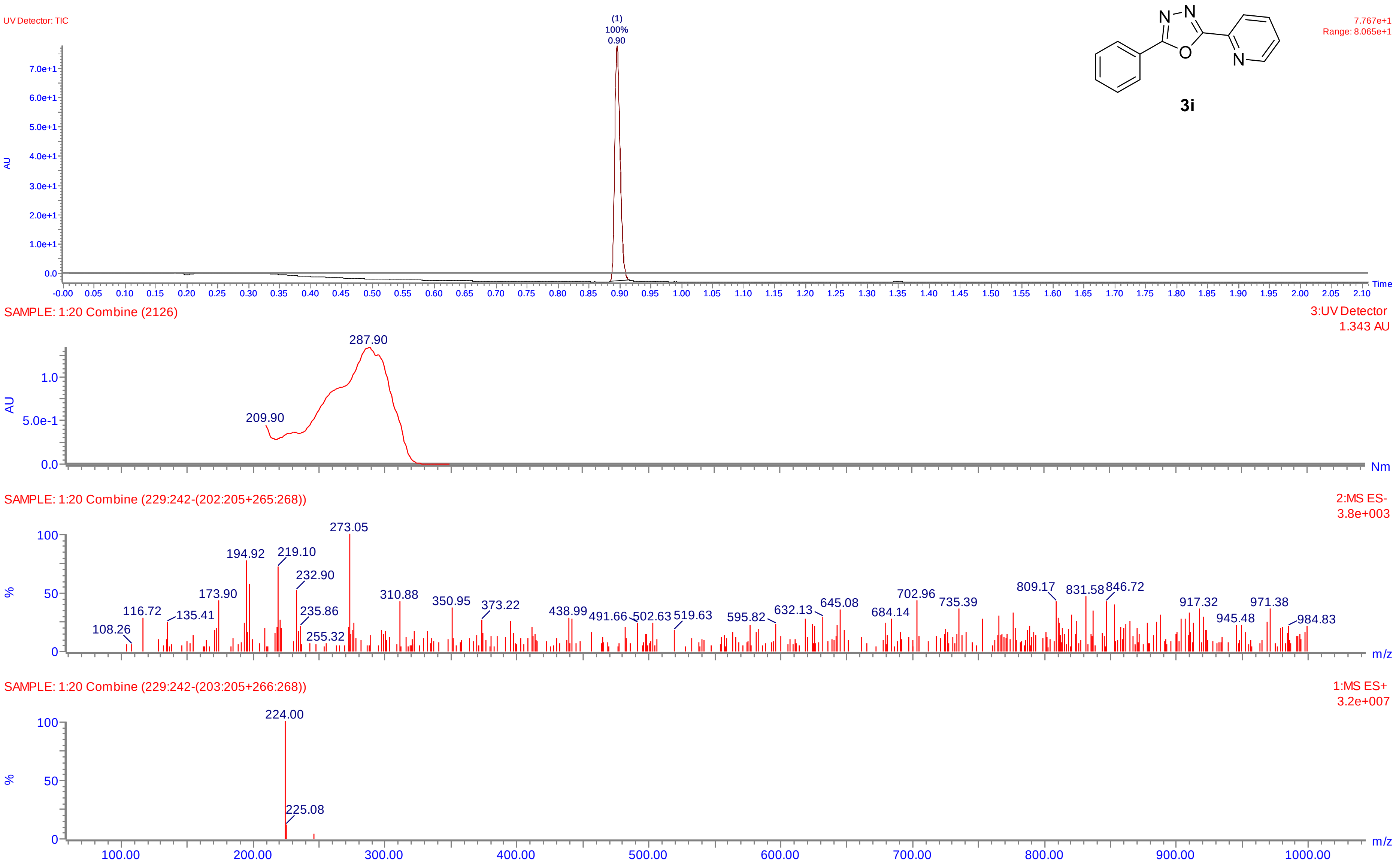
UV Detector: TIC
(1)
100%
0.90
7.767e+1
Range: 8.065e+1
3i
AU
Time
SAMPLE: 1:20 Combine (2126)
3:UV Detector
1.343 AU
287.90
209.90
Nm
SAMPLE: 1:20 Combine (229:242-(202:205+265:268))
2:MS ES-
3.8e+003
273.05
194.92
219.10
232.90
173.90
116.72
135.41
108.26
235.86
255.32
310.88
350.95
373.22
438.99
491.66
502.63
519.63
595.82
632.13
645.08
684.14
702.96
735.39
809.17
831.58
846.72
917.32
945.48
971.38
984.83
m/z
SAMPLE: 1:20 Combine (229:242-(203:205+266:268))
1:MS ES+
3.2e+007
224.00
225.08
%
m/z

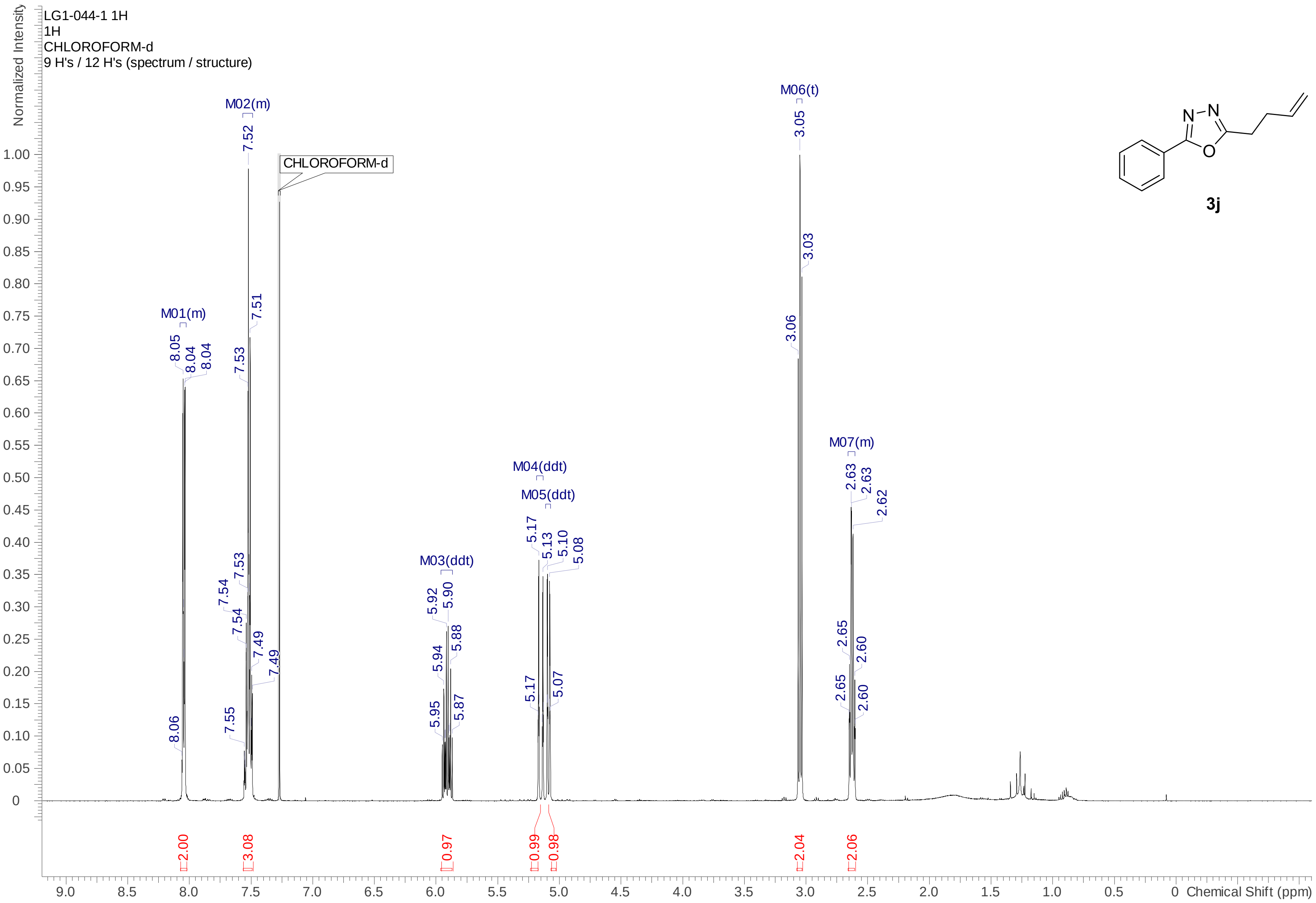
LG1-044-1 1H
1H
CHLOROFORM-d
9 H's / 12 H's (spectrum / structure)
Normalized Intensity
M01(m)
8.05
8.04
8.04
8.06
M02(m)
7.52
7.51
7.53
7.53
7.54
7.54
7.55
7.49
7.49
CHLOROFORM-d
M03(ddt)
5.92
5.90
5.88
5.94
5.95
5.87
M04(ddt)
M05(ddt)
5.17
5.13
5.10
5.08
5.17
5.07
M06(t)
3.05
3.03
3.06
M07(m)
2.63
2.63
2.62
2.65
2.60
2.65
2.60
2.00
3.08
0.97
0.99
0.98
2.04
2.06
Chemical Shift (ppm)
N
N
O
3j

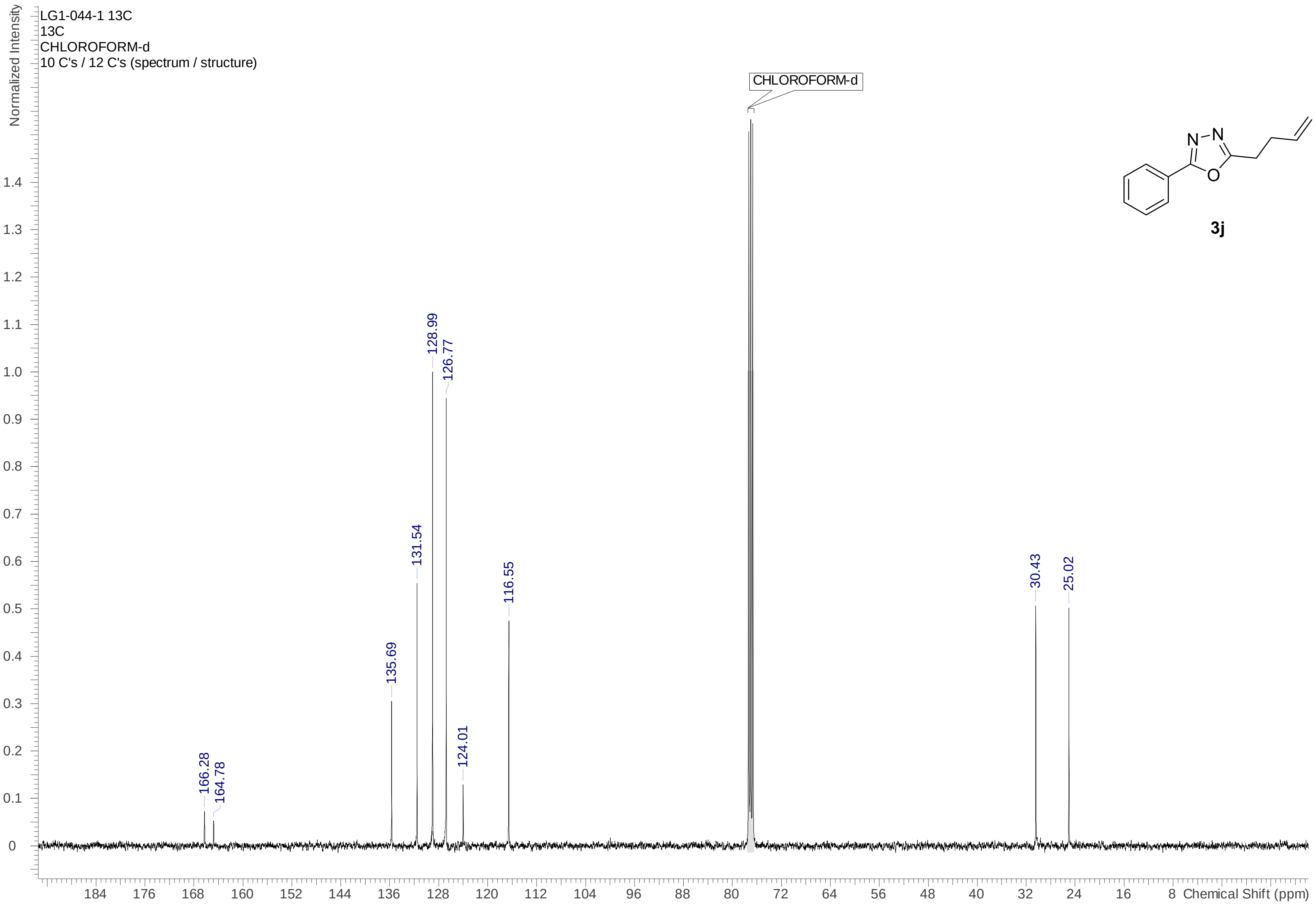
LG1-044-1 13C
13C
CHLOROFORM-d
10 C's / 12 C's (spectrum / structure)
CHLOROFORM-d
Normalized Intensity
166.28
164.78
135.69
131.54
128.99
126.77
124.01
116.55
30.43
25.02
Chemical Shift (ppm)
N
N
O

3j

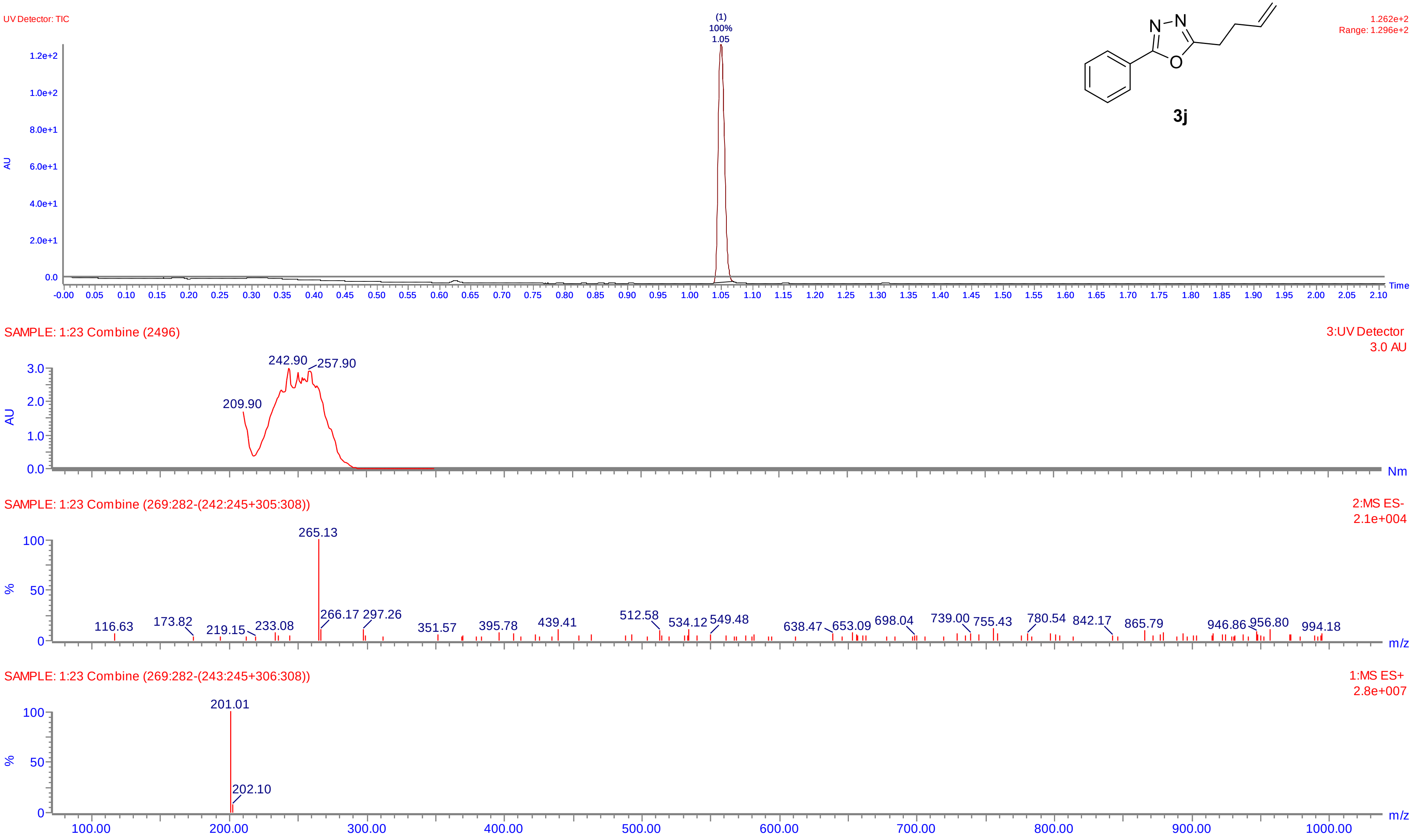

UV Detector: TIC
(1)
100%
1.05
1.262e+2
Range: 1.296e+2
AU
Time
SAMPLE: 1:23 Combine (2496)
3:UV Detector
3.0 AU
242.90
257.90
209.90
AU
Nm
SAMPLE: 1:23 Combine (269:282-(242:245+305:308))
2:MS ES-
2.1e+004
265.13
116.63
173.82
219.15
233.08
266.17
297.26
351.57
395.78
439.41
512.58
534.12
549.48
638.47
653.09
698.04
739.00
755.43
780.54
842.17
865.79
946.86
956.80
994.18
%
m/z
SAMPLE: 1:23 Combine (269:282-(243:245+306:308))
1:MS ES+
2.8e+007
201.01
202.10
%
m/z
N
N
O

3j

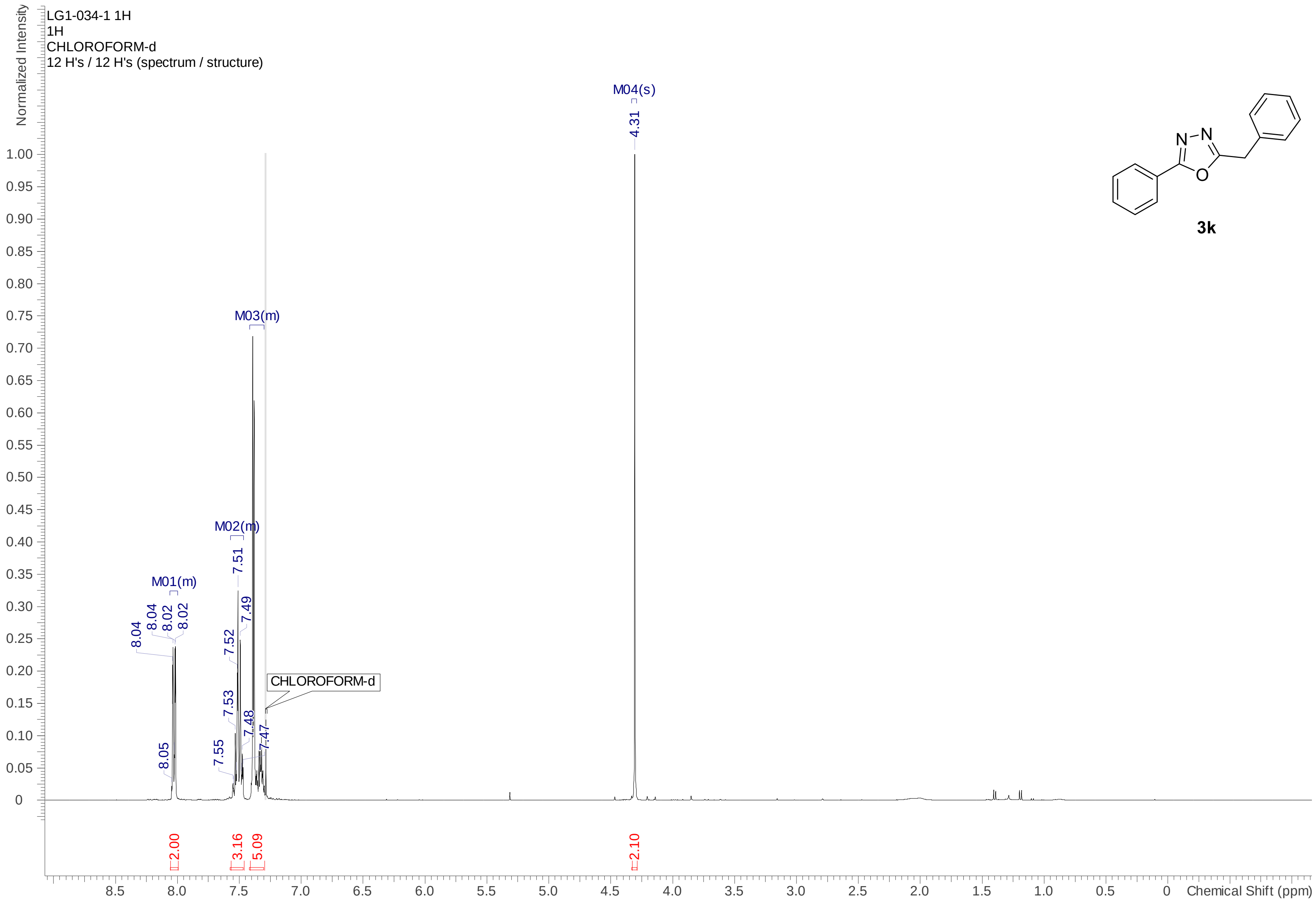
LG1-034-1 1H
1H
CHLOROFORM-d
12 H's / 12 H's (spectrum / structure)
Normalized Intensity
M04(s)
4.31
M03(m)
M02(m)
7.51
M01(m)
8.04
8.04
8.02
8.02
7.49
7.52
7.53
7.48
7.47
7.55
8.05
CHLOROFORM-d
2.00
3.16
5.09
2.10
Chemical Shift (ppm)
N
N
O
3k

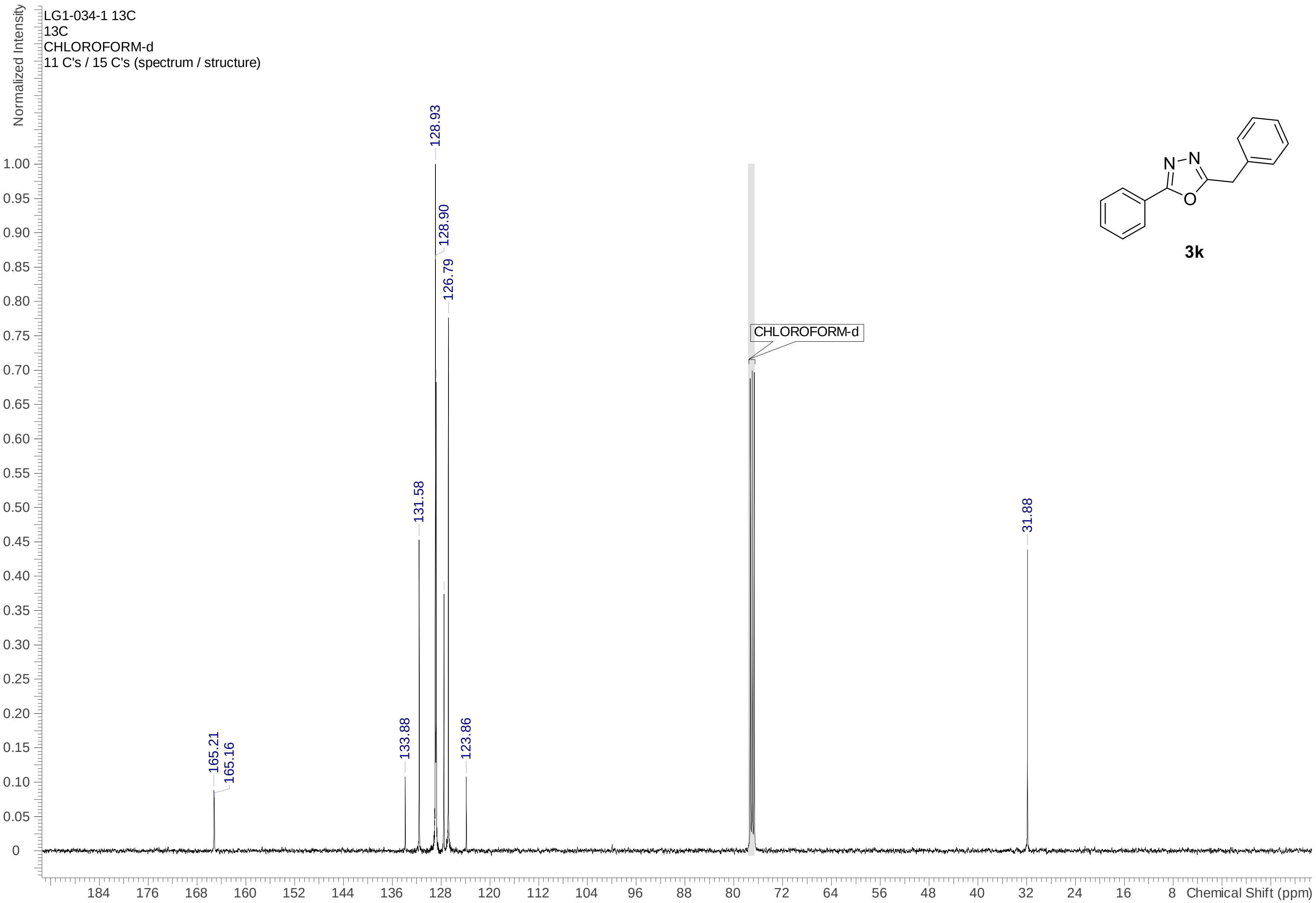

LG1-034-1 13C
13C
CHLOROFORM-d
11 C's / 15 C's (spectrum / structure)
Normalized Intensity
128.93
128.90
126.79
131.58
31.88
133.88
123.86
165.21
165.16
CHLOROFORM-d
N
N
O
3k
Chemical Shift (ppm)

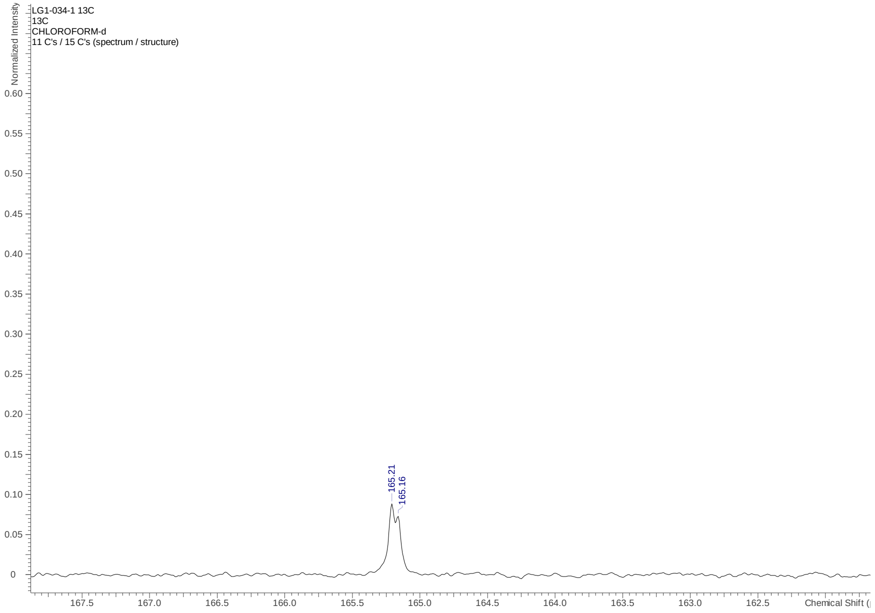
LG1-034-1 13C
13C
CHLOROFORM-d
11 C's / 15 C's (spectrum / structure)
Normalized Intensity
165.21
165.16
Chemical Shift (

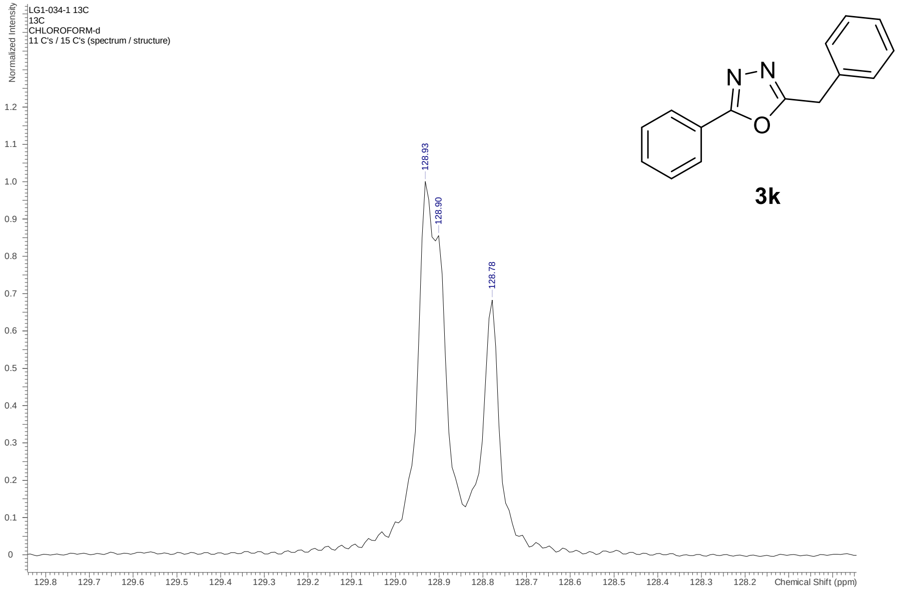
LG1-034-1 13C
13C
CHLOROFORM-d
11 C's / 15 C's (spectrum / structure)
Normalized Intensity
128.93
128.90
128.78
Chemical Shift (ppm)
N
N
O
3k

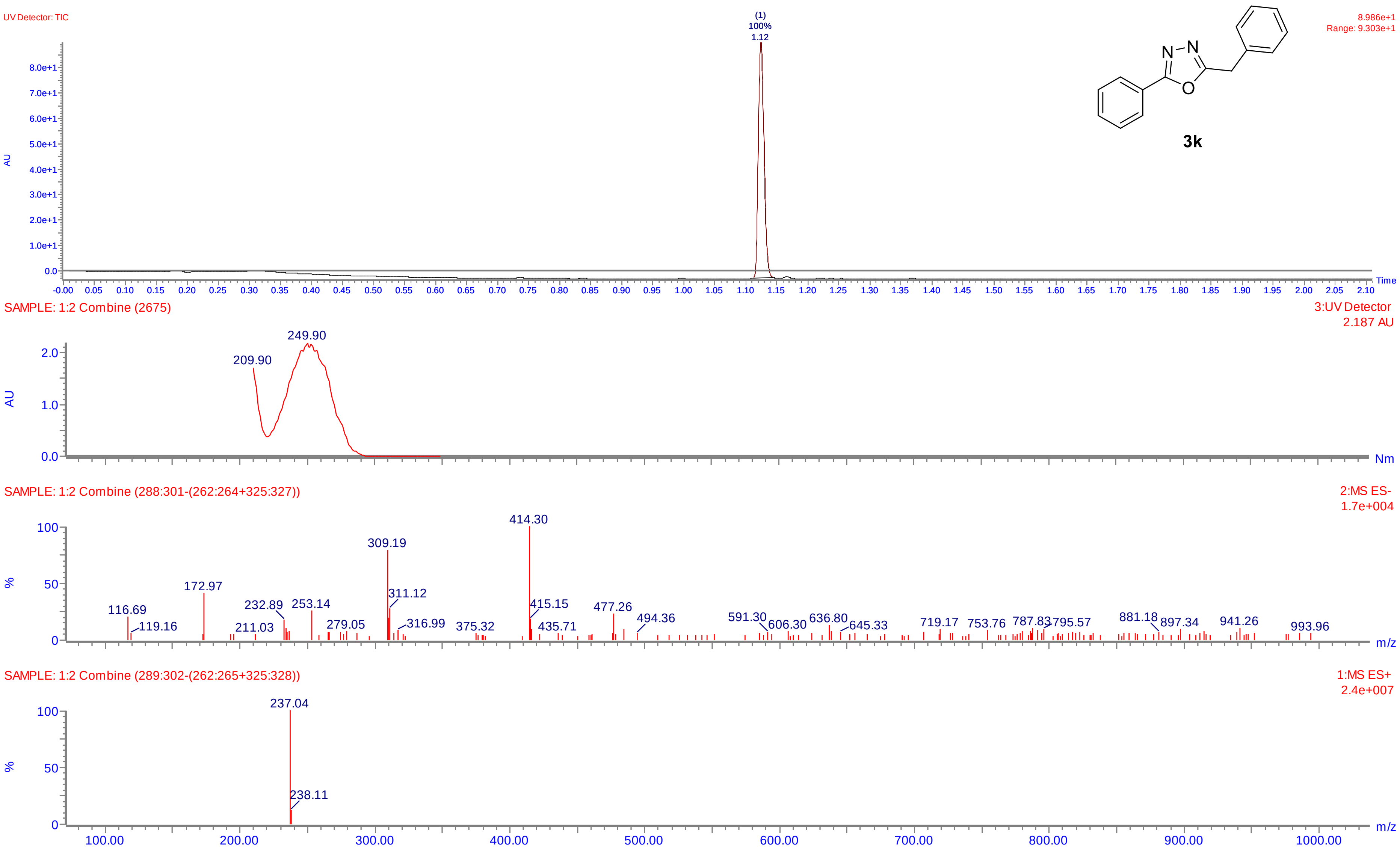

UV Detector: TIC
(1)
100%
1.12
8.986e+1
Range: 9.303e+1
AU
Time
3k
SAMPLE: 1:2 Combine (2675)
3:UV Detector
2.187 AU
249.90
209.90
Nm
SAMPLE: 1:2 Combine (288:301-(262:264+325:327))
2:MS ES-
1.7e+004
414.30
309.19
172.97
311.12
116.69
232.89
253.14
119.16
211.03
279.05
316.99
375.32
415.15
435.71
477.26
494.36
591.30
606.30
636.80
645.33
719.17
753.76
787.83
795.57
881.18
897.34
941.26
993.96
m/z
SAMPLE: 1:2 Combine (289:302-(262:265+325:328))
1:MS ES+
2.4e+007
237.04
238.11
m/z

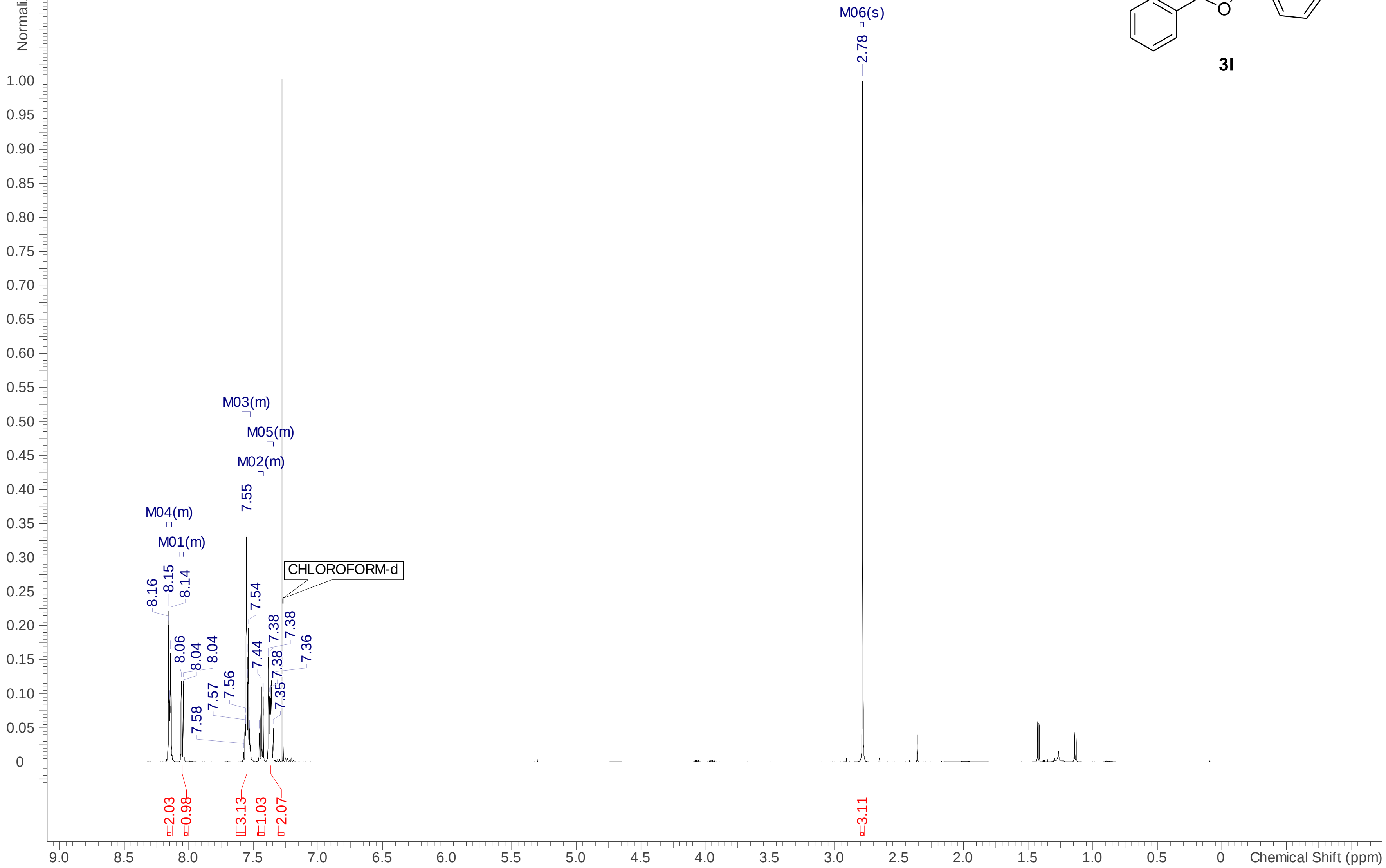
LG1-061-1 1H
1H
CHLOROFORM-d
12 H's / 12 H's (spectrum / structure)
Normalized Intensity
M06(s)
2.78
M03(m)
M05(m)
M02(m)
M04(m)
M01(m)
7.55
8.16
8.15
8.14
CHLOROFORM-d
7.54
8.06
8.04
8.04
7.44
7.38
7.38
7.38
7.36
7.35
7.58
7.57
7.56
2.03
0.98
3.13
1.03
2.07
3.11
Chemical Shift (ppm)

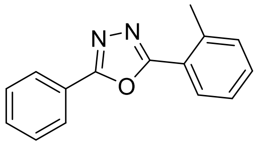

3l

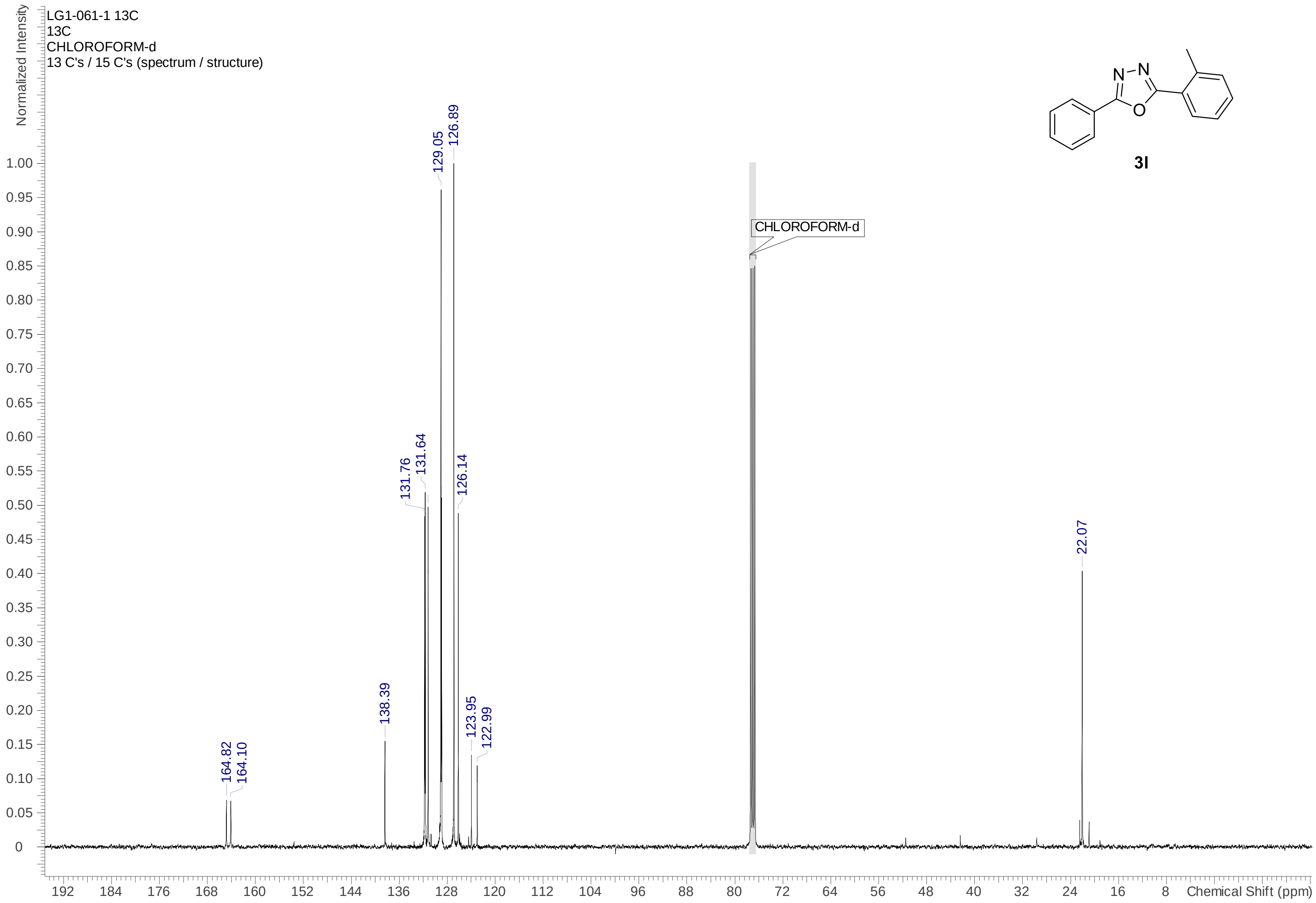
LG1-061-1 13C
13C
CHLOROFORM-d
13 C's / 15 C's (spectrum / structure)
Normalized Intensity
164.82
164.10
138.39
131.76
131.64
129.05
126.89
126.14
123.95
122.99
CHLOROFORM-d
22.07
N
N
O
Chemical Shift (ppm)

3l

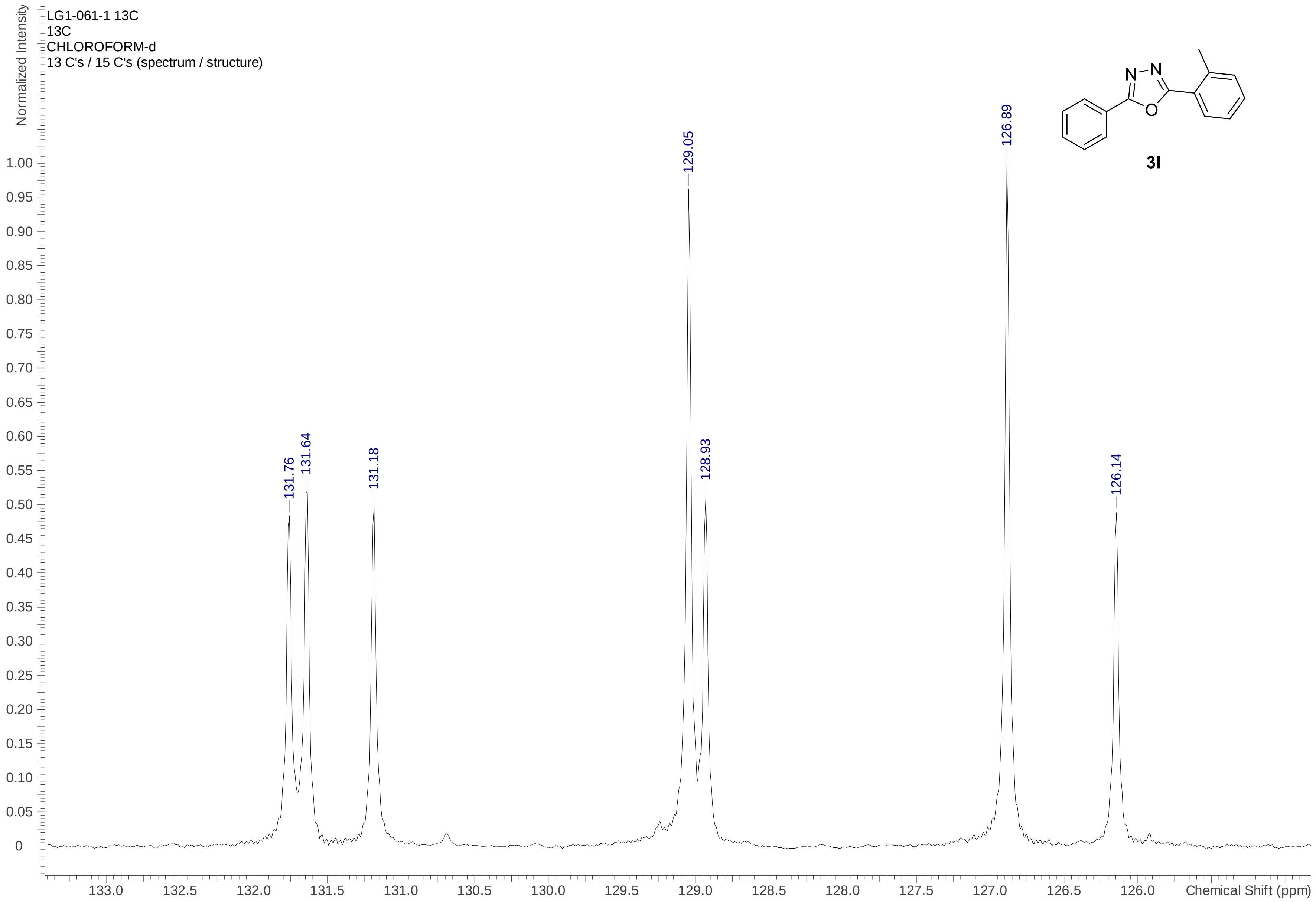
LG1-061-1 13C
13C
CHLOROFORM-d
13 C's / 15 C's (spectrum / structure)
Normalized Intensity
131.76
131.64
131.18
129.05
128.93
126.89
126.14
3l
Chemical Shift (ppm)

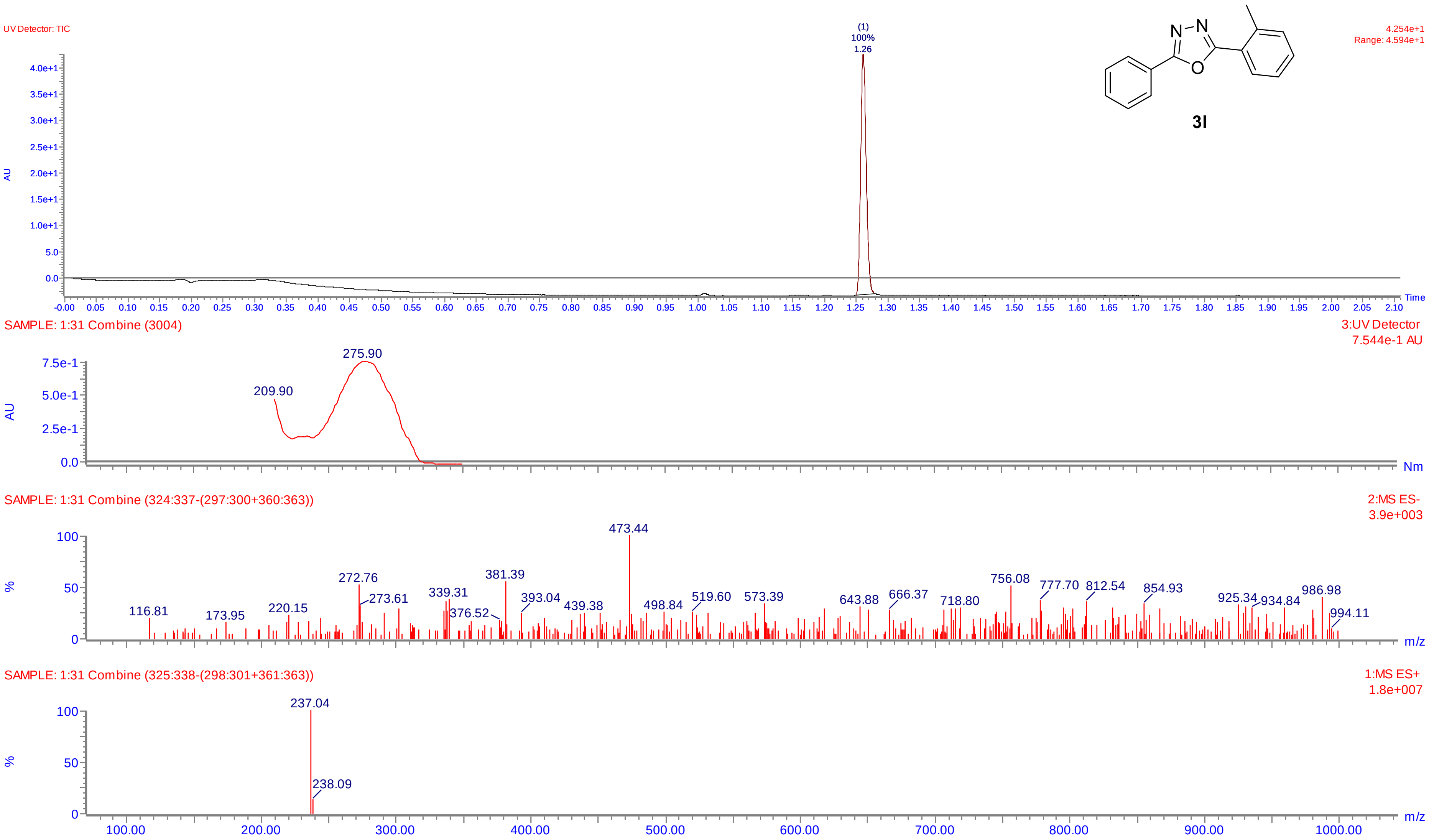
UV Detector: TIC
AU
(1)
100%
1.26
N
N
O
3l
4.254e+1
Range: 4.594e+1
Time
SAMPLE: 1:31 Combine (3004)
3:UV Detector
7.544e-1 AU
209.90
275.90
Nm
SAMPLE: 1:31 Combine (324:337-(297:300+360:363))
2:MS ES-
3.9e+003
473.44
272.76
273.61
339.31
376.52
381.39
393.04
439.38
498.84
519.60
573.39
643.88
666.37
718.80
756.08
777.70
812.54
854.93
925.34
934.84
986.98
994.11
116.81
173.95
220.15
m/z
SAMPLE: 1:31 Combine (325:338-(298:301+361:363))
1:MS ES+
1.8e+007
237.04
238.09
m/z
%

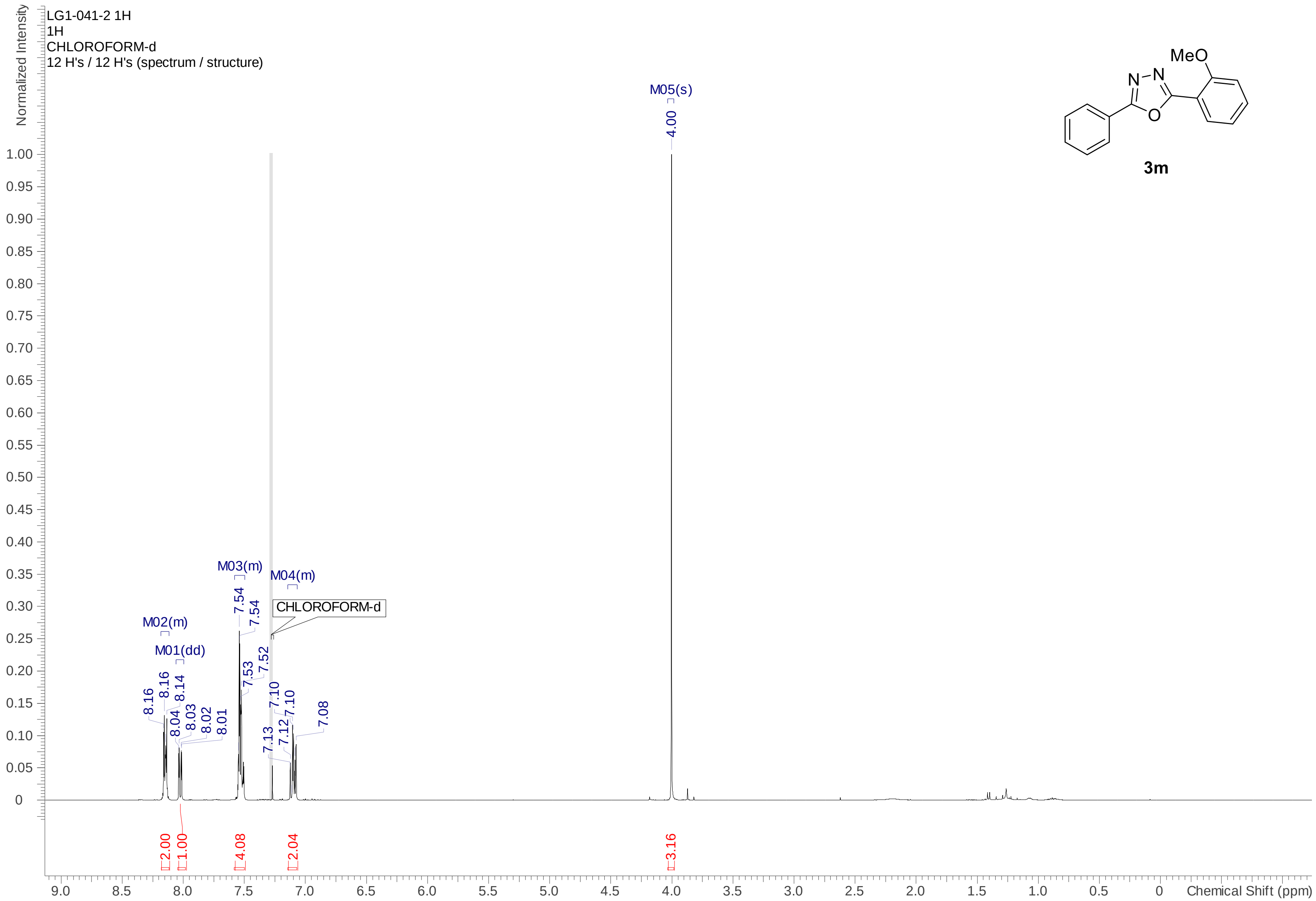

LG1-041-2 1H
1H
CHLOROFORM-d
12 H's / 12 H's (spectrum / structure)
Normalized Intensity
M05(s)
4.00
M03(m)
M04(m)
M02(m)
M01(dd)
CHLOROFORM-d
8.16
8.16
8.14
8.04
8.03
8.02
8.01
7.54
7.54
7.53
7.52
7.10
7.10
7.13
7.12
7.08
2.00
1.00
4.08
2.04
3.16
Chemical Shift (ppm)
MeO
N
N
O
3m

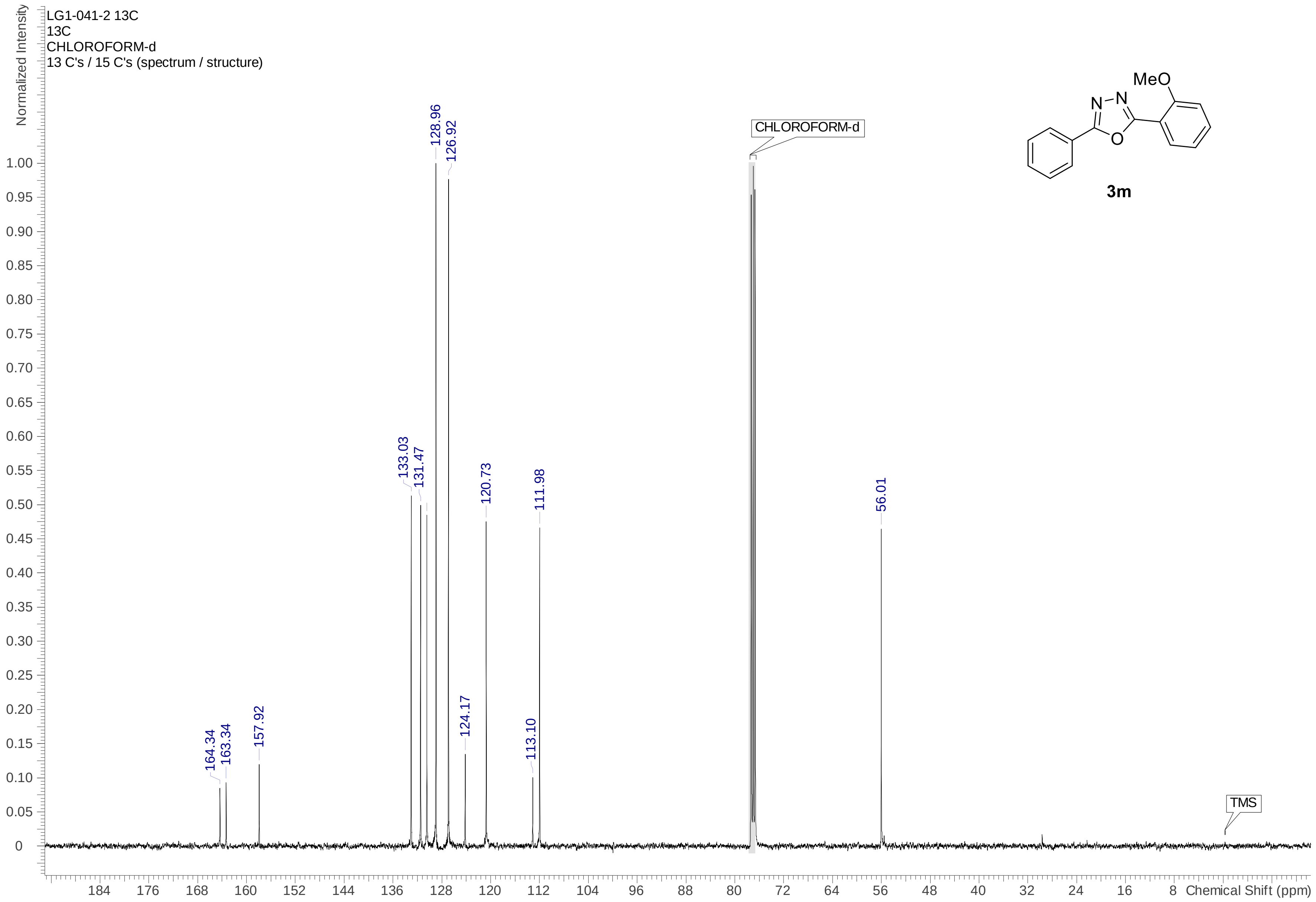
LG1-041-2 13C
13C
CHLOROFORM-d
13 C's / 15 C's (spectrum / structure)
Normalized Intensity
164.34
163.34
157.92
133.03
131.47
128.96
126.92
124.17
120.73
113.10
111.98
CHLOROFORM-d
56.01
TMS
MeO
N
N
O
3m
Chemical Shift (ppm)

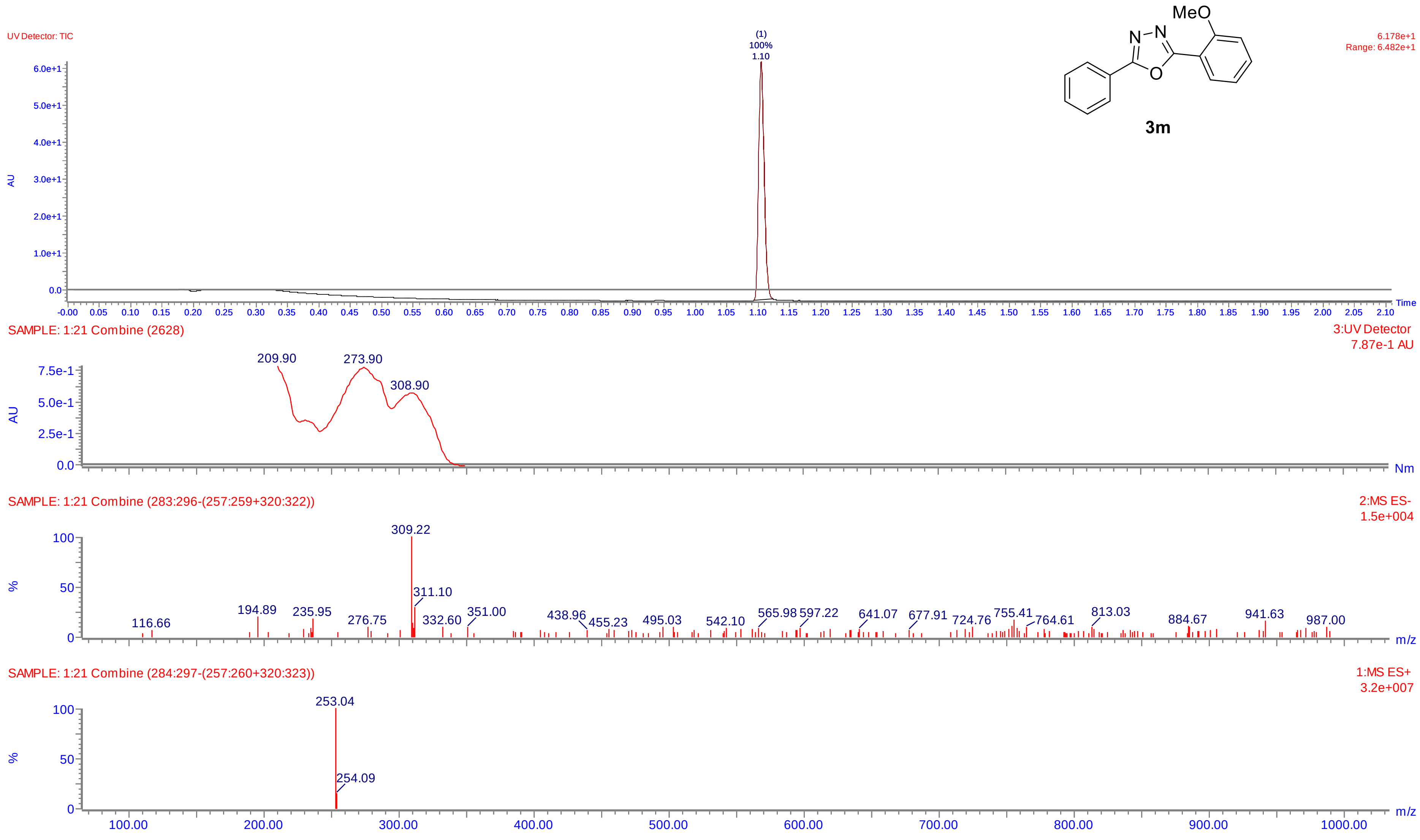
UV Detector: TIC
6.178e+1
Range: 6.482e+1
(1)
100%
1.10
AU
Time
MeO
N
N
O
3m
SAMPLE: 1:21 Combine (2628)
3:UV Detector
7.87e-1 AU
209.90
273.90
308.90
Nm
SAMPLE: 1:21 Combine (283:296-(257:259+320:322))
2:MS ES-
1.5e+004
309.22
311.10
116.66
194.89
235.95
276.75
332.60
351.00
438.96
455.23
495.03
542.10
565.98
597.22
641.07
677.91
724.76
755.41
764.61
813.03
884.67
941.63
987.00
m/z
SAMPLE: 1:21 Combine (284:297-(257:260+320:323))
1:MS ES+
3.2e+007
253.04
254.09
%
m/z

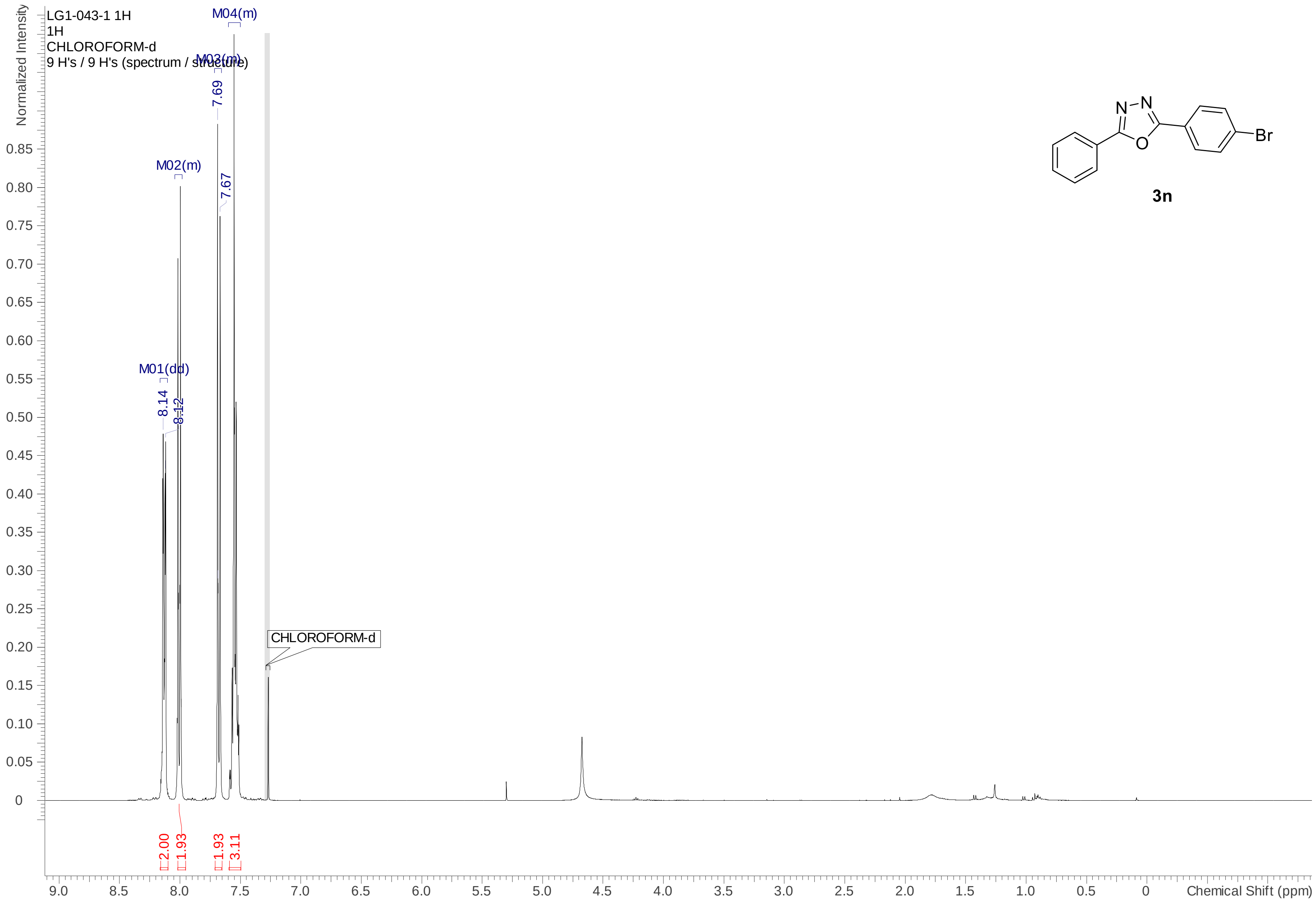
LG1-043-1 1H
1H
CHLOROFORM-d
9 H's / 9 H's (spectrum / structure)
Normalized Intensity
M01(dd)
8.14
8.12
M02(m)
M03(m)
7.69
7.67
M04(m)
CHLOROFORM-d
2.00
1.93
1.93
3.11
Chemical Shift (ppm)
N
N
O
Br

3n

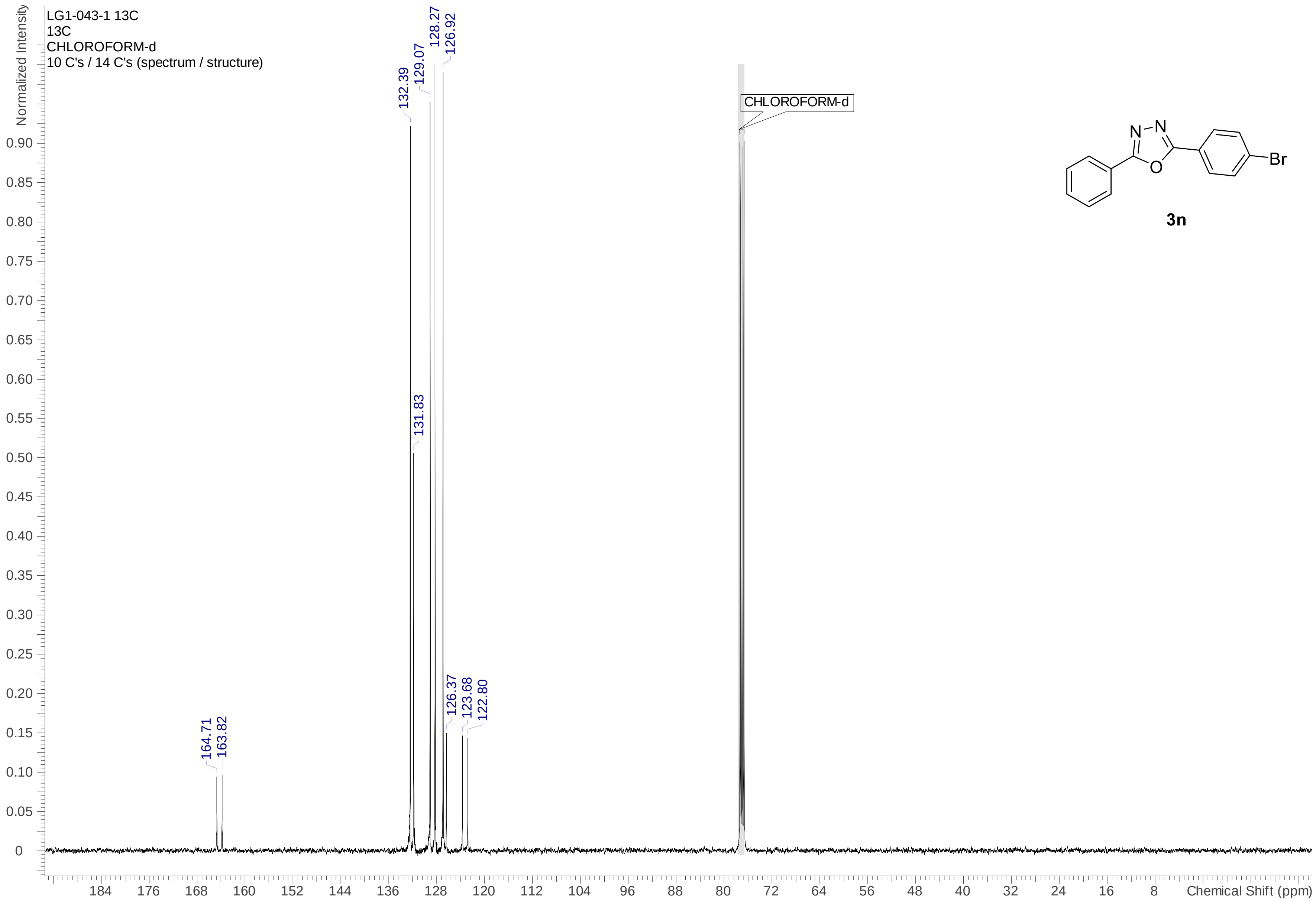
LG1-043-1 13C
13C
CHLOROFORM-d
10 C's / 14 C's (spectrum / structure)
Normalized Intensity
164.71
163.82
132.39
131.83
129.07
128.27
126.92
126.37
123.68
122.80
CHLOROFORM-d
Chemical Shift (ppm)
N
N
O
Br

3n

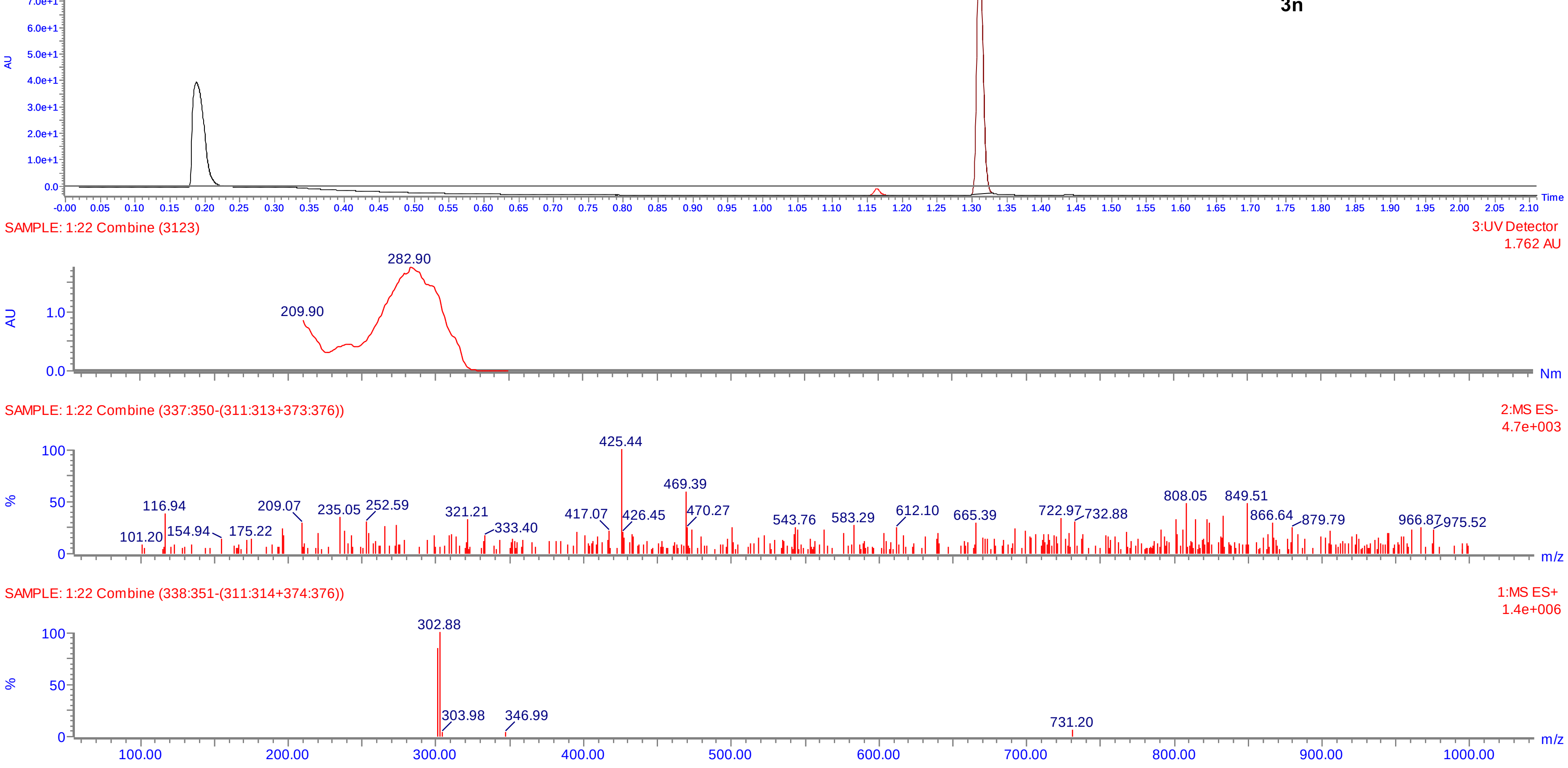
UV Detector: TIC
9.67e+1
Range: 1.002e+2
(2)
98%
1.31
AU
Time
SAMPLE: 1:22 Combine (3123)
3:UV Detector
1.762 AU
282.90
209.90
AU
Nm
SAMPLE: 1:22 Combine (337:350-(311:313+373:376))
2:MS ES-
4.7e+003
425.44
469.39
470.27
426.45
417.07
333.40
321.21
252.59
235.05
209.07
175.22
154.94
116.94
101.20
543.76
583.29
612.10
665.39
722.97
732.88
808.05
849.51
866.64
879.79
966.87
975.52
%
m/z
SAMPLE: 1:22 Combine (338:351-(311:314+374:376))
1:MS ES+
1.4e+006
302.88
303.98
346.99
731.20
%
m/z

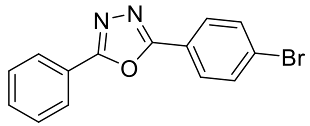

3n

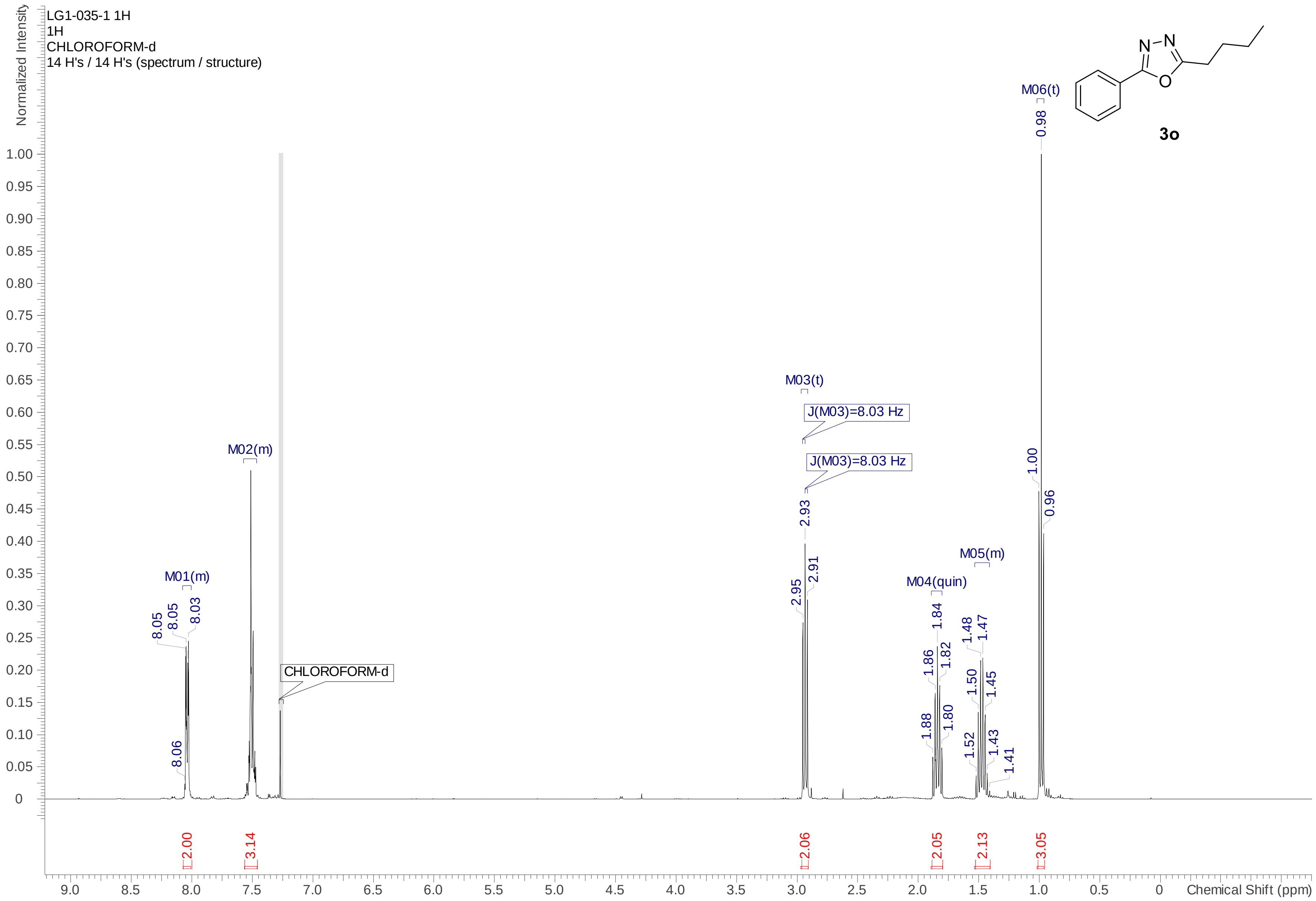
LG1-035-1 1H
1H
CHLOROFORM-d
14 H's / 14 H's (spectrum / structure)
Normalized Intensity
M01(m)
8.05
8.05
8.03
8.06
M02(m)
CHLOROFORM-d
M03(t)
J(M03)=8.03 Hz
J(M03)=8.03 Hz
2.95
2.93
2.91
M04(quin)
1.88
1.86
1.84
1.82
1.80
M05(m)
1.52
1.50
1.48
1.47
1.45
1.43
1.41
M06(t)
0.98
1.00
0.96
2.00
3.14
2.06
2.05
2.13
3.05
Chemical Shift (ppm)

3o

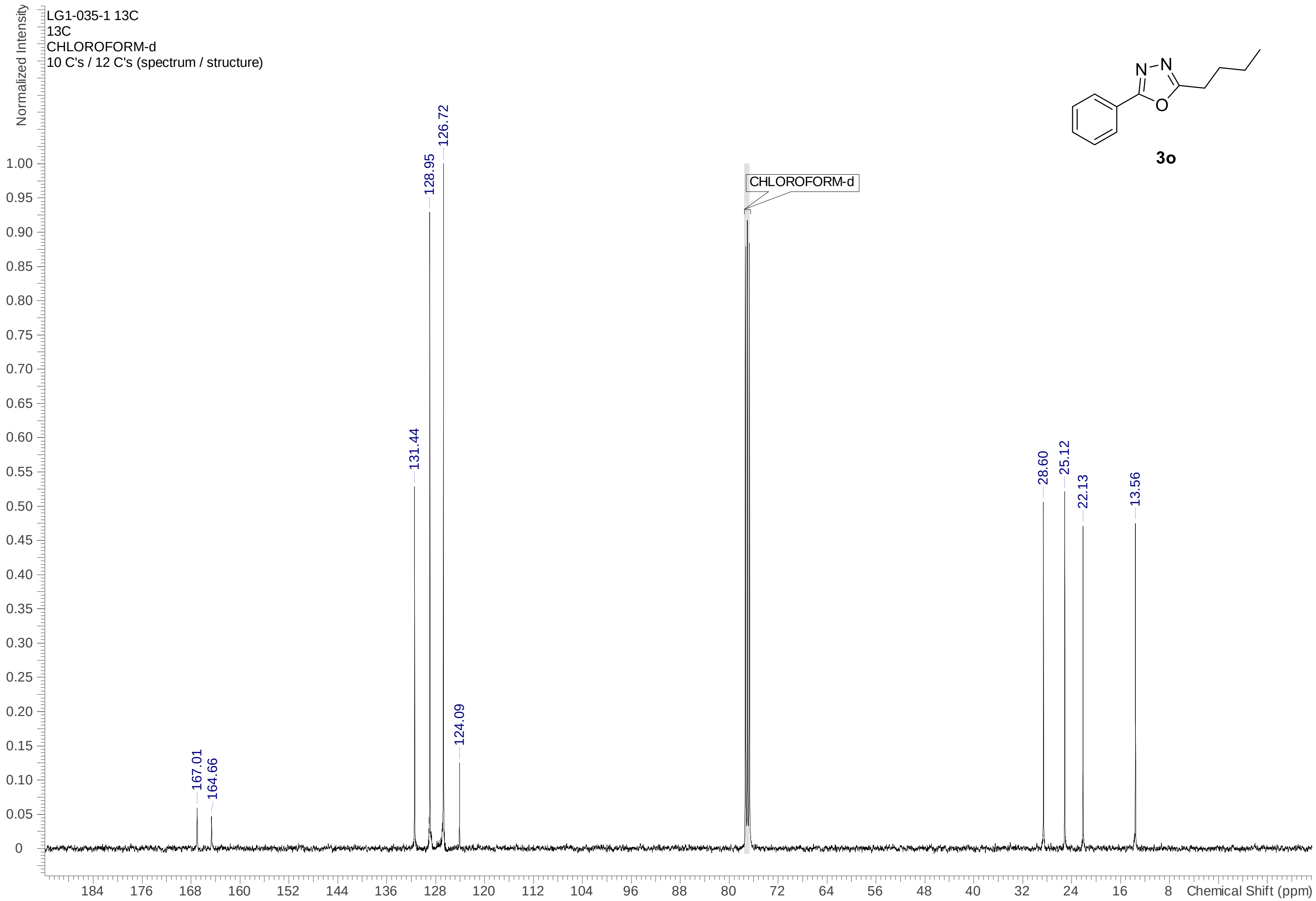
LG1-035-1 13C
13C
CHLOROFORM-d
10 C's / 12 C's (spectrum / structure)
Normalized Intensity
167.01
164.66
131.44
128.95
126.72
124.09
CHLOROFORM-d
28.60
25.12
22.13
13.56
Chemical Shift (ppm)
N
N
O
3o

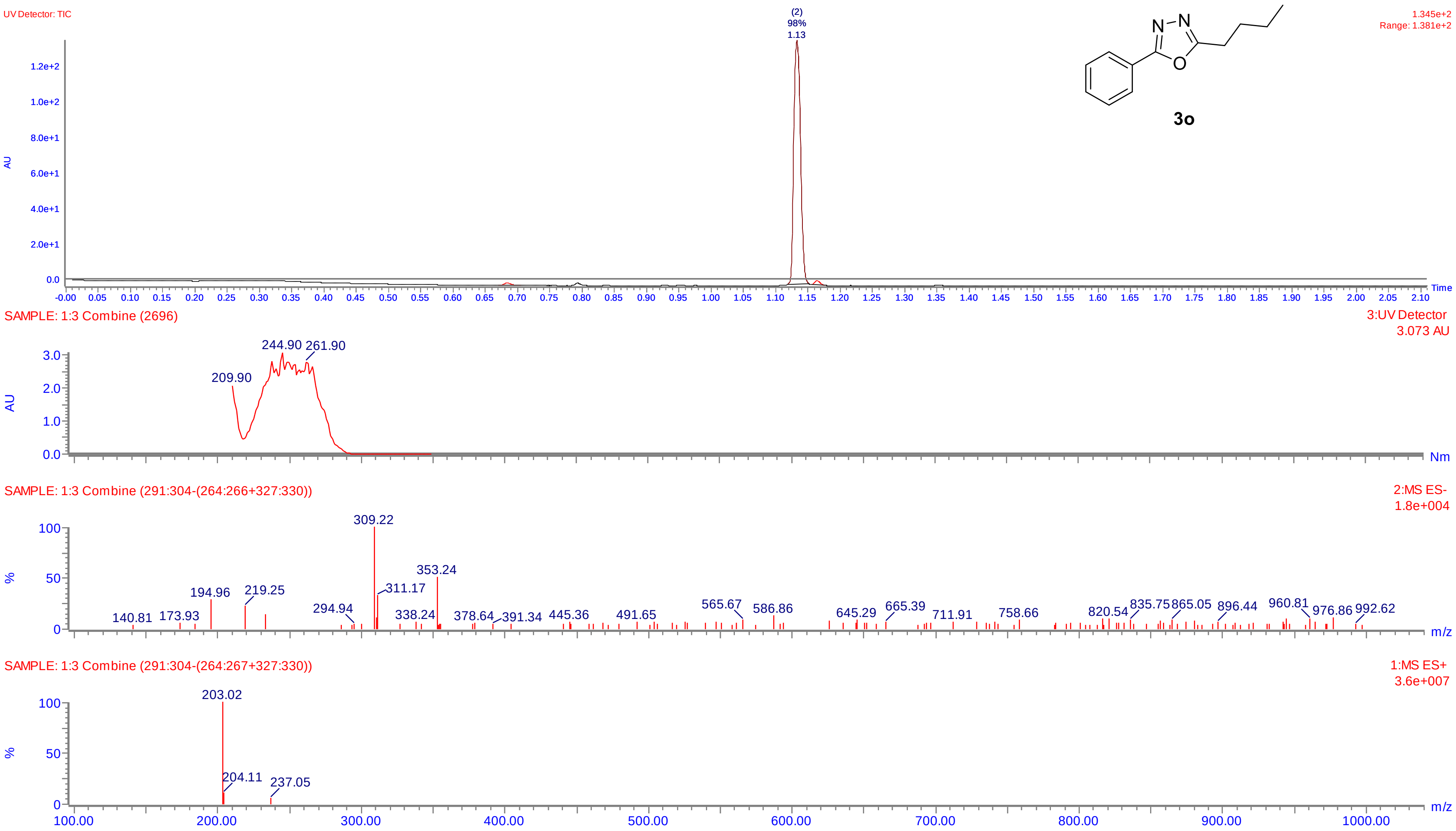

UV Detector: TIC
(2)
98%
1.13
1.345e+2
Range: 1.381e+2
AU
Time
SAMPLE: 1:3 Combine (2696)
3:UV Detector
3.073 AU
209.90
244.90 261.90
Nm
SAMPLE: 1:3 Combine (291:304-(264:266+327:330))
2:MS ES-
1.8e+004
309.22
353.24
311.17
194.96
219.25
140.81
173.93
294.94
338.24
378.64
391.34
445.36
491.65
565.67
586.86
645.29
665.39
711.91
758.66
820.54
835.75
865.05
896.44
960.81
976.86
992.62
m/z
SAMPLE: 1:3 Combine (291:304-(264:267+327:330))
1:MS ES+
3.6e+007
203.02
204.11
237.05
m/z

3o

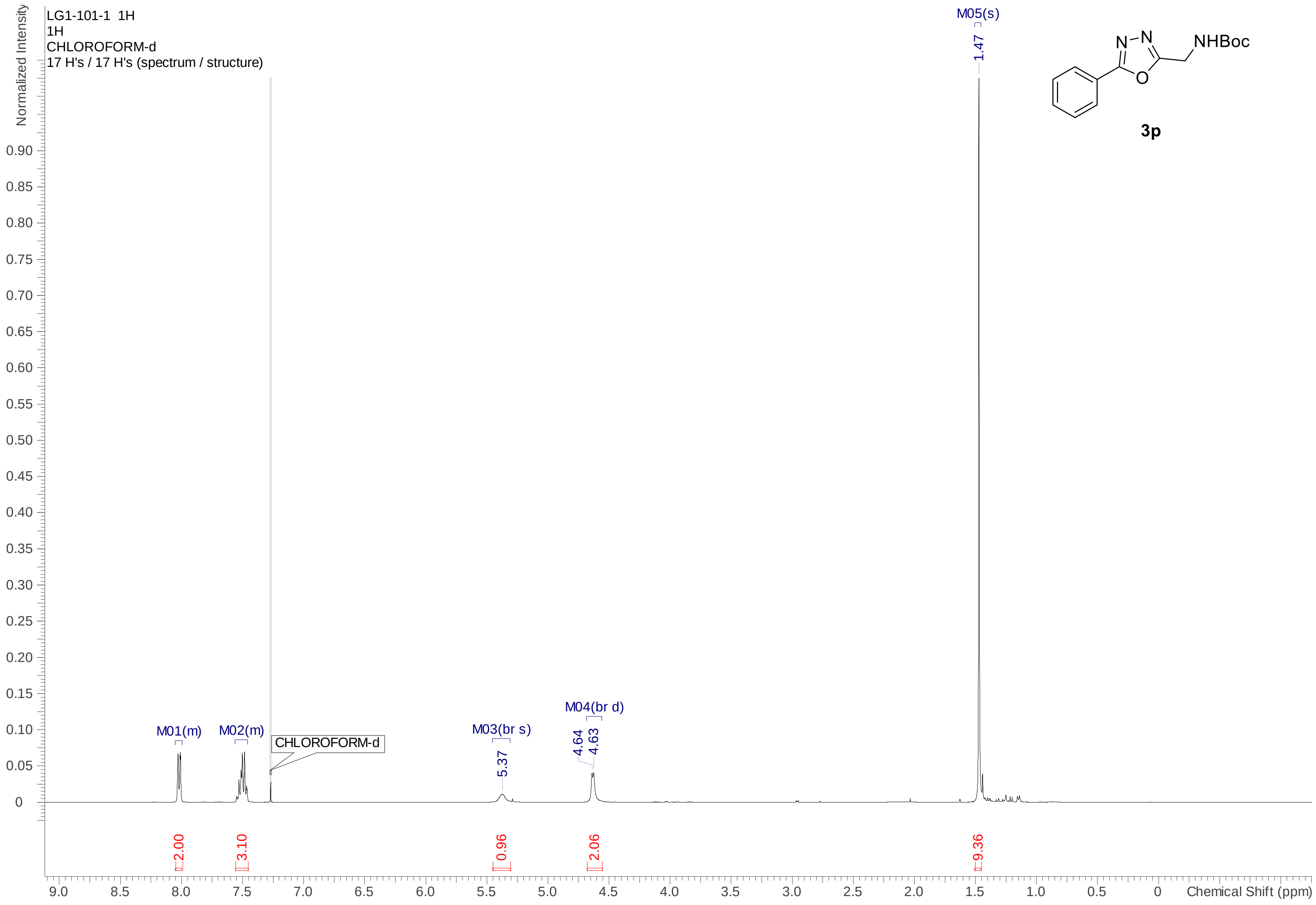
LG1-101-1 1H
1H
CHLOROFORM-d
17 H's / 17 H's (spectrum / structure)
Normalized Intensity
M01(m)
M02(m)
CHLOROFORM-d
M03(br s)
5.37
M04(br d)
4.64
4.63
M05(s)
1.47
2.00
3.10
0.96
2.06
9.36
N
N
O
NHBoc
3p
Chemical Shift (ppm)

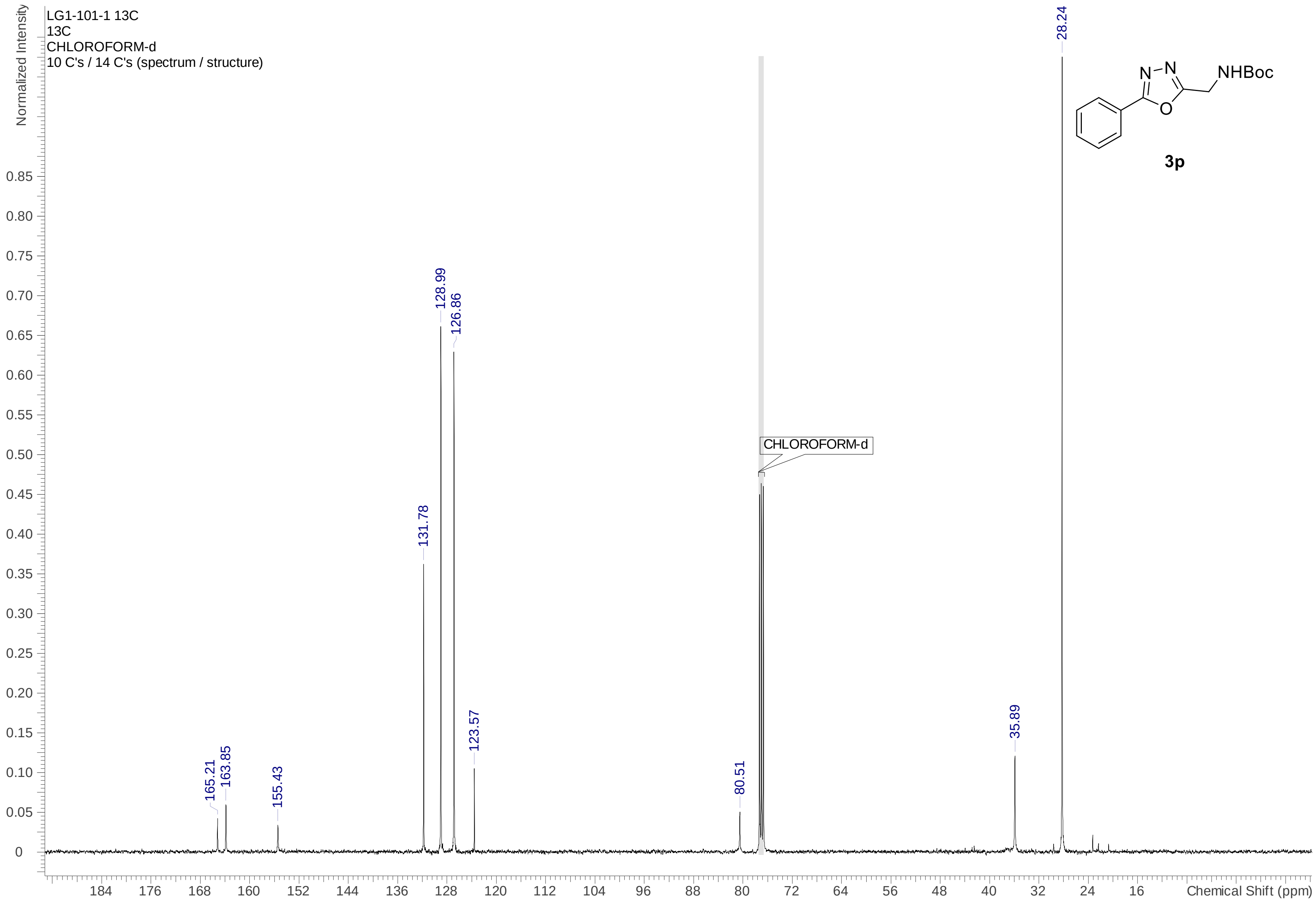

LG1-101-1 13C
13C
CHLOROFORM-d
10 C's / 14 C's (spectrum / structure)
Normalized Intensity
0.85
0.80
0.75
0.70
0.65
0.60
0.55
0.50
0.45
0.40
0.35
0.30
0.25
0.20
0.15
0.10
0.05
0
165.21
163.85
155.43
131.78
128.99
126.86
123.57
80.51
CHLOROFORM-d
35.89
28.24
N
N
NHBoc
O
3p
184
176
168
160
152
144
136
128
120
112
104
96
88
80
72
64
56
48
40
32
24
16
Chemical Shift (ppm)

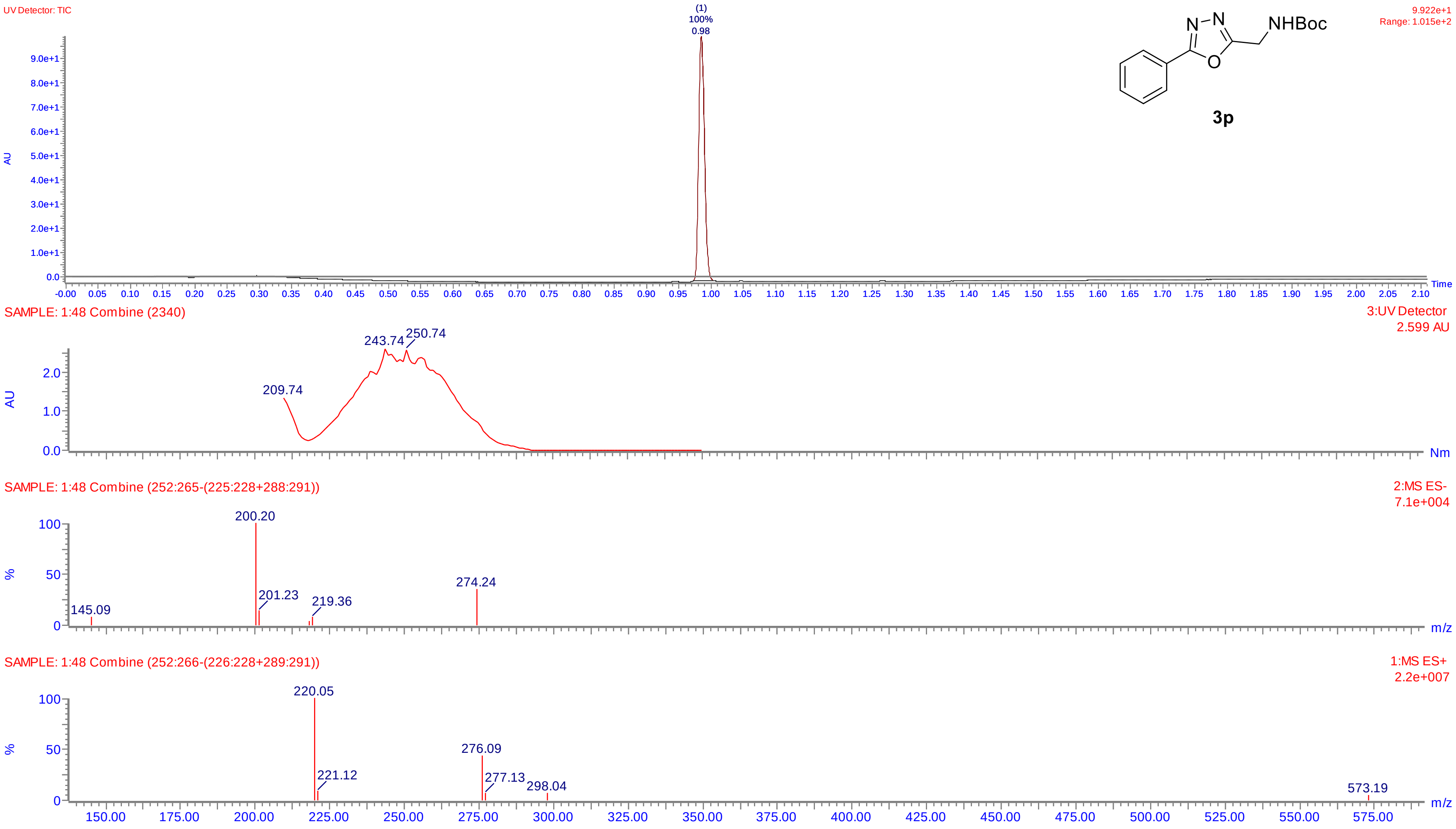
UV Detector: TIC
(1)
100%
0.98
N–N
NHBoc
O
3p
9.922e+1
Range: 1.015e+2
AU
Time
SAMPLE: 1:48 Combine (2340)
3:UV Detector
2.599 AU
243.74
250.74
209.74
Nm
SAMPLE: 1:48 Combine (252:265-(225:228+288:291))
2:MS ES-
7.1e+004
200.20
201.23
219.36
274.24
145.09
%
m/z
SAMPLE: 1:48 Combine (252:266-(226:228+289:291))
1:MS ES+
2.2e+007
220.05
221.12
276.09
277.13
298.04
573.19
%
m/z

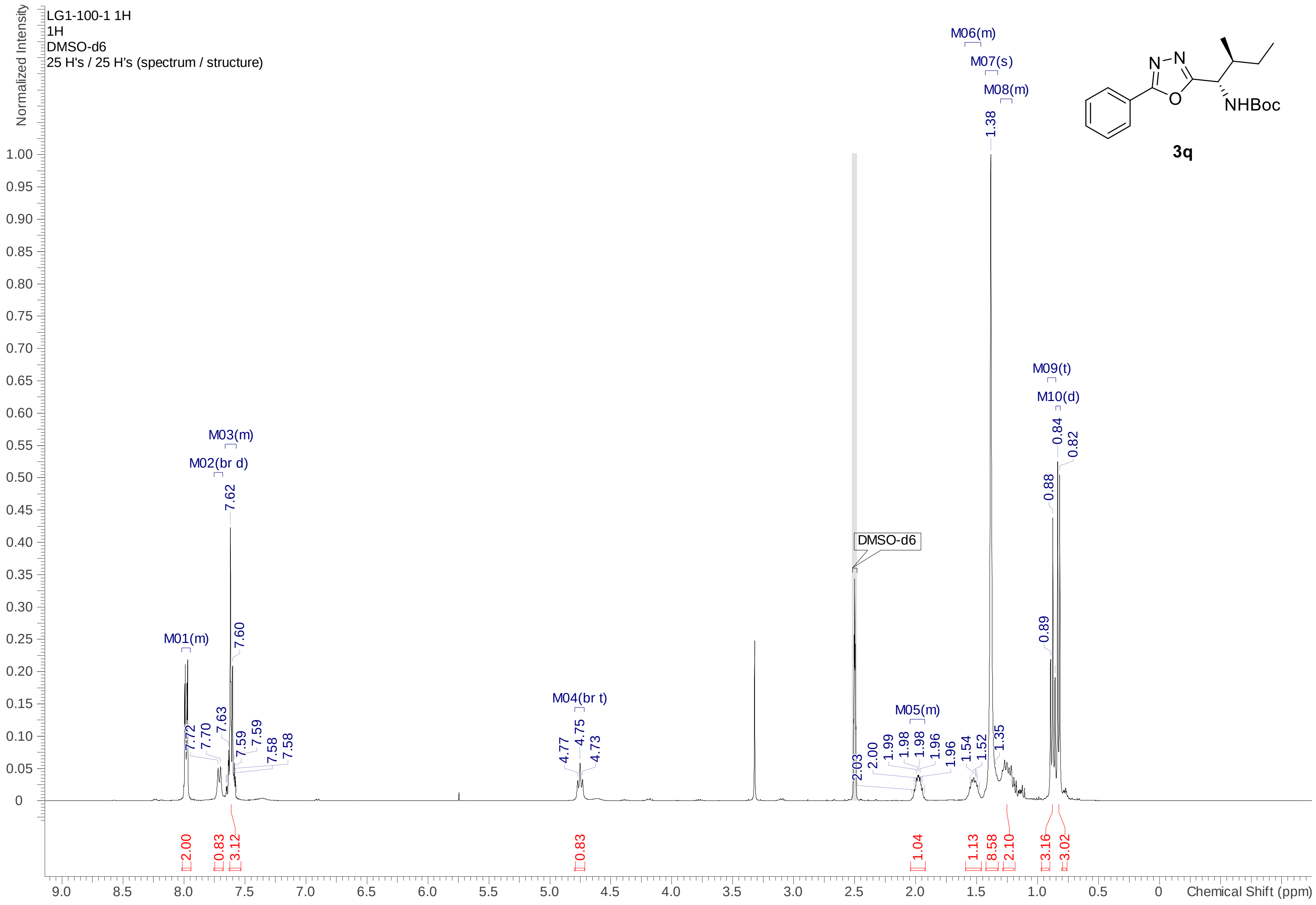

LG1-100-1 1H
1H
DMSO-d6
25 H's / 25 H's (spectrum / structure)
Normalized Intensity
M01(m)
M02(br d)
M03(m)
M04(br t)
M05(m)
M06(m)
M07(s)
M08(m)
M09(t)
M10(d)
DMSO-d6
NHBoc
3q
Chemical Shift (ppm)

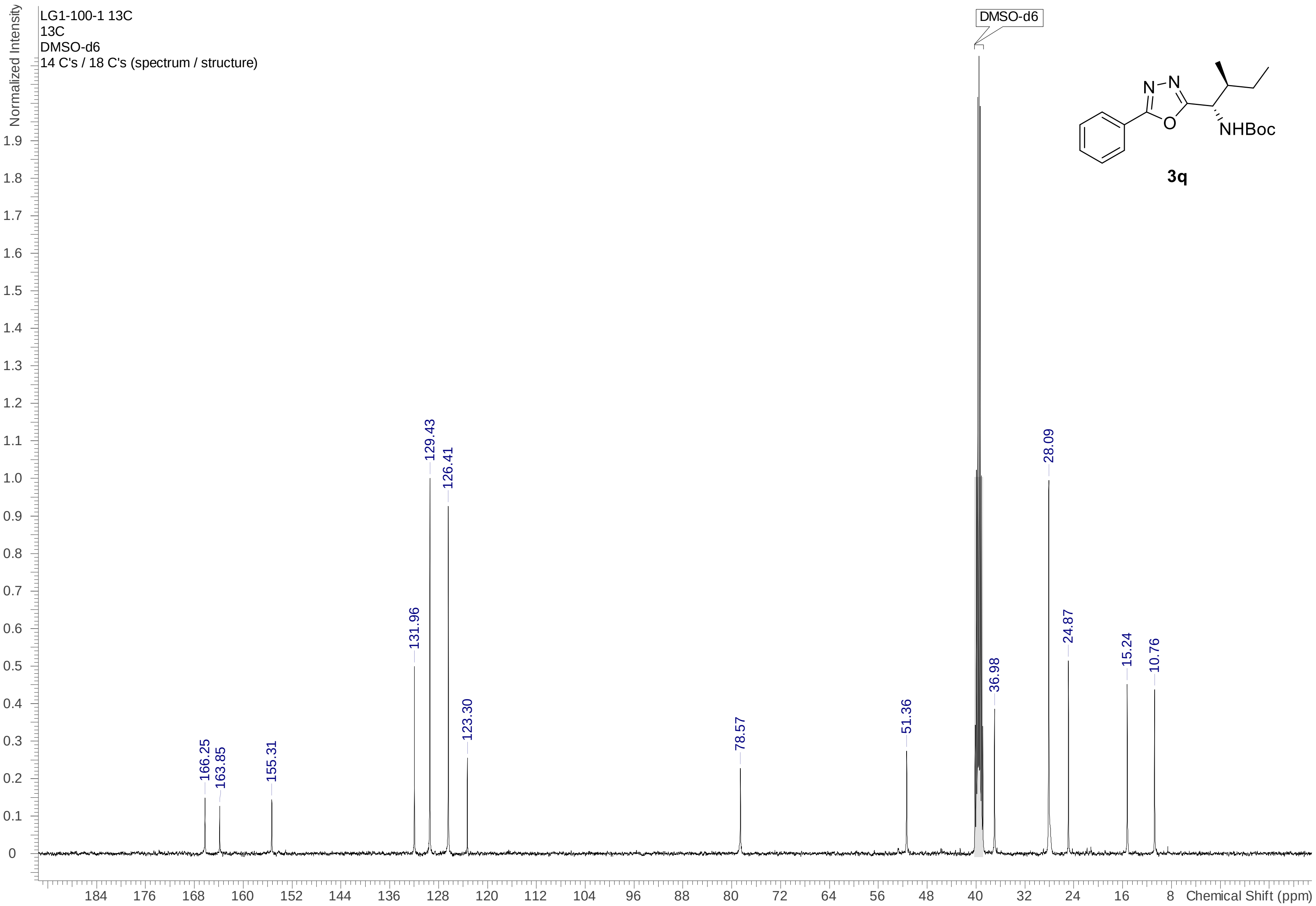
LG1-100-1 13C
13C
DMSO-d6
14 C's / 18 C's (spectrum / structure)
DMSO-d6
Normalized Intensity
Chemical Shift (ppm)
166.25
163.85
155.31
131.96
129.43
126.41
123.30
78.57
51.36
36.98
28.09
24.87
15.24
10.76
N
N
O
NHBoc
3q

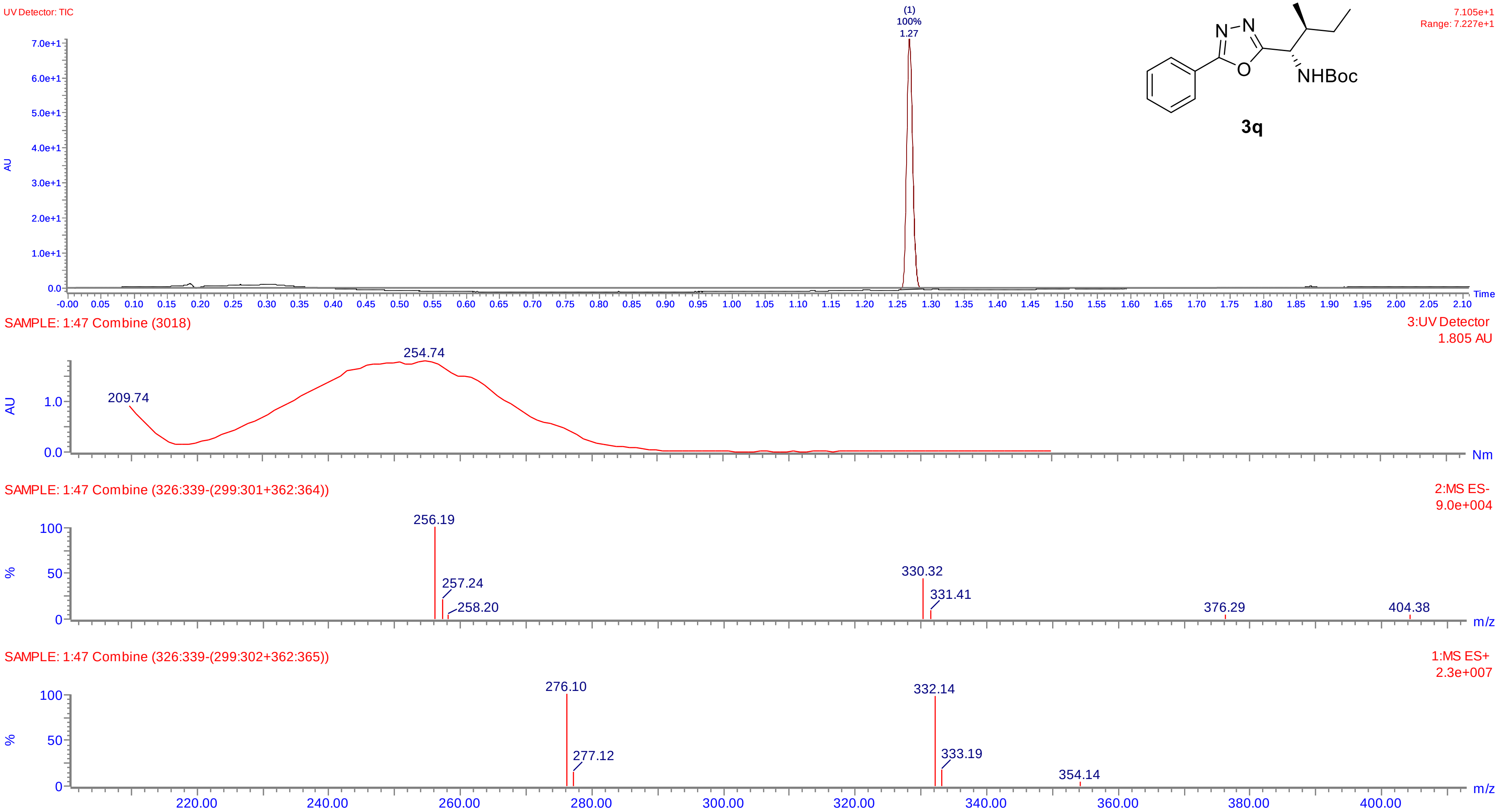
UV Detector: TIC
(1)
100%
1.27
7.105e+1
Range: 7.227e+1
AU
Time
N
N
O
NHBoc
3q
SAMPLE: 1:47 Combine (3018)
3:UV Detector
1.805 AU
254.74
209.74
AU
Nm
SAMPLE: 1:47 Combine (326:339-(299:301+362:364))
2:MS ES-
9.0e+004
256.19
257.24
258.20
330.32
331.41
376.29
404.38
%
m/z
SAMPLE: 1:47 Combine (326:339-(299:302+362:365))
1:MS ES+
2.3e+007
276.10
277.12
332.14
333.19
354.14
%
m/z

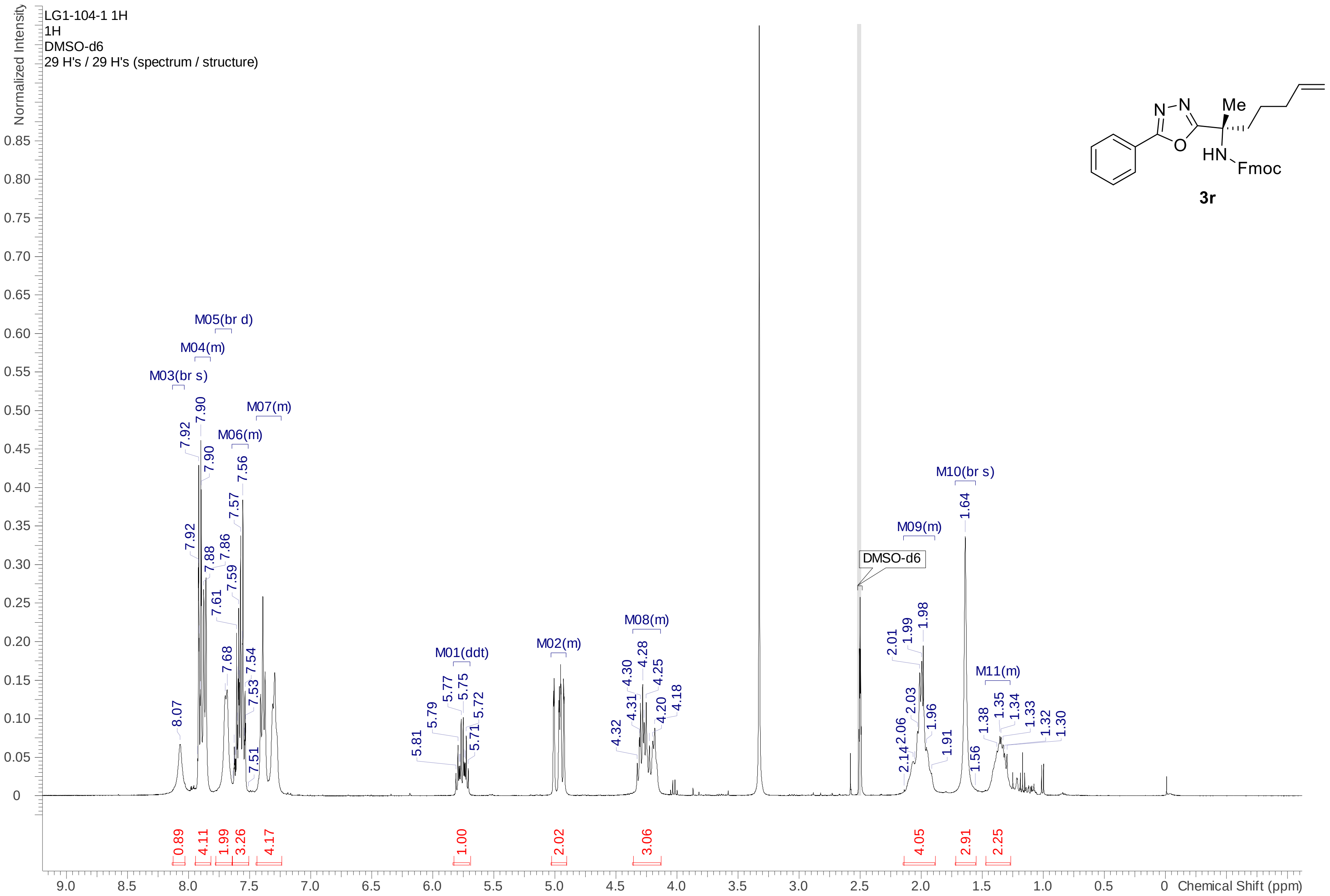

LG1-104-1 1H
1H
DMSO-d6
29 H's / 29 H's (spectrum / structure)
Normalized Intensity
Chemical Shift (ppm)
M01(ddt)
M02(m)
M03(br s)
M04(m)
M05(br d)
M06(m)
M07(m)
M08(m)
M09(m)
M10(br s)
M11(m)
DMSO-d6
0.89
4.11
1.99
3.26
4.17
1.00
2.02
3.06
4.05
2.91
2.25
Me
HN
Fmoc
3r

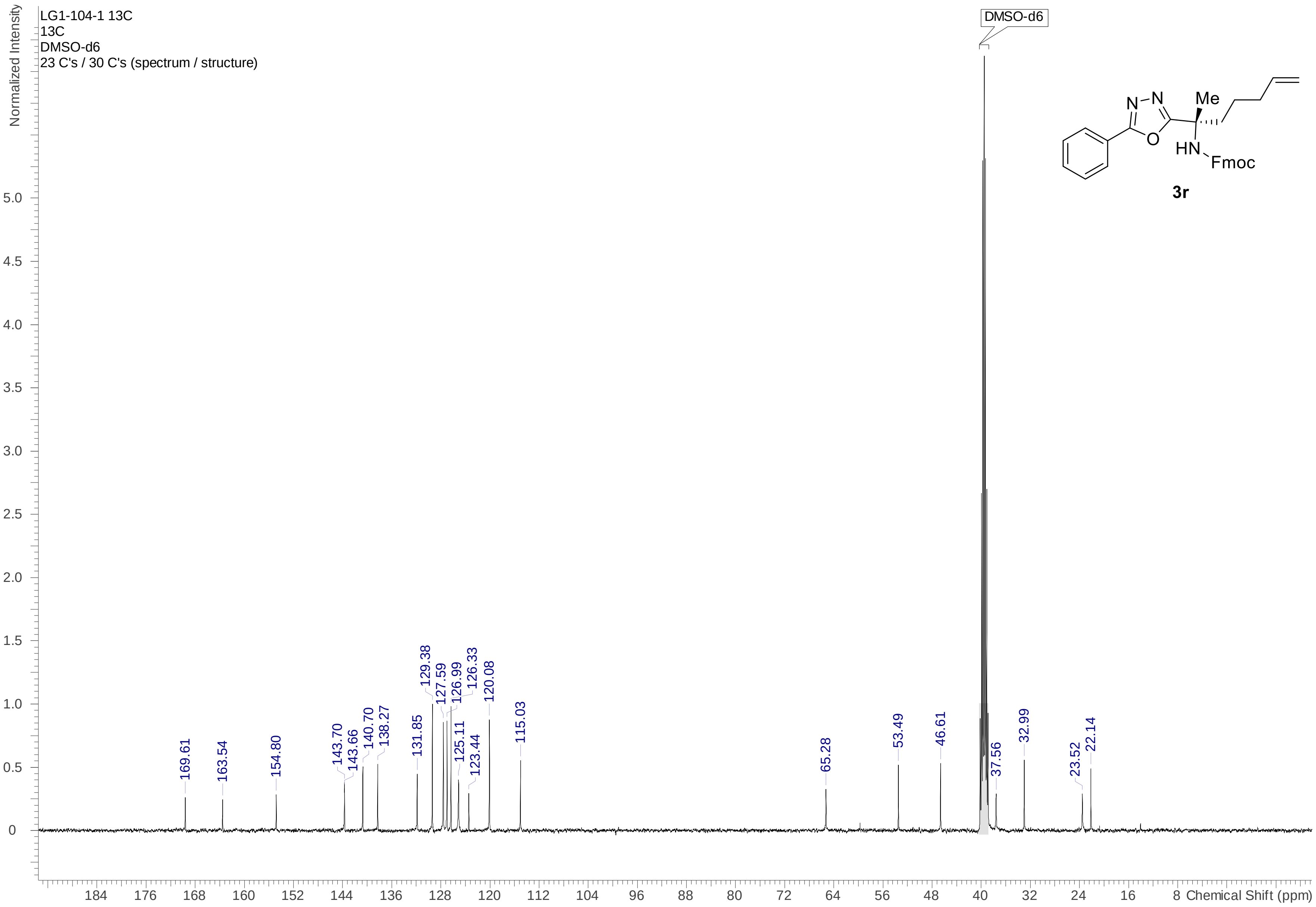
LG1-104-1 13C
13C
DMSO-d6
23 C's / 30 C's (spectrum / structure)
DMSO-d6
Normalized Intensity
Chemical Shift (ppm)
169.61
163.54
154.80
143.70
143.66
140.70
138.27
131.85
129.38
127.59
126.99
126.33
125.11
123.44
120.08
115.03
65.28
53.49
46.61
37.56
32.99
23.52
22.14
N
N
O
Me
HN
Fmoc
3r

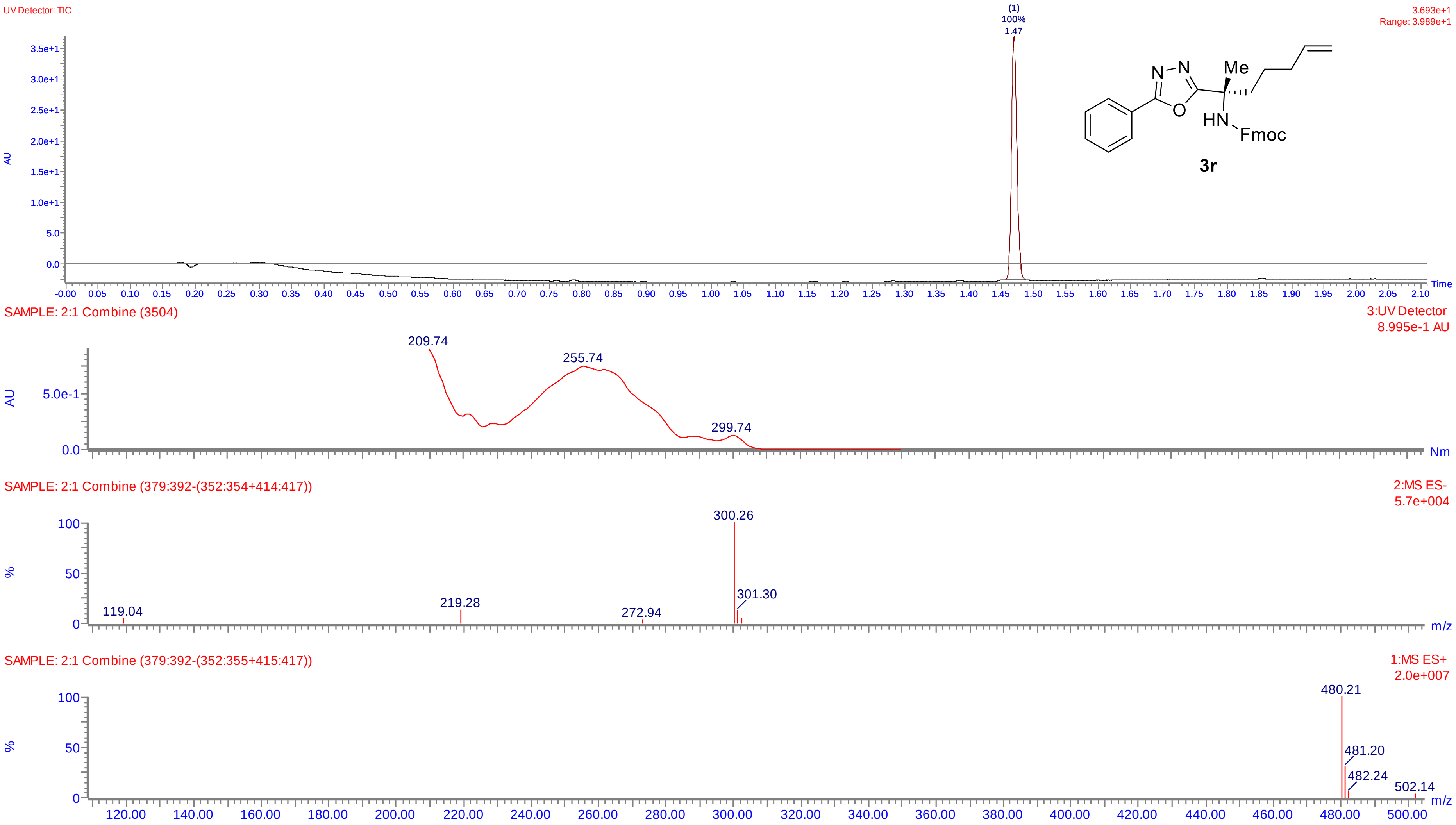
UV Detector: TIC
(1)
100%
1.47
3.693e+1
Range: 3.989e+1
AU
Time
Me
HN
Fmoc
3r
SAMPLE: 2:1 Combine (3504)
3:UV Detector
8.995e-1 AU
209.74
255.74
299.74
Nm
SAMPLE: 2:1 Combine (379:392-(352:354+414:417))
2:MS ES-
5.7e+004
300.26
301.30
119.04
219.28
272.94
m/z
SAMPLE: 2:1 Combine (379:392-(352:355+415:417))
1:MS ES+
2.0e+007
480.21
481.20
482.24
502.14
m/z

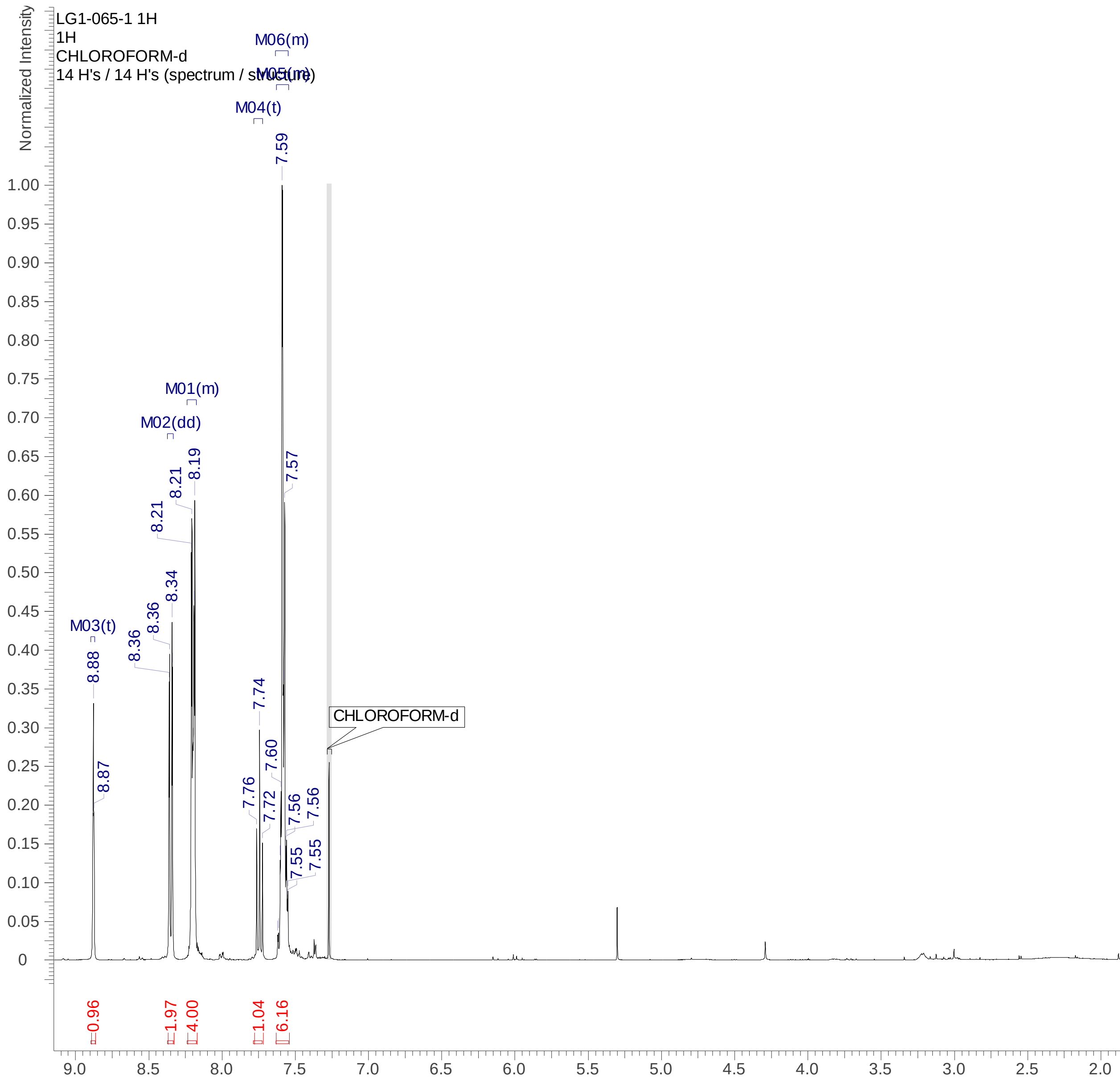
LG1-065-1 1H
1H
CHLOROFORM-d
14 H's / 14 H's (spectrum / structure)
Normalized Intensity
M01(m)
M02(dd)
M03(t)
M04(t)
M05(m)
M06(m)
8.88
8.87
8.36
8.36
8.34
8.21
8.21
8.19
7.76
7.74
7.72
7.60
7.59
7.57
7.56
7.56
7.55
7.55
CHLOROFORM-d
0.96
1.97
4.00
1.04
6.16
Chemical Shift (ppm)

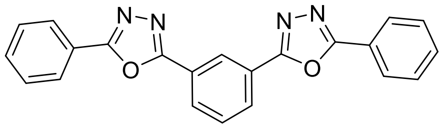

3s

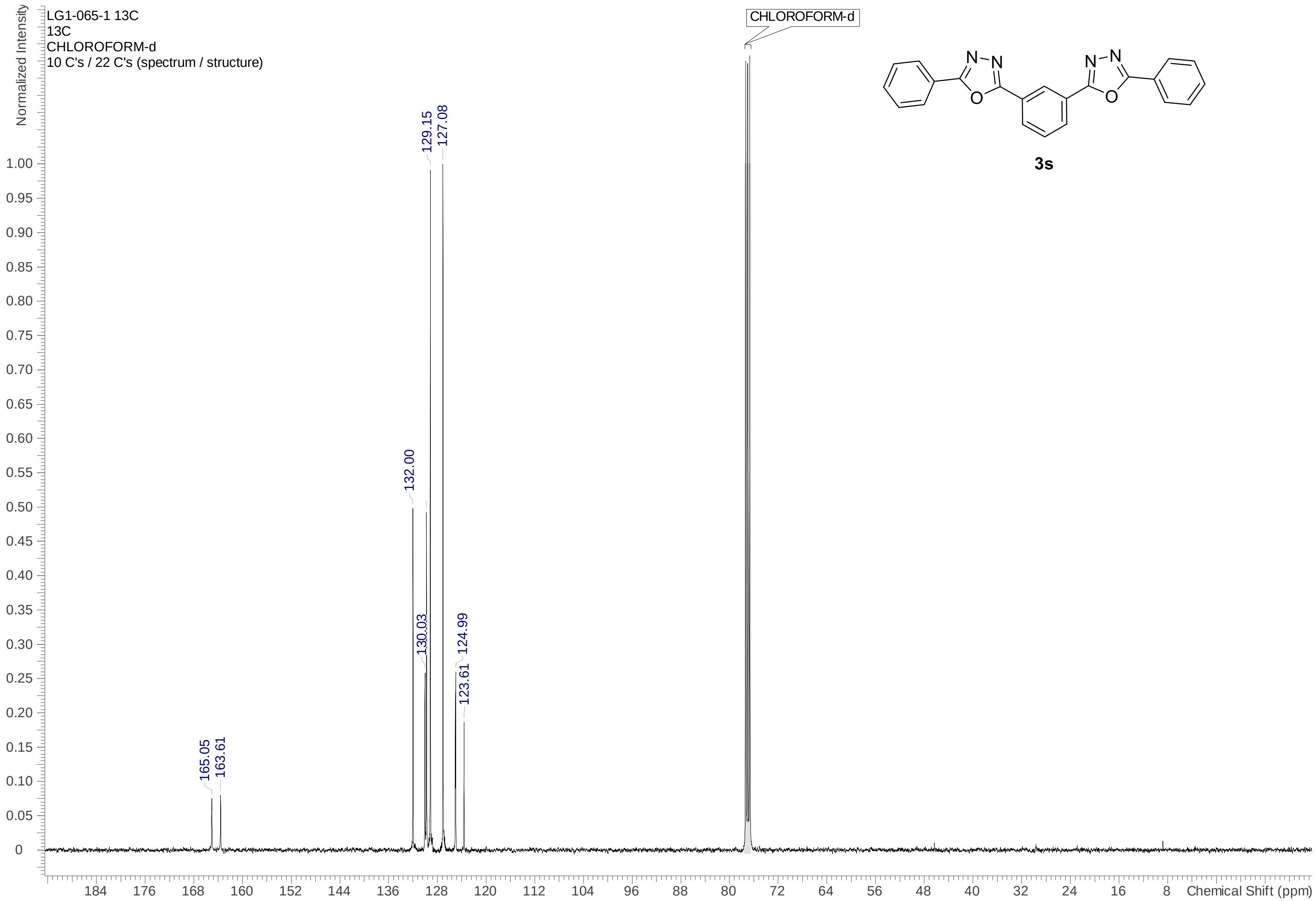
LG1-065-1 13C
13C
CHLOROFORM-d
10 C's / 22 C's (spectrum / structure)
Normalized Intensity
CHLOROFORM-d
165.05
163.61
132.00
130.03
129.15
127.08
124.99
123.61
3s
Chemical Shift (ppm)

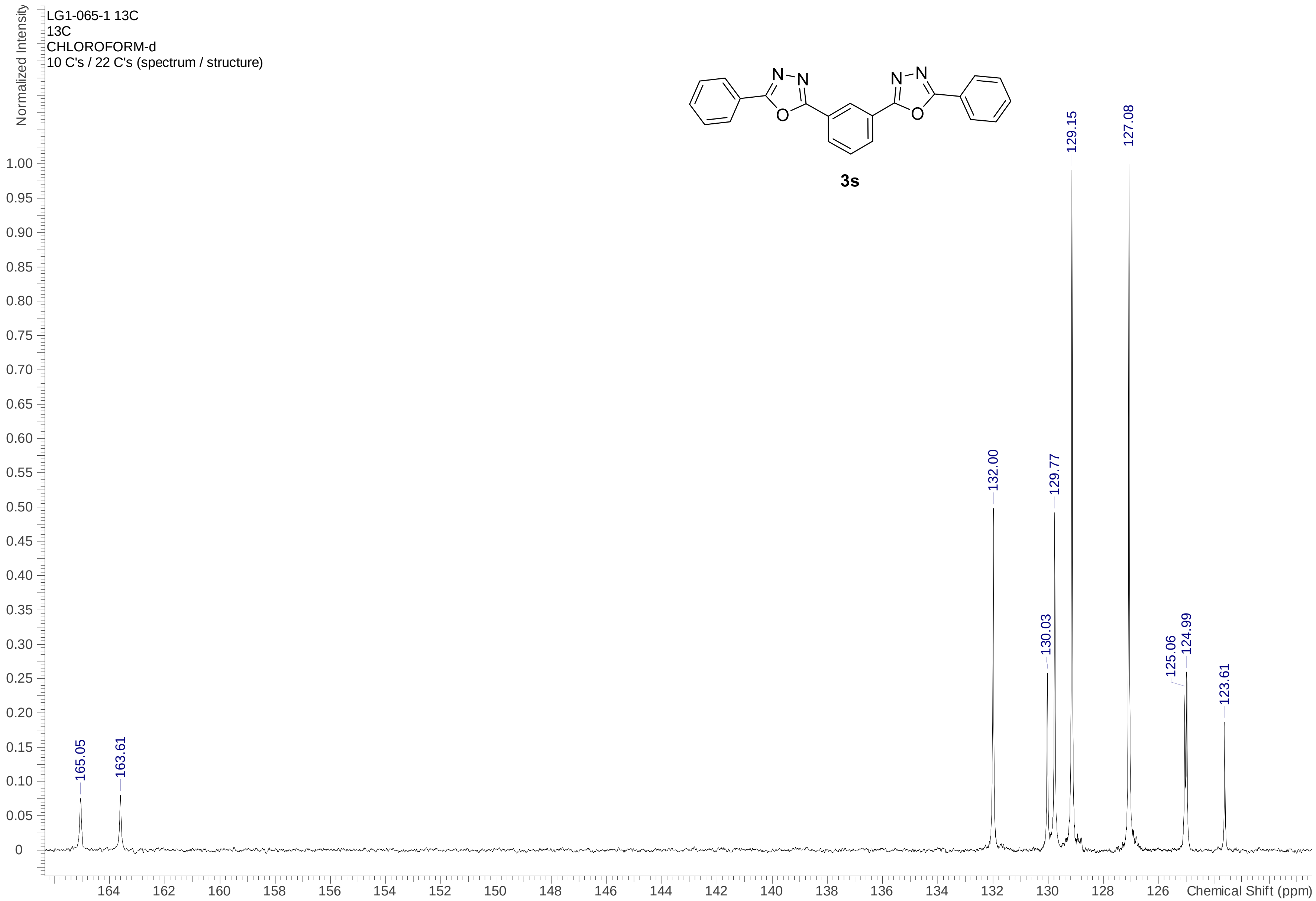

LG1-065-1 13C
13C
CHLOROFORM-d
10 C's / 22 C's (spectrum / structure)
3s
Normalized Intensity
Chemical Shift (ppm)
165.05
163.61
132.00
130.03
129.77
129.15
127.08
125.06
124.99
123.61

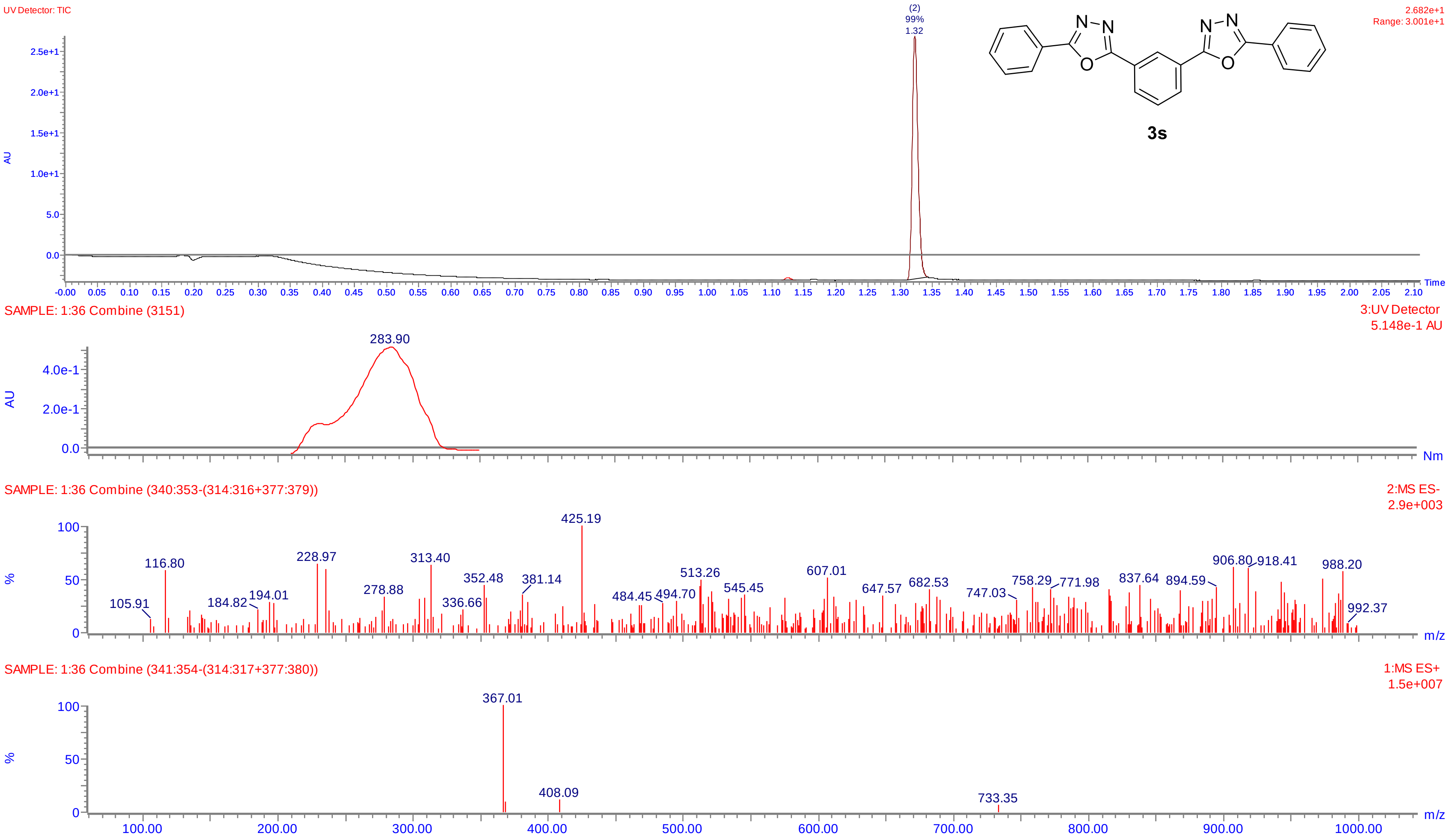

UV Detector: TIC
2.682e+1
Range: 3.001e+1
(2)
99%
1.32
AU
Time
3s
SAMPLE: 1:36 Combine (3151)
3:UV Detector
5.148e-1 AU
283.90
AU
Nm
SAMPLE: 1:36 Combine (340:353-(314:316+377:379))
2:MS ES-
2.9e+003
425.19
%
m/z
SAMPLE: 1:36 Combine (341:354-(314:317+377:380))
1:MS ES+
1.5e+007
367.01
408.09
733.35
%
m/z

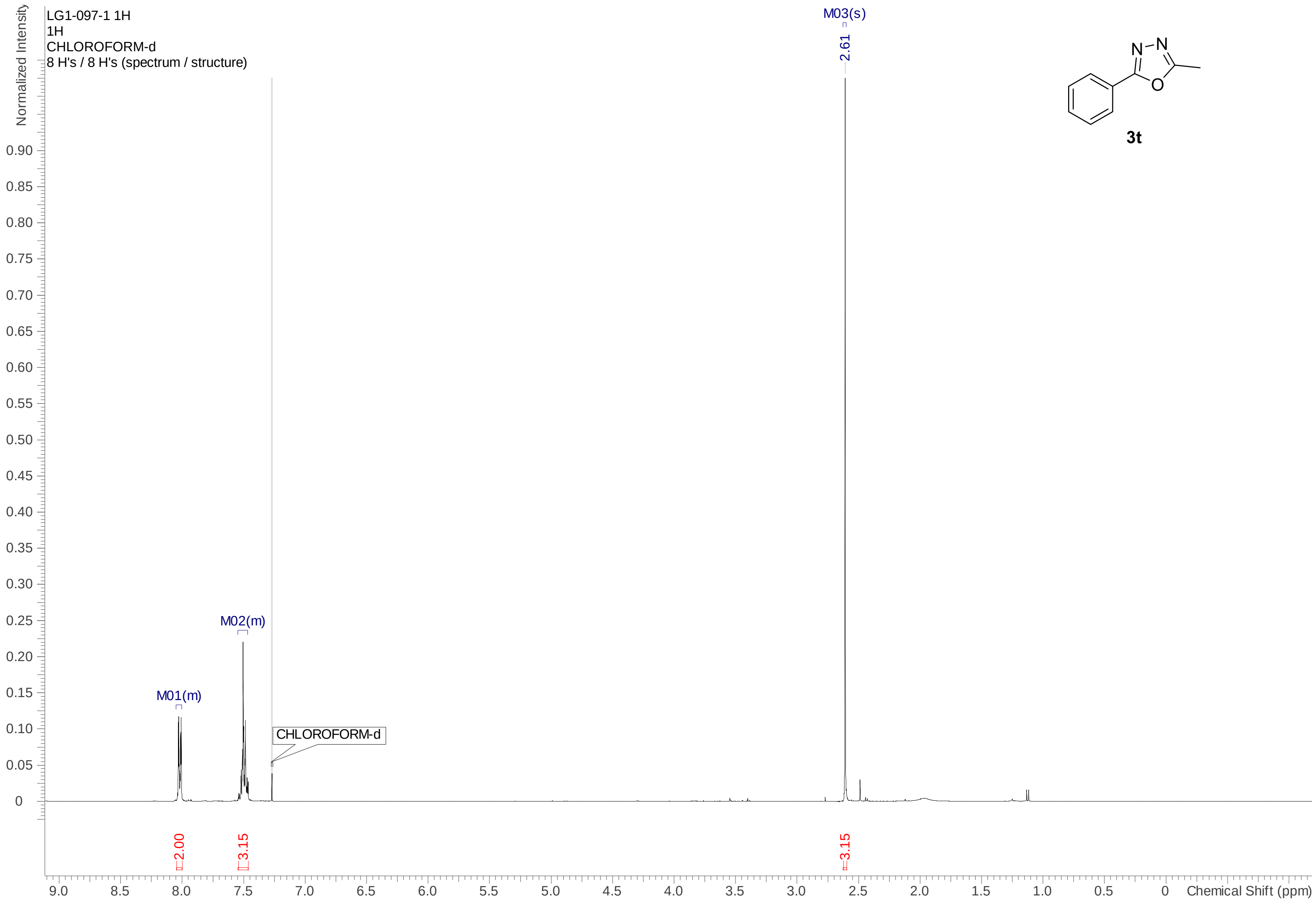
LG1-097-1 1H
1H
CHLOROFORM-d
8 H's / 8 H's (spectrum / structure)
Normalized Intensity
M01(m)
M02(m)
M03(s)
2.61
CHLOROFORM-d
2.00
3.15
3.15
N
N
O
3t
Chemical Shift (ppm)

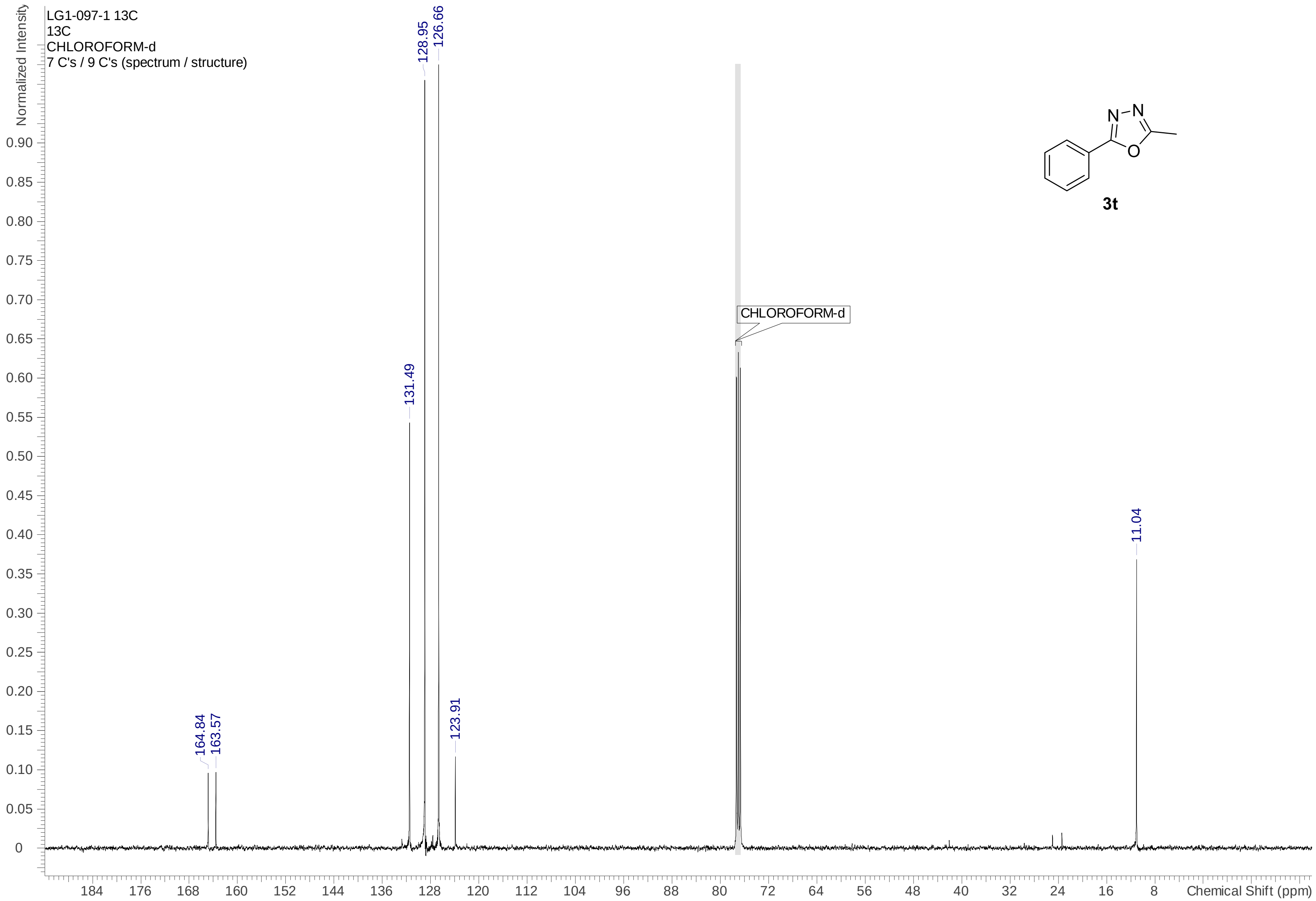
LG1-097-1 13C
13C
CHLOROFORM-d
7 C's / 9 C's (spectrum / structure)
Normalized Intensity
164.84
163.57
131.49
128.95
126.66
123.91
CHLOROFORM-d
11.04
N
N
O
3t
Chemical Shift (ppm)

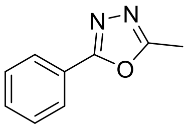
N
N
O

3t

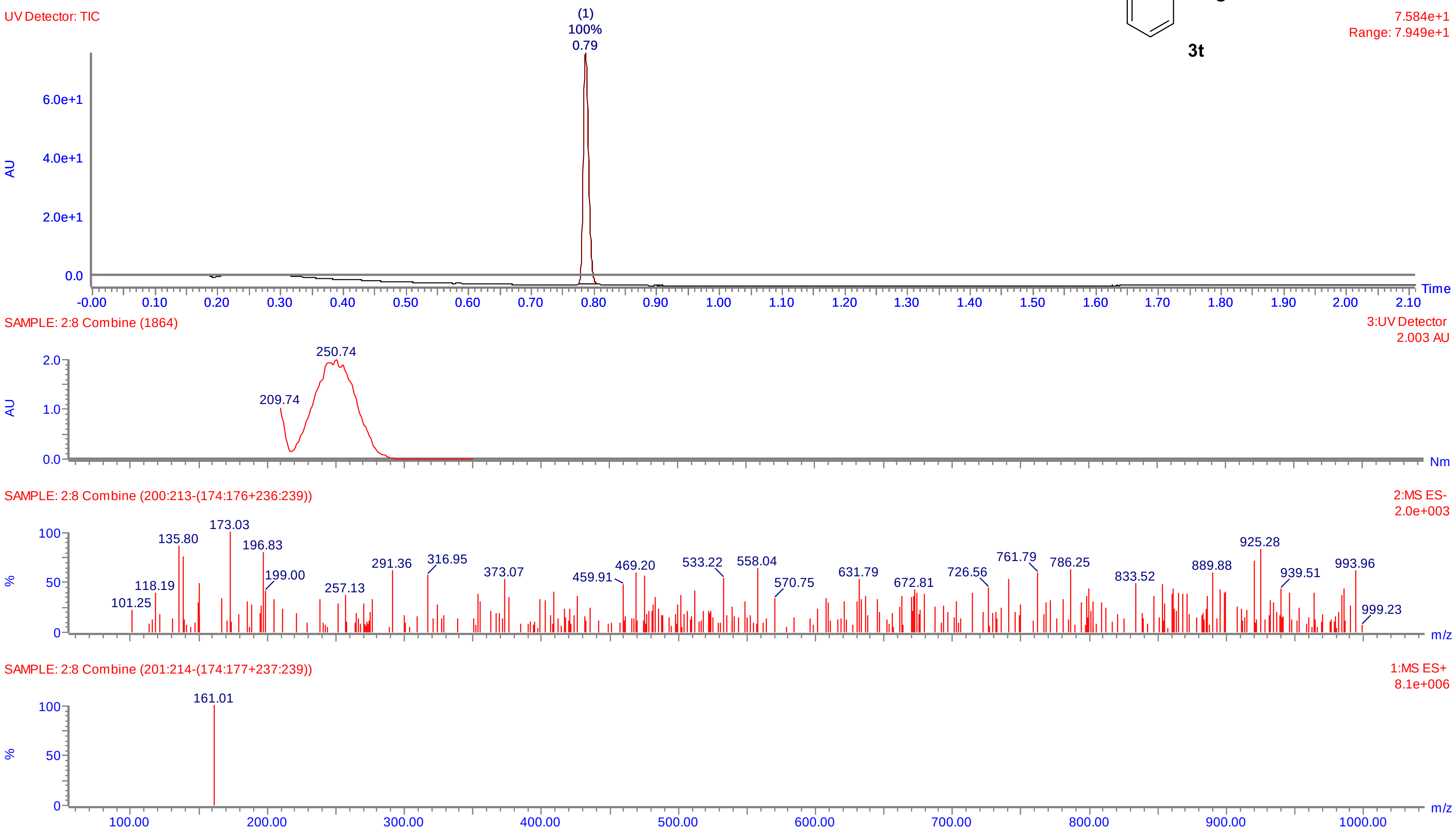
UV Detector: TIC
7.584e+1
Range: 7.949e+1
(1)
100%
0.79
AU
6.0e+1
4.0e+1
2.0e+1
0.0
Time
-0.00
0.10
0.20
0.30
0.40
0.50
0.60
0.70
0.80
0.90
1.00
1.10
1.20
1.30
1.40
1.50
1.60
1.70
1.80
1.90
2.00
2.10
SAMPLE: 2:8 Combine (1864)
3:UV Detector
2.003 AU
250.74
209.74
AU
2.0
1.0
0.0
Nm
SAMPLE: 2:8 Combine (200:213-(174:176+236:239))
2:MS ES-
2.0e+003
%
100
50
0
173.03
135.80
196.83
199.00
118.19
101.25
257.13
291.36
316.95
373.07
459.91
469.20
533.22
558.04
570.75
631.79
672.81
726.56
761.79
786.25
833.52
889.88
925.28
939.51
993.96
999.23
m/z
SAMPLE: 2:8 Combine (201:214-(174:177+237:239))
1:MS ES+
8.1e+006
161.01
%
100
50
0
m/z
100.00
200.00
300.00
400.00
500.00
600.00
700.00
800.00
900.00
1000.00

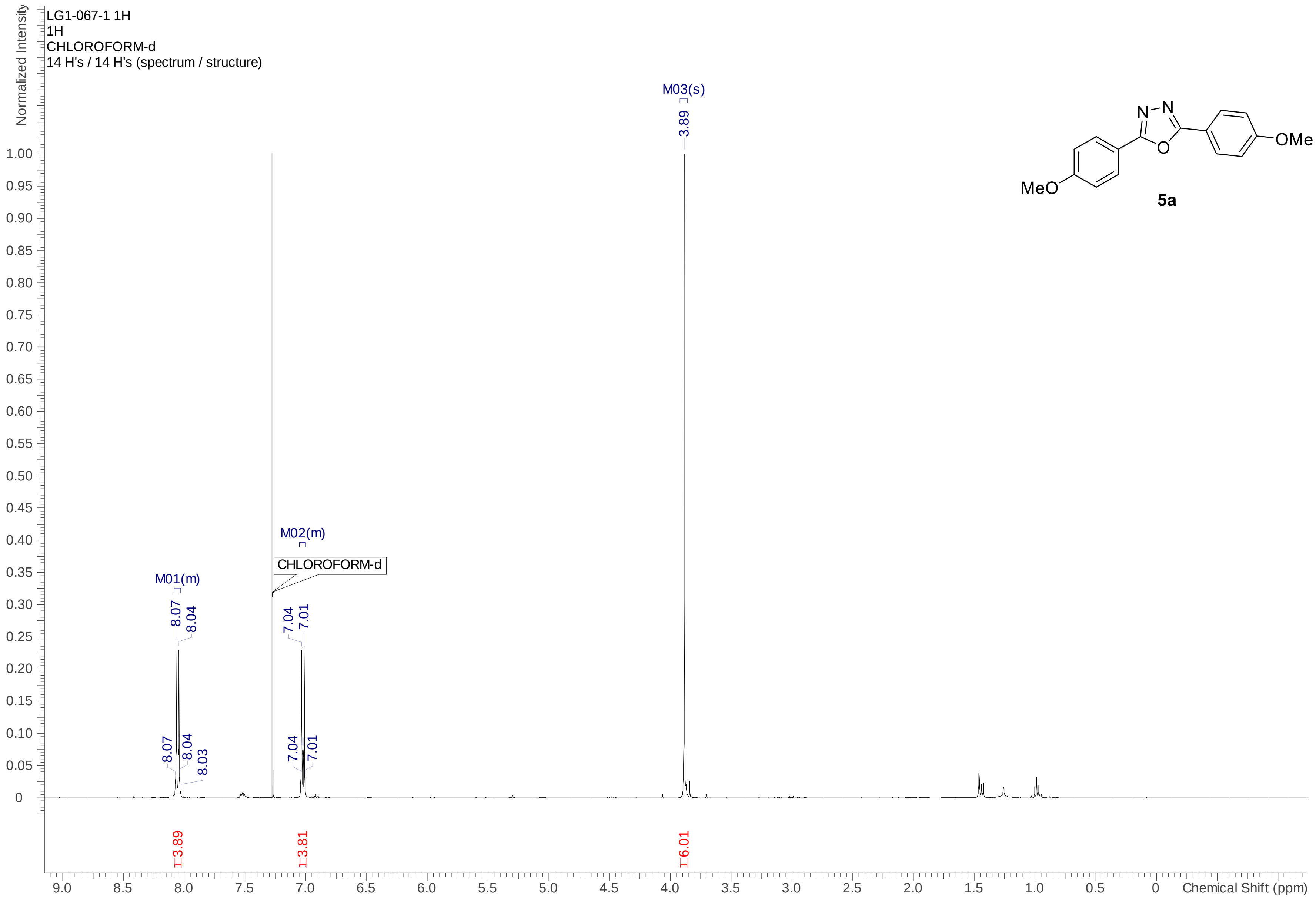

LG1-067-1 1H
1H
CHLOROFORM-d
14 H's / 14 H's (spectrum / structure)
Normalized Intensity
M03(s)
3.89
M02(m)
CHLOROFORM-d
M01(m)
8.07
8.04
7.04
7.01
8.07
8.04
8.03
7.04
7.01
3.89
3.81
6.01
Chemical Shift (ppm)
N–N
O
MeO
OMe

5a

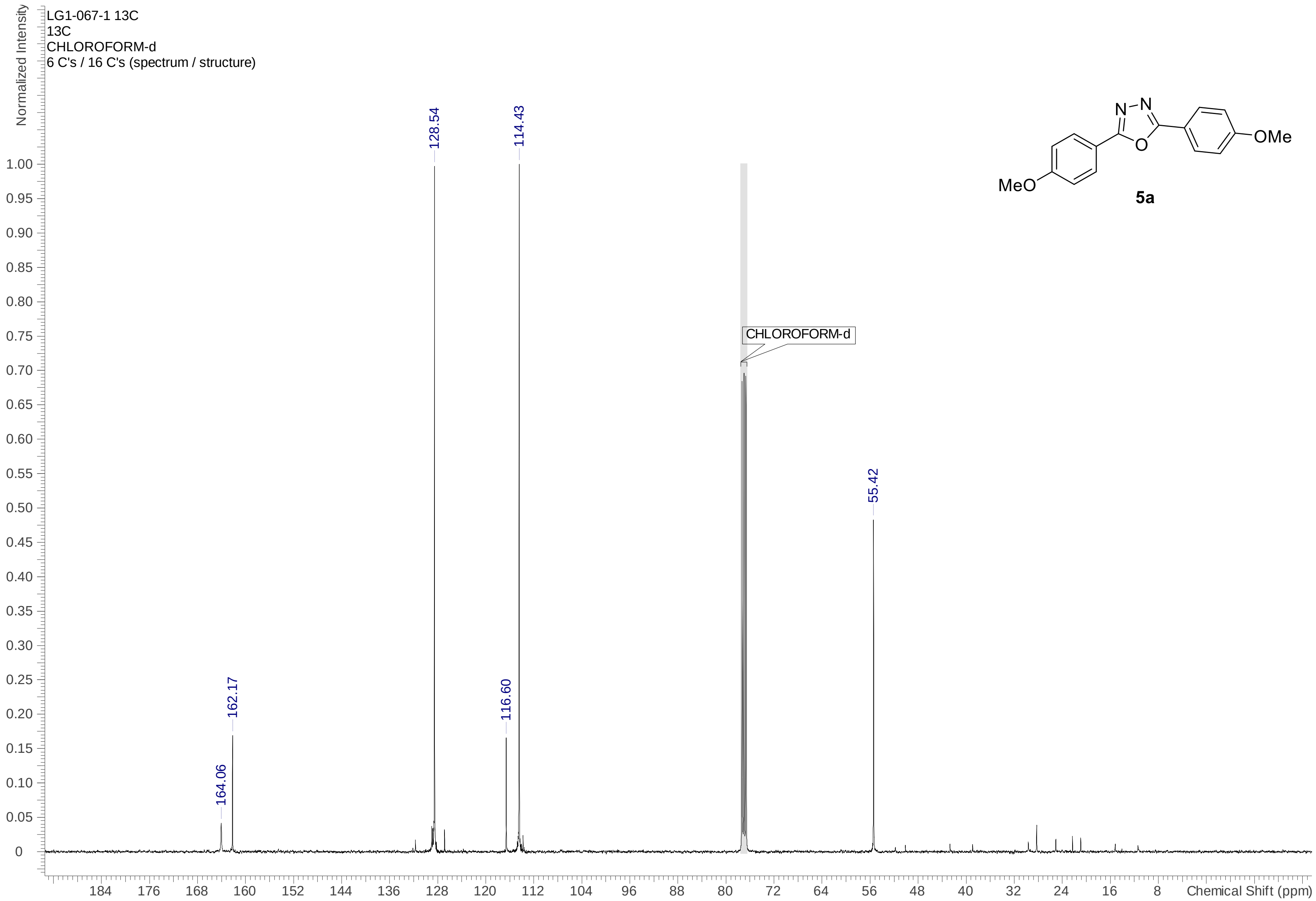
LG1-067-1 13C
13C
CHLOROFORM-d
6 C's / 16 C's (spectrum / structure)
Normalized Intensity
128.54
114.43
CHLOROFORM-d
55.42
162.17
116.60
164.06
Chemical Shift (ppm)
N
N
O
OMe
MeO
5a

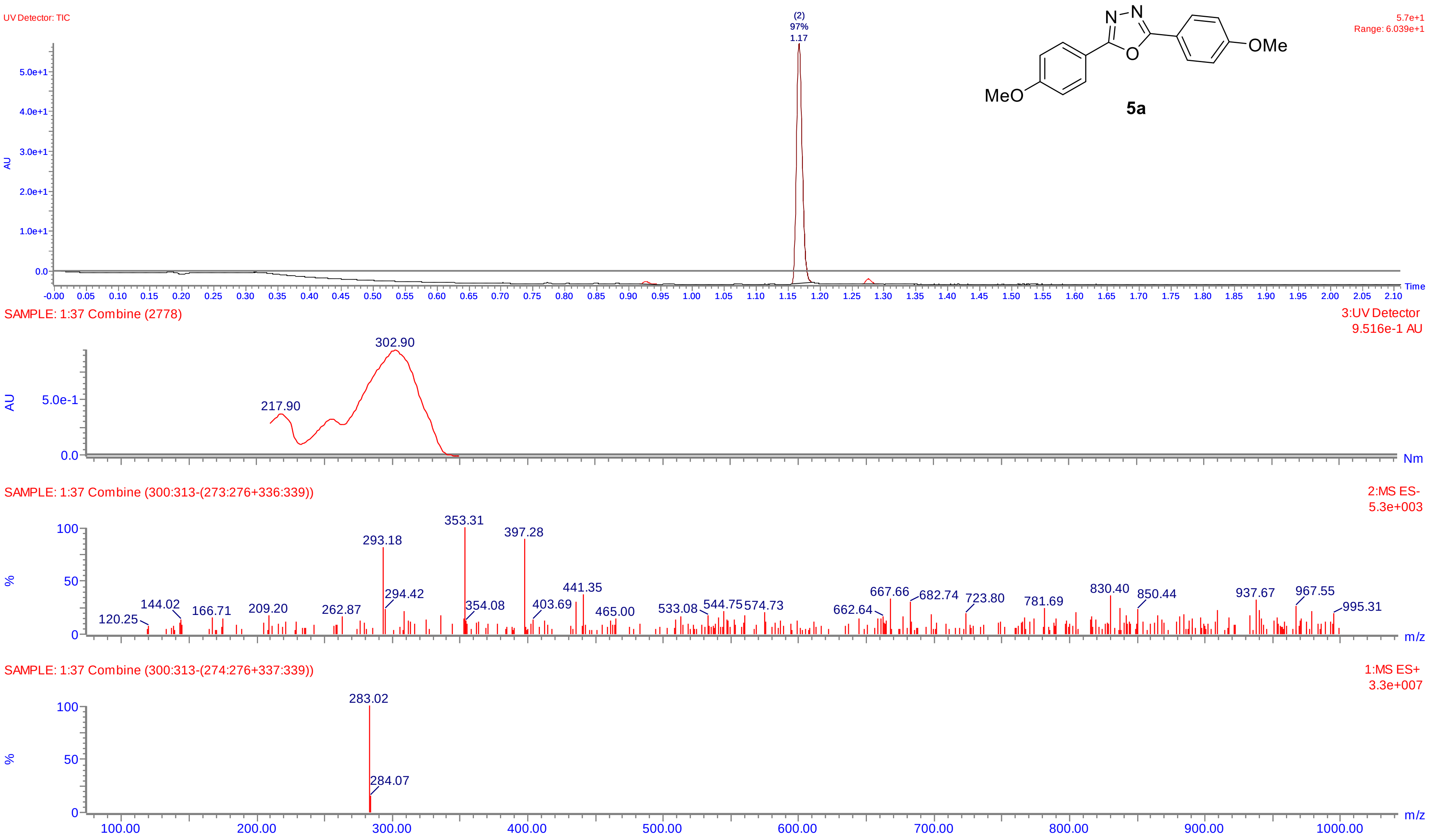
N–N
O
MeO
OMe
5a
UV Detector: TIC
(2)
97%
1.17
5.7e+1
Range: 6.039e+1
AU
Time
SAMPLE: 1:37 Combine (2778)
3:UV Detector
9.516e-1 AU
302.90
217.90
Nm
SAMPLE: 1:37 Combine (300:313-(273:276+336:339))
2:MS ES-
5.3e+003
353.31
293.18
397.28
294.42
441.35
144.02
166.71
209.20
262.87
354.08
403.69
465.00
533.08
544.75
574.73
662.64
667.66
682.74
723.80
781.69
830.40
850.44
937.67
967.55
995.31
120.25
m/z
SAMPLE: 1:37 Combine (300:313-(274:276+337:339))
1:MS ES+
3.3e+007
283.02
284.07
m/z

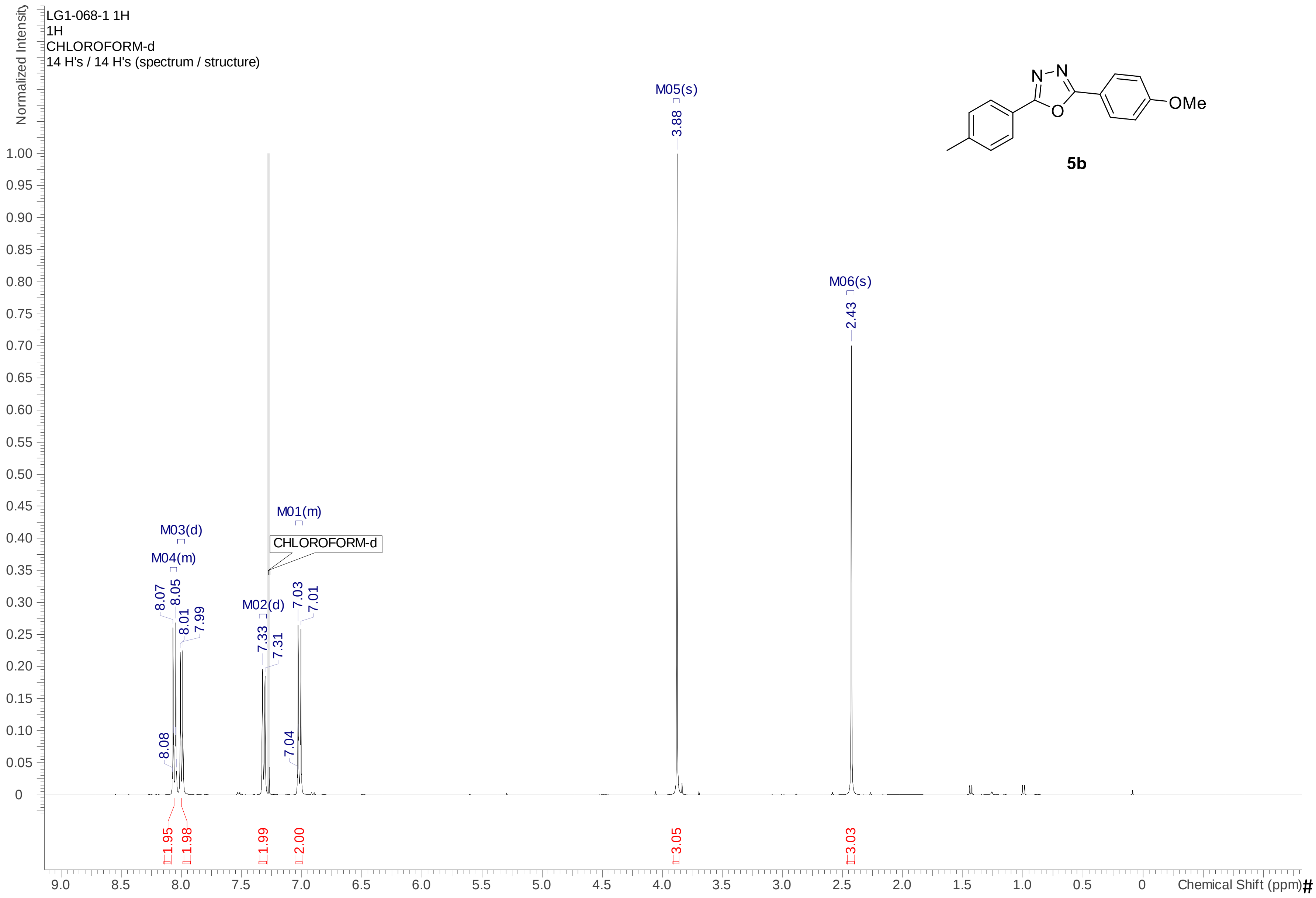
LG1-068-1 1H
1H
CHLOROFORM-d
14 H's / 14 H's (spectrum / structure)
Normalized Intensity
Chemical Shift (ppm)
M05(s)
3.88
M06(s)
2.43
M01(m)
CHLOROFORM-d
M03(d)
M04(m)
8.07
8.05
8.01
7.99
8.08
M02(d)
7.33
7.31
7.03
7.01
7.04
1.95
1.98
1.99
2.00
3.05
3.03
N
N
O
OMe
5b

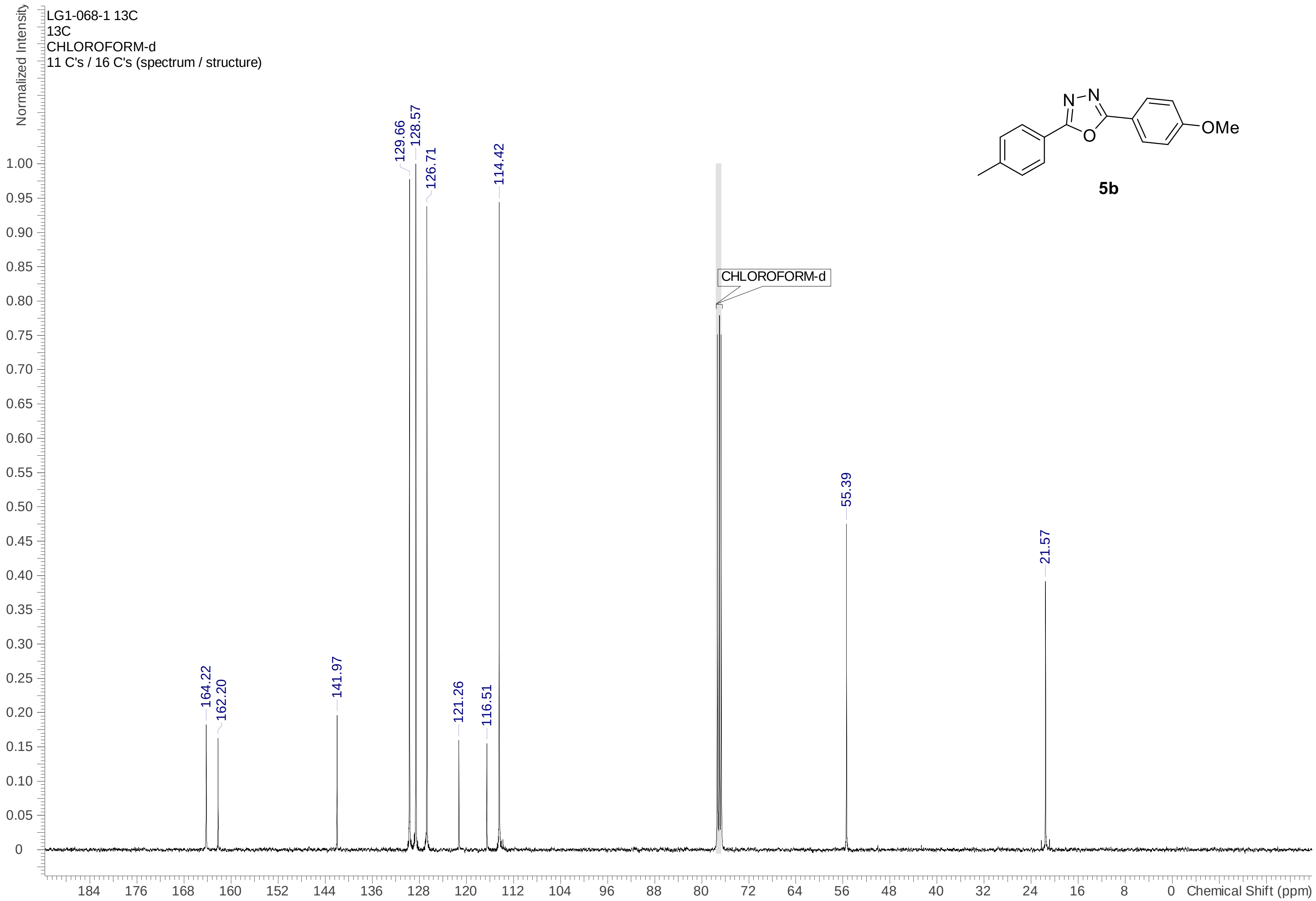
LG1-068-1 13C
13C
CHLOROFORM-d
11 C's / 16 C's (spectrum / structure)
Normalized Intensity
164.22
162.20
141.97
129.66
128.57
126.71
121.26
116.51
114.42
CHLOROFORM-d
55.39
21.57
Chemical Shift (ppm)
N
N
O
OMe
5b

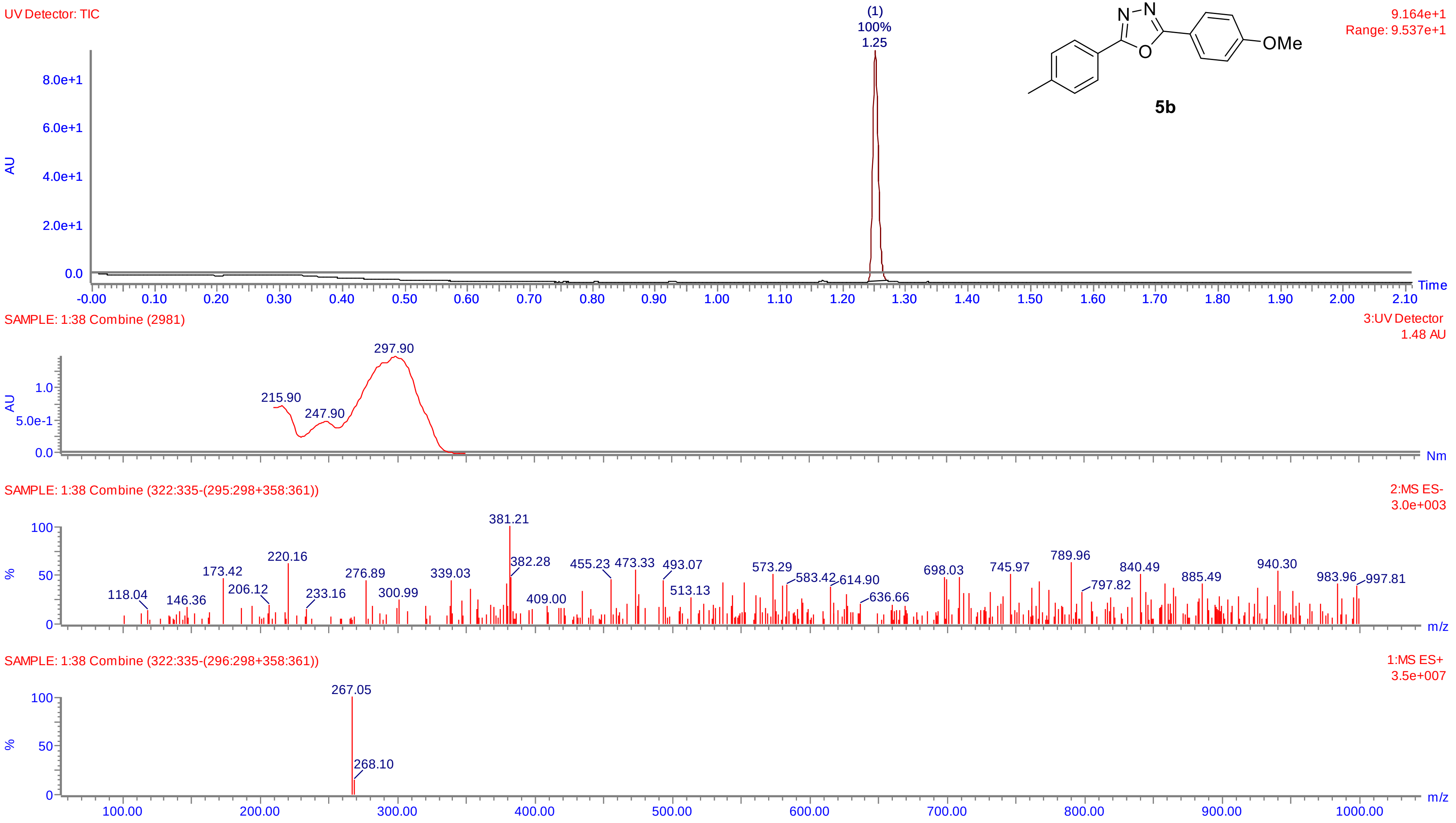
UV Detector: TIC
(1)
100%
1.25
9.164e+1
Range: 9.537e+1
N
N
O
OMe
5b
8.0e+1
6.0e+1
4.0e+1
2.0e+1
0.0
AU
Time
-0.00 0.10 0.20 0.30 0.40 0.50 0.60 0.70 0.80 0.90 1.00 1.10 1.20 1.30 1.40 1.50 1.60 1.70 1.80 1.90 2.00 2.10
SAMPLE: 1:38 Combine (2981)
3:UV Detector
1.48 AU
297.90
215.90
247.90
1.0
5.0e-1
0.0
Nm
SAMPLE: 1:38 Combine (322:335-(295:298+358:361))
2:MS ES-
3.0e+003
381.21
382.28
220.16
173.42
206.12
118.04
146.36
233.16
276.89
300.99
339.03
409.00
455.23
473.33
493.07
513.13
573.29
583.42
614.90
636.66
698.03
745.97
789.96
797.82
840.49
885.49
940.30
983.96
997.81
100
50
0
%
m/z
SAMPLE: 1:38 Combine (322:335-(296:298+358:361))
1:MS ES+
3.5e+007
267.05
268.10
100.00 200.00 300.00 400.00 500.00 600.00 700.00 800.00 900.00 1000.00

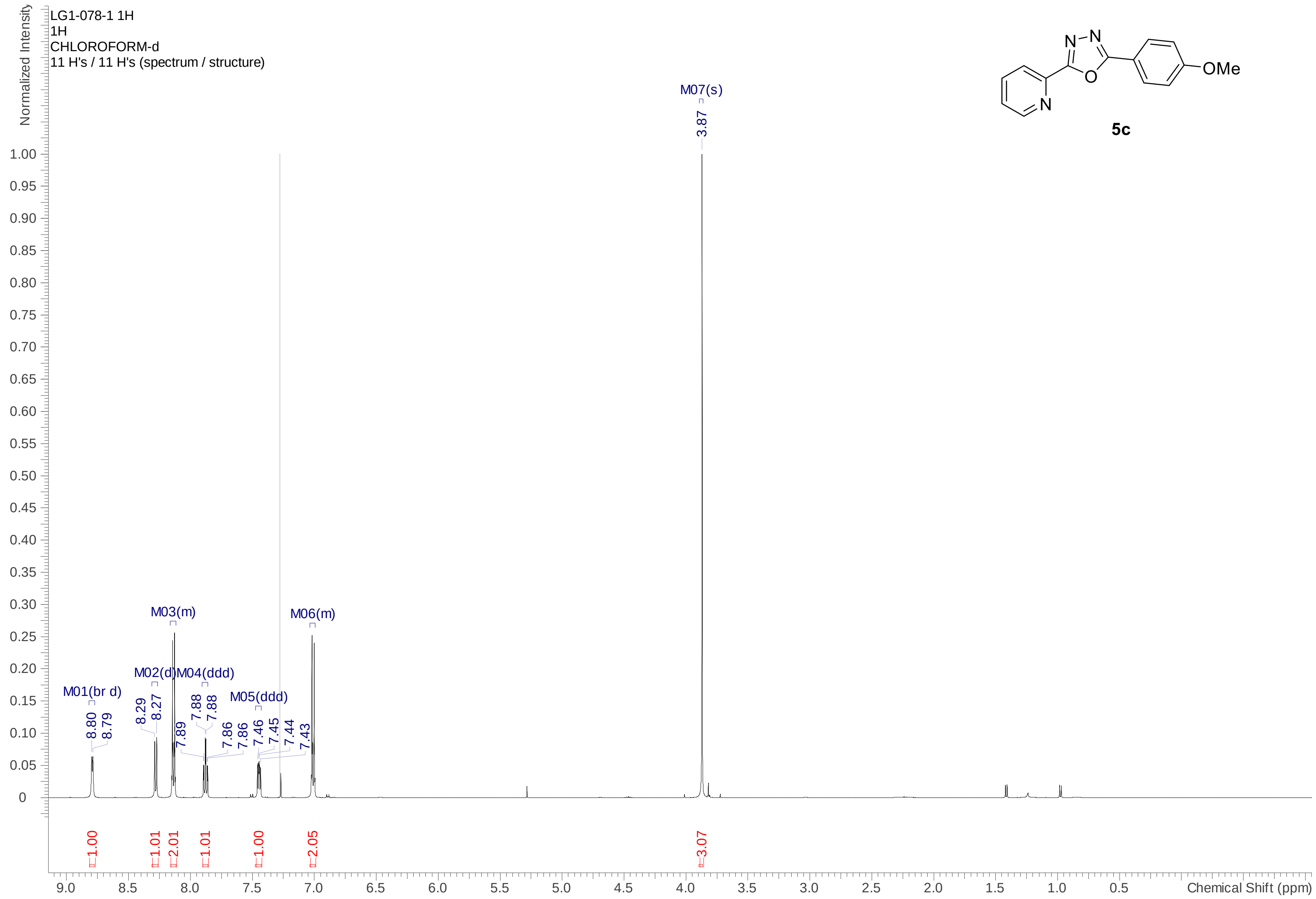

LG1-078-1 1H
1H
CHLOROFORM-d
11 H's / 11 H's (spectrum / structure)
Normalized Intensity
M01(br d)
8.80
8.79
M02(d)
8.29
8.27
M03(m)
M04(ddd)
7.89
7.88
7.88
7.86
7.86
M05(ddd)
7.46
7.45
7.44
7.43
M06(m)
M07(s)
3.87
1.00
1.01
2.01
1.01
1.00
2.05
3.07
Chemical Shift (ppm)
N
N
O
N
OMe

5c

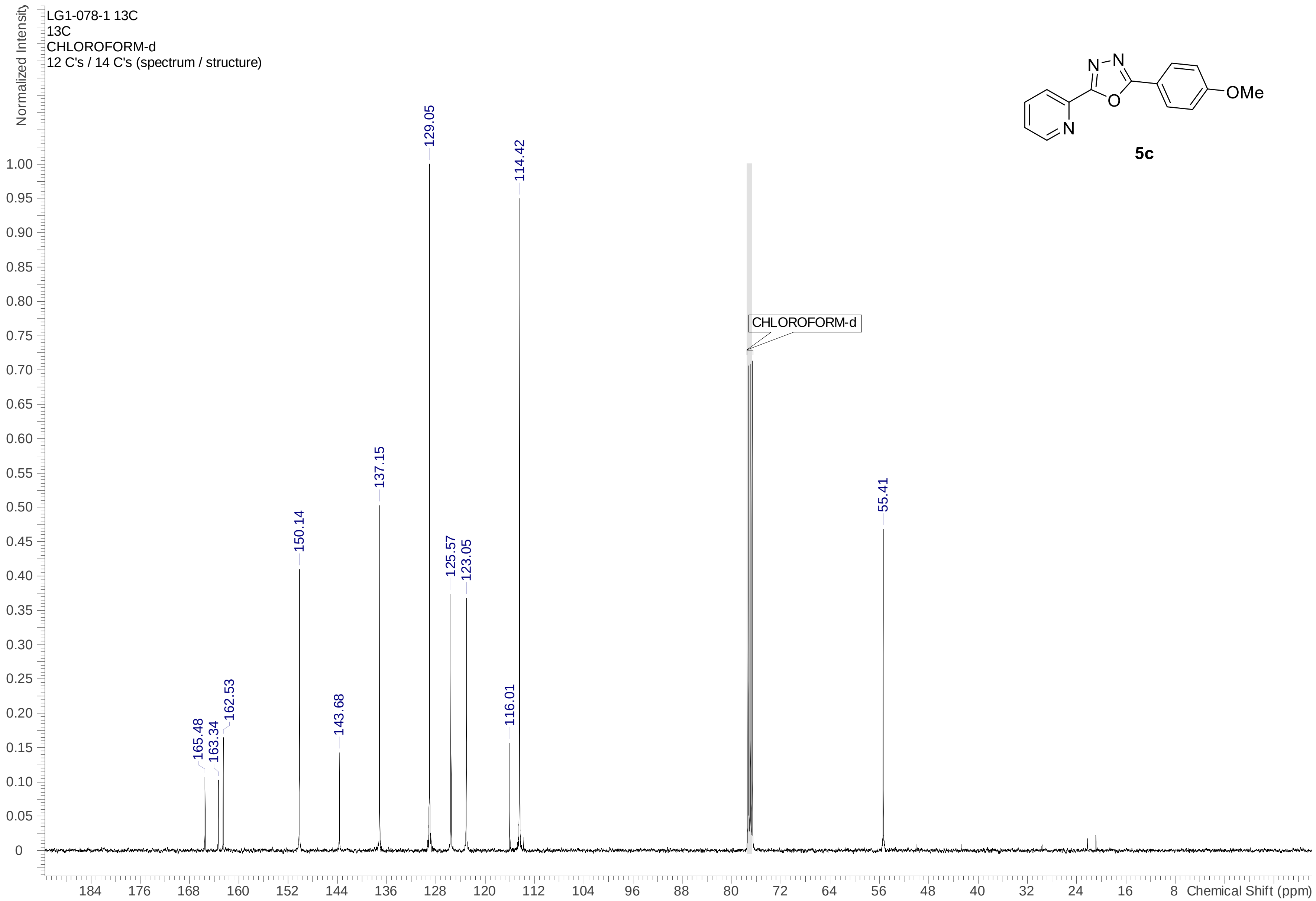
LG1-078-1 13C
13C
CHLOROFORM-d
12 C's / 14 C's (spectrum / structure)
Normalized Intensity
165.48
163.34
162.53
150.14
143.68
137.15
129.05
125.57
123.05
116.01
114.42
CHLOROFORM-d
55.41
OMe
5c
Chemical Shift (ppm)

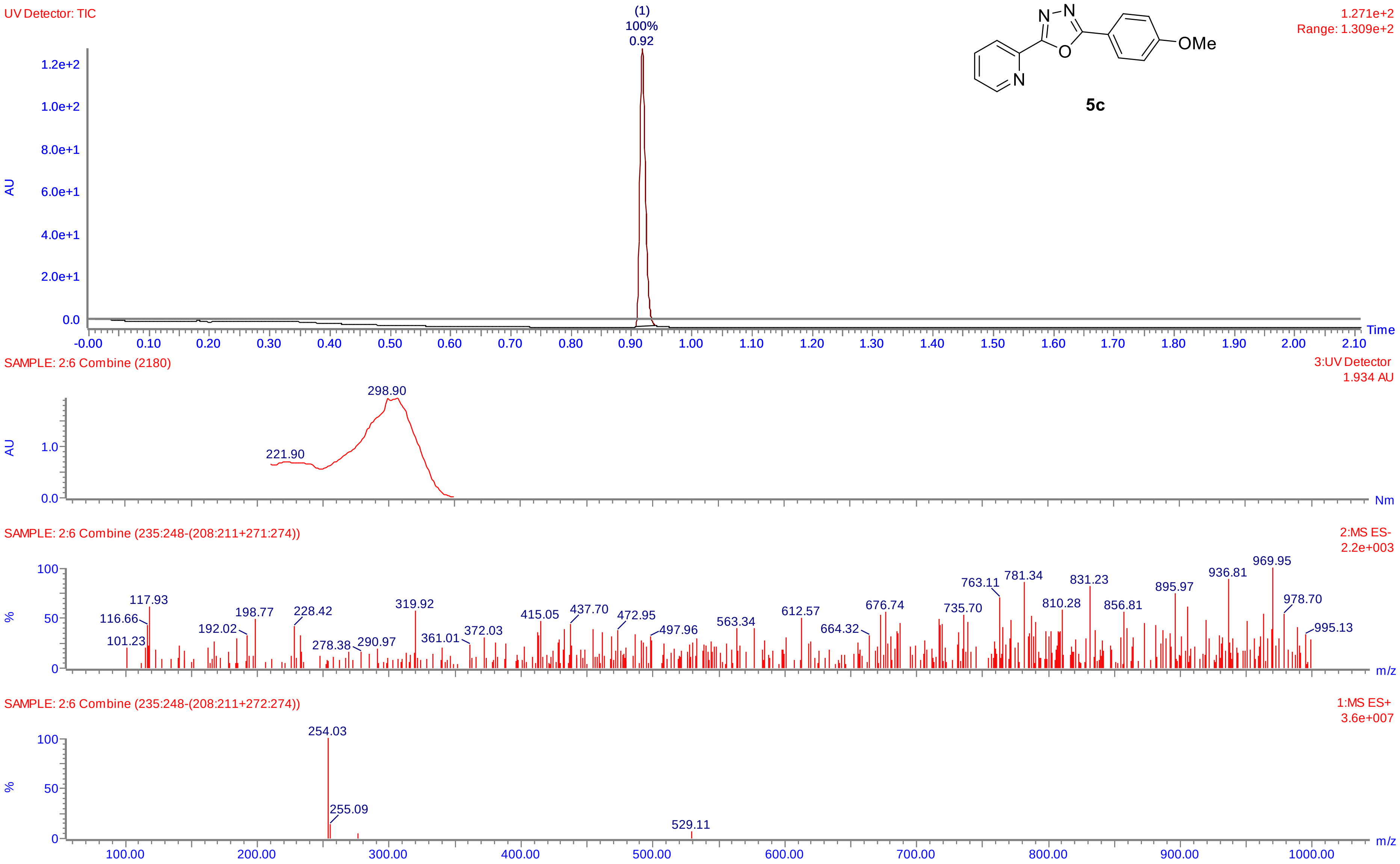

UV Detector: TIC
(1)
100%
0.92
1.271e+2
Range: 1.309e+2
AU
Time
SAMPLE: 2:6 Combine (2180)
3:UV Detector
1.934 AU
298.90
221.90
Nm
SAMPLE: 2:6 Combine (235:248-(208:211+271:274))
2:MS ES-
2.2e+003
117.93
116.66
101.23
192.02
198.77
228.42
278.38
290.97
319.92
361.01
372.03
415.05
437.70
472.95
497.96
563.34
612.57
664.32
676.74
735.70
763.11
781.34
810.28
831.23
856.81
895.97
936.81
969.95
978.70
995.13
m/z
SAMPLE: 2:6 Combine (235:248-(208:211+272:274))
1:MS ES+
3.6e+007
254.03
255.09
529.11
m/z
N
N
N
O
OMe

5c

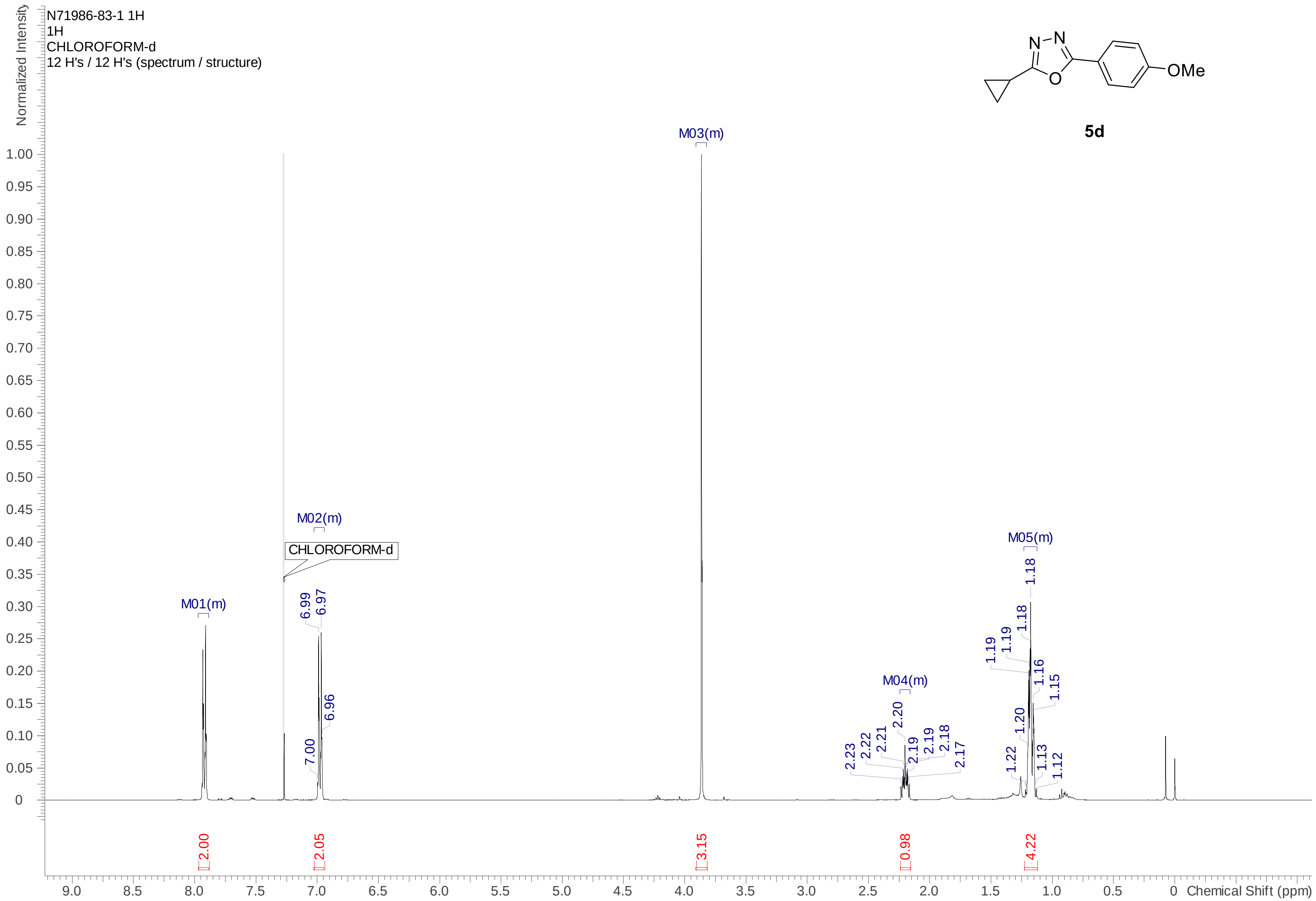

N71986-83-1 1H
1H
CHLOROFORM-d
12 H's / 12 H's (spectrum / structure)
Normalized Intensity
Chemical Shift (ppm)
M01(m)
M02(m)
M03(m)
M04(m)
M05(m)
CHLOROFORM-d
OMe

5d

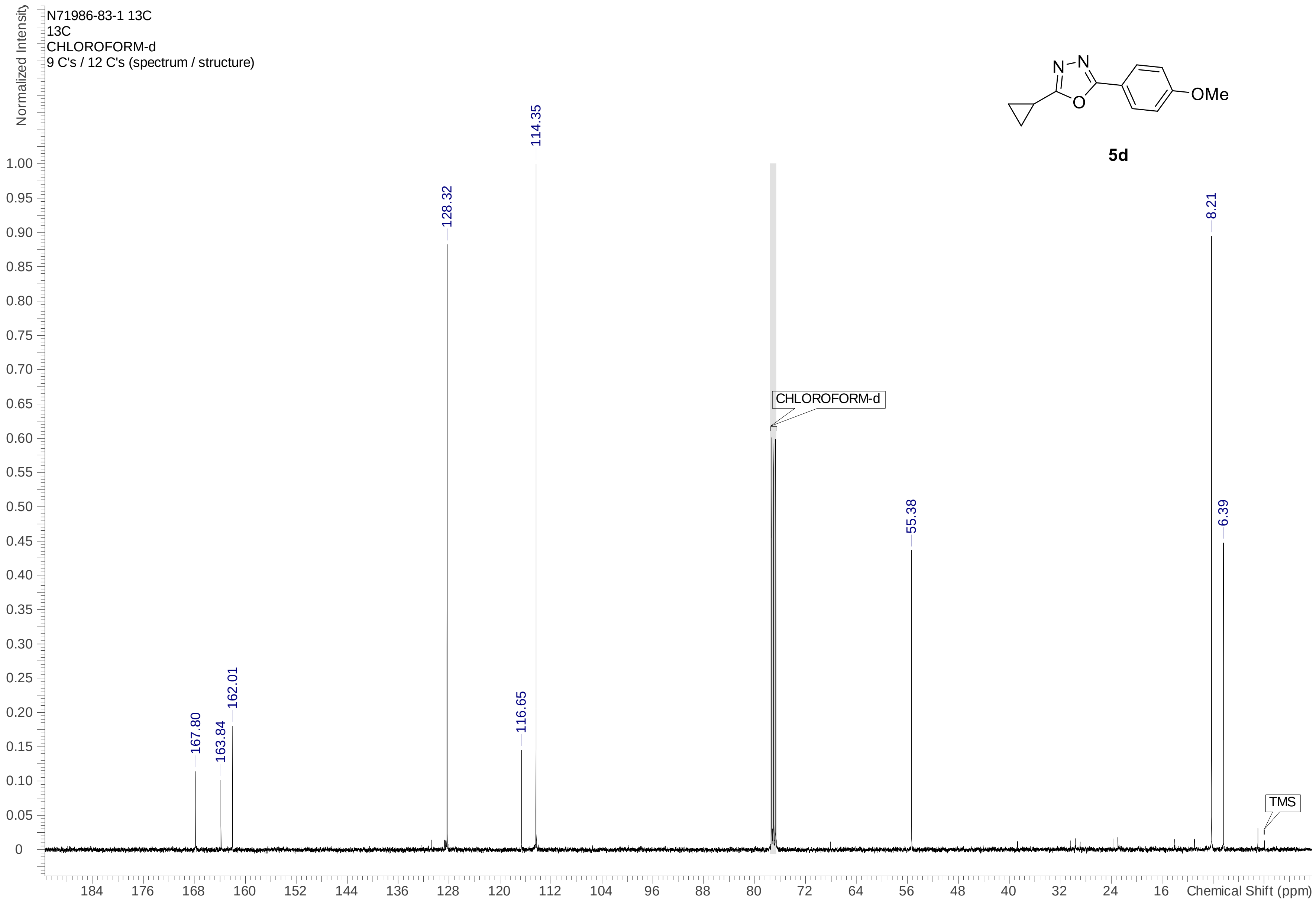
N71986-83-1 13C
13C
CHLOROFORM-d
9 C's / 12 C's (spectrum / structure)
Normalized Intensity
Chemical Shift (ppm)
167.80
163.84
162.01
128.32
116.65
114.35
CHLOROFORM-d
55.38
8.21
6.39
TMS
OMe
5d

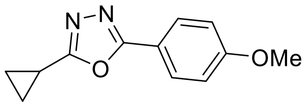
N–N
O
OMe

5d

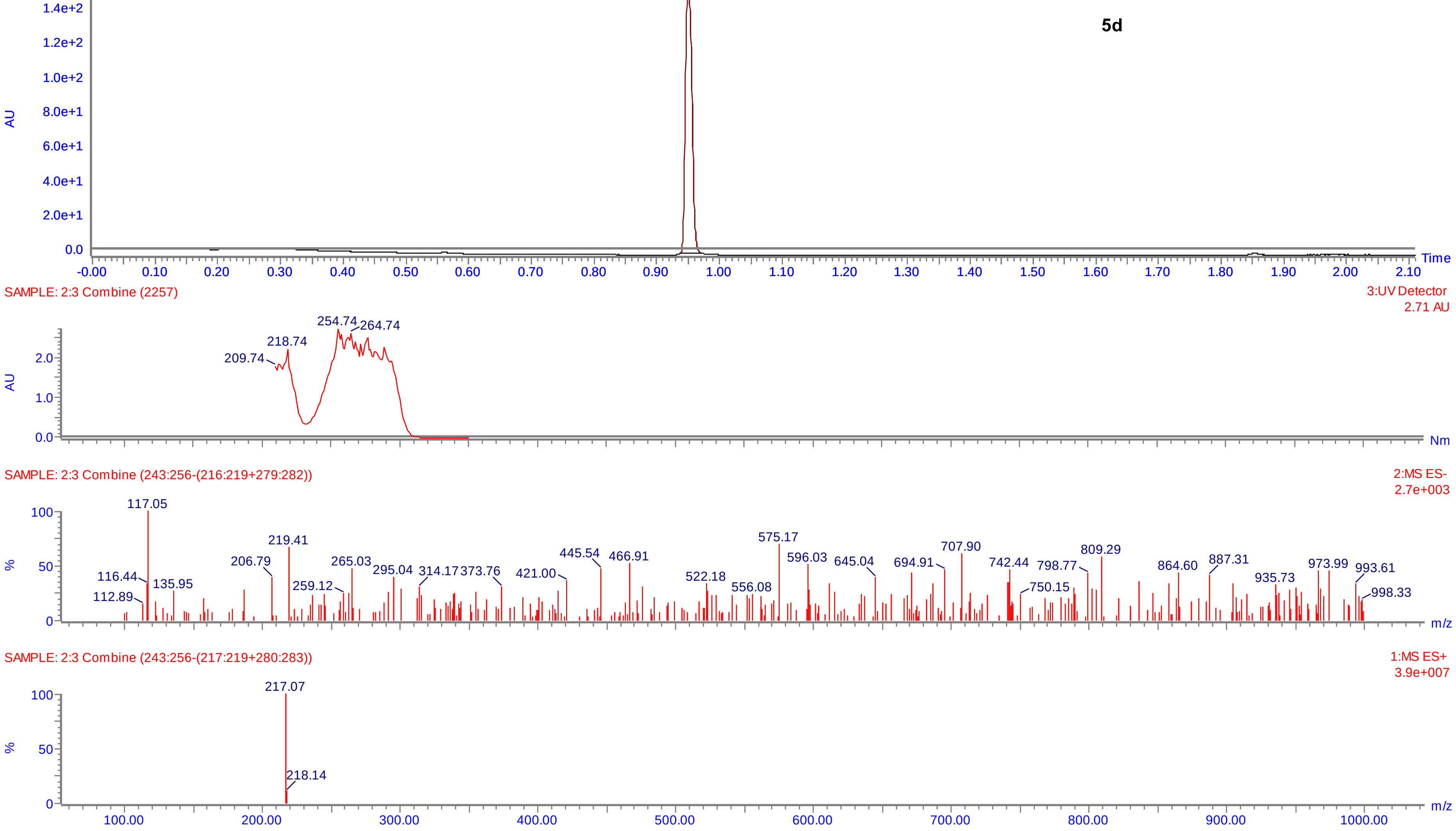
UV Detector: TIC
(1)
100%
0.95
1.538e+2
Range: 1.574e+2
AU
Time
SAMPLE: 2:3 Combine (2257)
3:UV Detector
2.71 AU
209.74
218.74
254.74
264.74
Nm
SAMPLE: 2:3 Combine (243:256-(216:219+279:282))
2:MS ES-
2.7e+003
117.05
219.41
206.79
116.44
112.89
135.95
259.12
265.03
295.04
314.17
373.76
421.00
445.54
466.91
522.18
556.08
575.17
596.03
645.04
694.91
707.90
742.44
750.15
798.77
809.29
864.60
887.31
935.73
973.99
993.61
998.33
m/z
SAMPLE: 2:3 Combine (243:256-(217:219+280:283))
1:MS ES+
3.9e+007
217.07
218.14
m/z

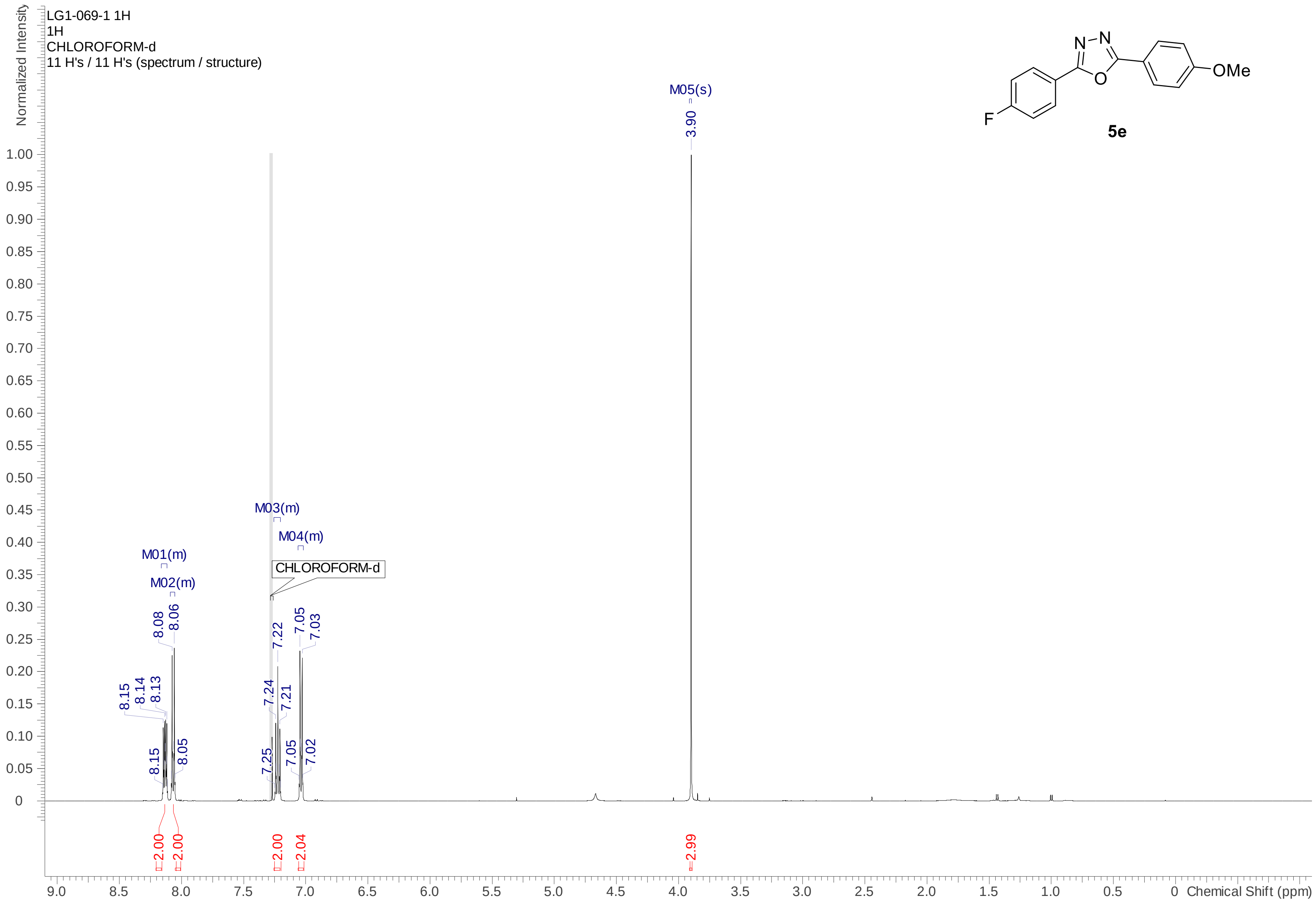
LG1-069-1 1H
1H
CHLOROFORM-d
11 H's / 11 H's (spectrum / structure)
Normalized Intensity
M01(m)
M02(m)
M03(m)
M04(m)
M05(s)
CHLOROFORM-d
8.15
8.14
8.13
8.08
8.06
8.05
7.25
7.24
7.22
7.21
7.05
7.03
7.02
3.90
2.00
2.00
2.00
2.04
2.99
N
N
O
F
OMe
5e
Chemical Shift (ppm)

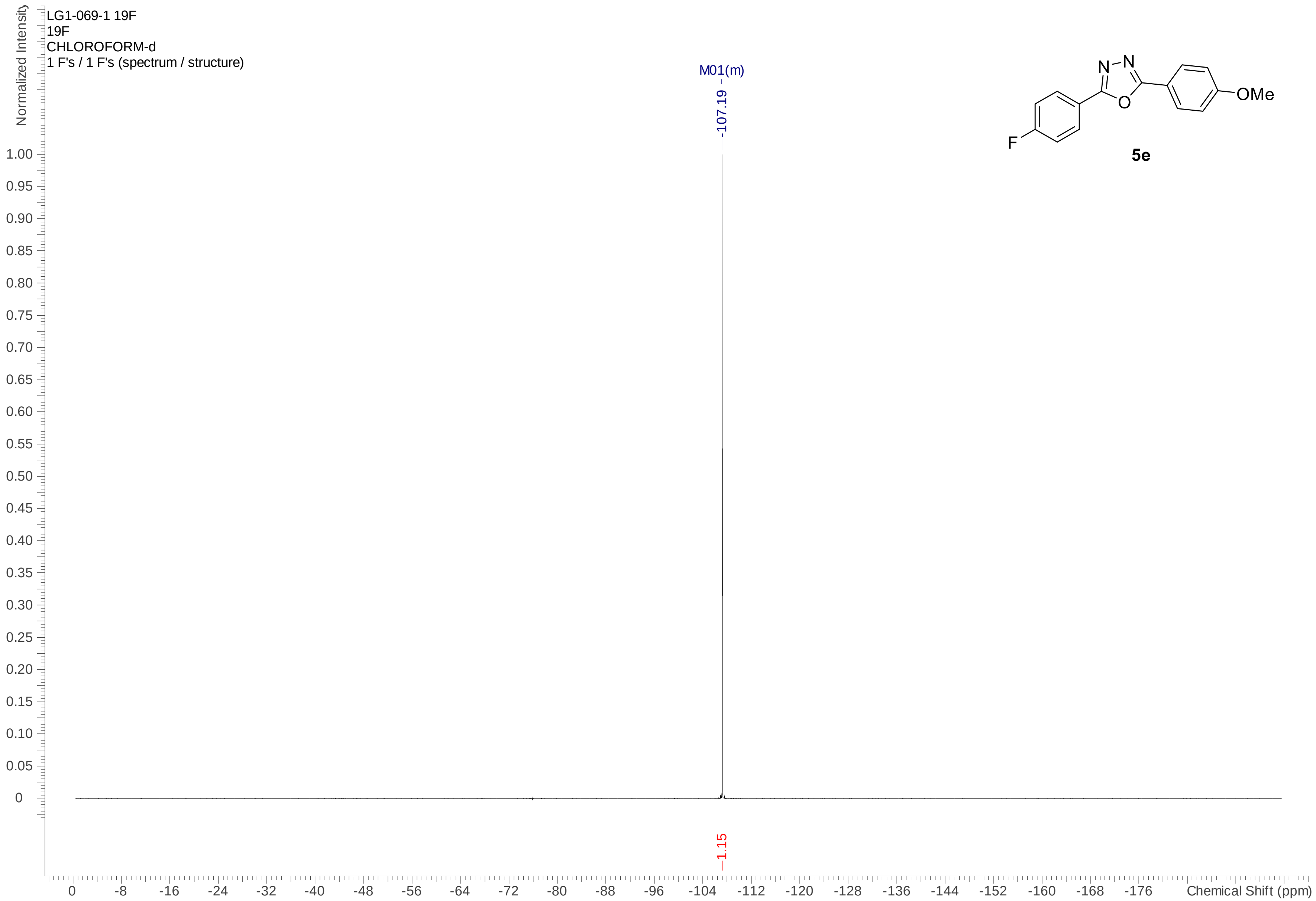
LG1-069-1 19F
19F
CHLOROFORM-d
1 F's / 1 F's (spectrum / structure)
M01(m)
-107.19
-1.15
Normalized Intensity
Chemical Shift (ppm)
F
OMe
5e

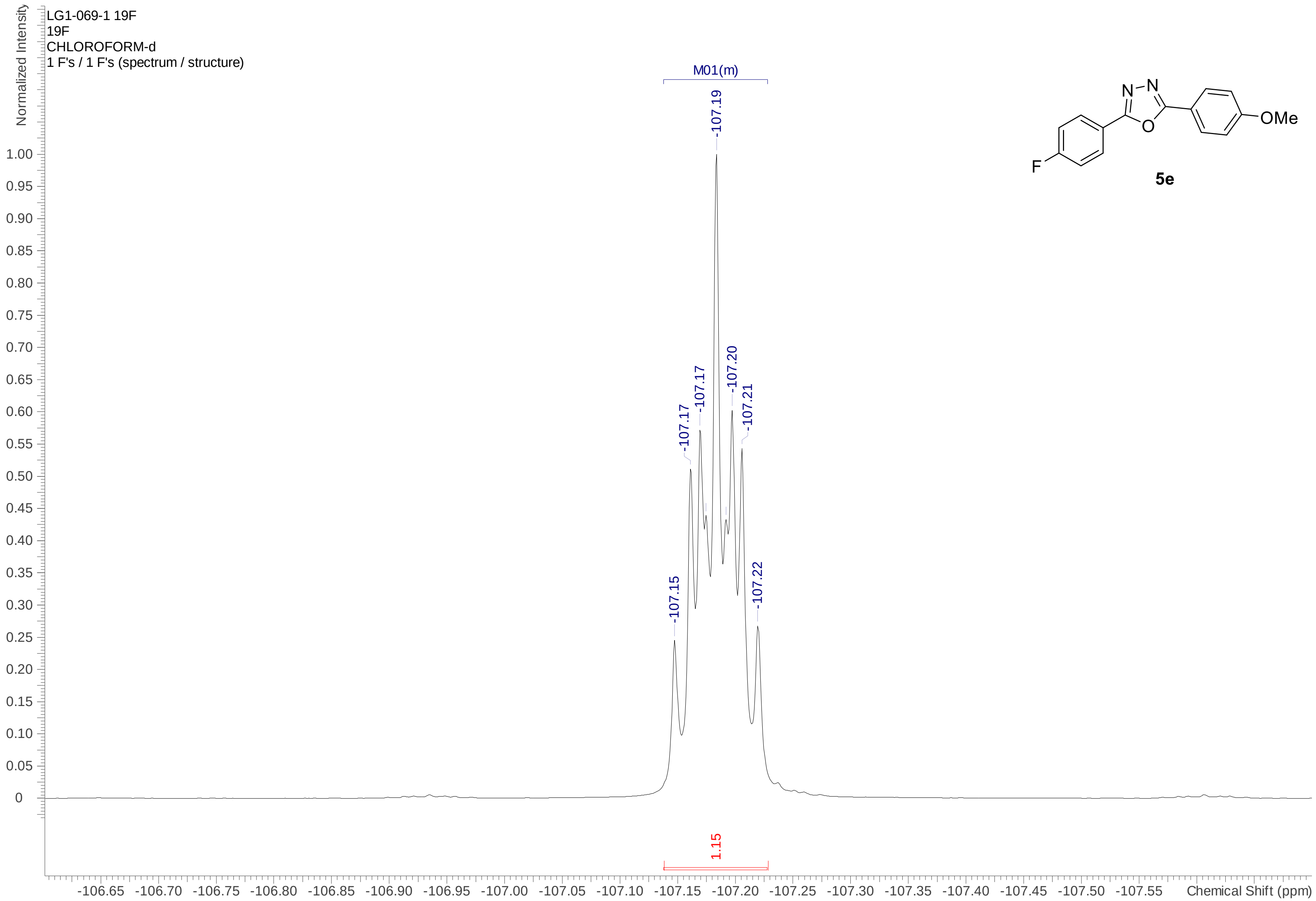
LG1-069-1 19F
19F
CHLOROFORM-d
1 F's / 1 F's (spectrum / structure)
Normalized Intensity
M01(m)
-107.19
-107.17
-107.17
-107.20
-107.21
-107.15
-107.22
1.15
Chemical Shift (ppm)
F
N
N
O
OMe
5e

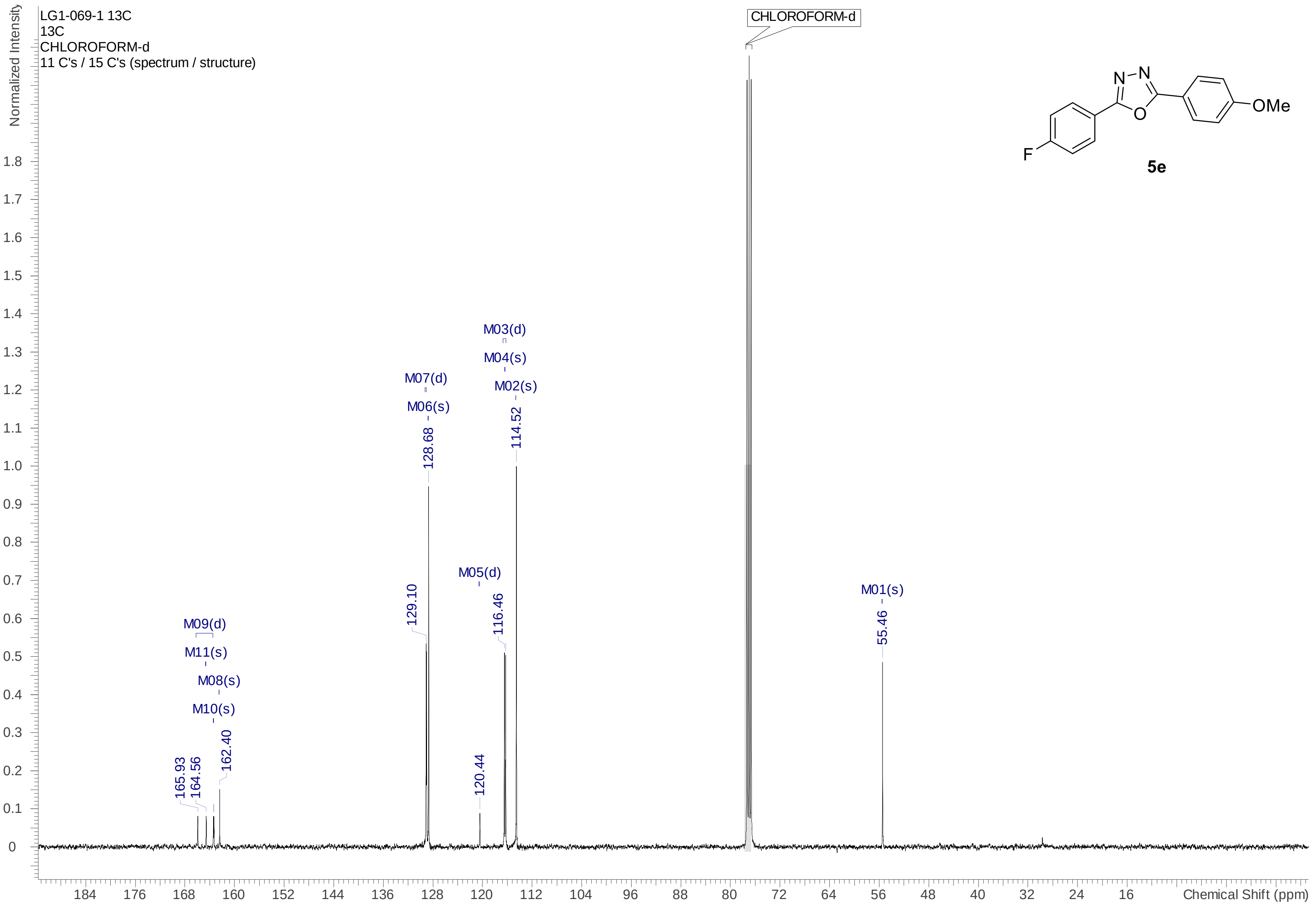
LG1-069-1 13C
13C
CHLOROFORM-d
11 C's / 15 C's (spectrum / structure)
Normalized Intensity
CHLOROFORM-d
N–N
O
OMe
F
5e
M09(d)
M11(s)
M08(s)
M10(s)
165.93
164.56
162.40
M07(d)
M06(s)
128.68
129.10
M05(d)
120.44
116.46
M03(d)
M04(s)
M02(s)
114.52
M01(s)
55.46
Chemical Shift (ppm)

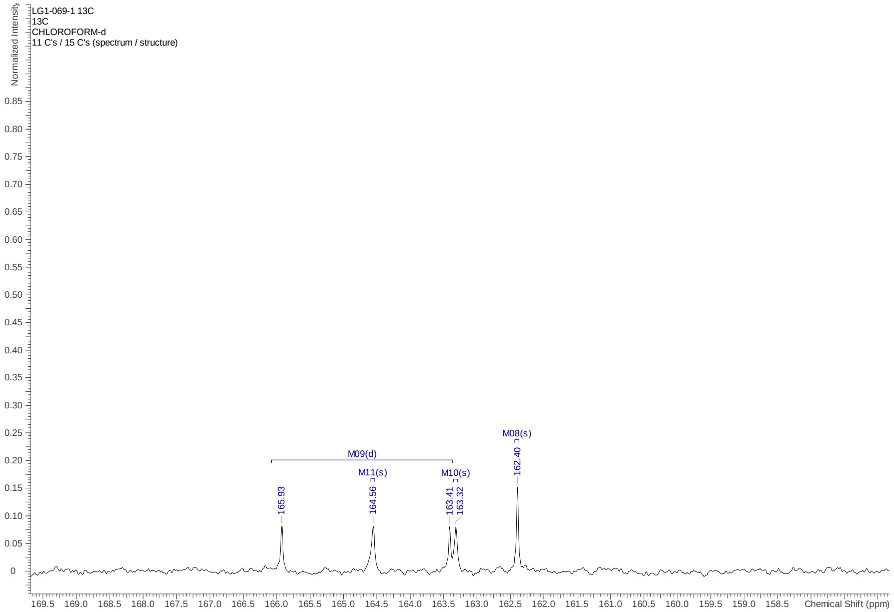
LG1-069-1 13C
13C
CHLOROFORM-d
11 C's / 15 C's (spectrum / structure)
Normalized Intensity
M09(d)
M11(s)
M10(s)
M08(s)
165.93
164.56
163.41
163.32
162.40
Chemical Shift (ppm)

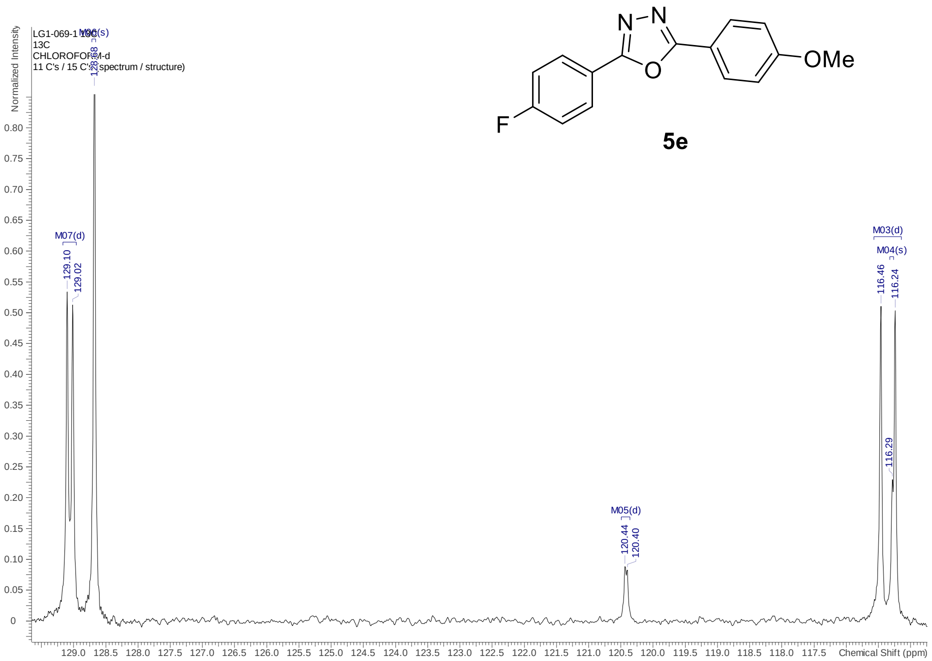
LG1-069-1 13C
13C
CHLOROFORM-d
11 C's / 15 C's (spectrum / structure)
Normalized Intensity
M07(d)
129.10
129.02
M03(d)
M04(s)
116.46
116.24
116.29
M05(d)
120.44
120.40
Chemical Shift (ppm)
N
N
O
F
OMe

5e

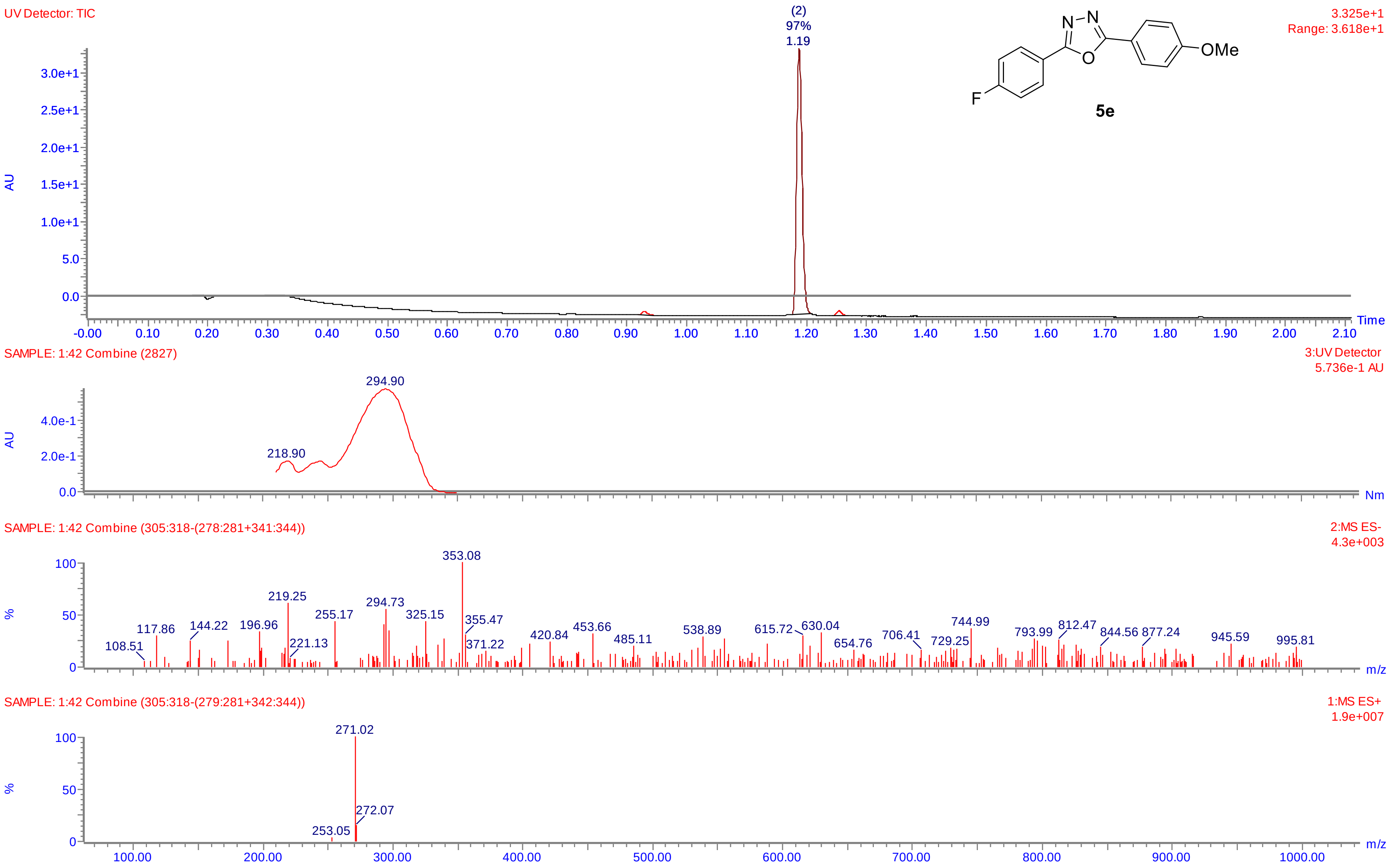
UV Detector: TIC
(2)
97%
1.19
3.325e+1
Range: 3.618e+1
AU
Time
SAMPLE: 1:42 Combine (2827)
3:UV Detector
5.736e-1 AU
294.90
218.90
Nm
SAMPLE: 1:42 Combine (305:318-(278:281+341:344))
2:MS ES-
4.3e+003
353.08
219.25
294.73
325.15
255.17
355.47
371.22
SAMPLE: 1:42 Combine (305:318-(279:281+342:344))
1:MS ES+
1.9e+007
271.02
272.07
253.05
m/z

5e

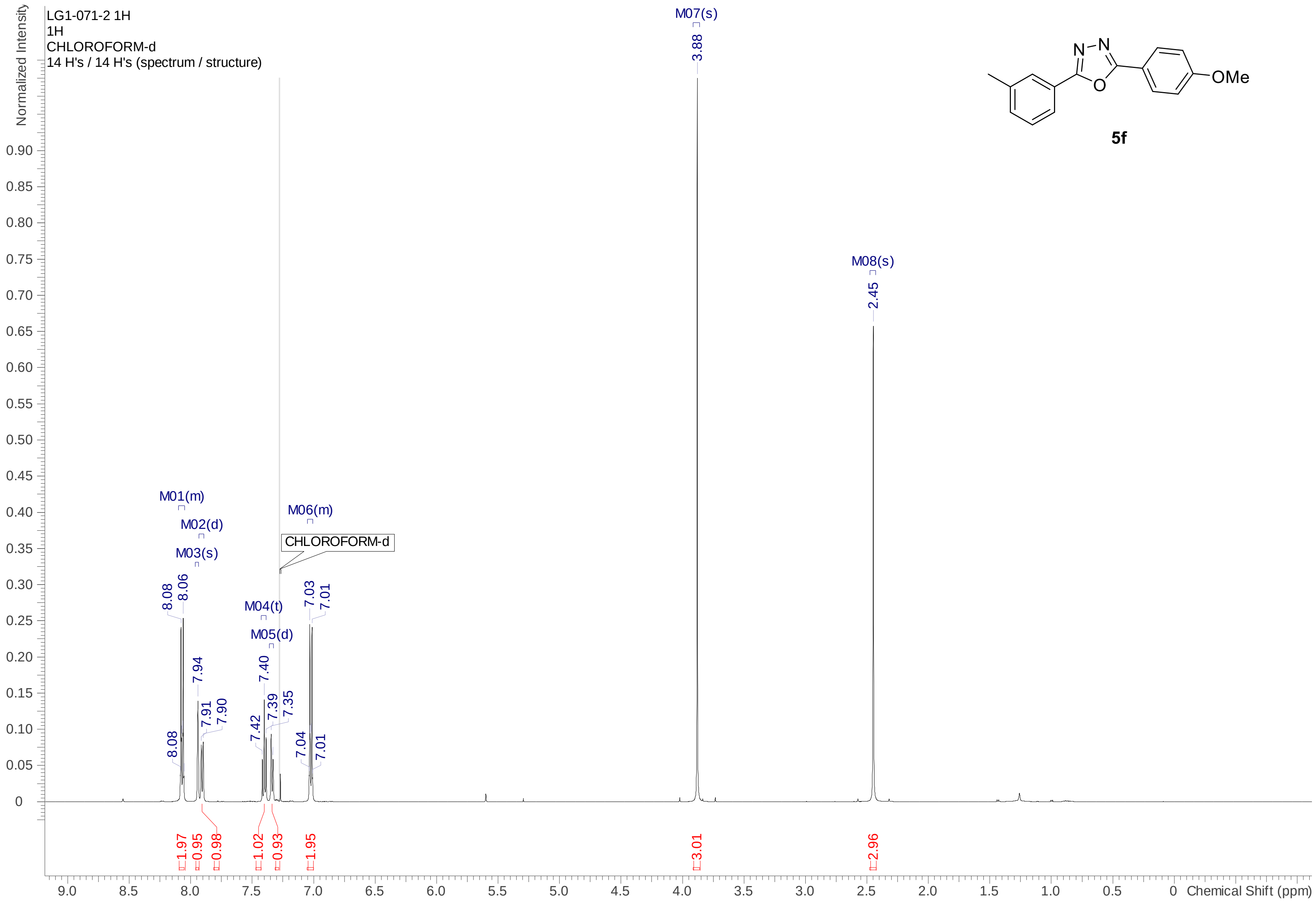
LG1-071-2 1H
1H
CHLOROFORM-d
14 H's / 14 H's (spectrum / structure)
Normalized Intensity
Chemical Shift (ppm)
M01(m)
M02(d)
M03(s)
M04(t)
M05(d)
M06(m)
M07(s)
M08(s)
CHLOROFORM-d
N
N
O
OMe
5f

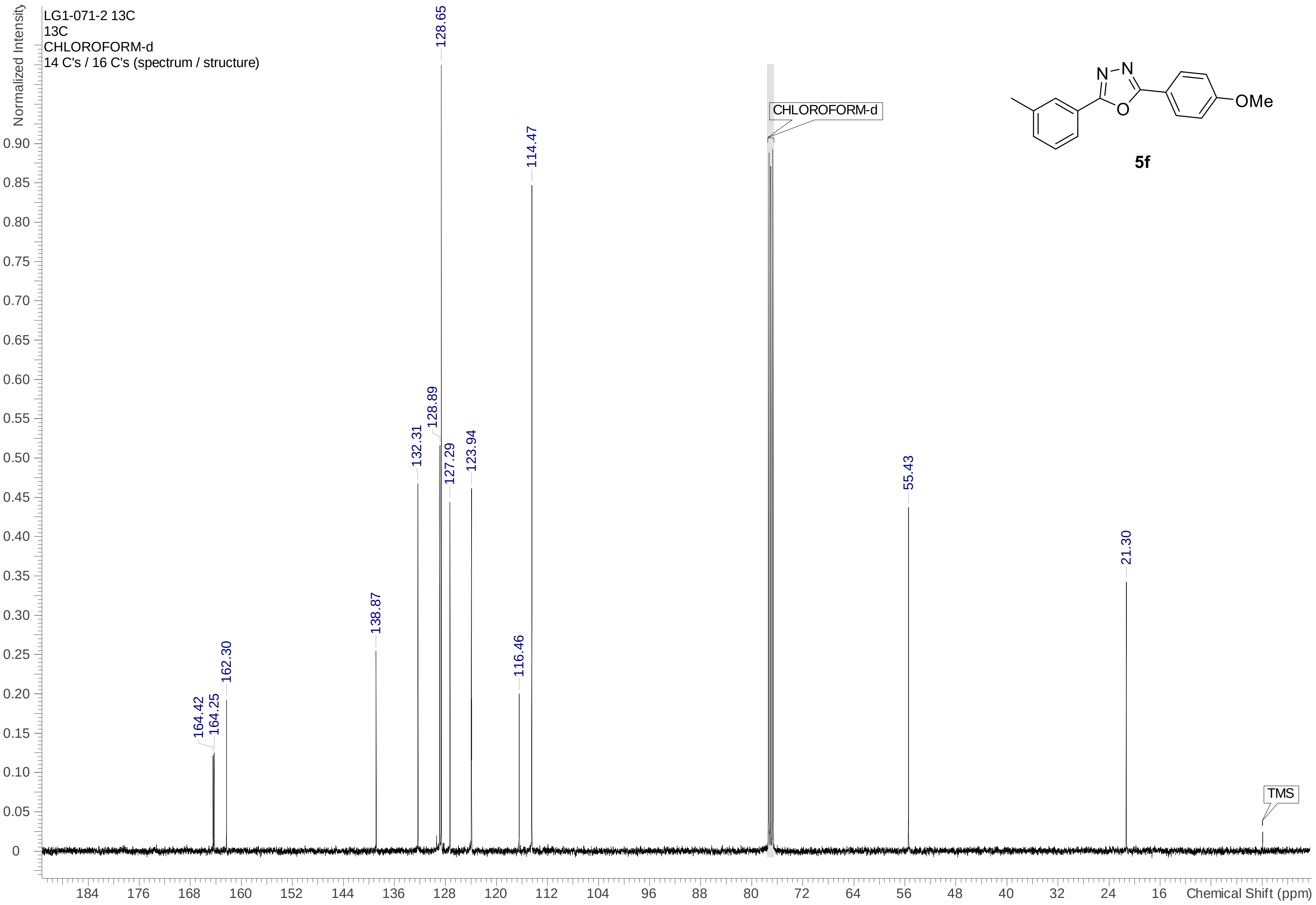
LG1-071-2 13C
13C
CHLOROFORM-d
14 C's / 16 C's (spectrum / structure)
Normalized Intensity
128.65
114.47
128.89
132.31
127.29
123.94
138.87
162.30
164.42
164.25
116.46
55.43
21.30
CHLOROFORM-d
TMS
OMe
N
N
O
5f
0.90
0.85
0.80
0.75
0.70
0.65
0.60
0.55
0.50
0.45
0.40
0.35
0.30
0.25
0.20
0.15
0.10
0.05
0
184
176
168
160
152
144
136
128
120
112
104
96
88
80
72
64
56
48
40
32
24
16
Chemical Shift (ppm)

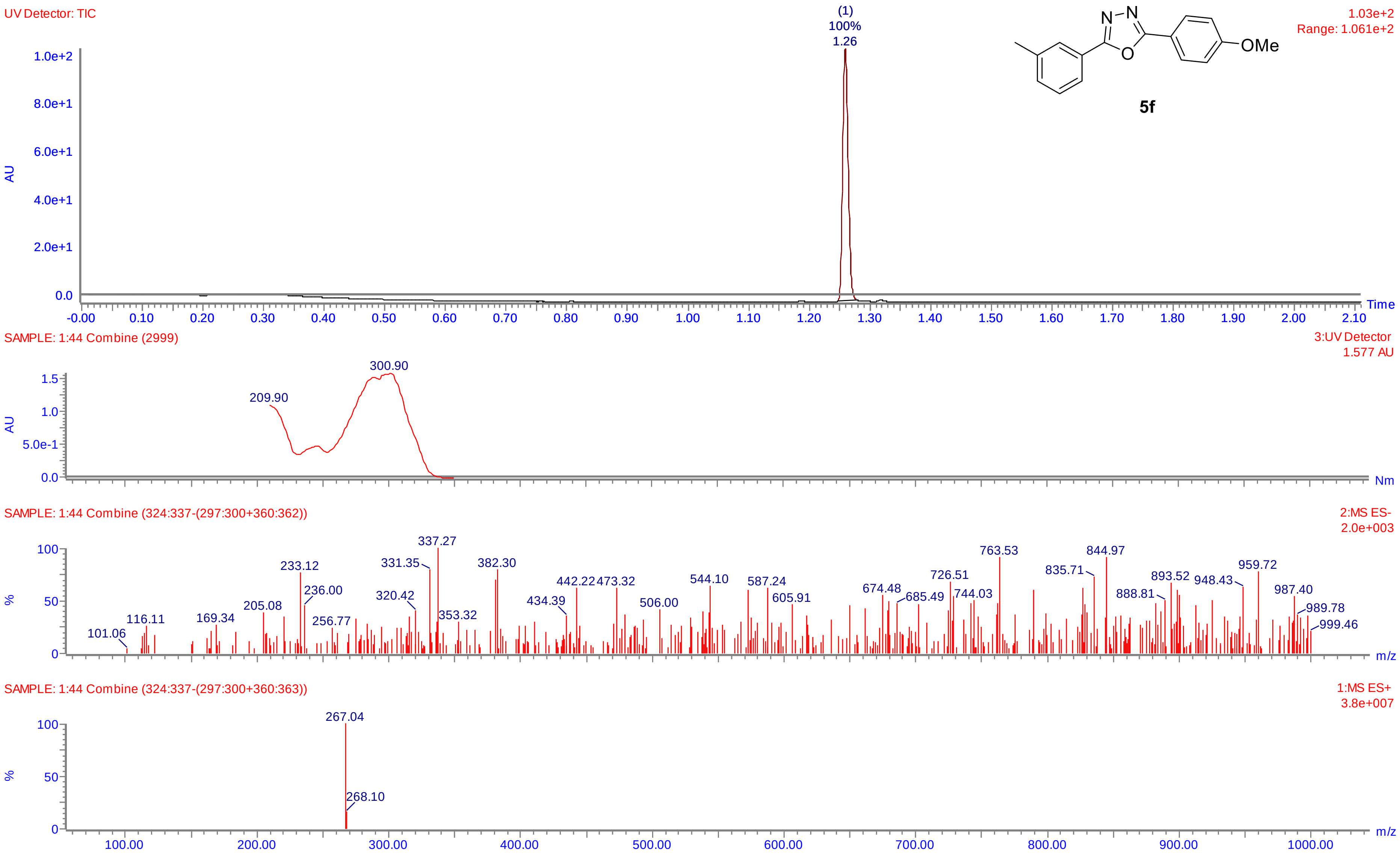
UV Detector: TIC
1.03e+2
Range: 1.061e+2
(1)
100%
1.26
N–N
O
OMe
5f
AU
Time
SAMPLE: 1:44 Combine (2999)
3:UV Detector
1.577 AU
209.90
300.90
Nm
SAMPLE: 1:44 Combine (324:337-(297:300+360:362))
2:MS ES-
2.0e+003
101.06
116.11
169.34
205.08
233.12
236.00
256.77
320.42
331.35
337.27
353.32
382.30
434.39
442.22
473.32
506.00
544.10
587.24
605.91
674.48
685.49
726.51
744.03
763.53
835.71
844.97
888.81
893.52
948.43
959.72
987.40
989.78
999.46
m/z
SAMPLE: 1:44 Combine (324:337-(297:300+360:363))
1:MS ES+
3.8e+007
267.04
268.10
%
m/z

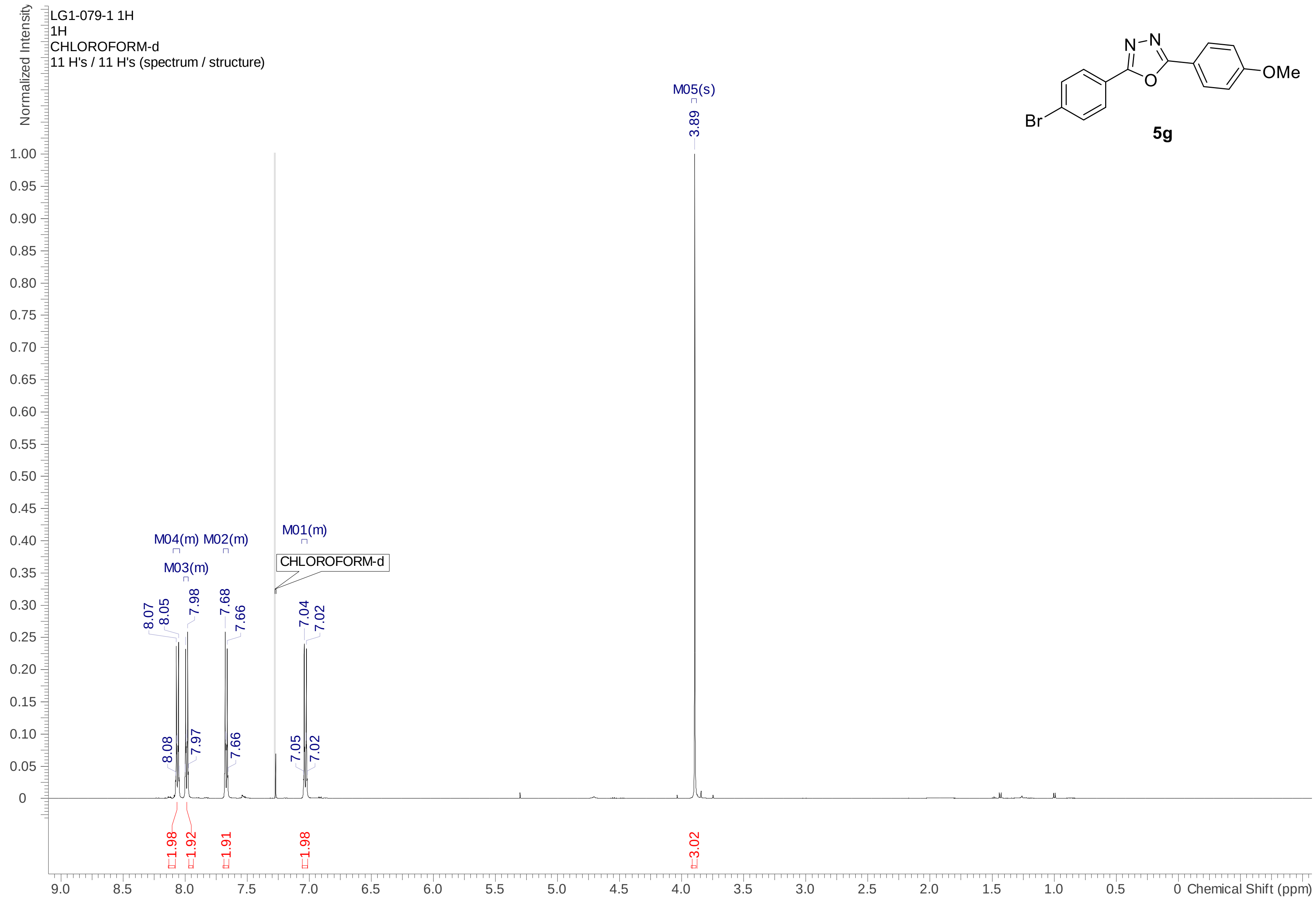

LG1-079-1 1H
1H
CHLOROFORM-d
11 H's / 11 H's (spectrum / structure)
Normalized Intensity
M05(s)
3.89
M04(m) M02(m)
M03(m)
M01(m)
CHLOROFORM-d
8.07
8.05
7.98
7.68
7.66
7.04
7.02
8.08
7.97
7.66
7.05
7.02
1.98
1.92
1.91
1.98
3.02
Chemical Shift (ppm)
Br
N
N
O
OMe

5g

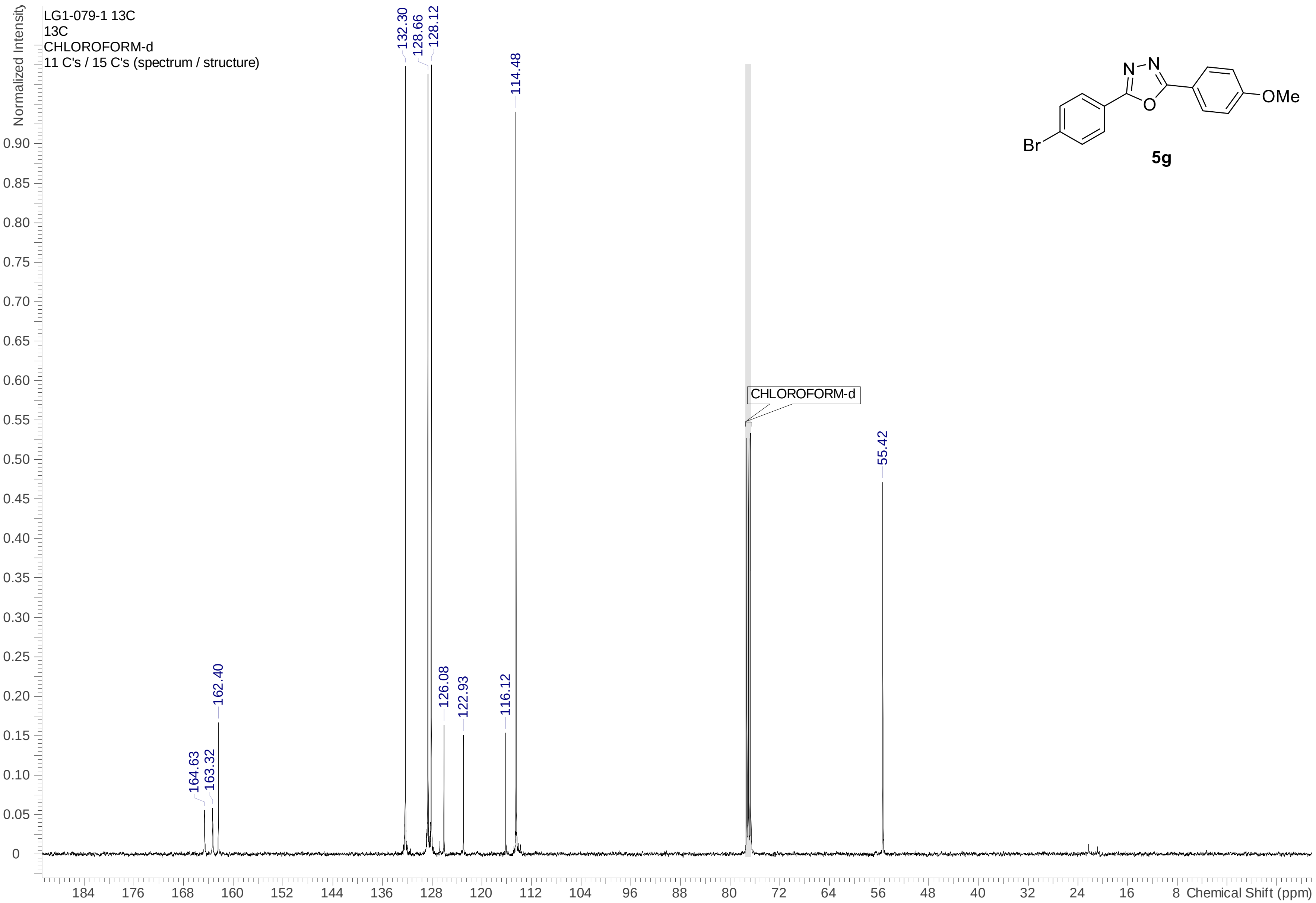
LG1-079-1 13C
13C
CHLOROFORM-d
11 C's / 15 C's (spectrum / structure)
Normalized Intensity
Chemical Shift (ppm)
164.63
163.32
162.40
132.30
128.66
128.12
126.08
122.93
116.12
114.48
CHLOROFORM-d
55.42
Br
OMe
N
N
O

5g

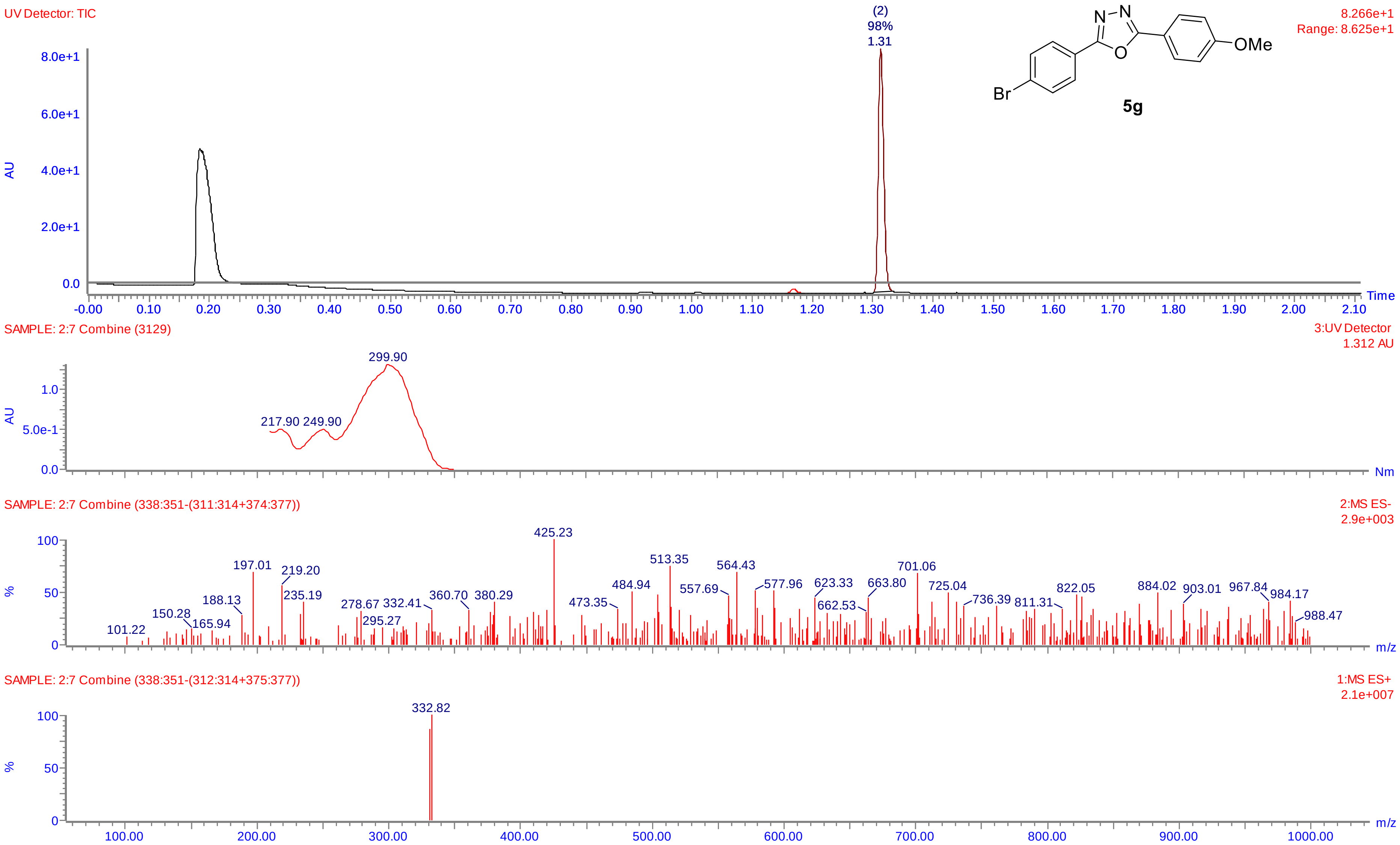
N–N
O
Br
OMe
5g
UV Detector: TIC
8.266e+1
Range: 8.625e+1
(2)
98%
1.31
AU
8.0e+1
6.0e+1
4.0e+1
2.0e+1
0.0
Time
-0.00 0.10 0.20 0.30 0.40 0.50 0.60 0.70 0.80 0.90 1.00 1.10 1.20 1.30 1.40 1.50 1.60 1.70 1.80 1.90 2.00 2.10
SAMPLE: 2:7 Combine (3129)
3:UV Detector
1.312 AU
299.90
217.90 249.90
1.0
5.0e-1
0.0
Nm
SAMPLE: 2:7 Combine (338:351-(311:314+374:377))
2:MS ES-
2.9e+003
425.23
513.35
564.43
701.06
197.01
219.20
484.94
557.69
577.96
623.33
663.80
725.04
822.05
884.02
903.01
967.84
984.17
235.19
188.13
360.70
380.29
473.35
662.53
736.39
811.31
988.47
150.28
278.67
332.41
165.94
295.27
101.22
100
50
0
%
m/z
SAMPLE: 2:7 Combine (338:351-(312:314+375:377))
1:MS ES+
2.1e+007
332.82
100.00 200.00 300.00 400.00 500.00 600.00 700.00 800.00 900.00 1000.00

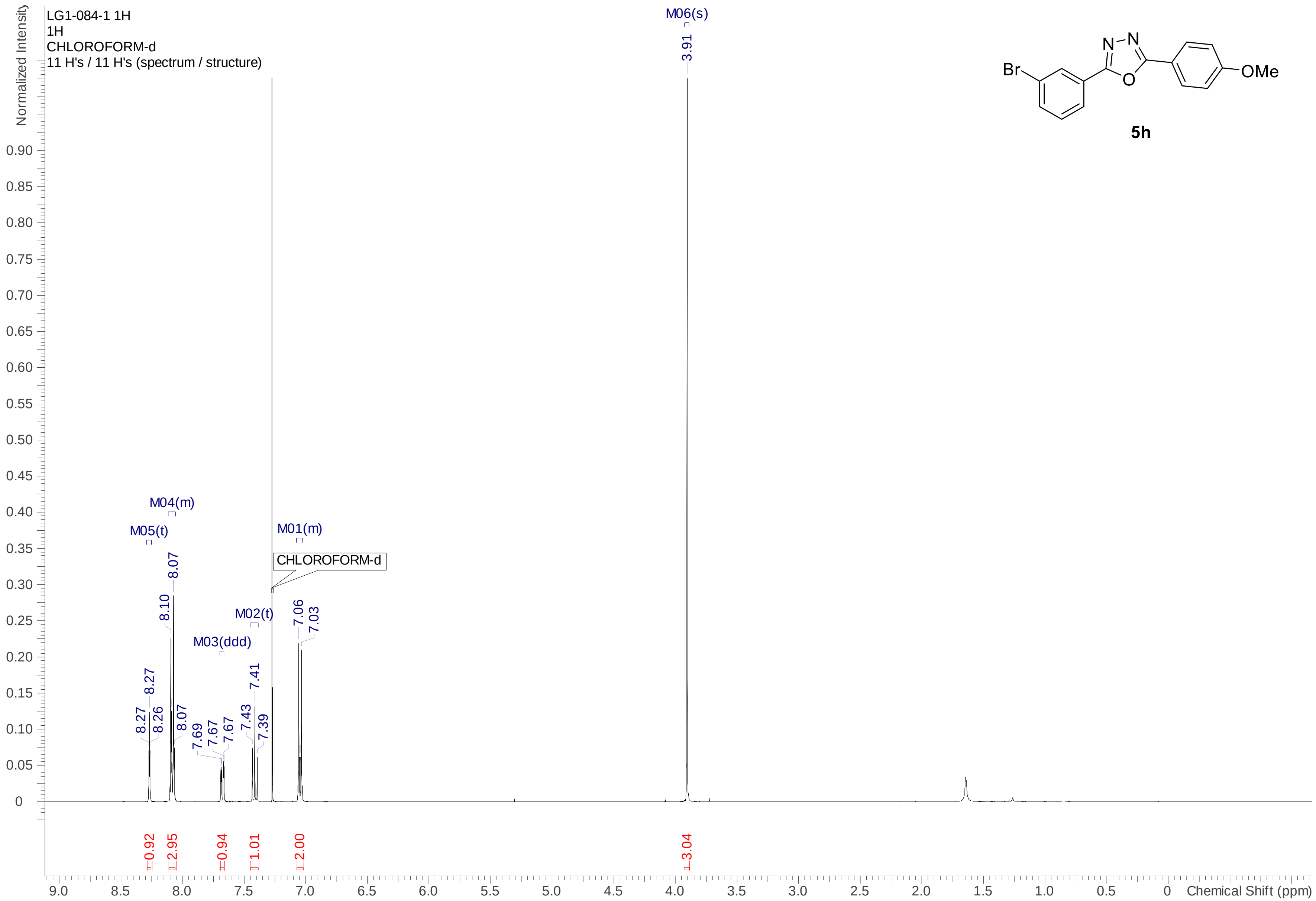

LG1-084-1 1H
1H
CHLOROFORM-d
11 H's / 11 H's (spectrum / structure)
Normalized Intensity
M06(s)
3.91
M04(m)
M05(t)
M01(m)
M02(t)
M03(ddd)
CHLOROFORM-d
8.27
8.27
8.26
8.10
8.07
8.07
7.69
7.67
7.67
7.43
7.41
7.39
7.06
7.03
0.92
2.95
0.94
1.01
2.00
3.04
Chemical Shift (ppm)
Br
N
N
O
OMe

5h

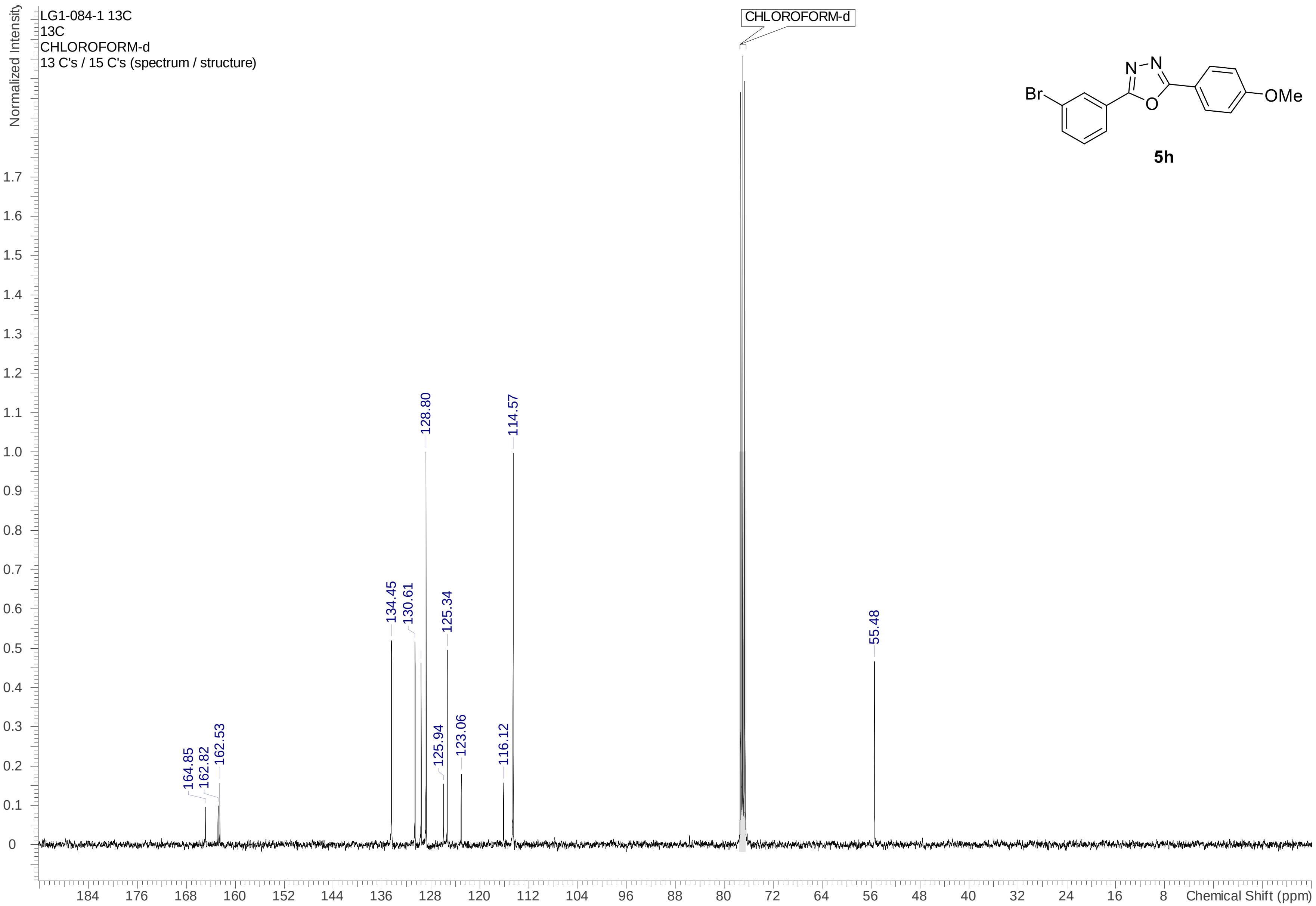
LG1-084-1 13C
13C
CHLOROFORM-d
13 C's / 15 C's (spectrum / structure)
CHLOROFORM-d
Normalized Intensity
Chemical Shift (ppm)
164.85
162.82
162.53
134.45
130.61
128.80
125.94
125.34
123.06
116.12
114.57
55.48
Br
N
N
O
OMe
5h

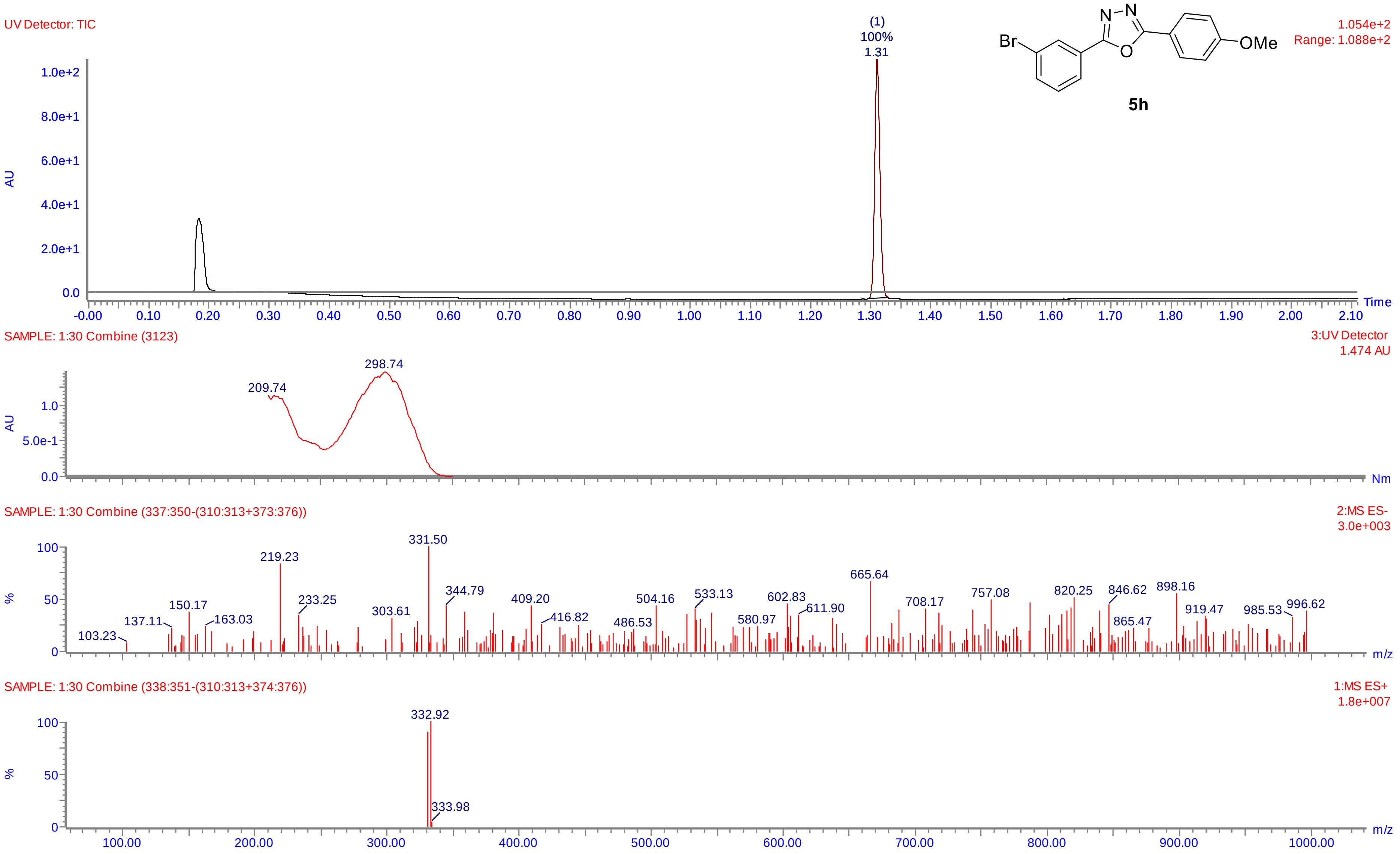
UV Detector: TIC
(1)
100%
1.31
1.054e+2
Range: 1.088e+2
Br
N
N
O
OMe
5h
AU
1.0e+2
8.0e+1
6.0e+1
4.0e+1
2.0e+1
0.0
Time
-0.00
0.10
0.20
0.30
0.40
0.50
0.60
0.70
0.80
0.90
1.00
1.10
1.20
1.30
1.40
1.50
1.60
1.70
1.80
1.90
2.00
2.10
SAMPLE: 1:30 Combine (3123)
3:UV Detector
1.474 AU
209.74
298.74
1.0
5.0e-1
0.0
Nm
SAMPLE: 1:30 Combine (337:350-(310:313+373:376))
2:MS ES-
3.0e+003
%
100
50
0
331.50
219.23
344.79
150.17
233.25
409.20
504.16
533.13
602.83
665.64
708.17
757.08
820.25
846.62
898.16
137.11
163.03
303.61
416.82
486.53
580.97
611.90
865.47
919.47
985.53
996.62
103.23
m/z
SAMPLE: 1:30 Combine (338:351-(310:313+374:376))
1:MS ES+
1.8e+007
332.92
333.98
100.00
200.00
300.00
400.00
500.00
600.00
700.00
800.00
900.00
1000.00

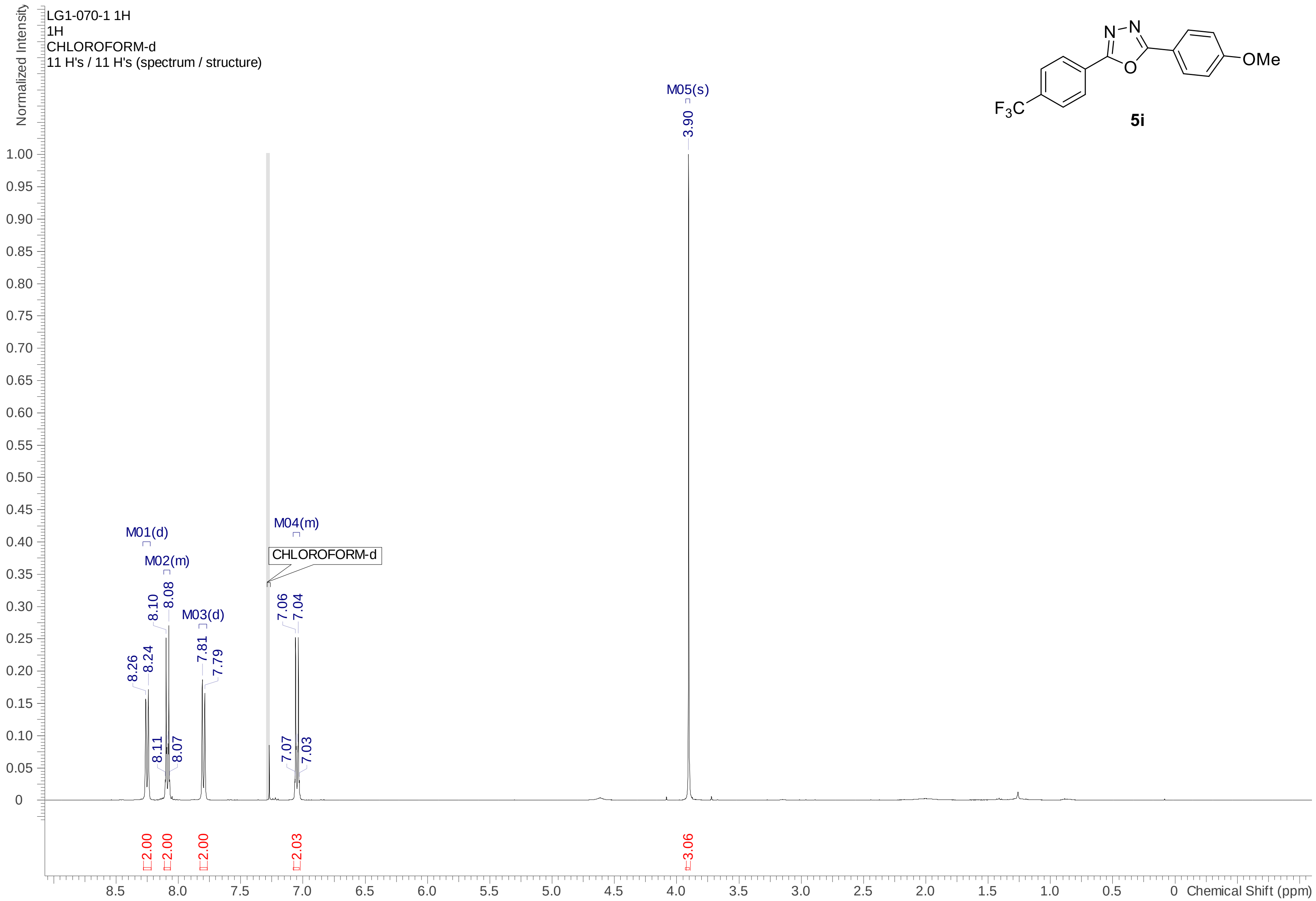

LG1-070-1 1H
1H
CHLOROFORM-d
11 H's / 11 H's (spectrum / structure)
Normalized Intensity
M01(d)
M02(m)
M03(d)
M04(m)
M05(s)
CHLOROFORM-d
8.26
8.24
8.10
8.08
8.11
8.07
7.81
7.79
7.06
7.04
7.07
7.03
3.90
2.00
2.00
2.00
2.03
3.06
Chemical Shift (ppm)
N
N
O
OMe
F_3C
5i

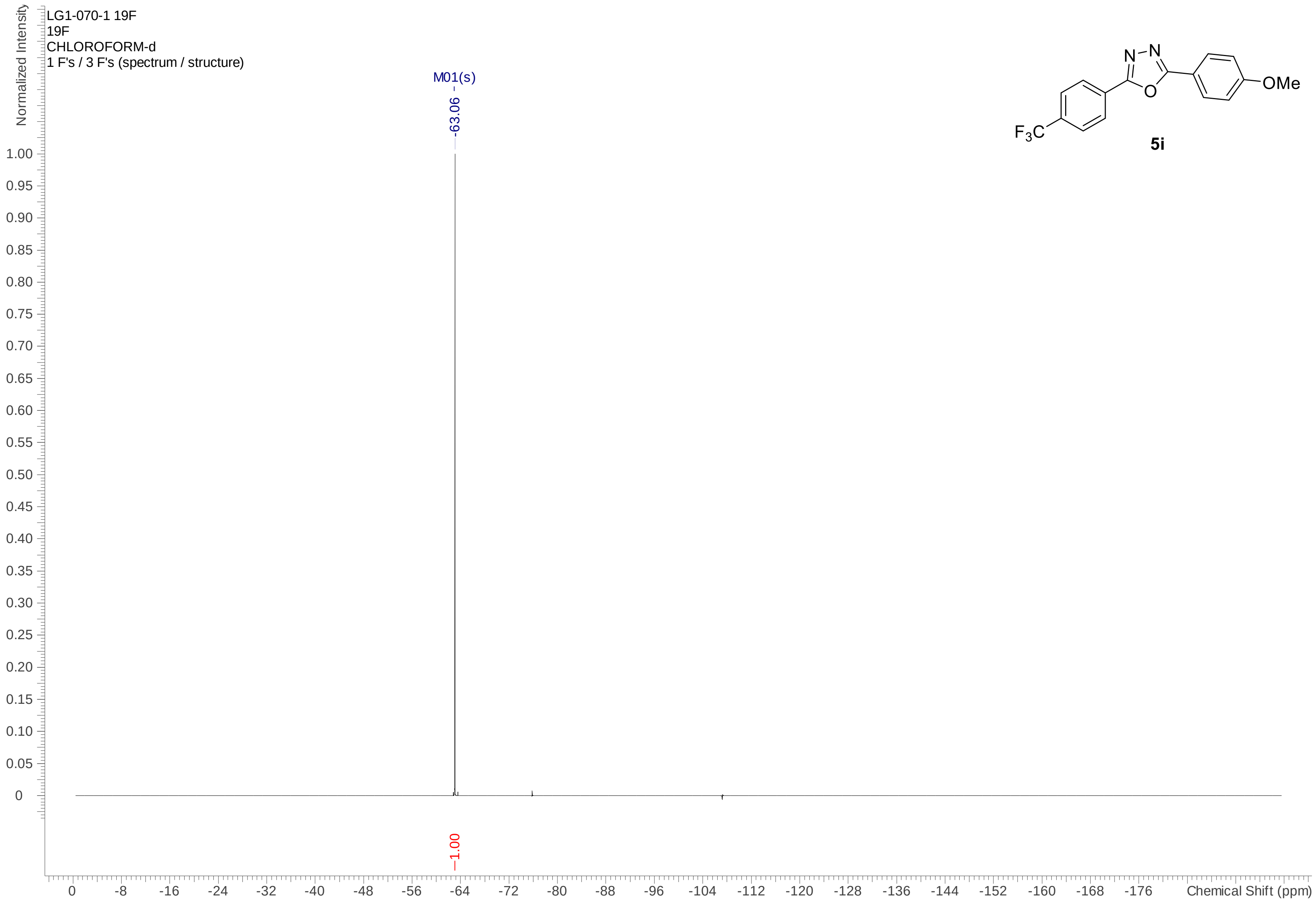
LG1-070-1 19F
19F
CHLOROFORM-d
1 F's / 3 F's (spectrum / structure)
M01(s)
-63.06
1.00
Normalized Intensity
Chemical Shift (ppm)
N
N
O
OMe
F_3C

5i

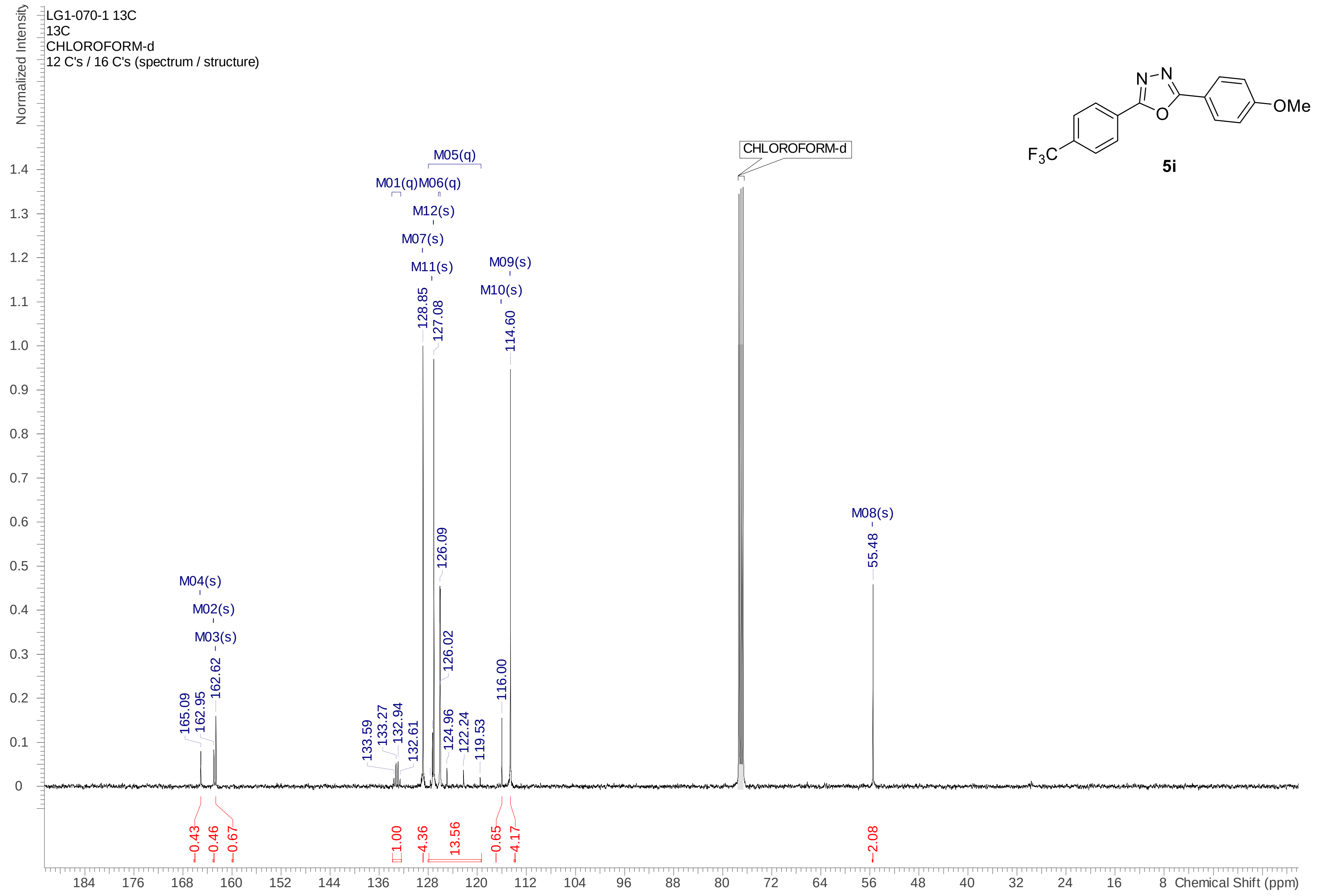
LG1-070-1 13C
13C
CHLOROFORM-d
12 C's / 16 C's (spectrum / structure)
Normalized Intensity
Chemical Shift (ppm)
CHLOROFORM-d
M01(q) M06(q) M05(q) M12(s) M07(s) M11(s) M09(s) M10(s) M04(s) M02(s) M03(s) M08(s)
165.09
162.95
162.62
133.59
133.27
132.94
132.61
128.85
127.08
126.09
126.02
124.96
122.24
119.53
116.00
114.60
55.48
0.43
0.46
0.67
1.00
4.36
13.56
0.65
4.17
2.08
N
N
O
F3C
OMe
5i

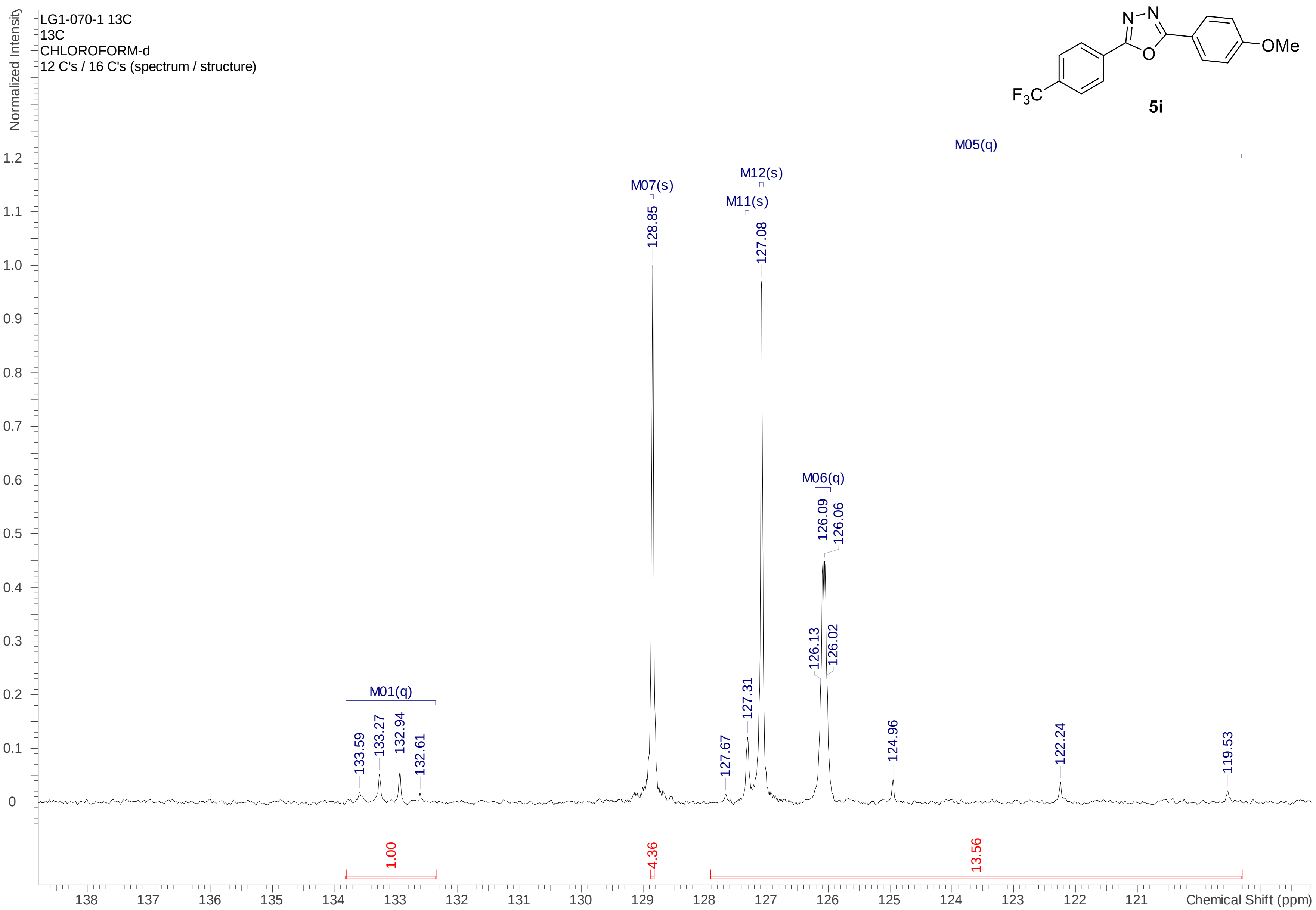
LG1-070-1 13C
13C
CHLOROFORM-d
12 C's / 16 C's (spectrum / structure)
Normalized Intensity
5i
OMe
F3C
M05(q)
M12(s)
M07(s)
M11(s)
128.85
127.08
M06(q)
126.09
126.06
126.13
126.02
M01(q)
133.59
133.27
132.94
132.61
127.67
127.31
124.96
122.24
119.53
1.00
4.36
13.56
Chemical Shift (ppm)

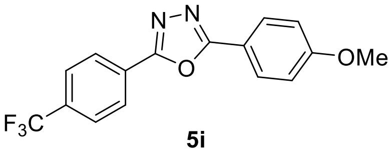
N
N
O
OMe
F3C

5i

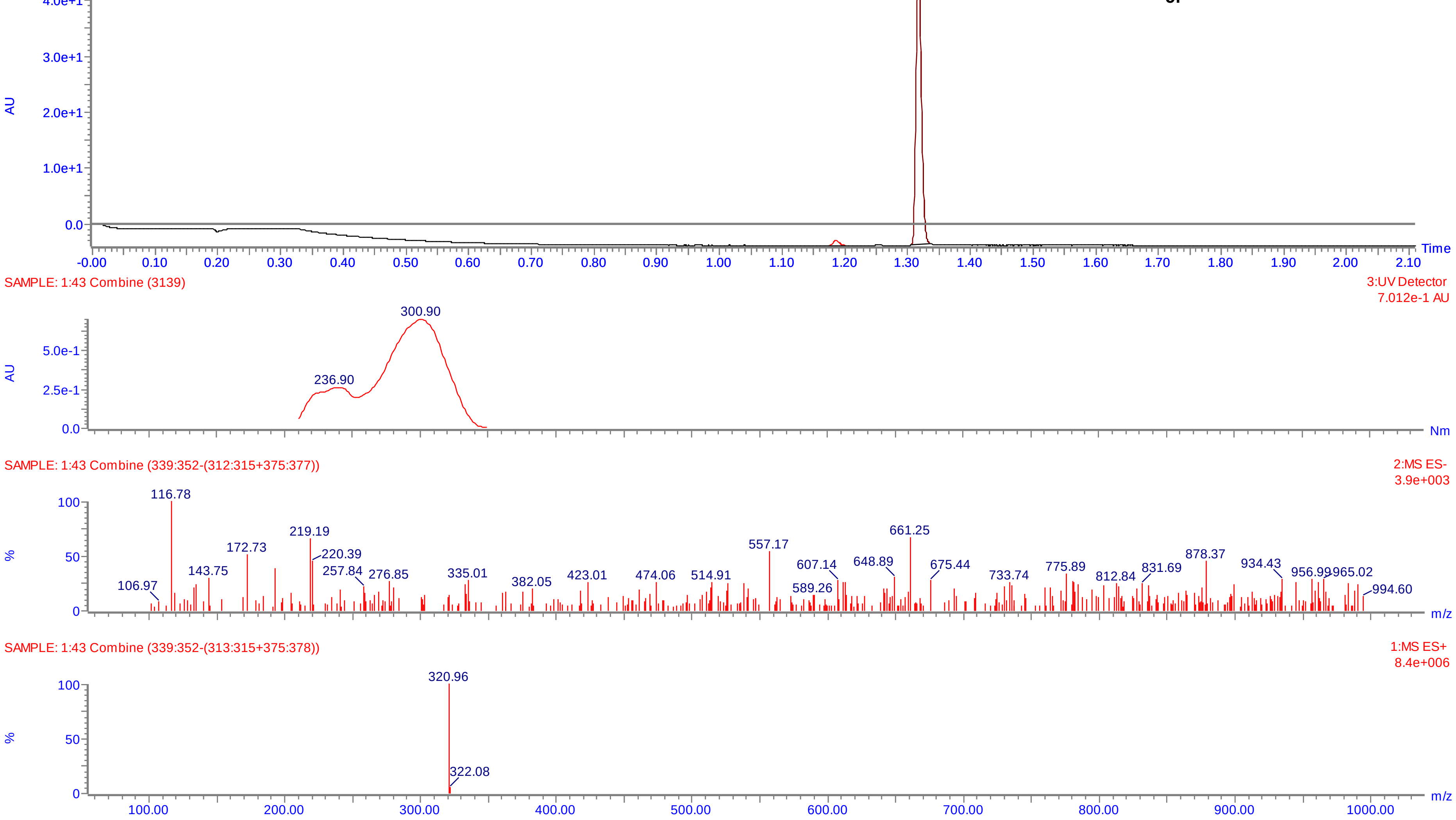
UV Detector: TIC
(2)
98%
1.32
4.557e+1
Range: 4.945e+1
AU
4.0e+1
3.0e+1
2.0e+1
1.0e+1
0.0
Time
-0.00 0.10 0.20 0.30 0.40 0.50 0.60 0.70 0.80 0.90 1.00 1.10 1.20 1.30 1.40 1.50 1.60 1.70 1.80 1.90 2.00 2.10
SAMPLE: 1:43 Combine (3139)
3:UV Detector
7.012e-1 AU
300.90
236.90
AU
5.0e-1
2.5e-1
0.0
Nm
SAMPLE: 1:43 Combine (339:352-(312:315+375:377))
2:MS ES-
3.9e+003
116.78
219.19
172.73
220.39
143.75
257.84
276.85
106.97
335.01
382.05
423.01
474.06
514.91
557.17
589.26
607.14
648.89
661.25
675.44
733.74
775.89
812.84
831.69
878.37
934.43
956.99
965.02
994.60
%
100
50
0
m/z
SAMPLE: 1:43 Combine (339:352-(313:315+375:378))
1:MS ES+
8.4e+006
320.96
322.08
%
100
50
0
m/z
100.00 200.00 300.00 400.00 500.00 600.00 700.00 800.00 900.00 1000.00

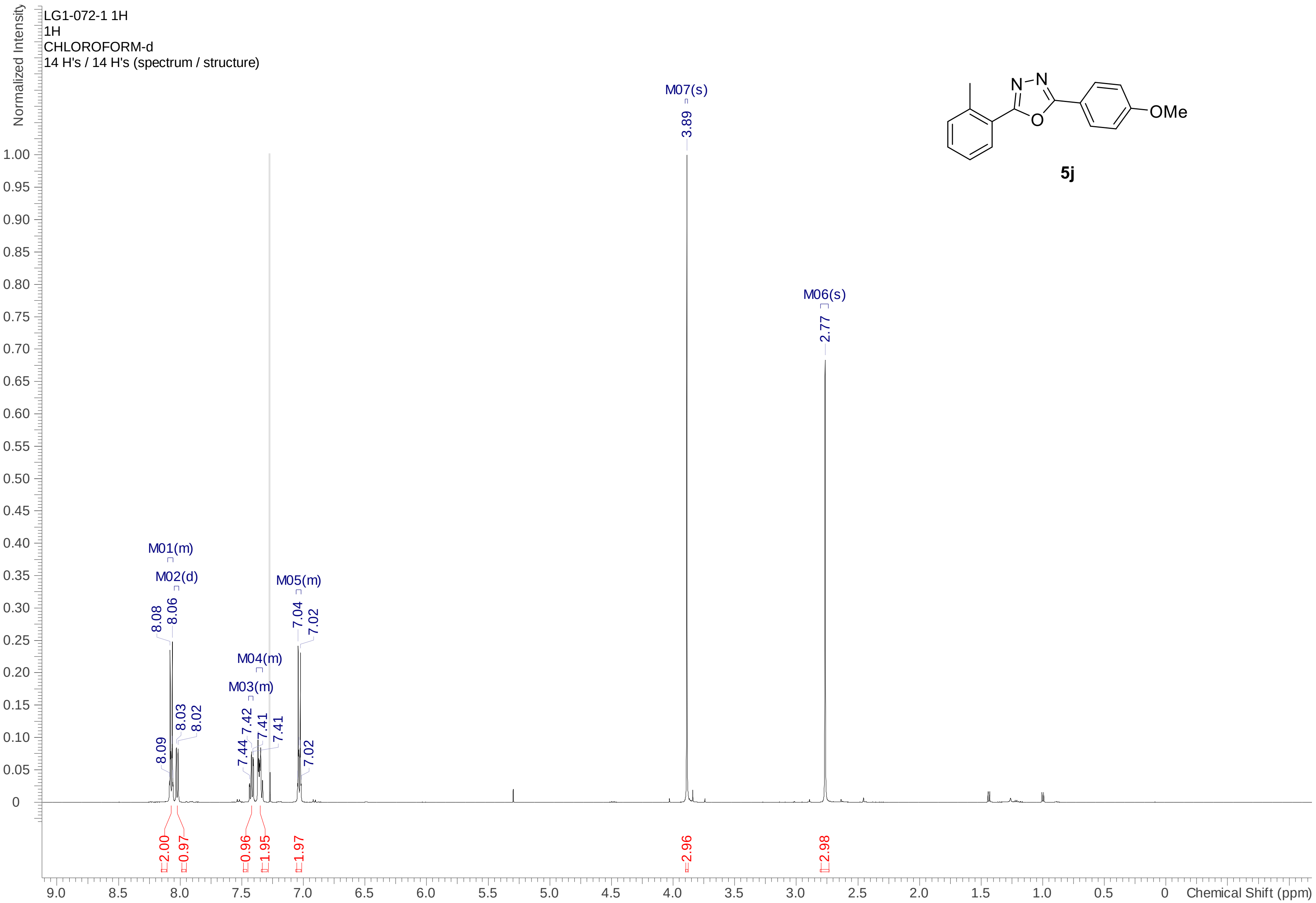
LG1-072-1 1H
1H
CHLOROFORM-d
14 H's / 14 H's (spectrum / structure)
Normalized Intensity
M01(m)
M02(d)
M03(m)
M04(m)
M05(m)
M06(s)
M07(s)
8.09
8.08
8.06
8.03
8.02
7.44
7.42
7.41
7.41
7.04
7.02
7.02
3.89
2.77
2.00
0.97
0.96
1.95
1.97
2.96
2.98
N
N
O
OMe
5j
Chemical Shift (ppm)

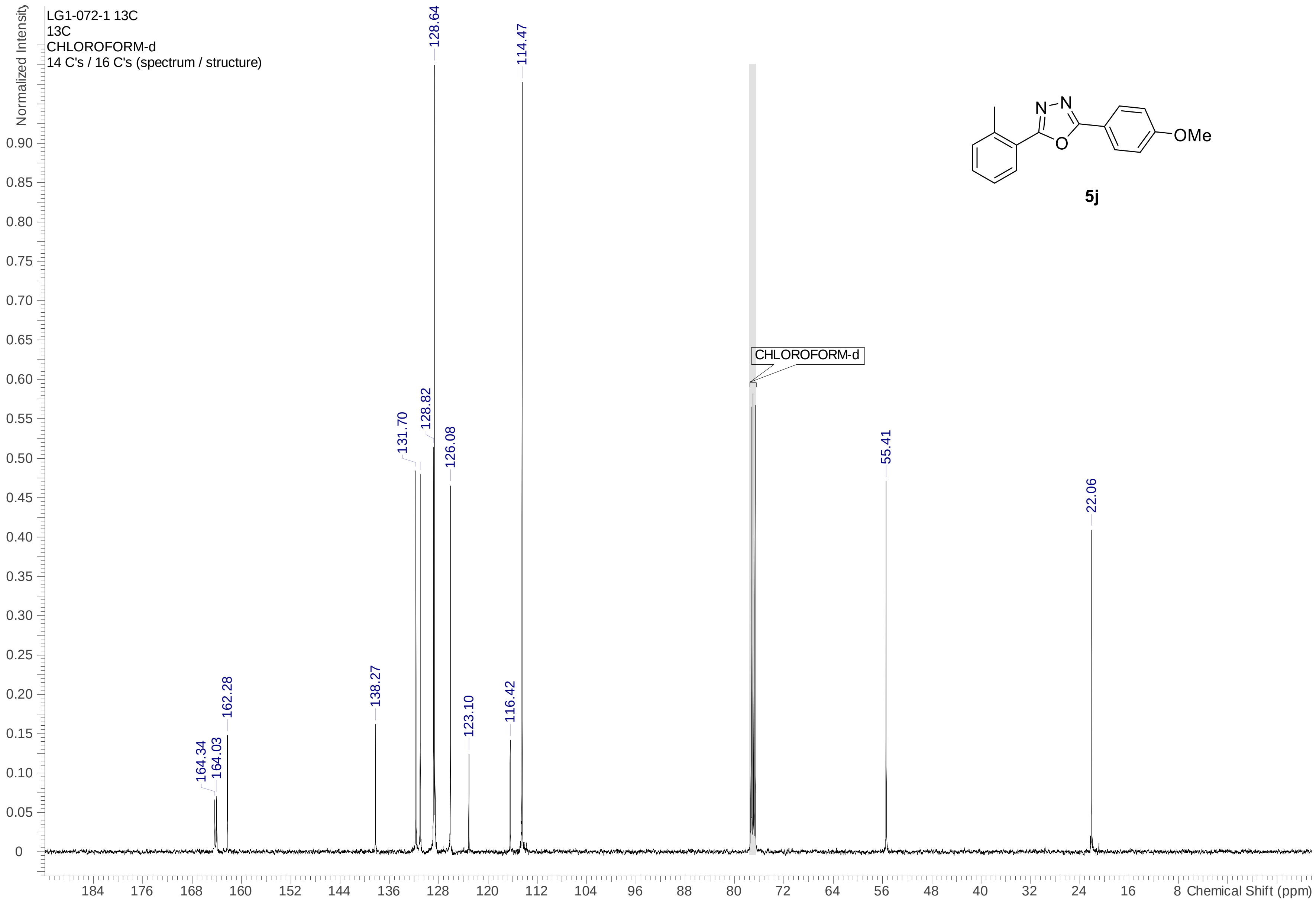
LG1-072-1 13C
13C
CHLOROFORM-d
14 C's / 16 C's (spectrum / structure)
Normalized Intensity
128.64
114.47
131.70
128.82
126.08
164.34
164.03
162.28
138.27
123.10
116.42
CHLOROFORM-d
55.41
22.06
Chemical Shift (ppm)
OMe
5j

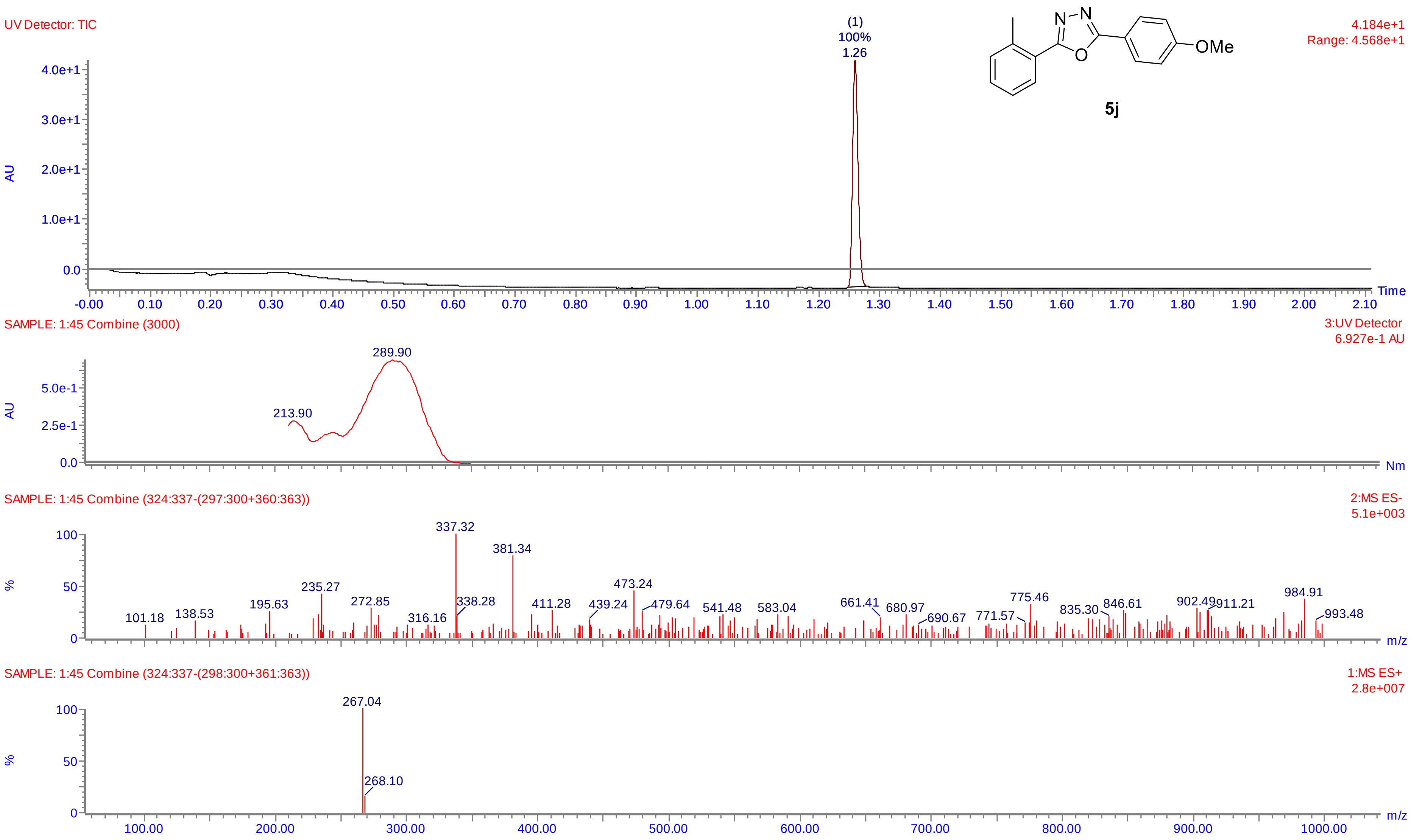

UV Detector: TIC
(1)
100%
1.26
N
N
O
OMe
5j
4.184e+1
Range: 4.568e+1
AU
Time
SAMPLE: 1:45 Combine (3000)
3:UV Detector
6.927e-1 AU
289.90
213.90
Nm
SAMPLE: 1:45 Combine (324:337-(297:300+360:363))
2:MS ES-
5.1e+003
337.32
381.34
473.24
984.91
m/z
SAMPLE: 1:45 Combine (324:337-(298:300+361:363))
1:MS ES+
2.8e+007
267.04
268.10
%
m/z

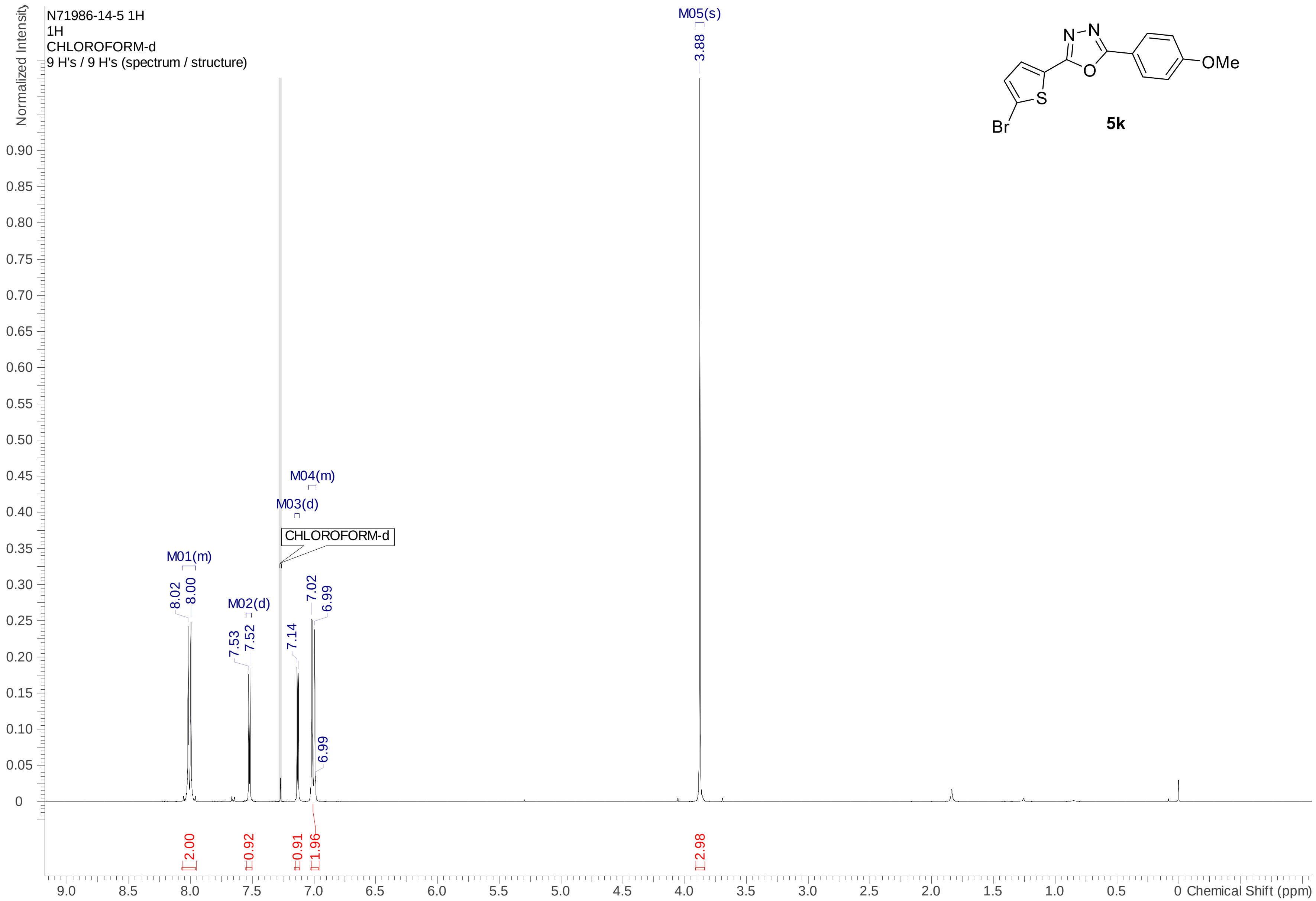

N71986-14-5 1H
1H
CHLOROFORM-d
9 H's / 9 H's (spectrum / structure)
Normalized Intensity
M05(s)
3.88
M04(m)
M03(d)
CHLOROFORM-d
M01(m)
8.02
8.00
M02(d)
7.53
7.52
7.14
7.02
6.99
6.99
2.00
0.92
0.91
1.96
2.98
Chemical Shift (ppm)
N
N
O
S
Br
OMe
5k

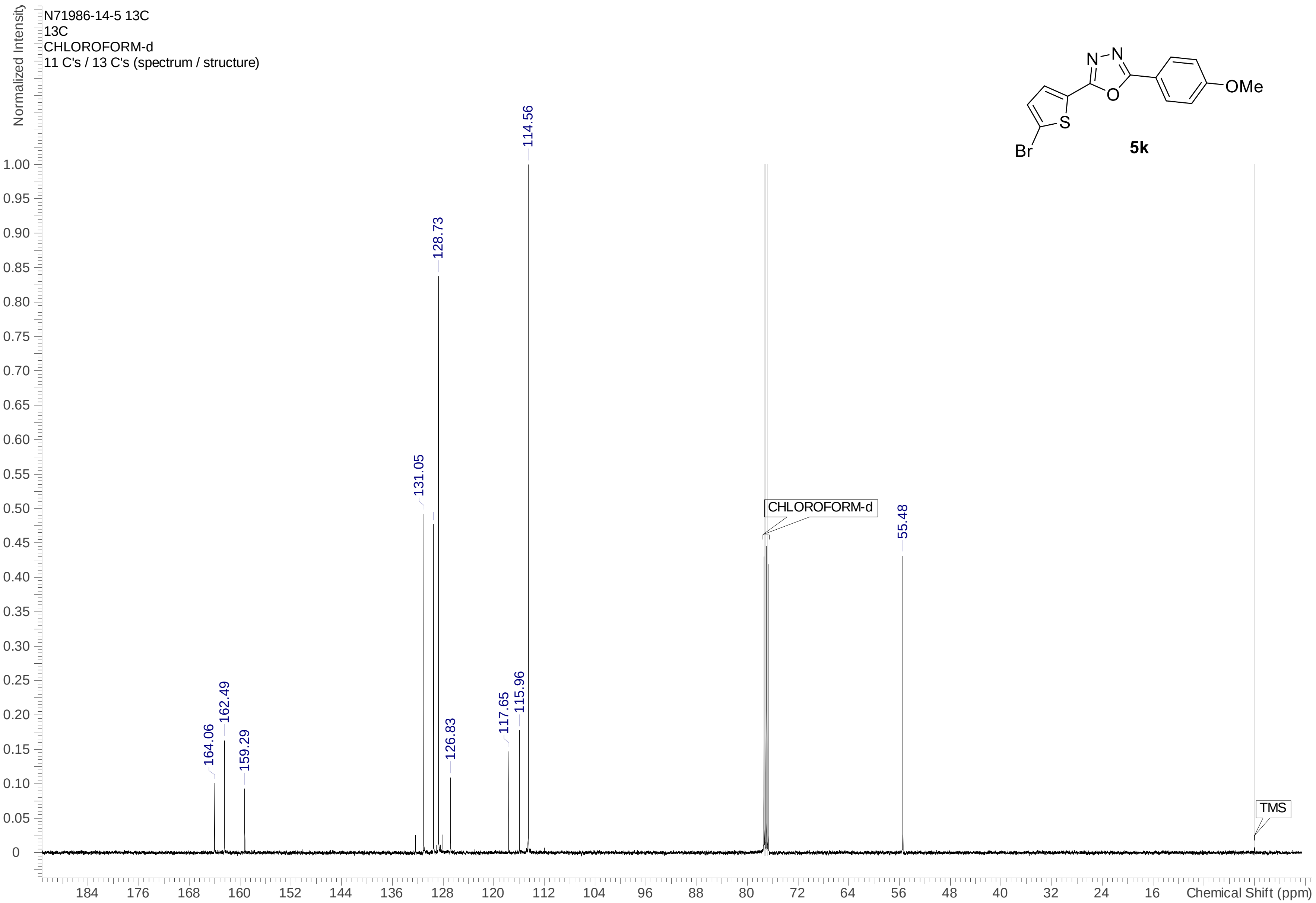
N71986-14-5 13C
13C
CHLOROFORM-d
11 C's / 13 C's (spectrum / structure)
Normalized Intensity
N
N
O
S
Br
OMe
5k
164.06
162.49
159.29
131.05
128.73
126.83
117.65
115.96
114.56
CHLOROFORM-d
55.48
TMS
Chemical Shift (ppm)

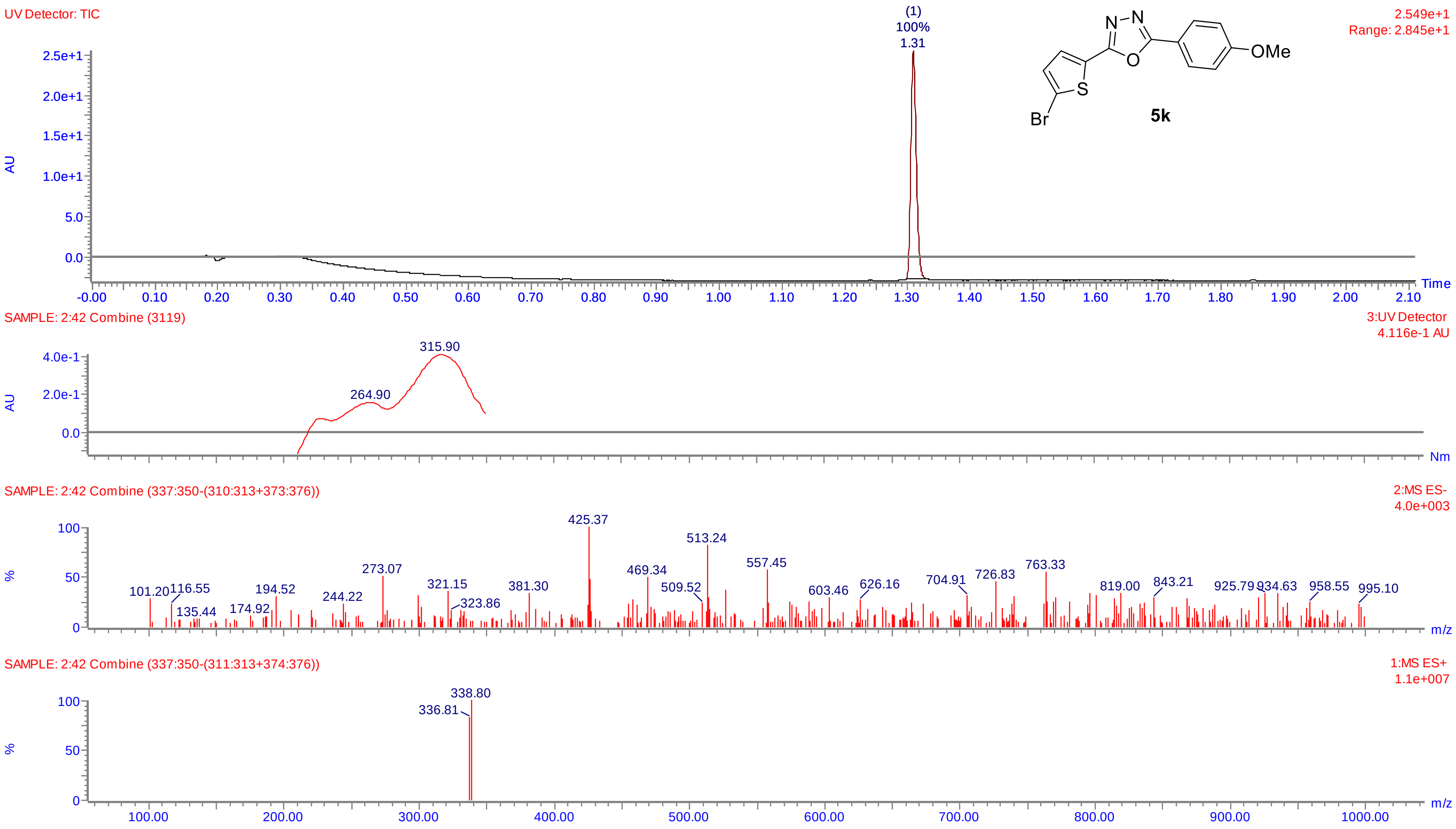
UV Detector: TIC
2.549e+1
Range: 2.845e+1
(1)
100%
1.31
N
N
O
S
Br
OMe
5k
AU
Time
SAMPLE: 2:42 Combine (3119)
3:UV Detector
4.116e-1 AU
315.90
264.90
Nm
SAMPLE: 2:42 Combine (337:350-(310:313+373:376))
2:MS ES-
4.0e+003
425.37
513.24
557.45
469.34
273.07
763.33
726.83
704.91
626.16
603.46
509.52
381.30
321.15
323.86
194.52
244.22
101.20
116.55
135.44
174.92
819.00
843.21
925.79
934.63
958.55
995.10
m/z
SAMPLE: 2:42 Combine (337:350-(311:313+374:376))
1:MS ES+
1.1e+007
338.80
336.81
m/z

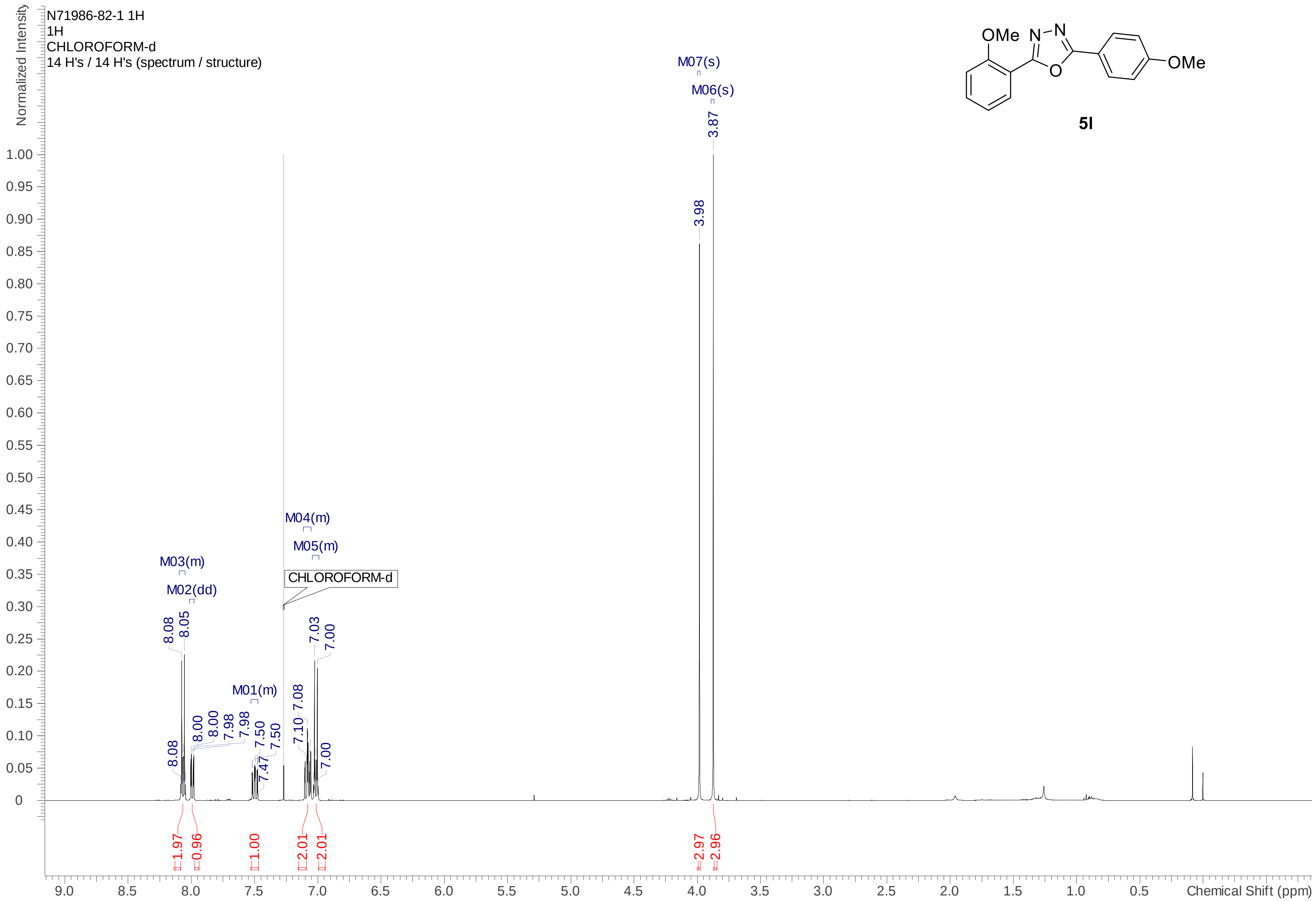
N71986-82-1 1H
1H
CHLOROFORM-d
14 H's / 14 H's (spectrum / structure)
Normalized Intensity
OMe
N
N
O
OMe
5l
M07(s)
M06(s)
3.87
3.98
M03(m)
M02(dd)
M01(m)
M04(m)
M05(m)
CHLOROFORM-d
8.08
8.05
8.08
8.00
8.00
7.98
7.98
7.50
7.50
7.47
7.08
7.10
7.03
7.00
7.00
1.97
0.96
1.00
2.01
2.01
2.97
2.96
Chemical Shift (ppm)

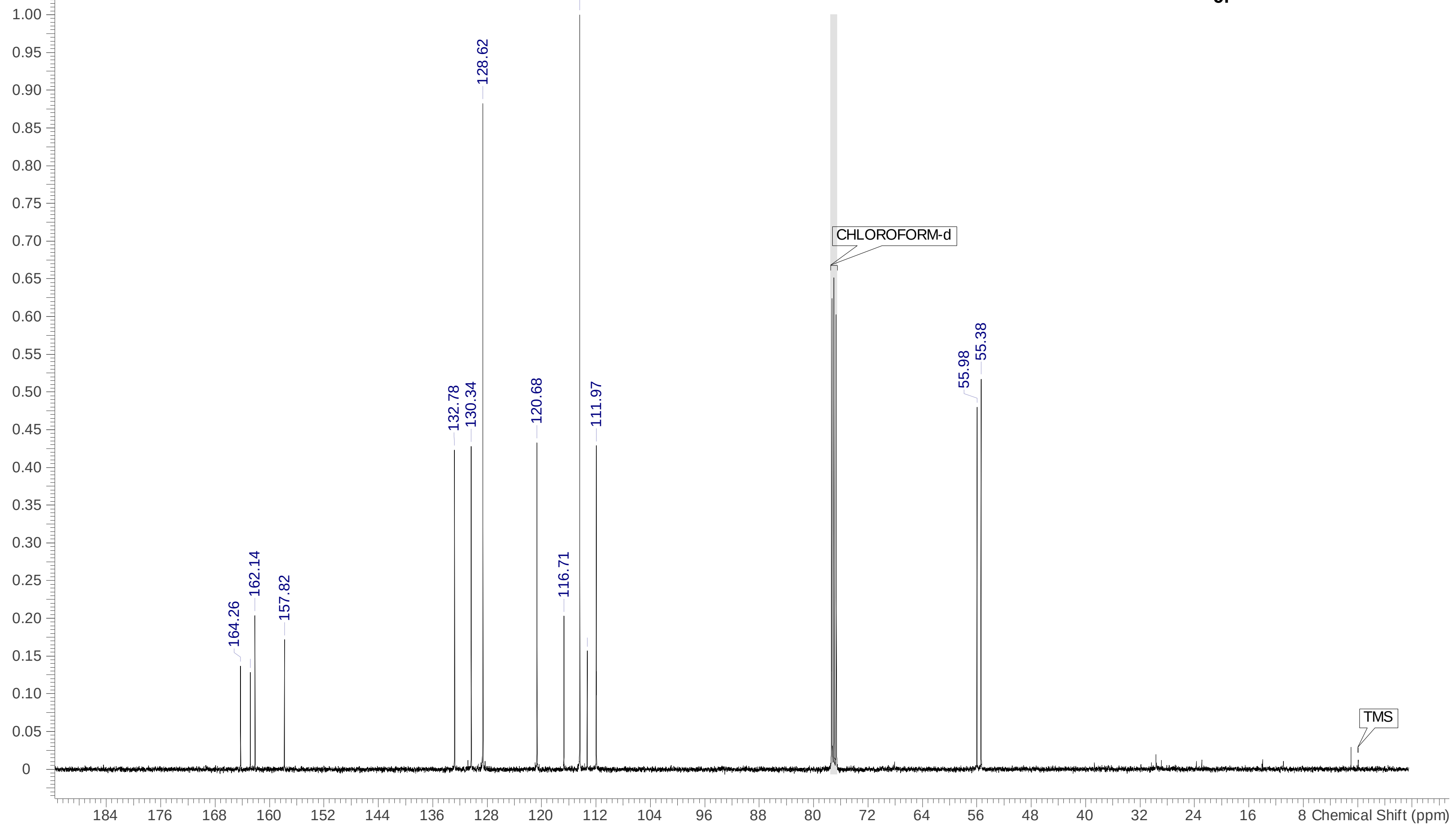
N71986-82-1 13C
13C
CHLOROFORM-d
14 C's / 16 C's (spectrum / structure)
Normalized Intensity
164.26
162.14
157.82
132.78
130.34
128.62
120.68
116.71
114.38
111.97
CHLOROFORM-d
55.98
55.38
TMS
Chemical Shift (ppm)

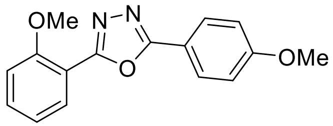
OMe
N–N
O
OMe

5l

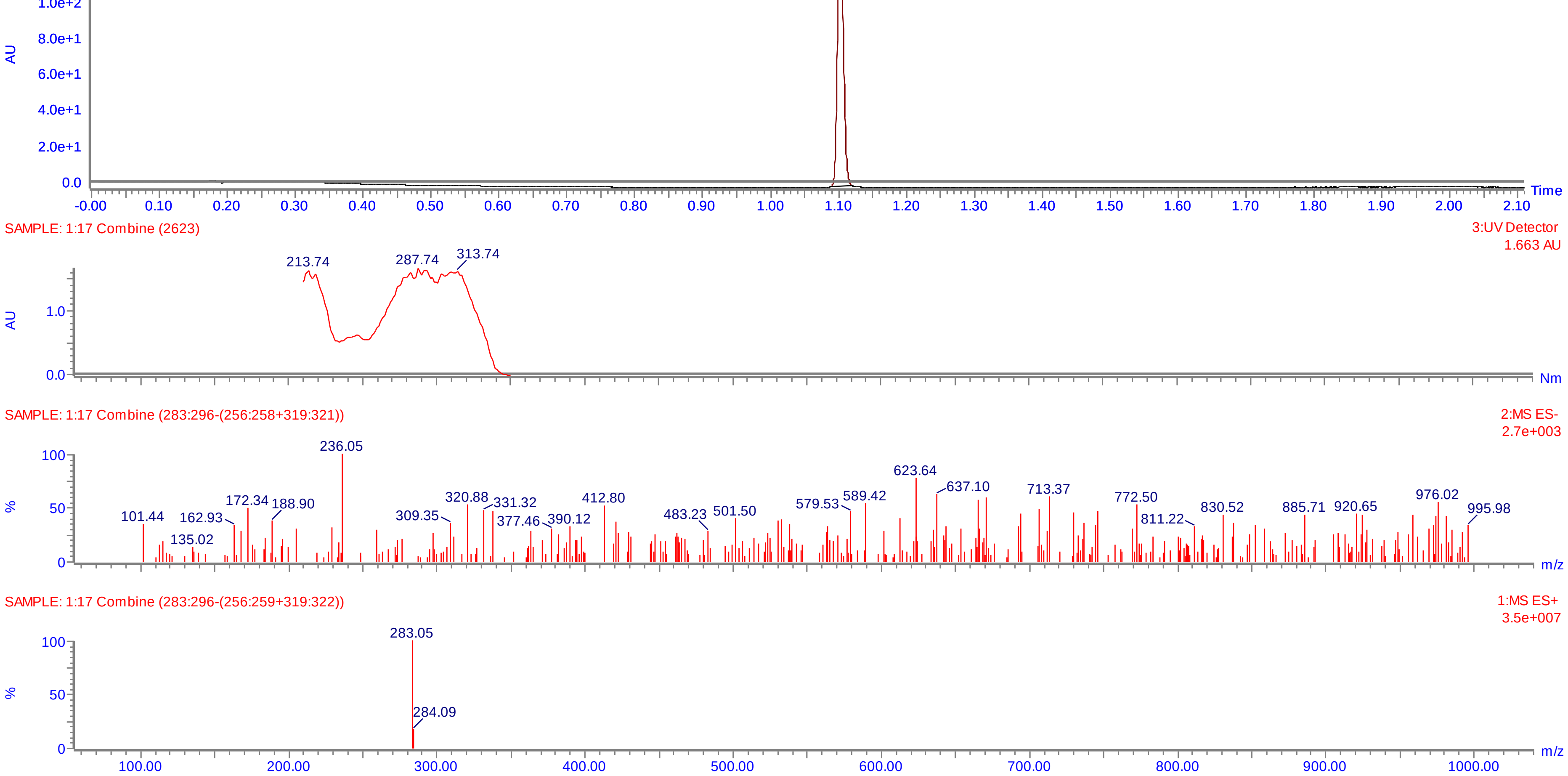
UV Detector: TIC
(1)
100%
1.10
1.45e+2
Range: 1.485e+2
AU
Time
SAMPLE: 1:17 Combine (2623)
3:UV Detector
1.663 AU
213.74
287.74
313.74
Nm
SAMPLE: 1:17 Combine (283:296-(256:258+319:321))
2:MS ES-
2.7e+003
236.05
623.64
637.10
713.37
976.02
995.98
m/z
SAMPLE: 1:17 Combine (283:296-(256:259+319:322))
1:MS ES+
3.5e+007
283.05
284.09
m/z

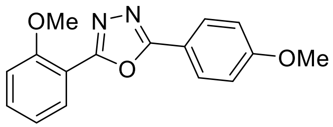

5l

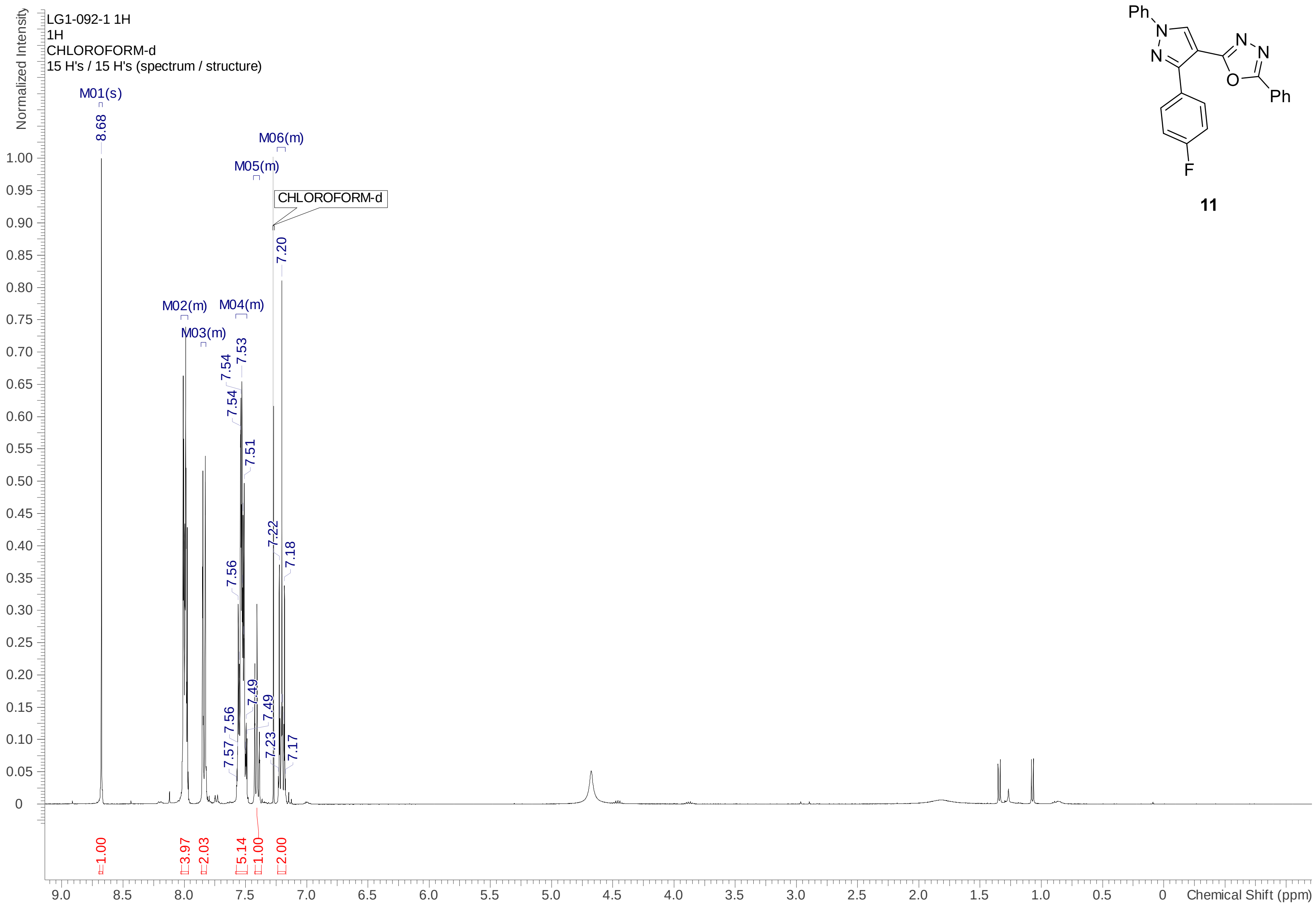

LG1-092-1 1H
1H
CHLOROFORM-d
15 H's / 15 H's (spectrum / structure)
Normalized Intensity
M01(s)
M02(m)
M03(m)
M04(m)
M05(m)
M06(m)
CHLOROFORM-d
8.68
7.54
7.53
7.54
7.51
7.56
7.22
7.18
7.20
7.49
7.49
7.56
7.57
7.23
7.17
1.00
3.97
2.03
5.14
1.00
2.00
Chemical Shift (ppm)
Ph
N
N
N
N
O
Ph
F

11

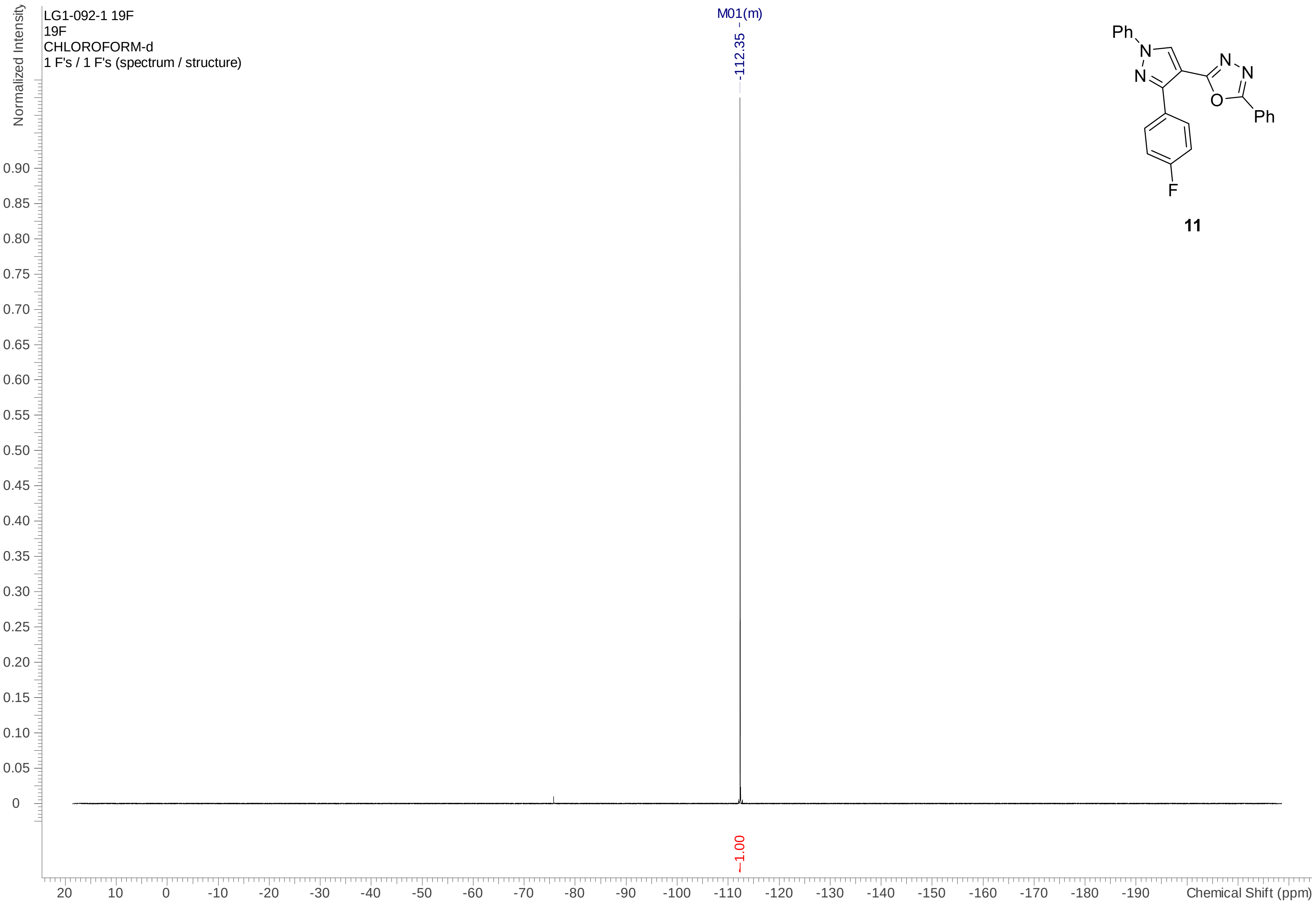

LG1-092-1 19F
19F
CHLOROFORM-d
1 F's / 1 F's (spectrum / structure)
M01(m)
-112.35
1.00
Normalized Intensity
0.90
0.85
0.80
0.75
0.70
0.65
0.60
0.55
0.50
0.45
0.40
0.35
0.30
0.25
0.20
0.15
0.10
0.05
0
20 10 0 -10 -20 -30 -40 -50 -60 -70 -80 -90 -100 -110 -120 -130 -140 -150 -160 -170 -180 -190
Chemical Shift (ppm)
Ph
N
N
N
N
O
Ph
F
11

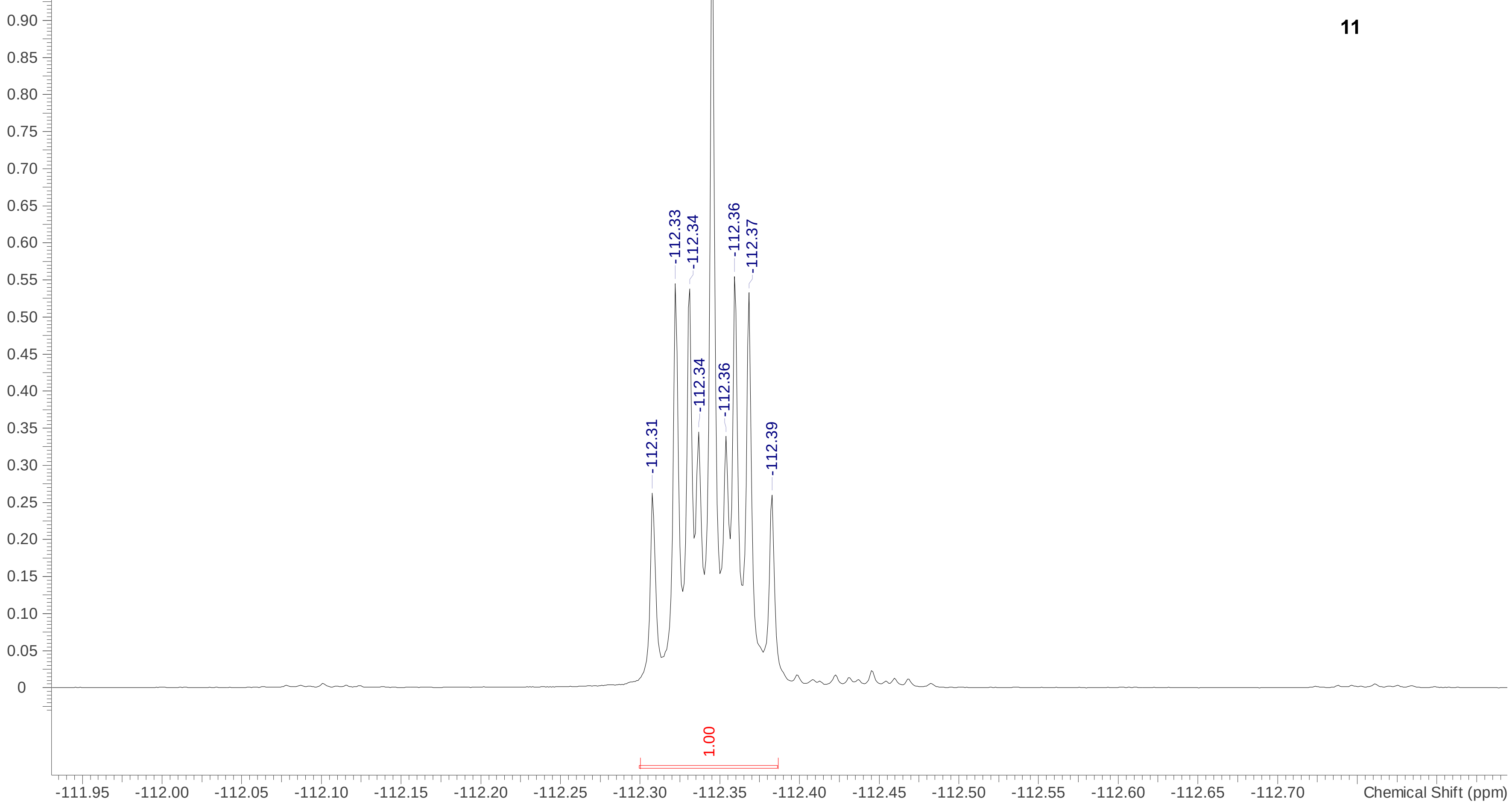
LG1-092-1 19F
19F
CHLOROFORM-d
1 F's / 1 F's (spectrum / structure)
M01(m)
-112.35
-112.33
-112.34
-112.36
-112.37
-112.34
-112.36
-112.31
-112.39
1.00
Normalized Intensity
Chemical Shift (ppm)

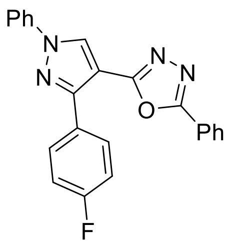
Ph
N
N
N
N
O
Ph
F

11

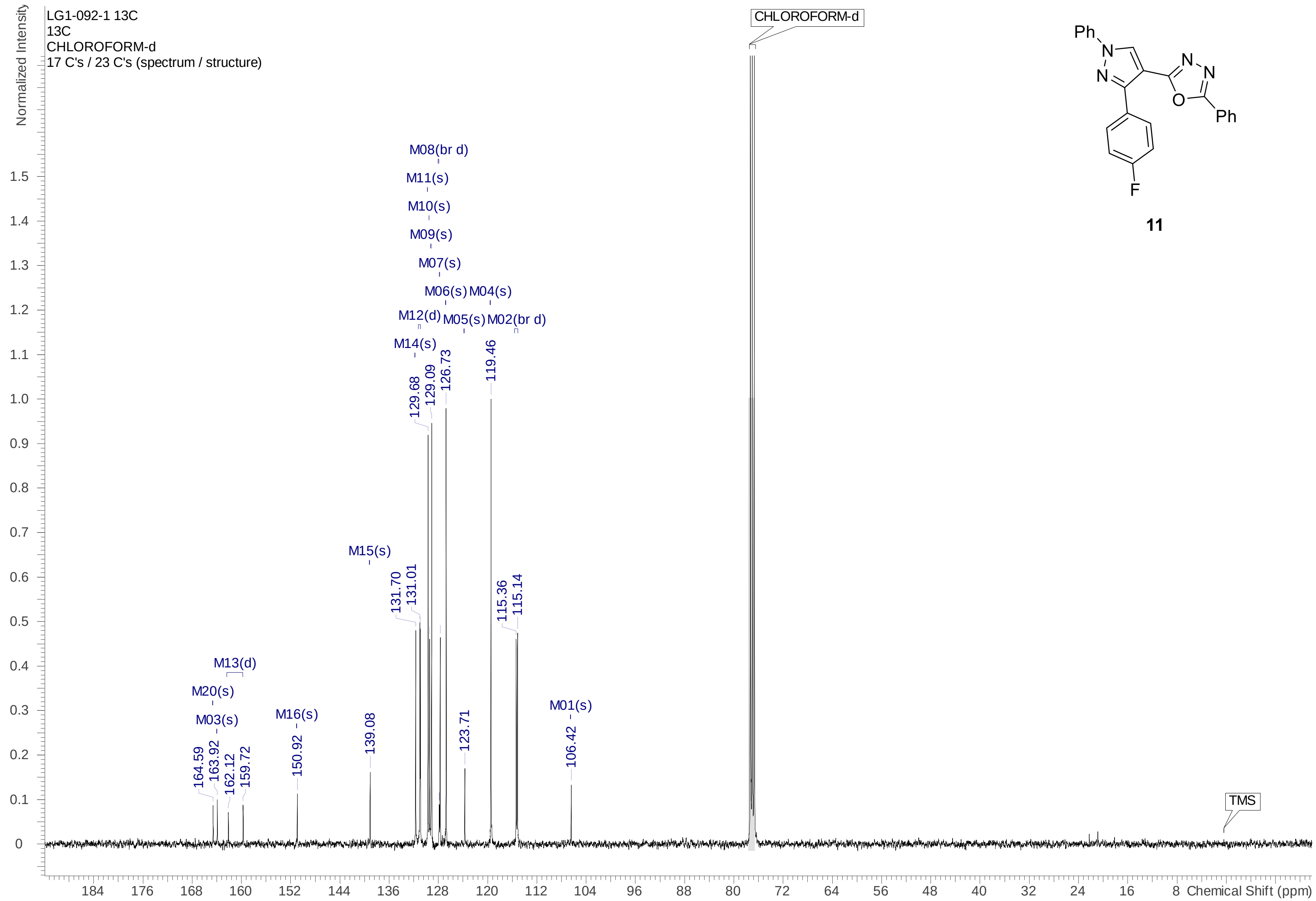

LG1-092-1 13C
13C
CHLOROFORM-d
17 C's / 23 C's (spectrum / structure)
Normalized Intensity
CHLOROFORM-d
TMS
M08(br d)
M11(s)
M10(s)
M09(s)
M07(s)
M06(s) M04(s)
M12(d) M05(s) M02(br d)
M14(s)
M15(s)
M13(d)
M20(s)
M03(s)
M16(s)
M01(s)
164.59 163.92 162.12 159.72 150.92 139.08 131.70 131.01 129.68 129.09 126.73 123.71 119.46 115.36 115.14 106.42
Chemical Shift (ppm)
Ph
N
N
N
N
O
Ph
F

11

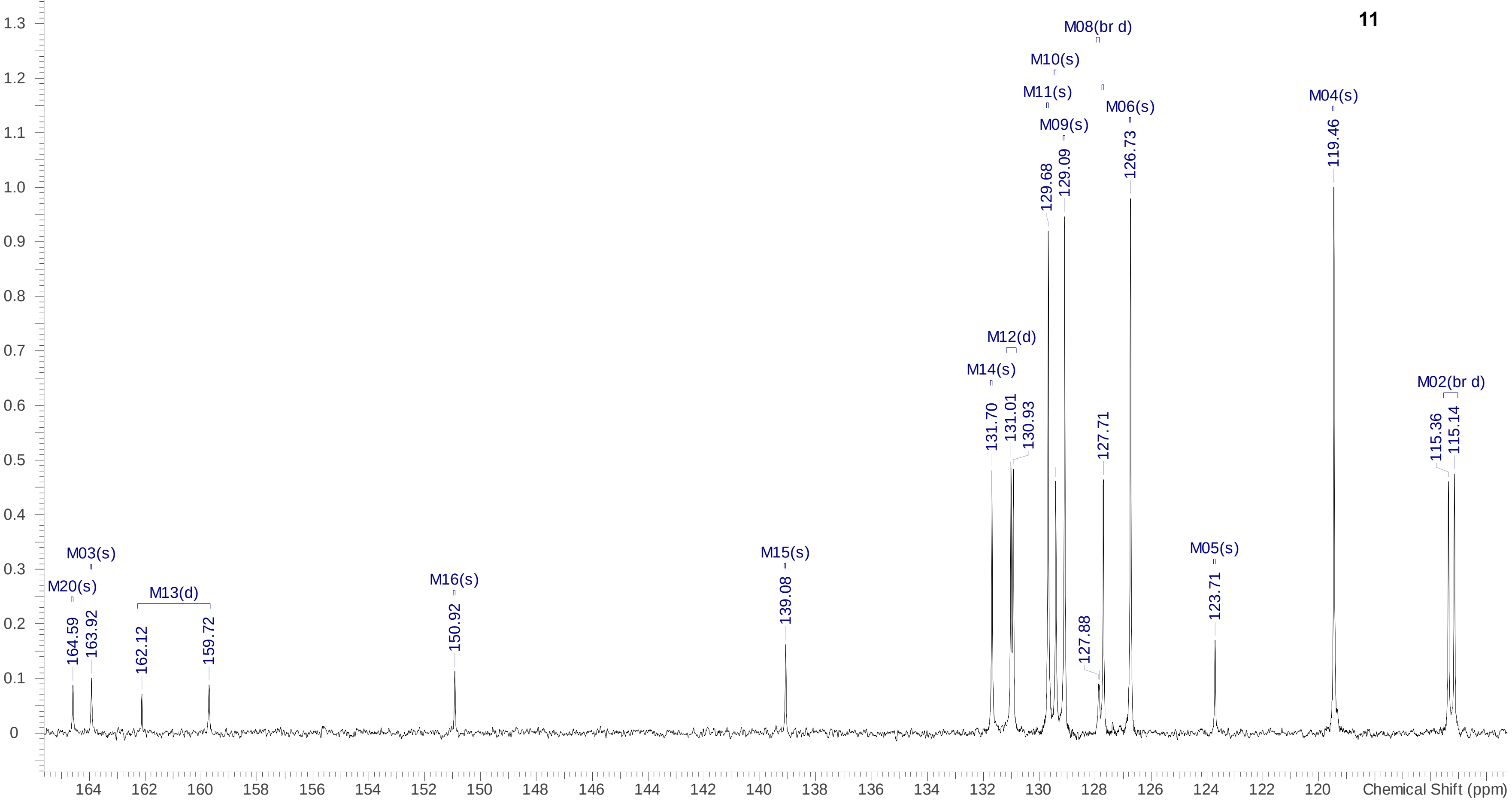
LG1-092-1 13C
13C
CHLOROFORM-d
17 C's / 23 C's (spectrum / structure)
Normalized Intensity
Chemical Shift (ppm)
M20(s) 164.59
M03(s) 163.92
M13(d) 162.12 159.72
M16(s) 150.92
M15(s) 139.08
M14(s) 131.70
M12(d) 131.01 130.93
M11(s) 129.68
M10(s)
M09(s) 129.09
M08(br d) 127.88 127.71
M06(s) 126.73
M05(s) 123.71
M04(s) 119.46
M02(br d) 115.36 115.14

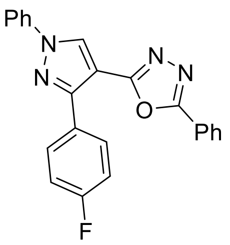
Ph
N
N
N
N
O
Ph
F

11

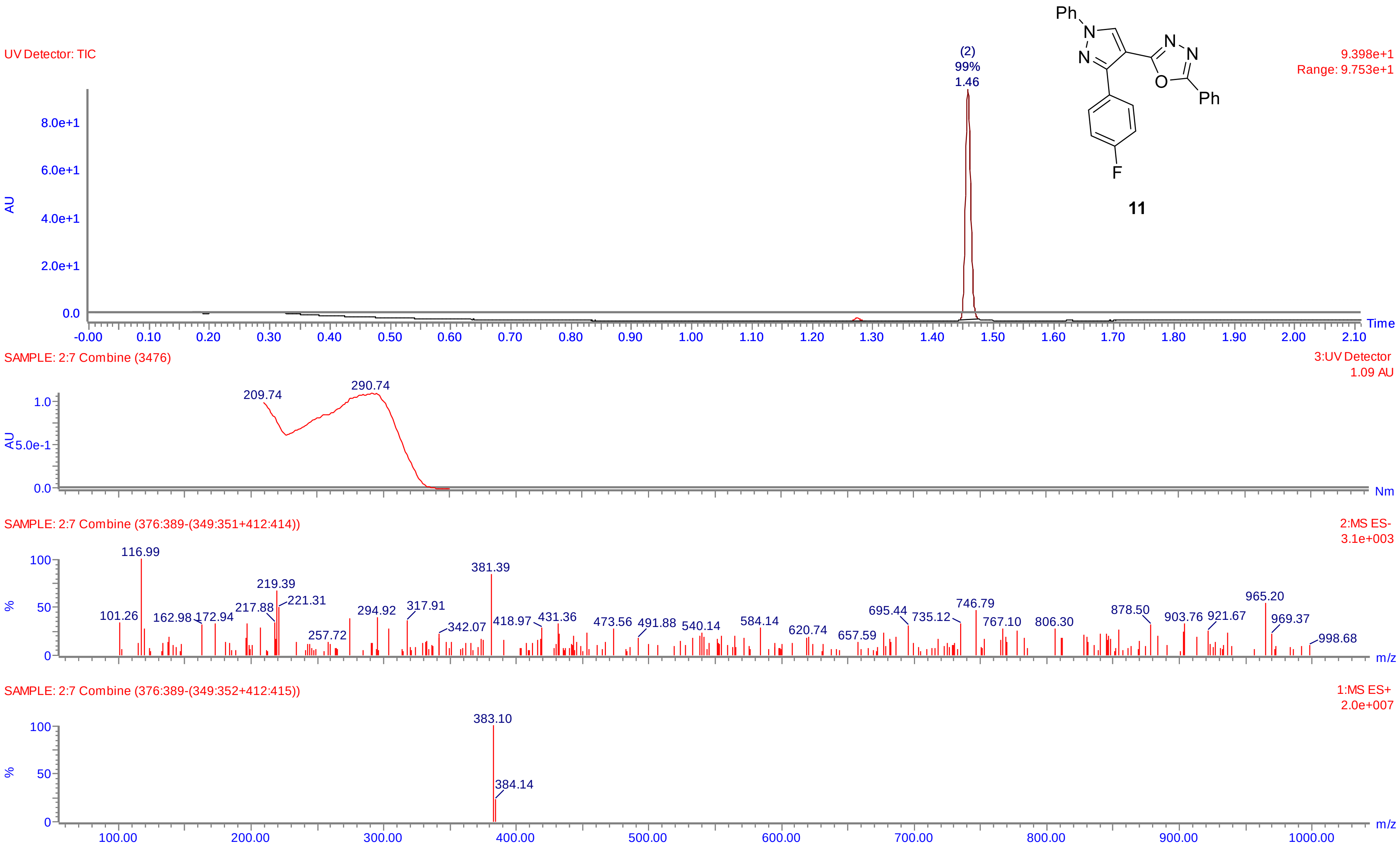
UV Detector: TIC
9.398e+1
Range: 9.753e+1
(2)
99%
1.46
AU
Time
SAMPLE: 2:7 Combine (3476)
3:UV Detector
1.09 AU
209.74
290.74
Nm
SAMPLE: 2:7 Combine (376:389-(349:351+412:414))
2:MS ES-
3.1e+003
116.99
381.39
219.39
221.31
101.26
162.98
172.94
217.88
257.72
294.92
317.91
342.07
418.97
431.36
473.56
491.88
540.14
584.14
620.74
657.59
695.44
735.12
746.79
767.10
806.30
878.50
903.76
921.67
965.20
969.37
998.68
m/z
SAMPLE: 2:7 Combine (376:389-(349:352+412:415))
1:MS ES+
2.0e+007
383.10
384.14
m/z
Ph
N
N
N
N
O
Ph
F

11

9. LCMS and HRMS Parameters

2 minute UPLC-MS solvents and gradients

For formic acid runs, the solvents employed were:

A. 0.1% v/v solution of formic acid in water
B. 0.1% v/v solution of formic acid in acetonitrile

The gradient was as follows:

Time (min)	Flow rate (mL/min)	% A	% B
0.0	1	97	3
1.5	1	3	97
1.9	1	3	97
2.0	1	98	2

For high pH runs, the solvents employed were:

A. 10 mM ammonium bicarbonate in water adjusted to pH 10 with ammonia solution
B. Acetonitrile

The gradient was as follows:

Time (min)	Flow rate (mL/min)	% A	% B
0.0	1	100	100
0.05	1	100	0
1.5	1	3	97
1.9	1	3	97
2.0	1	100	0

UPLC-HRMS solvents and gradients

For 10 minute formic acid runs, the solvents employed were:

A. 0.1% v/v solution of formic acid in water
B. 0.1% v/v solution of formic acid in acetonitrile

The gradient was as follows:

Time (min)	Flow rate (mL/min)	% A	% B
0.0	0.8	95	5
8.5	0.8	7	93
9.0	0.8	7	93
9.5	0.8	95	5
10.0	0.8	95	5

For 20 minute high pH runs, the solvents employed were:

A. 10 mM ammonium bicarbonate in water adjusted to pH 10 with ammonia solution
B. Acetonitrile

The gradient was as follows:

Time (min)	Flow rate (mL/min)	% A	% B
0.0	0.8	99	1
0.5	0.8	99	1
17.0	0.8	10	90
18.5	0.8	10	90
19.0	0.8	99	1
20.0	0.8	99	1
